# Supplementary material for: The Position-Reputation-Information (PRI) scale of individual prestige
Source: PLoS One. 2020 Jun 25;15(6):e0234428. doi: 10.1371/journal.pone.0234428 (PMC7316272; doi:10.1371/journal.pone.0234428)
Supplement: S1 Appendix — Complete description of methods used and additional supporting results, in Word DOCX format. (DOCX) [file pone.0234428.s001.docx]

**The Position-Reputation-Information (PRI) scale of individual prestige**

Richard E.W. Berl^1*^, Alarna N. Samarasinghe^2^, Fiona M. Jordan^2,3^, Michael C. Gavin^1,3^

^1^ Department of Human Dimensions of Natural Resources, Colorado State University, Fort Collins, Colorado, United States of America

^2^ Department of Anthropology and Archaeology, University of Bristol, Bristol, United Kingdom

^3^ Max Planck Institute for the Science of Human History, Jena, Germany

* Corresponding author

E-mail: rewberl@colostate.edu (REWB)

# S1 Appendix: Supplementary methods

## Study 1: Scale construction

### Item generation

In the development of this scale, we used a combination of deductive and inductive methods to collect the items most relevant to the concept of individual prestige. This methodological approach incorporated emic, operational determinants of prestige from a real-world Western context, as well as shared items from previous scales, in order to evaluate all possible components of a prestige scale concurrently. We sampled items from a salience analysis of responses to a free listing task, from existing attitudinal scales in the literature, and from responses to a pilot study investigating sociolinguistic prestige. We favored the use of inductive methods, specifically the free listing task, because they are generalizable and facilitate replication and extension to other contexts and cultures.

Free listing is a tool from cultural domain analysis used to elicit responses on a particular classification of knowledge [1–3]. The task conducted as part of this study consisted of a survey in which participants responded to the following three prompts, in order:

1. *List all of the words or phrases that you can think of that are related to “prestige.”*
2. *List all of the words or phrases that you can think of that describe “prestigious” people.*
3. *List all of the characteristics that you can think of that make a person “prestigious.”*

Responses were limited to 2 minutes per question. We allowed repetition of terms from prior questions, but participants could not refer back to previous responses. We recruited participants for this task through advertisements in local undergraduate courses (*n* = 6 US) and social media networks (*n* = 42 US, 20 UK), for a final sample of 68 participants. We compensated undergraduate students for their participation and social media participants engaged voluntarily. Participants ranged in age from 18 to 50 (*M* = 28.9, *SD* = 7.3), with 18 that identified as male and 50 that identified as female. All participants were native English speakers. All participants self-identified as white, except for one person of mixed ethnicity from the US and one person of color from the UK. Participants came from a variety of backgrounds with respect to the size of their childhood settlement and educational attainment, but for occupation most were either students (25.0%) or were in management or professional positions (26.5%), with others in service (11.8%) and sales (5.9%) positions.

After obtaining the three free lists of items from each participant, we grouped items for common meaning, reducing the pool of unique items from 717 to 303. Generally, this procedure consisted of replacing multi-word phrases with single-word synonyms and converting words to adjective form (e.g. “lots of education” to “educated” and “influence” to “influential”). We left given terms as-is if their intended meaning was ambiguous. On the whole, groupings were done with the intent of minimal replacement, so as to allow participants to speak for themselves, and all co-authors verified the groupings. We then calculated a salience value for each of the 303 items using Smith’s S [4], which takes into account both the frequency of an item’s occurrence across lists and order of occurrence within lists. From a scree plot of the items by their salience values, we chose the cutoff near the inflection point at the highest local proportional drop in salience (0.0148) to capture the set of most salient items (**Fig S1.1**) [1]. The items retained from this exercise were: *wealthy*, *high social status*, *powerful*, *respected*, *educated*, *hardworking*, and *successful*.


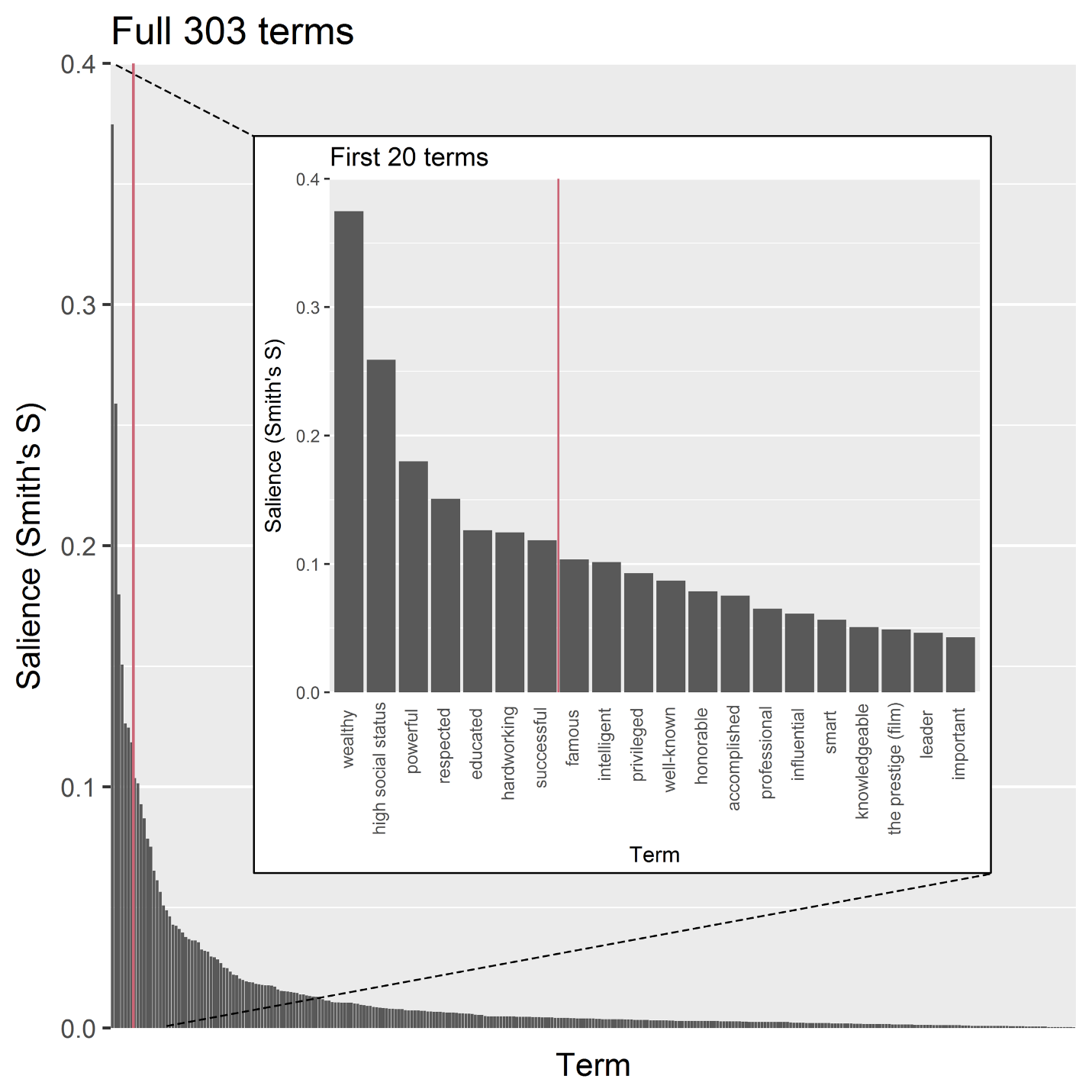


**Fig S1.1. Scree plot of free list items by salience value.** Chosen cutoff includes *successful* (*S* = 0.1185) and items of higher salience and excludes *famous* (*S* = 0.1037) and items of lower salience.

Given the use of attitudes toward regional accents as a measurement tool in this study, and in the interest of full coverage of the domain of interest (i.e. content validity), we chose to supplement the pool of potential scale items by reviewing items used in established scales of language attitudes that incorporated a prestige or status dimension. The two scales we selected for this purpose were the Speech Dialect Attitudinal Scale (“SDAS”: [5]) and its revised version (“SDAS-R”: [6]) and the Speech Evaluation Instrument (“SEI”: [7]) and its short form version (“SEI-S,” as used by [8]). The following items were represented in some form within both scales under a dimension of “prestige,” “status,” or “competence” and were therefore retained: *wealthy*, *high social status*, *educated*, and *intelligent* (as a note, we collapsed *upper class* into the broader *high social status* and *literate* into *educated*). These items agreed closely with those used in other sociolinguistic studies for these dimensions [9], and therefore can be regarded as representative of the literature. We also collected items from the selected scales to represent two other domains commonly used in speech evaluation studies [9,10]: “solidarity” and “dynamism.” We included these domains, which are unrelated to prestige but similarly positively valenced, to assess the ability of prestige items to represent prestige itself and not merely a positive evaluation of the speaker (i.e. discriminant validity). The additional items we selected were: *friendly*, *kind*, *good natured*, *warm*, and *comforting* for the “solidarity” dimension, and *aggressive*, *active*, *confident*, and *enthusiastic* for the “dynamism” dimension. One item, *clear*, was also initially included within “dynamism,” but we later removed it from analyses due it clustering more closely with items in other dimensions.

A third and final source of items was a previously unpublished pilot study that we conducted on speech, accent, and prestige in October and November of 2015. The sample of this pilot study consisted of 100 US and 44 UK participants (undergraduate and graduate/postgraduate students) ranging in age from 18 to 64 years (*M* = 21.8, *SD* = 5.3). Of the participants, 47 identified as male and 95 as female. The majority of participants, 141, identified as native English speakers, with 2 non-native speakers. Participants were asked to rate two speakers—one with a locally standard accent (US or UK) and the other with a nonstandard accent—on 15 attitudinal items using a 7-point Likert-type scale. The items in this pilot study were also drawn from prior linguistic studies. Following exploratory factor analysis and the sequential elimination of items following the same criteria described below for the present study, as well as examining inter-item correlations with a *prestigious* item to find the most closely associated items, we retained the following items from the pilot study: *hardworking*, *reputable*, *intelligent*, and *ambitious*.

The combined pool of items retained from all three sources (**Table 1**) were then used in the scale construction and scale evaluation studies to establish and verify the scale.

### Questionnaire construction and administration

We developed an online questionnaire for use in the United States and United Kingdom using the pool of attitudinal items retained from the item generation stage.

For the stimulus, we presented each participant with four audio recordings of the same short passage (approximately 30 seconds in length), each read by a speaker with a different regional accent of English, and asked them to rate each speaker on all 20 attitudinal items using a 7-point Likert-type scale from low (1) to neutral (4) to high (7). All recordings consisted of the first paragraph of *Comma Gets a Cure* (see Acknowledgements), a passage which uses the Wells Standard Lexical Sets for English [11] to highlight the most differentiable elements of accents.

We selected accents from the dialect regions defined by Labov et al. [12] for the United States and Shackleton [13] for the United Kingdom. The US-based accents in this study were American West and Inland South and the UK-based accents were Received Pronunciation (“RP”) and Northwest England. We recorded a speaker from urban Colorado to represent the American West accent, and for the other three accents we used recordings under license from the International Dialects of English Archive (“IDEA”; see Acknowledgements). A full list of the recordings used and speaker demographics is available in **S5 Table**.

The IDEA data sources predominately represented white male speakers. As a result of controlling for speaker demographics and audio quality from the available recordings, our speakers all self-identified as white males ranging from 42 to 59 years old. In the sociolinguistic sense, American West (which phonologically is in the spectrum of the “General American” accent) and Received Pronunciation represent standard or “high-prestige” variants within the US and UK, respectively, and Inland South and Northwest England are nonstandard “low-prestige” variants [10,14,15]. The American West and RP speakers used for this study held university degrees and the Inland South and Northwest England speakers did not. The American West and RP speakers were employed in professional teaching occupations and the Northwest England and Inland South speakers were employed in skilled trades. Therefore, their educational and occupational attainment matched the indexical class and status associated with their accents. We presented all participants with all four recordings, regardless of their location.

Prior to being presented with the recordings or giving attitudinal responses, participants each completed a triad test [1] with a lambda-3 balanced incomplete block design [16] for the 11 prestige domain items, resulting in 55 triadic comparisons per participant. In each comparison, participants chose which of the three items was perceived to be least like the others, thereby creating a pair of like items. This could be used to assess whether the perception of the structure of prestige items was consistent beyond the sociolinguistic context of the prestige of regional accents.

We collected a number of demographic variables from participants, to be able to examine any systematic differences in responses. The demographic variables chosen were: country, age, gender, ethnicity, locality size, English proficiency, education, occupation, and income. Each variable and its levels are described in detail in **S4 Metadata** and their distributions within the sample are displayed in **Fig S1.4** in comparison with those of the subsequent scale evaluation study.

We collected data in May and June 2016 using online surveys implemented on SurveyMonkey and distributed using social media (*n* = 5 US, 2 UK), the Amazon Mechanical Turk and TurkPrime [17] platforms (*n* = 148 US), and the Prolific platform (*n* = 153 UK), for a final sample of 308 (153 US, 155 UK). There were 5 participants (4 US, 1 UK) that completed the triad test but not the attitudinal speech evaluation, so the final sample for the attitudinal data was 303 (149 US, 154 UK). There were otherwise no missing attitudinal or triad data, as we required participants to complete every item in order to receive payment.

### Exploratory factor analysis

First, we checked the data for conformity to the assumptions of exploratory factor analysis (“EFA”). Though strict multivariate normality is not required for exploratory or confirmatory methods using categorical models, and violations are allowable under continuous models (i.e. maximum likelihood) if measurement invariance is established [18], we found that the distribution of responses to the attitudinal items was not multivariate normal, with *p* ≅ 0 for Mardia’s test [19,20], the Henze-Zirkler test [21], and Royston’s test [22,23]. We identified multivariate outliers using adjusted chi-square quantile-quantile plots of Mahalanobis distances and removed one participant (from the US sample) with extreme outlier values.

We then assessed the distributions of attitudinal items for approximate univariate normality, as well as for acceptable values of skewness and kurtosis. Following Bulmer [24], absolute values of skewness below 0.5 indicated an approximately symmetric distribution, values between 0.5 and 1.0 were considered moderately skewed, and values above 1.0 were highly skewed. According to the findings of West et al. [25] and Curran et al. [26], issues of bias due to non-normality may result from the analysis of data distributed with absolute skewness values above 2.0 or kurtosis values above 7.0. We found individual variables to be approximately normal and values of skewness (*M* = -0.242, *SD* = 0.407) and kurtosis (*M* = -0.536, *SD* = 0.476) to be within acceptable ranges.

We evaluated linear relationships between items and their factorability by examining inter-item correlations, using the Kaiser-Meyer-Olkin (“KMO”) test of sampling adequacy [27], with values greater than or equal to 0.50 considered suitable [28–30], and using Bartlett’s test of sphericity [31] to test whether the correlation matrix was factorable. We calculated a polychoric correlation matrix because attitudinal items were measured using an ordinal scale [32]. Following Savalei [33], no adjustments were made to zero frequency cells in the bivariate tables. A large proportion of inter-item correlations (73/210, or 34.8%) were above 0.50, indicating the presence of linear relationships. KMO values were well above 0.50 for all variables (overall = 0.946, *M* = 0.935, *SD* = 0.039) and the result of the Bartlett’s test was highly significant (*p* ≅ 0), together indicating suitable factorability.

Lastly, we evaluated whether our sample sizes were adequate, using the guidelines of having a total sample size of at least $\frac{p\left( p-1 \right)}{2}$ [34], where $p$ is the number of items or variables, and a subjects-to-variables ratio of at least 10:1 [28] or 20:1 [30]. The sample size for this study (after outlier removal) was 302, which (at $p=20$) exceeds the suggested minimum of 190, and the subjects-to-variables ratio was 15.1:1, which lies above the recommendation of 10:1 and below 20:1.

We then conducted exploratory factor analysis for the purpose of exploring the structure and dimensionality of the prestige construct. Our analyses used a three-stage robust diagonally weighted least squares estimation technique (weighted least squares, mean and variance adjusted, or “WLSMV”) due to its suitability for use on ordinal data with an adequate number of categories [34–36]. We used a conservative oblimin (oblique) factor rotation method to allow for potential intercorrelations between factors, which may be expected in real-world attitudinal data [37].

We eliminated items sequentially, first to remove items that had poor value in discriminating the prestige domain from the other two domains included—solidarity and dynamism—and then to determine the most parsimonious structure within the prestige domain. Items needed to meet all of the following acceptance criteria to be retained: *a*) primary factor loading with an absolute value > 0.32; *b*) cross-loadings with absolute values < 0.32; *c*) gap between primary and cross-loadings > 0.2; and *d*) communality > 0.4 [38]. We re-evaluated the optimal number of factors at each step using the parallel analysis with comparison data method of Ruscio & Roche [39].

Through this process, we obtained the overall factor structure for the attitudinal items across all three domains (**Table S1.1A**; **Fig 1**), as well as the internal factor structure of the prestige domain items (**Table S1.1B**; **Fig 2**). Using EFA, items within the prestige domain were partitioned into three factors: *wealthy*, *powerful*, and *high social status* in the first factor, hereafter referred to as “position”; *reputable* and *respected* in the second factor, referred to as “reputation”; and *educated* and *intelligent* in the third factor, referred to as “information.” We therefore denote the resulting factor structure as Position-Reputation-Information, or “PRI.”

After completing EFA using the attitudinal data, we then repeated the process using the data from the triad test as a second, parallel source of information on the structure of the prestige construct absent the embedded sociolinguistic context. Since the pairings in the triad data are represented as a series of dichotomous observations, we calculated a tetrachoric correlation matrix [40], using a correction of 0.5 for empty bivariate cells (following Savalei [33]) and eigenvector smoothing to ensure the matrix was positive definite. We chose related methods to maximize comparability between the attitudinal and triad data sources. We used a non-robust weighted least squares (“WLS”) estimator with standard parallel analysis and identical acceptance criteria to those used for the EFA of the attitudinal data described above.

The inter-item correlations between triad items had 4/55 (7.3%) above 0.50. The overall KMO value was 0.364 (*M* = 0.368, *SD* = 0.160), with the lowest individual values being *successful* at 0.075 and *powerful* at 0.157, and the highest being *wealthy* at 0.585. While the result of the Bartlett’s test was highly significant (*p* ≅ 0), it is also dependent upon sample size, which was reasonably large (*n* = 308). Taken together, these results suggested that factorability could be poor due to the nature of how the data were represented; specifically, the triadic comparisons generated a matrix with a large amount of “missing” data, as only 3 items in each observation (out of 11 total) had values. The sample size for the triad data was much higher than the suggested minimum of 45 in this case (given the lower number of items), and the subjects-to-variables ratio was 30.8:1, which is above both recommended values. We obtained the internal factor structure for the prestige domain items in the triad data (**Table S1.1C**) using the EFA methods described. The structure closely resembled the attitudinal results in all respects except that *powerful* was dropped from the position factor due to negative loadings and low communality.

**Table S1.1. Factor loadings and communalities from exploratory factor analysis of attitudinal and triad data.** Values are from analyses of: **(A)** all items from attitudinal data over the three domains; **(B)** internal prestige domain items from attitudinal data; and **(C)** internal prestige domain items from triad data. All cases show the items remaining after eliminating prestige domain items that failed to meet acceptance criteria, and after removing *prestigious* from **(B)** and **(C)**. *Note:* Factor loadings with absolute value < 0.20 are suppressed.

| **(A)** |  |  |  |  |
| --- | --- | --- | --- | --- |
|  | **PRESTIGE** | **SOLIDARITY** | **DYNAMISM** | communality |
| *educated* | 0.962 | -0.201 |  | 0.852 |
| *intelligent* | 0.921 |  |  | 0.806 |
| *high social status* | 0.893 |  |  | 0.862 |
| *successful* | 0.880 |  |  | 0.805 |
| *prestigious* | 0.861 |  |  | 0.781 |
| *wealthy* | 0.847 |  |  | 0.818 |
| *respected* | 0.736 | 0.237 |  | 0.638 |
| *powerful* | 0.734 |  | 0.260 | 0.727 |
| *reputable* | 0.694 | 0.278 |  | 0.573 |
| *warm* |  | 0.893 |  | 0.787 |
| *friendly* |  | 0.890 |  | 0.795 |
| *kind* |  | 0.865 |  | 0.749 |
| *good-natured* |  | 0.837 |  | 0.705 |
| *comforting* |  | 0.808 |  | 0.660 |
| *enthusiastic* | 0.278 | 0.442 | 0.383 | 0.460 |
| *aggressive* |  | -0.441 | 0.409 | 0.394 |
| *active* | 0.341 |  | 0.394 | 0.364 |
|  |  |  |  |  |
| **(B)** |  |  |  |  |
|  | **Position** | **Reputation** | **Information** | communality |
| *wealthy* | 0.935 |  |  | 0.862 |
| *powerful* | 0.819 |  |  | 0.688 |
| *high social status* | 0.771 |  |  | 0.872 |
| *reputable* |  | 0.824 |  | 0.729 |
| *respected* | 0.238 | 0.590 |  | 0.668 |
| *educated* |  |  | 0.893 | 0.880 |
| *intelligent* |  |  | 0.845 | 0.826 |
|  |  |  |  |  |
| **(C)** |  |  |  |  |
|  | **Position** | **Reputation** | **Information** | communality |
| *wealthy* | 0.776 |  |  | 0.571 |
| *high social status* | 0.657 |  |  | 0.497 |
| *reputable* |  | 0.820 |  | 0.655 |
| *respected* |  | 0.705 |  | 0.532 |
| *intelligent* |  |  | 0.962 | 0.891 |
| *educated* |  |  | 0.832 | 0.749 |

### Cluster analysis

Following the EFA for both the attitudinal data and the triad data, we also elected to conduct cluster analysis on the items in both data sets to compare results with the EFA findings on the internal structure of the prestige construct. Though the outputs of EFA and cluster analysis are qualitatively similar, the two methods have substantively different goals (dimensionality reduction to latent constructs versus classification to subgroups, respectively) and algorithms. We chose the Partitioning Around Medoids (“PAM”) method [41], a type of *k*-medoids algorithm in the *k*-means family, due to its flexibility in accommodating various dissimilarity measures and its robustness against outliers. For the attitudinal data, we used Manhattan distances rather than Euclidean due to their suitability for ordinal data [41]. Visual examination of the Manhattan distance matrix using multidimensional scaling suggested that the attitudinal data were amenable to cluster analysis.

We eliminated items sequentially to remove items with poor discriminant value and to determine the internal prestige structure, retaining items which had a positive silhouette width of at least 0.1 and the removal of which did not substantially improve the overall clustering structure (as measured by average silhouette width of the solution). The silhouette width of an item represents the relative consistency of that item within its cluster. At each step, we used the Duda-Hart test [42] to determine whether more than one cluster was supported and the number of clusters was determined by the highest average silhouette width.

The PAM method resulted in a 2-cluster solution for all attitudinal items (**Fig S1.2**) and a 3-cluster solution for the internal prestige domain items (**Fig S1.3A**). The average silhouette width of the 2-cluster solution for all items was 0.428, while the next highest, at 4 clusters, was 0.294. For the internal prestige domain items, the average silhouette width of the 3-cluster solution was 0.282, with 0.300 for 2 clusters. However, the Dunn index, or the ratio of minimum inter-cluster distance to maximum intra-cluster distance [43], was 1.052 for the 3-cluster internal solution and 0.882 for the 2-cluster internal solution, indicating that the 3-cluster solution has better validity. These results support the 3-cluster solution for the internal prestige domain items and this solution matches exactly the PRI structure found through EFA.

Applying the PAM method to the triad data gave similar results, with the highest average silhouette width overall (0.388) found for a 3-cluster solution that matched the PRI structure (**Fig S1.3B**). However, we reached this solution by eliminating the *hardworking* and *ambitious* items based on information from the EFA showing their poor fit within the prestige domain. The triadic comparisons included only prestige domain items so, within the context of the triad data alone, this information about the ability to discriminate from other domains would be unavailable. Additionally, the Dunn index suggested better support for this 3-cluster solution (1.112) than for a 4-cluster solution that included *hardworking* and *ambitious* (1.051). These results are consistent with what we found from the attitudinal data and replicate the PRI structure in the best-fitting solution.


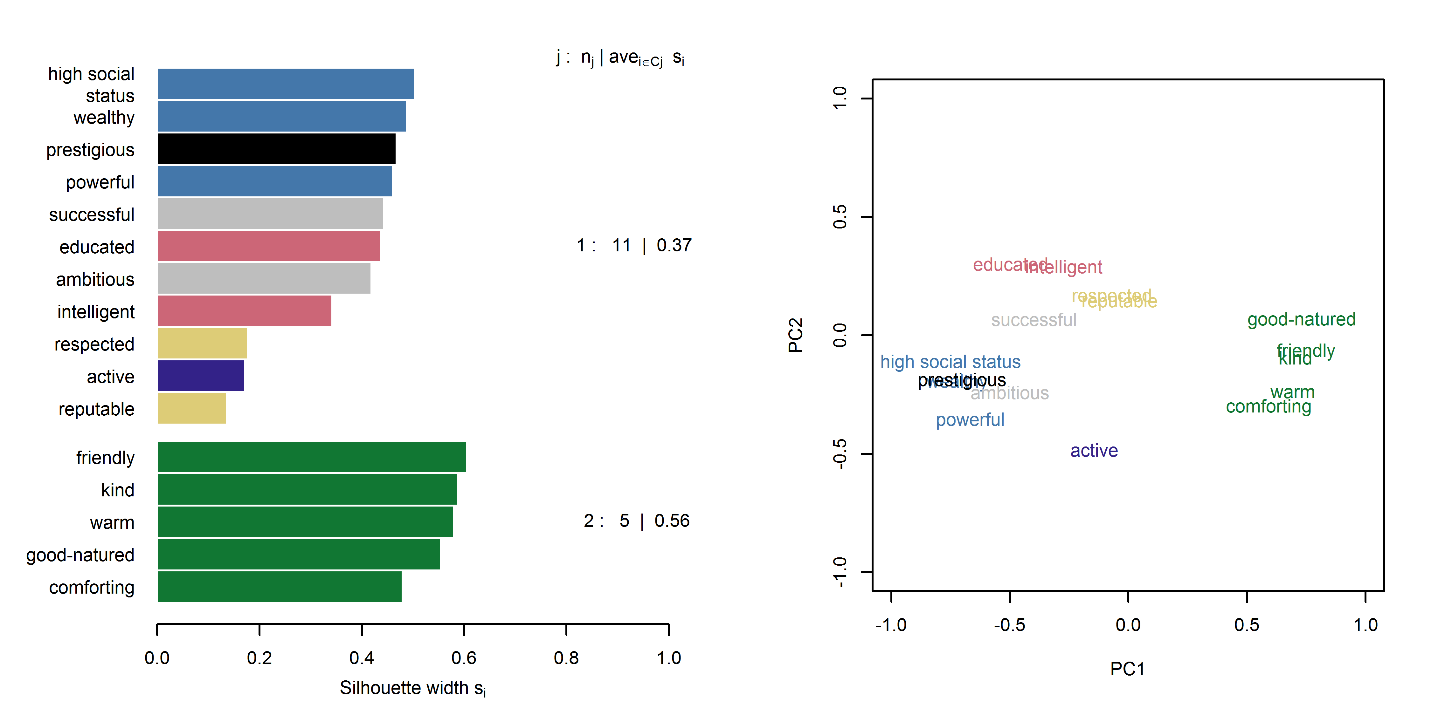


**Fig S1.2. Silhouette and multidimensional scaling plots of the clustering of prestige, solidarity, and dynamism attitudinal items.** Position, reputation, and information items are shown in light blue, gold, and light red, respectively. Other prestige items are shown in black (*prestigious*, not used in scale) and grey (later dropped from internal prestige structure shown in **Fig S1.3A**). Solidarity items are in green. The remaining dynamism item (*active*) is in purple. To the right of each cluster is the number of items in that cluster and its average silhouette width. Silhouette width values represent the relative consistency of each item within its cluster.


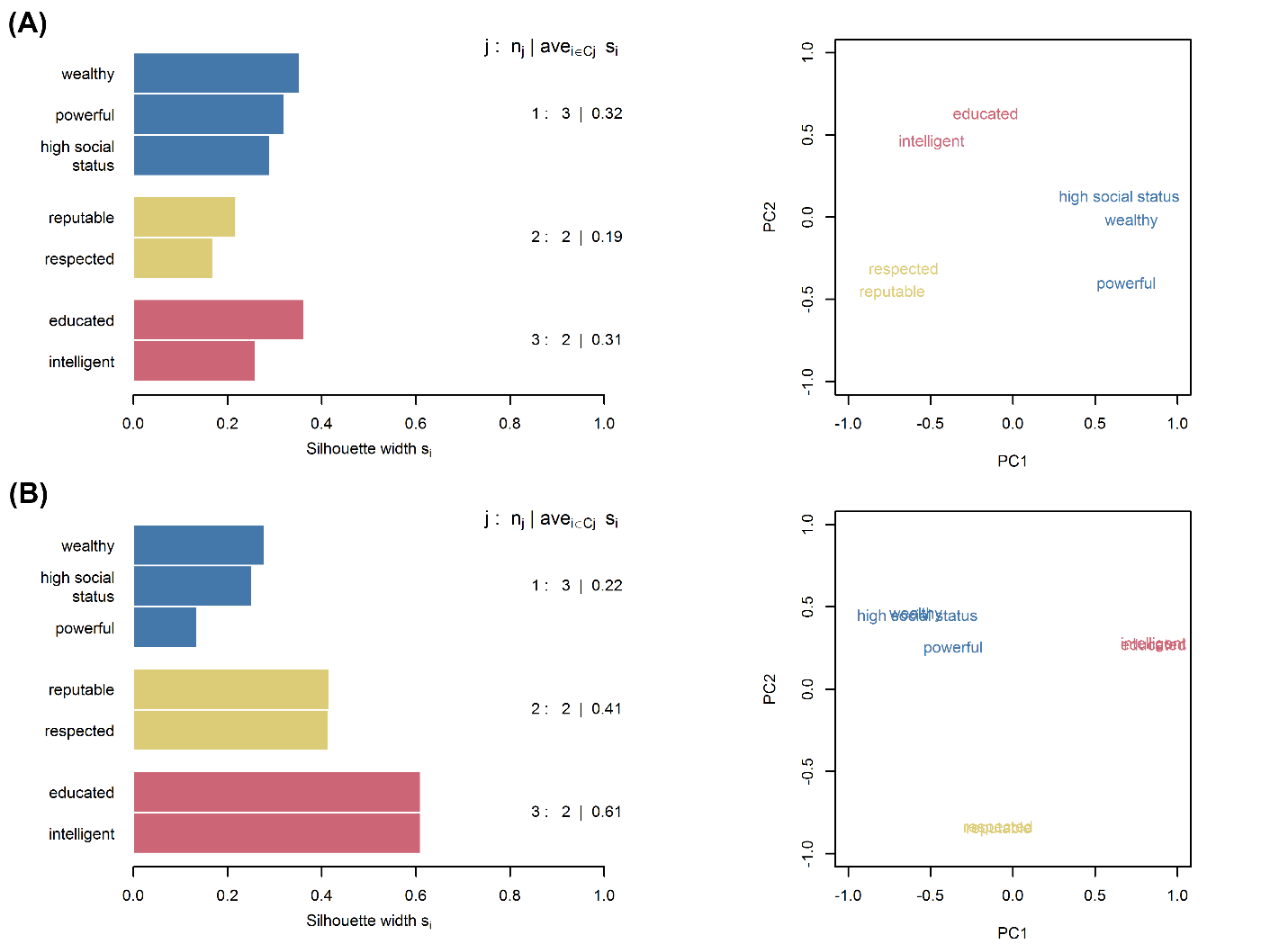


**Fig S1.3. Silhouette plots and multidimensional scaling plots of the clustering of prestige domain items.** Plots depict clustering of: **(A)** internal prestige domain attitudinal items, after eliminating items that failed to meet acceptance criteria; and **(B)** prestige domain triad items, after eliminating items that failed to meet acceptance criteria as well as *hardworking* and *ambitious* (see text). Position, reputation, and information items are shown in light blue, gold, and light red, respectively. To the right of each cluster is the number of items in that cluster and its average silhouette width. Silhouette width values represent the relative consistency of each item within its cluster. Multidimensional scaling was done using Manhattan distances for attitudinal items and tetrachoric correlations for triad items.

## Study 2: Scale evaluation

### Item generation

We used the full set of items generated for the previous scale construction study (**Table 1**) for evaluation and validation of the scale. We selected three additional prestige items (*talented*, *driven*, and *skilled*) from those generated by the free listing exercise to explore whether the inclusion of additional terms would have any effect on the PRI structure or provide additional explanatory power. As a number of the existing terms could be interpreted as measures of “ascribed” prestige (i.e. traits that are largely assigned or fixed based on the circumstances of one’s birth), we chose these terms as representative of the concept of “achieved” prestige (i.e. traits that can be earned or acquired) [44,45].

We also reverse-scored three items (*intelligent*-*unintelligent*, *ambitious*-*unambitious*, and *kind*-*unkind*) to reduce potential bias in responses [46], selected intentionally to avoid potentially ambiguous reversals. However, during exploratory analyses, we found that the distributions of responses to the reversed items were significantly skewed toward higher values than for the same items in the scale construction study. This suggests that participants were less likely to agree with a negative assessment of a speaker (i.e. *unintelligent*) than they were to disagree with its opposite positive assessment (*intelligent*). These differences caused issues with the consistency of responses and negatively affected model fit, similar to the problems seen later with reversed items in the Cheng et al. [47] scale (see Criterion validity) but to a lesser degree. Due to these issues, we do not recommend reversal for future studies using attitudinal items scored on a Likert-type scale (cf. [48]).

### Questionnaire construction and administration

In the online questionnaire for the scale evaluation study, we presented each participant with 10 audio recordings of the same passage used in the scale construction study: the first paragraph of *Comma Gets a Cure*. Each recording used a speaker with a different regional accent of English, and we asked participants to rate each speaker on all 23 attitudinal items (**Table 1**, plus *talented*, *driven*, and *skilled* under prestige) using a 7-point Likert-type scale from strongly disagree (1) to strongly agree (7).

We presented participants in the US with 8 US-based accents and 2 UK-based accents, while participants in the UK were presented with 8 UK-based accents and 2 US-based accents, for a total of 16 different accents across the entire sample, 4 of which were cross-tested in both countries (**Table S1.2**). The recordings for the 4 cross-tested accents were identical to those used in the scale construction study. All recordings were used under license from IDEA (see Acknowledgements) except for American West (Urban) and Wales, which we recruited from local contacts and recorded.

**Table S1.2. Regional accents and the participants to which they were presented in the scale evaluation study.**

| **United States** | **All Participants** | **United Kingdom** |
| --- | --- | --- |
| American West (Rural) | American West (Urban) | Southwest England |
| Midland | American Inland South (Blue-Collar) | Southeast England |
| Inland North | Received Pronunciation | Yorkshire |
| American Inland South (White-Collar) | Northwest England | Scotland |
| Mid-Atlantic |  | Ireland |
| New York City |  | Wales |

As in the scale construction study, we selected speakers for consistency from the recordings available. All speakers self-identified as white men and ranged in age from 31 to 59 years. Speakers varied in their level of education, occupation, and settlement size during childhood. The speaker from Wales was 45 years old at the time of recording, held an advanced degree, and was employed in an academic profession. The demographics of the American West (Urban) speaker are given in the methods for the scale construction study and all speaker demographics are available in **S5 Table**.

We collected data in June 2016 using online surveys implemented on SurveyMonkey and distributed using Amazon Mechanical Turk and TurkPrime [17] (*n* = 151 US) and Prolific (*n* = 144 UK), for a sample size of 295. We excluded participants from the prior scale construction study to ensure an independent sample. The results did not contain any missing data for attitudinal items.

### Demographic comparisons

Demographic characteristics of the scale evaluation sample were similar to the scale construction sample (**Fig S1.4**). Permutation tests of independence [49], adjusted for multiple comparisons to control for the false discovery rate, confirmed that significant differences were present only in the distributions of the age (*p* < 0.001) and occupation (*p* < 0.001) variables between the two studies, as a result of a larger proportion of relatively younger students in the scale evaluation sample. Given the similarity across all other variables, we considered this to be a relatively minor issue, and one that could be checked analytically by examining measurement invariance (see Confirmatory factor analysis).


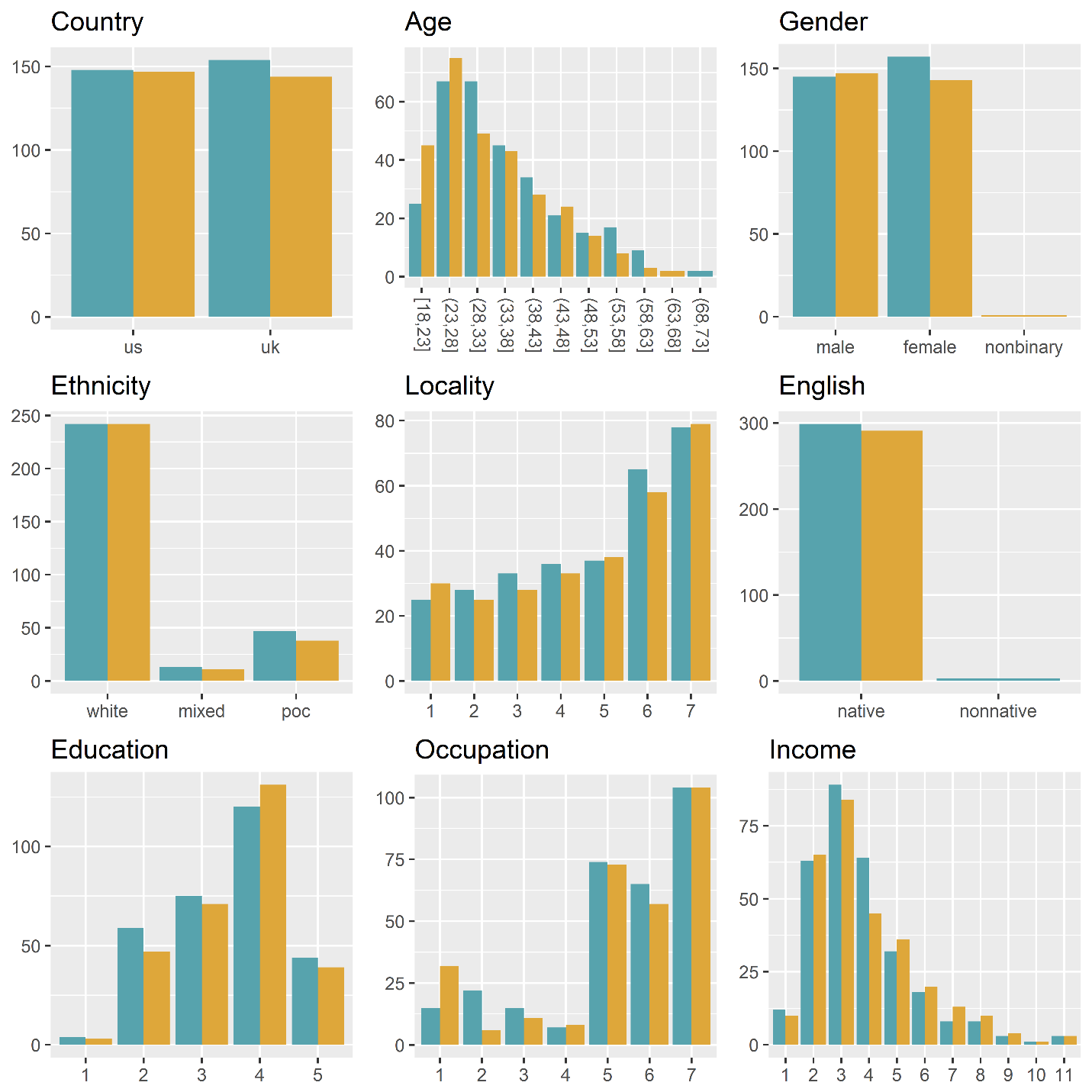


**Fig S1.4.** **Demographic comparisons of the samples from the scale construction and scale evaluation studies.** Vertical axes display counts of participants for each variable. Teal bars represent participants in the first (scale construction) study and orange bars represent participants in the second (scale evaluation) study. Numerical levels for categorical variables are defined in **S4 Metadata**.

### Exploratory factor analysis

Following checks of assumptions, outliers, item relationships, item factorability, and sample size, we conducted EFA on the scale evaluation data using methods and criteria identical to those used in the scale construction study, to address the question of whether the items generated adequately represented the breadth and structure of the individual prestige concept. The items that were previously eliminated in the EFA of the scale construction study were eliminated again in the process of conducting this EFA, due to violations of acceptance criteria. All three additional “achieved” prestige items—*talented*, *driven*, and *skilled—*were also eliminated, particularly because of high cross-loadings or primary loadings on other factors. We therefore made no changes to the structure of the model or the items included and found the scope of the existing PRI model to be adequate for use in CFA.

### Confirmatory factor analysis

The distribution of responses to the attitudinal items was not multivariate normal (*p* ≅ 0 for all tests). We removed four participants with six extreme outlier values (all from the US sample) as a result of examining Mahalanobis distances, leaving a final sample size of 291. The individual variables were approximately normal, and values of skewness (*M* = -0.209, *SD* = 0.369) and kurtosis (*M* = -0.617, *SD* = 0.279) were within acceptable ranges.

We then performed measurement invariance testing [50], to ensure that the relationships between indicators and latent variables within the prestige construct were consistent across participant demographic groups by country, age, gender, ethnicity, locality size, educational attainment, occupation, and income (see **S4 Metadata** for details on demographic variables). The sample contained an insufficient number of non-native English speakers to test for invariance by native English proficiency; therefore, we excluded this variable. We tested five increasingly constrained models in sequence: configural invariance (Model 1), metric or “weak” invariance (Model 2), scalar or “strong” invariance (Model 3), residual or “strict” invariance (Model 4), and residual invariance with constrained means (Model 5). We established configural invariance using the permutation method proposed by Jorgensen et al. [51]. We looked at changes in two noncentrality-based fit indices, the Comparative Fit Index (“CFI”) and the Root Mean Square Error of Approximation (“RMSEA”), to evaluate the relative fit of each successive nested model, with ΔCFI values less than or equal to 0.010 and ΔRMSEA values less than or equal to 0.015 indicating invariance [52]. Fulfilment of scalar invariance was considered sufficient to proceed with confirmatory factor analysis [50].

Measurement invariance of the model was upheld across the demographic variables of country, age, gender, occupation, and income. We found metric non-invariance by locality size (ΔCFI = 0.011, ΔRMSEA = 0.030), and ethnicity and educational attainment were borderline metric non-invariant (ΔCFI = 0.007, ΔRMSEA = 0.022; and ΔCFI = 0.009, ΔRMSEA = 0.026, respectively). Given these results, we defined a complex survey design which re-fit the model using pseudo-maximum likelihood and provided adjusted point and variance estimates [53,54]. In this design, the potentially non-invariant demographic variables were incorporated as weighted sampling strata using weights approximated from US [55–57] and UK census data [58–60].

We then performed confirmatory factor analysis (“CFA”) to assess the fit of this model to the scale evaluation data. As the WLSMV estimation method used in previous analyses could not be applied to a complex survey design, we used maximum likelihood with robust standard errors (mean and variance adjusted using the Satterthwaite approach [61], “MLMVS”) for the CFA based on the complex survey design. Equivalent results should be obtained from either method, as the two perform comparably for 7-point ordinal data [35].

We assessed the goodness of fit of confirmatory models with and without the complex survey design using two incremental fit indices (the CFI, as above, and the Tucker-Lewis Index, or “TLI”) and two absolute fit indices (the RMSEA, as above, and the Standardized Root Mean Square Residual, or “SRMR”). We drew cutoff criteria from Hu & Bentler [62] and adjusted them to the recommendations of Yu [63] as follows: CFI > 0.95; TLI > 0.96; RMSEA < 0.05; and SRMR < 0.07. We obtained parameter estimates using both robust maximum likelihood and robust weighted least squares methods, and compared fit indices for the models using MLMVS estimation, MLMVS estimation with a complex survey design, and WLSMV estimation (**Table S1.3**).

**Table S1.3. Goodness of fit indices for the Position-Reputation-Information scale model.** Robust indicators were estimated using: maximum likelihood methods, maximum likelihood methods with fit adjustments from a complex survey design to account for demographic non-invariance, and weighted least squares methods. Bounds for 90% confidence intervals are provided for RMSEA.

| **Model** | **CFI** | **TLI** | **RMSEA [90% CI]** | **SRMR** |
| --- | --- | --- | --- | --- |
| MLMVS | 0.948 | 0.983 | 0.056 [0.048, 0.064] | 0.020 |
| MLMVS with Complex Survey Design | 0.959 | 0.983 | 0.031 [0.026, 0.036] | 0.023 |
| WLSMV | 0.994 | 0.989 | 0.094 [0.085, 0.104] | 0.022 |

All three models shared the identical PRI structure and had comparable fit indices. We selected the model using MLMVS with adjustments from the complex survey design as the preferred model (**Fig 3**) because it fulfilled the cutoff criteria for all fit indices and properly incorporated information on all potentially non-invariant demographic variables.

## Scale validity and reliability

### Content validity

Content validity is the assessment of whether the scale adequately represents the extent of the domain of interest [64]. As content validity is essentially a qualitative judgment rather than a statistical one [65], we worked to establish and report the content validity of the PRI scale through the methods used to generate the items and those used to construct and verify the scale.

As mentioned previously, items were generated in part by participants through an inductive, endogenous process in the free listing task, which produced a broad but consistent sample of items. We supplemented this with more traditional deductive sampling of terms from previous literature and a pilot study. Rather than consulting external subject matter experts (cf. [66]), we valued more highly the validity of judgments by the study participants in generating and associating items.

Additionally, we included three “achieved” prestige items (*talented*, *driven*, and *skilled*), drawn from the free listed terms, to confirm the content validity of the model being tested. These items (and all of the same items dropped previously from the scale construction study) were dropped due to failure to meet the acceptance criteria, which lends support to the validity of the PRI scale and the sufficiency of its domain breadth.

Finally, we considered that the relative lack of demographic diversity among free listing participants in the initial scale construction study (compared to that of our other samples) could have negatively impacted the breadth of items generated and hence the content validity of the scale. However, we found no specific evidence to suggest this was the case, aside from potential issues of measurement non-invariance (see Confirmatory factor analysis) which could have occurred regardless. We therefore do not consider this to have been a point of concern for the present study but would recommend future studies endeavor to recruit a maximally diverse and representative sample from the population of interest for item generation.

### Construct validity

Construct validity is the property that the scale measures what it is intended to measure, which is generally confirmed by showing correlations among elements expected to be similar (“convergent validity”) and a lack of correlation among elements expected to be dissimilar (“discriminant validity”). The construct validity of the PRI scale was established by examining the convergent validity of scale items, the discriminant validity between PRI subscales (position, reputation, and information), and the discriminant validity between the prestige scale and the other two domains included in the data (solidarity and dynamism).

We assessed convergent validity within the scale by examining the polychoric correlation matrices of the scale items in both studies and the average variance explained (“AVE”) of the scale items in the scale evaluation study. Following common practice, correlation coefficients between 0.10 and 0.30 were considered small, between 0.30 and 0.50 were moderate, and greater than 0.50 were large [67]. AVE values greater than 0.50 were deemed acceptable, as they indicate sufficient variance attributed to the construct as opposed to measurement error [68].

We found polychoric correlations (ρ) between all PRI scale items to be high (*M* = 0.631, *SD* = 0.094) and correlations were higher between items within the same subscale than between items in different subscales (**Table S1.4**). The AVE values for each of the three subscales—position, reputation, and information—were 0.675, 0.630, and 0.699, respectively; all were above the criterion of 0.50, supporting convergent validity.

**Table S1.4.** **Polychoric correlations between Position-Reputation-Information scale items.** Mean (and standard deviation) polychoric correlations between Position-Reputation-Information items within the same subscale are shown on the main diagonal and those for items between different factors are shown below the main diagonal. Values were calculated using the combined scale construction and scale evaluation data sets. Correlations within 2-item factors have no mean or standard deviation because they consist of only one measurement.

|  | **Position** | **Reputation** | **Information** |
| --- | --- | --- | --- |
| **Position** | 0.733 (0.097) |  |  |
| **Reputation** | 0.580 (0.043) | 0.707 |  |
| **Information** | 0.625 (0.118) | 0.594 (0.054) | 0.733 |

The discriminant validity of constructs, which naturally opposes convergent validity, was assessed using the heterotrait-monotrait ratio of correlations criterion (“HTMT”), a method developed to avoid the potential issues of other indices [69]. For this criterion, lower values indicate greater discriminant validity. HTMT values between the prestige PRI subscales and the other two constructs—solidarity and dynamism—were all below the cutoff of 0.85 advised by Voorhees et al. [70], verifying discriminant validity of the prestige construct (**Table S1.5**). Similarly, HTMT values showed good discriminant validity between the three PRI subscales. This shows that the three PRI subscales, along with showing good convergent validity (as their items are all measuring elements of the same prestige construct), also exhibit substantial discriminant validity from other constructs and from one another. We consider these results to be support for the PRI scale’s overall construct validity and simple structure.

**Table S1.5.** **Heterotrait-monotrait ratio of correlations between items from Position-Reputation-Information subscales and solidarity and dynamism constructs.** HTMT values between each Position-Reputation-Information subscale and the solidarity and dynamism constructs are shown below the main diagonal. Lower HTMT values indicate greater discriminant validity. Values were calculated using the scale evaluation data set.

|  |  | **PRESTIGE** | | | **SOLIDARITY** |
| --- | --- | --- | --- | --- | --- |
|  |  | ***Position*** | ***Reputation*** | ***Information*** |  |
| **PRESTIGE** | ***Position*** |  |  |  |  |
|  | ***Reputation*** | 0.818 |  |  |  |
|  | ***Information*** | 0.841 | 0.835 |  |  |
| **SOLIDARITY** |  | 0.086 | 0.442 | 0.246 |  |
| **DYNAMISM** |  | 0.727 | 0.773 | 0.735 | 0.670 |

### Criterion validity

The criterion validity of a scale relates to its ability to be used as a measurement tool for the construct of interest, either assessed concurrently with a direct measure of that construct, in comparison with other available tests, or as a predictive indicator of independent or future outcomes. Predictive validity could not be assessed in this instance, as we did not have any future measurements or any independent prestige-related traits that were not already used in scale construction and evaluation, so we assessed the concurrent criterion validity of the scale through the other two avenues.

We first compared each item’s polychoric correlation with the *prestigious* item. The *prestigious* item was included in the surveys but excluded from the scale, and was used as a direct representative of the general construct of prestige that we intended to measure. In the scale evaluation data set, polychoric correlations between scale items and the *prestigious* item were high overall (*M* = 0.678, *SD* = 0.104), as were mean correlations with *prestigious* within each of the PRI factors (position: *M* = 0.748, *SD* = 0.096; reputation: *M* = 0.626, *SD* = 0.026; information: *M* = 0.627, *SD* = 0.143). Estimated factor scores for each PRI factor (using the Empirical Bayes Modal approach [71] for ordinal variables and the MLMVS model with adjustments from the complex survey design) were even more highly correlated with *prestigious* than the raw item scores (PRI individual prestige: ρ = 0.815; position: ρ = 0.844; reputation: ρ = 0.764; information: ρ = 0.745).

Secondly, to compare with another test of prestige, we asked a new set of participants (*n* = 91 US, 53 UK; again recruited through Amazon Mechanical Turk and Prolific) to rate two new speakers (having the Inland South and Received Pronunciation accents) using the present scale alongside the prestige-dominance scale of Cheng et al. (as detailed in the Electronic Supplementary Material of [47]). We modified the text of the items in the Cheng et al. scale (from “members of your/the group” to “people”) to better fit the context of our study. We removed outliers from the data and, using the same methods as above (with WLSMV estimation for the Cheng et al. scale data as the previous estimation method was not specified [47]), calculated factor scores for the PRI subscales, the solidarity and dynamism dimensions, and the prestige and dominance factors from the Cheng et al. prestige-dominance scale. We calculated polychoric correlations to examine the level of agreement between these measures.

In this additional comparative data set, we found substantial correlations between factor scores of the PRI scale and the prestige factor of the Cheng et al. scale (PRI overall: ρ = 0.850, position: ρ = 0.805, reputation: ρ = 0.861, information: ρ = 0.828) and, in general, we found that the individual prestige items of each scale were correlated (*M* = 0.567, *SD* = 0.221). However, one item in particular from the Cheng et al. scale (item 17: “Other people do NOT enjoy hanging out with him”) was relatively uncorrelated with PRI items and with the other Cheng et al. prestige items. Notably, this is one of the three reversed items in the Cheng et al. prestige factor, the other two of which (items 2 and 6: “People do NOT want to be like him” and “People do NOT value his opinion”) had only moderate correlations with PRI items and other Cheng et al. prestige items. The removal of all three reversed items had little effect on correlations between the Cheng et al. prestige factor and the PRI subscales (PRI overall: ρ = 0.856, position: ρ = 0.810, reputation: ρ = 0.867, information: ρ = 0.832) but improved the mean correlation between individual items (*M* = 0.690, *SD* = 0.066).

The reversed items contributed to the poor fit of the Cheng et al. scale overall in this data set (CFI = 0.875, TLI = 0.856, RMSEA = 0.229 [90% CI: 0.219, 0.238], SRMR = 0.154; using WLSMV estimation). The model fit improved with the removal of all reversed items (CFI = 0.973, TLI = 0.966, RMSEA = 0.151 [90% CI: 0.137, 0.165], SRMR = 0.083), but remained unacceptable under criteria for the two absolute fit indices, RMSEA and SRMR. We found the fit of the PRI scale using the same data and estimation method (WLSMV) met the cutoffs for all indices except RMSEA (CFI = 0.998, TLI = 0.995, RMSEA = 0.106 [90% CI: 0.075, 0.139], SRMR = 0.019). Notably, polychoric correlations between—first—the factor scores for dominance in the Cheng et al. scale (reversed items removed) and—second—the Cheng et al. prestige factor scores, the *prestigious* item, and the PRI factor scores, were all moderate to high (Cheng et al. prestige: ρ = 0.449, *prestigious*: ρ = 0.561, PRI prestige overall: ρ = 0.533, position: ρ = 0.569, reputation: ρ = 0.489, information: ρ = 0.501), which may indicate issues with the validity of the dominance construct.

### Interrater reliability

In these studies, we did not expect participants to rate each speaker identically for each item, nor is such agreement required to obtain a reliable scale of individual prestige. As mentioned in the Introduction, prior work has shown that different demographic groups will evaluate accents differently. By testing and adjusting the fit of the confirmatory model, we already incorporated information on patterns of variation in item ratings, both by individual and between demographic groups. Our results showed that participants displayed a consistent understanding of the overall prestige construct regardless of disagreements about particular speakers. This being said, measures of interrater reliability can be obtained and so we provide them here for completeness.

We calculated Krippendorff’s alpha coefficient [72] using ordinal weights, as well as the intraclass correlation coefficient (“ICC,” specifically ICC(C,1) of McGraw & Wong [73]). The level of Krippendorff’s alpha indicating agreement was 0.8, with values between 0.667 and 0.800 allowing for “tentative conclusions” [72]. For the ICC, values less than 0.40 were considered to be poor, between 0.40 and 0.60 were fair, between 0.60 and 0.75 were good, and greater than 0.75 were excellent [74]. The reliability values of Krippendorff’s alpha obtained for the scale construction and scale evaluation data sets were 0.414 and 0.383, respectively. ICC values for the two data sets were 0.473 [95% CI: 0.359, 0.625] and 0.459 [95% CI: 0.346, 0.612], using only the ratings of speakers that were cross-tested in both countries.

### Internal consistency

Lastly, the internal consistency of a scale measures the similarity of results across scale items. We examined this by calculating Cronbach’s alpha [75] as well as three variations of the omega coefficient (Raykov, Bentler, and McDonald, as described in [76]). The criterion used for acceptable values of internal consistency measures, given that this study is basic research for the purpose of developing a scale, was 0.80 [28,77].

Using the fitted MLMVS model with adjustments from the complex survey design, internal consistency measures were well above the cutoff for the overall scale, and above or slightly below it for the three PRI latent factors (**Table S1.6**). Analyses showed that these values would only decrease if we removed any individual scale item, suggesting that they are all vital to the structure of the scale.

**Table S1.6. Internal consistency measures for the Position-Reputation-Information scale and its subscales.**

|  | **Cronbach's alpha** | **Omega** |  |  |
| --- | --- | --- | --- | --- |
| **PRESTIGE** | 0.892 | 0.918 | | |
|  |  | **Raykov** | **Bentler** | **McDonald** |
| ***Position*** | 0.844 | 0.858 | 0.858 | 0.859 |
| ***Reputation*** | 0.772 | 0.773 | 0.773 | 0.773 |
| ***Information*** | 0.794 | 0.818 | 0.818 | 0.818 |

## Supplementary references

1. Bernard HR. Research methods in anthropology: Qualitative and quantitative approaches. 5th ed. Rowman Altamira; 2011.

2. Quinlan M. Considerations for collecting freelists in the field: Examples from ethobotany. Field Methods. 2005;17: 219–234. doi:10.1177/1525822X05277460

3. Weller SC. Structured interviewing and questionnaire construction. 2nd ed. In: Bernard HR, Gravlee CC, editors. Handbook of methods in cultural anthropology. 2nd ed. Rowman & Littlefield; 2015. pp. 343–390.

4. Smith JJ, Borgatti SP. Salience counts-and so does accuracy: Correcting and updating a measure for free-list-item salience. Journal of Linguistic Anthropology. 1997;7: 208–209. doi:10.1525/jlin.1997.7.2.208

5. Mulac A. Evaluation of the speech dialect attitudinal scale. Speech Monographs. 1975;42: 184–189. doi:10.1080/03637757509375893

6. Mulac A. Assessment and application of the revised speech dialect attitudinal scale. Communication Monographs. 1976;43: 238. doi:10.1080/03637757609375935

7. Zahn CJ, Hopper R. Measuring language attitudes: The Speech Evaluation Instrument. Journal of Language and Social Psychology. 1985;4: 113–123. doi:10.1177/0261927X8500400203

8. Gundersen DF, Perrill NK. Extending the “Speech Evaluation Instrument” to public speaking settings. Journal of Language and Social Psychology. 1989;8: 59–61. doi:10.1177/0261927X8900800105

9. Fuertes JN, Gottdiener WH, Martin H, Gilbert TC, Giles H. A meta-analysis of the effects of speakers’ accents on interpersonal evaluations: Effects of speakers’ accents. European Journal of Social Psychology. 2012;42: 120–133. doi:10.1002/ejsp.862

10. Giles H. Evaluative Reactions to Accents. Educational Review. 1970;22: 211–227. doi:10.1080/0013191700220301

11. Wells JC. Accents of English. Cambridge University Press; 1982.

12. Labov W, Ash S, Boberg C. The atlas of North American English: Phonetics, phonology and sound change. Walter de Gruyter; 2005.

13. Shackleton RG. Phonetic variation in the traditional English dialects: A computational analysis. Journal of English Linguistics. 2007;35: 30–102. doi:10.1177/0075424206297857

14. Bishop H, Coupland N, Garrett P. Conceptual accent evaluation: Thirty years of accent prejudice in the UK. Acta Linguistica Hafniensia. 2005;37: 131–154. doi:10.1080/03740463.2005.10416087

15. Milroy L. Britain and the United States: Two nations divided by the same language (and different language ideologies). Journal of Linguistic Anthropology. 2000;10: 56–89. doi:10.1525/jlin.2000.10.1.56

16. Burton ML, Nerlove SB. Balanced designs for triads tests: Two examples from English. Social Science Research. 1976;5: 247–267. doi:10.1016/0049-089X(76)90002-8

17. Litman L, Robinson J, Abberbock T. TurkPrime.com: A versatile crowdsourcing data acquisition platform for the behavioral sciences. Behavior Research Methods. 2017;49: 433–442. doi:10.3758/s13428-016-0727-z

18. Lubke GH, Muthén BO. Applying multigroup confirmatory factor models for continuous outcomes to Likert scale data complicates meaningful group comparisons. Structural Equation Modeling. 2004;11: 514–534. doi:10.1207/s15328007sem1104_2

19. Mardia KV. Measures of multivariate skewness and kurtosis with applications. Biometrika. 1970;57: 519–530. doi:10.1093/biomet/57.3.519

20. Mardia KV. Applications of some measures of multivariate skewness and kurtosis in testing normality and robustness studies. Sankhyā: The Indian Journal of Statistics, Series B. 1974;36: 115–128.

21. Henze N, Zirkler B. A class of invariant consistent tests for multivariate normality. Communications in Statistics - Theory and Methods. 1990;19: 3595–3617. doi:10.1080/03610929008830400

22. Royston JP. An extension of Shapiro and Wilk’s W test for normality to large samples. Applied Statistics. 1982;31: 115–124. doi:10.2307/2347973

23. Royston JP. Some techniques for assessing multivarate normality based on the Shapiro-Wilk W. Applied Statistics. 1983;32: 121–133. doi:10.2307/2347291

24. Bulmer MG. Principles of statistics. Courier Corporation; 1979.

25. West SG, Finch JF, Curran PJ. Structural equation models with nonnormal variables: Problems and remedies. In: Hoyle RH, editor. Structural equation modeling: Concepts, issues, and applications. Thousand Oaks, CA: Sage Publications; 1995. pp. 56–75.

26. Curran PJ, West SG, Finch JF. The robustness of test statistics to nonnormality and specification error in confirmatory factor analysis. Psychological Methods. 1996;1: 16–29. doi:10.1037/1082-989X.1.1.16

27. Kaiser HF, Rice J. Little jiffy, mark IV. Educational and Psychological Measurement. 1974;34: 111–117. doi:10.1177/001316447403400115

28. Nunnally JC. Psychometric theory. 2nd ed. New York: McGraw-Hill; 1978.

29. Tabachnick BG, Fidell LS, Osterlind SJ. Using multivariate statistics. Allyn & Bacon/Pearson Education; 2001.

30. Hair JF, Black WC, Babin BJ, Anderson RE. Multivariate data analysis. 10th ed. Upper Saddle River, NJ: Prentice Hall; 2010.

31. Bartlett MS. Properties of sufficiency and statistical tests. Proceedings of the Royal Society A. 1937;160: 268–282. doi:10.1098/rspa.1937.0109

32. Holgado-Tello FP, Chacón-Moscoso S, Barbero-García I, Vila-Abad E. Polychoric versus Pearson correlations in exploratory and confirmatory factor analysis of ordinal variables. Quality & Quantity. 2010;44: 153–166. doi:10.1007/s11135-008-9190-y

33. Savalei V. What to do about zero frequency cells when estimating polychoric correlations. Structural Equation Modeling. 2011;18: 253–273. doi:10.1080/10705511.2011.557339

34. Jöreskog KG, Sörbom D. PRELIS 2 user’s reference guide: A program for multivariate data screening and data summarization: A preprocessor for LISREL. Scientific Software International; 1996.

35. Rhemtulla M, Brosseau-Liard PÉ, Savalei V. When can categorical variables be treated as continuous? A comparison of robust continuous and categorical SEM estimation methods under suboptimal conditions. Psychological Methods. 2012;17: 354. doi:10.1037/a0029315

36. DiStefano C, Morgan GB. A comparison of diagonal weighted least squares robust estimation techniques for ordinal data. Structural Equation Modeling. 2014;21: 425–438. doi:10.1080/10705511.2014.915373

37. Kline P. Intelligence: The psychometric view. Florence, KY: Taylor & Frances/Routledge; 1991.

38. Costello AB, Osborne JW. Best practices in exploratory factor analysis: Four recommendations for getting the most from your analysis. Practical Assessment, Research & Evaluation. 2005;10: 1–9.

39. Ruscio J, Roche B. Determining the number of factors to retain in an exploratory factor analysis using comparison data of known factorial structure. Psychological Assessment. 2012;24: 282. doi:10.1037/a0025697

40. Hershberger SL. Tetrachoric correlation. Encyclopedia of Statistics in Behavioral Science. John Wiley & Sons, Ltd; 2005. doi:10.1002/0470013192.bsa676

41. Kaufman L, Rousseeuw PJ. Partitioning around medoids (Program PAM). Finding groups in data: An introduction to cluster analysis. New York: John Wiley & Sons; 1990. pp. 68–125. doi:10.1002/9780470316801.ch2

42. Duda RO, Hart PE, Stork DG. Pattern classification and scene analysis. New York: Wiley; 1973.

43. Dunn JC. Well-separated clusters and optimal fuzzy partitions. Journal of Cybernetics. 1974;4: 95–104. doi:10.1080/01969727408546059

44. Svalastoga K. Prestige, class, and mobility. Scandinavian University Books; 1959.

45. Linton R. The study of man. New York: Appleton-Century-Crofts; 1936.

46. Schriesheim CA, Hill KD. Controlling acquiescence response bias by item reversals: The effect on questionnaire validity. Educational and Psychological Measurement. 1981;41: 1101–1114. doi:10.1177/001316448104100420

47. Cheng JT, Tracy JL, Henrich J. Pride, personality, and the evolutionary foundations of human social status. Evolution and Human Behavior. 2010;31: 334–347. doi:10.1016/j.evolhumbehav.2010.02.004

48. Harrison DA, McLaughlin ME. Cognitive processes in self-report responses: Tests of item context effects in work attitude measures. Journal of Applied Psychology. 1993;78: 129–140. doi:10.1037/0021-9010.78.1.129

49. Hothorn T, Hornik K, Van De Wiel MA, Zeileis A. A Lego system for conditional inference. The American Statistician. 2006;60: 257–263. doi:10.1198/000313006X118430

50. Putnick DL, Bornstein MH. Measurement invariance conventions and reporting: The state of the art and future directions for psychological research. Developmental Review. 2016;41: 71–90. doi:10.1016/j.dr.2016.06.004

51. Jorgensen TD, Kite BA, Chen P-Y, Short SD. Finally! A valid test of configural invariance using permutation in multigroup CFA. The Annual Meeting of the Psychometric Society. Springer; 2016. pp. 93–103. doi:10.1007/978-3-319-56294-0_9

52. Chen FF. Sensitivity of goodness of fit indexes to lack of measurement invariance. Structural Equation Modeling. 2007;14: 464–504. doi:10.1080/10705510701301834

53. Lumley T. Complex surveys: A guide to analysis using R. John Wiley & Sons; 2011.

54. Oberski DL. lavaan.survey: An R package for complex survey analysis of structural equation models. Journal of Statistical Software. 2014;57: 1–27. doi:10.18637/jss.v057.i01

55. United States Census Bureau, American FactFinder. B02001: Race. In: 2012-2016 American Community Survey [Internet]. 2016 [cited 7 Dec 2017]. Available: https://factfinder.census.gov/

56. United States Census Bureau, American FactFinder. P2: Urban and rural. In: 2010 US Census [Internet]. 2010 [cited 20 Sep 2017]. Available: https://factfinder.census.gov/

57. United States Census Bureau, American FactFinder. S1501: Educational attainment. In: 2012-2016 American Community Survey [Internet]. 2016 [cited 7 Dec 2017]. Available: https://factfinder.census.gov/

58. Office for National Statistics, Nomis. KS201UK: Ethnic group. In: 2011 UK Census [Internet]. 2011 [cited 20 Sep 2017]. Available: https://www.nomisweb.co.uk/

59. Department for Environment, Food & Rural Affairs. Rural population 2014/15. In: UK Rural Population and Migration Statistics [Internet]. 2012 [cited 20 Sep 2017]. Available: https://www.gov.uk/government/publications/rural-population-and-migration/rural-population-201415

60. Office for National Statistics, Nomis. QS501UK: Highest level of qualification. In: 2011 UK Census [Internet]. 2011 [cited 7 Dec 2017]. Available: https://www.nomisweb.co.uk/

61. Satorra A, Bentler PM. Corrections to test statistics and standard errors in covariance structure analysis. In: von Eye A, Clogg CC, editors. Latent variables analysis: Applications to developmental research. Thousand Oaks, CA: SAGE Publications, Inc.; 1994. pp. 399–419.

62. Hu L, Bentler PM. Cutoff criteria for fit indexes in covariance structure analysis: Conventional criteria versus new alternatives. Structural Equation Modeling. 1999;6: 1–55. doi:10.1080/10705519909540118

63. Yu C-Y. Evaluating cutoff criteria of model fit indices for latent variable models with binary and continuous outcomes. University of California, Los Angeles. 2002.

64. Cronbach LJ, Meehl PE. Construct validity in psychological tests. Psychological Bulletin. 1955;52: 281. doi:10.1037/h0040957

65. Haynes SN, Richard D, Kubany ES. Content validity in psychological assessment: A functional approach to concepts and methods. Psychological Assessment. 1995;7: 238. doi:10.1037/1040-3590.7.3.238

66. Lawshe CH. A quantitative approach to content validity. Personnel Psychology. 1975;28: 563–575. doi:10.1111/j.1744-6570.1975.tb01393.x

67. Cohen J. Statistical power analysis for the behavioral sciences. 2nd ed. New Jersey: Lawrence Erlbaum Associates; 1988.

68. Fornell C, Larcker DF. Evaluating structural equation models with unobservable variables and measurement error. Journal of Marketing Research. 1981;18: 39–50. doi:10.2307/3151312

69. Henseler J, Ringle CM, Sarstedt M. A new criterion for assessing discriminant validity in variance-based structural equation modeling. Journal of the Academy of Marketing Science. 2015;43: 115–135. doi:10.1007/s11747-014-0403-8

70. Voorhees CM, Brady MK, Calantone R, Ramirez E. Discriminant validity testing in marketing: An analysis, causes for concern, and proposed remedies. Journal of the Academy of Marketing Science. 2016;44: 119–134. doi:10.1007/s11747-015-0455-4

71. Skrondal A, Rabe-Hesketh S. Prediction in multilevel generalized linear models. Journal of the Royal Statistical Society: Series A. 2009;172: 659–687. doi:10.1111/j.1467-985X.2009.00587.x

72. Krippendorff K. Content analysis: An introduction to its methodology. 3rd ed. Thousand Oaks, CA: Sage; 2012.

73. McGraw KO, Wong SP. Forming inferences about some intraclass correlation coefficients. Psychological Methods. 1996;1: 30–46. doi:10.1037/1082-989X.1.1.30

74. Fleiss JL, Fleiss JL. Reliability of measurement. Design and analysis of clinical experiments. John Wiley & Sons; 1986. pp. 1–32.

75. Cronbach LJ. Coefficient alpha and the internal structure of tests. Psychometrika. 1951;16: 297–334. doi:10.1007/BF02310555

76. Revelle W, Zinbarg RE. Coefficients alpha, beta, omega, and the glb: Comments on Sijtsma. Psychometrika. 2009;74: 145–154. doi:10.1007/s11336-008-9102-z

77. Lance CE, Butts MM, Michels LC. The sources of four commonly reported cutoff criteria: What did they really say? Organizational Research Methods. 2006;9: 202–220. doi:10.1177/1094428105284919
