# Supplementary material for: The Position-Reputation-Information (PRI) scale of individual prestige
Source: PLoS One. 2020 Jun 25;15(6):e0234428. doi: 10.1371/journal.pone.0234428 (PMC7316272; doi:10.1371/journal.pone.0234428)
Supplement: S1 Form — List of final Position-Reputation-Information items with 7-point Likert-type rating scale, in Word DOCX format. Additional items of interest can be included and the order of all items should be randomized prior to presentation. We do not recommend reverse-scoring any items, due to observed biases in responses (see text). (DOCX) [file pone.0234428.s002.docx]

**The Position-Reputation-Information (PRI) scale of individual prestige**

Richard E.W. Berl^1*^, Alarna N. Samarasinghe^2^, Fiona M. Jordan^2,3^, Michael C. Gavin^1,3^

^1^ Department of Human Dimensions of Natural Resources, Colorado State University, Fort Collins, Colorado, United States of America

^2^ Department of Anthropology and Archaeology, University of Bristol, Bristol, United Kingdom

^3^ Max Planck Institute for the Science of Human History, Jena, Germany

* Corresponding author

E-mail: rewberl@colostate.edu (REWB)

# S2 Form: PRI scale administration form

**Instructions:** Please indicate your impressions of the speaker. Please try to use the entire breadth of the scale. Do not mark '4 (Neither Agree nor Disagree)' for every item.

The speaker sounds…

|  | **1** | **2** | **3** | **4** | **5** | **6** | **7** |
| --- | --- | --- | --- | --- | --- | --- | --- |
|  | Strongly Disagree | Disagree | Somewhat Disagree | Neither Agree nor Disagree | Somewhat Agree | Agree | Strongly Agree |
| wealthy |  |  |  |  |  |  |  |
| powerful |  |  |  |  |  |  |  |
| high social status |  |  |  |  |  |  |  |
| reputable |  |  |  |  |  |  |  |
| respected |  |  |  |  |  |  |  |
| educated |  |  |  |  |  |  |  |
| intelligent |  |  |  |  |  |  |  |
