# Supplementary material for: The Position-Reputation-Information (PRI) scale of individual prestige
Source: PLoS One. 2020 Jun 25;15(6):e0234428. doi: 10.1371/journal.pone.0234428 (PMC7316272; doi:10.1371/journal.pone.0234428)
Supplement: S1 Data — Archive of data sets (as RDS and CSV) and R code (pri_analyses, as R Markdown script and PDF document) used for all analyses and the generation of figures, in ZIP format. Data sets included are: free list data (list_f and data_f), pilot study attitudinal data (data_p), scale construction study attitudinal data (data_s) and triad data (data_t), scale evaluation study attitudinal data (data_c), and criterion validity comparative attitudinal data (data_v). The final confirmatory lavaan model object (cfa_pri.RDS) is also included. See S1 Metadata for full descriptions of data sets, types, and variables. (ZIP) [file pone.0234428.s003.zip › pri_analyses.pdf]

The Position-Reputation-Information (PRI) scale of individual prestige

Supplementary Material Data S2: Analyses

Richard E.W. Berl [1*], **Alarna N. Samarasinghe** [2], **Fiona M. Jordan** [2,3], **Michael C. Gavin** [1,3]

[1] Department of Human Dimensions of Natural Resources, Colorado State University, Fort Collins, Colorado, United States of America

[2] Department of Anthropology and Archaeology, University of Bristol, Bristol, United Kingdom

[3] Max Planck Institute for the Science of Human History, Jena, Germany

[*] Corresponding author
E-mail: rewberl@colostate.edu

Requirements

1. The R statistical computing environment and (optionally, but highly recommended) RStudio
2. R packages: *Rmisc*, *psych*, *e1071*, *MVN*, *lavaan*, *semTools*, *survey*, *lavaan.survey*, *semPlot*, *tidyr*, *dplyr*, *coin*, *rcompanion*, *ggplot2*, *corrplot*, *scatterplot3d*, *cluster*, *fpc*, *irr* (and dependencies)

```
#   Run the following lines to install needed packages
install.packages(c("Rmisc", "psych", "e1071", "MVN", "lavaan", "semTools",
                   "survey", "lavaan.survey", "semPlot", "tidyr", "dplyr",
                   "coin", "rcompanion", "ggplot2", "corrplot",
                   "scatterplot3d", "cluster", "fpc", "irr"))
```

3. Data sets from the S2 Data ZIP in RDS format, extracted to the same directory as this file
4. The *EFA Comparison Data.R* script that implements the parallel analysis with comparison data method from Ruscio and Roche (2012), available from the ZIP file on John Ruscio's website
5. To re-knit this document, you will also need:
   - Additional libraries including *knitr* and *rmarkdown*
   - An up-to-date installation of LaTeX (e.g. MiKTeX or MacTeX)

However, code chunks can be run sequentially from the *.Rmd* file without re-knitting.

The code in this file is commented to identify the analyses being done, but we do not provide any interpretation of the results here. See the Methods section of the paper for details.

CONTENTS

- STUDY 1: SCALE CONSTRUCTION
  - Item Generation
    * Pilot Study
    * Free Listing
  - Exploratory Data Analysis
  - Exploratory Factor Analysis
    * Attitudinal Data
    * Triad Data
  - Cluster Analysis
    * Attitudinal Data
    * Triad Data
- STUDY 2: SCALE EVALUATION
  - Exploratory Data Analysis
  - Exploratory Factor Analysis
    * Checking Assumptions
    * Exploratory Factor Analysis
  - Confirmatory Factor Analysis
    * Measurement Invariance
    * Confirmatory Factor Analysis
- SCALE VALIDITY AND RELIABILITY
  - Content Validity
  - Construct Validity
    * Convergent Validity
    * Discriminant Validity
  - Criterion Validity
    * Comparative Study
  - Interrater Reliability
  - Internal Consistency
- R Session Information
- References

STUDY 1: SCALE CONSTRUCTION

Item Generation

Pilot Study

Checking Assumptions

```
#   Check for multivariate normality
mardiaTest(data=data.p[,2:16], qqplot=T)
```

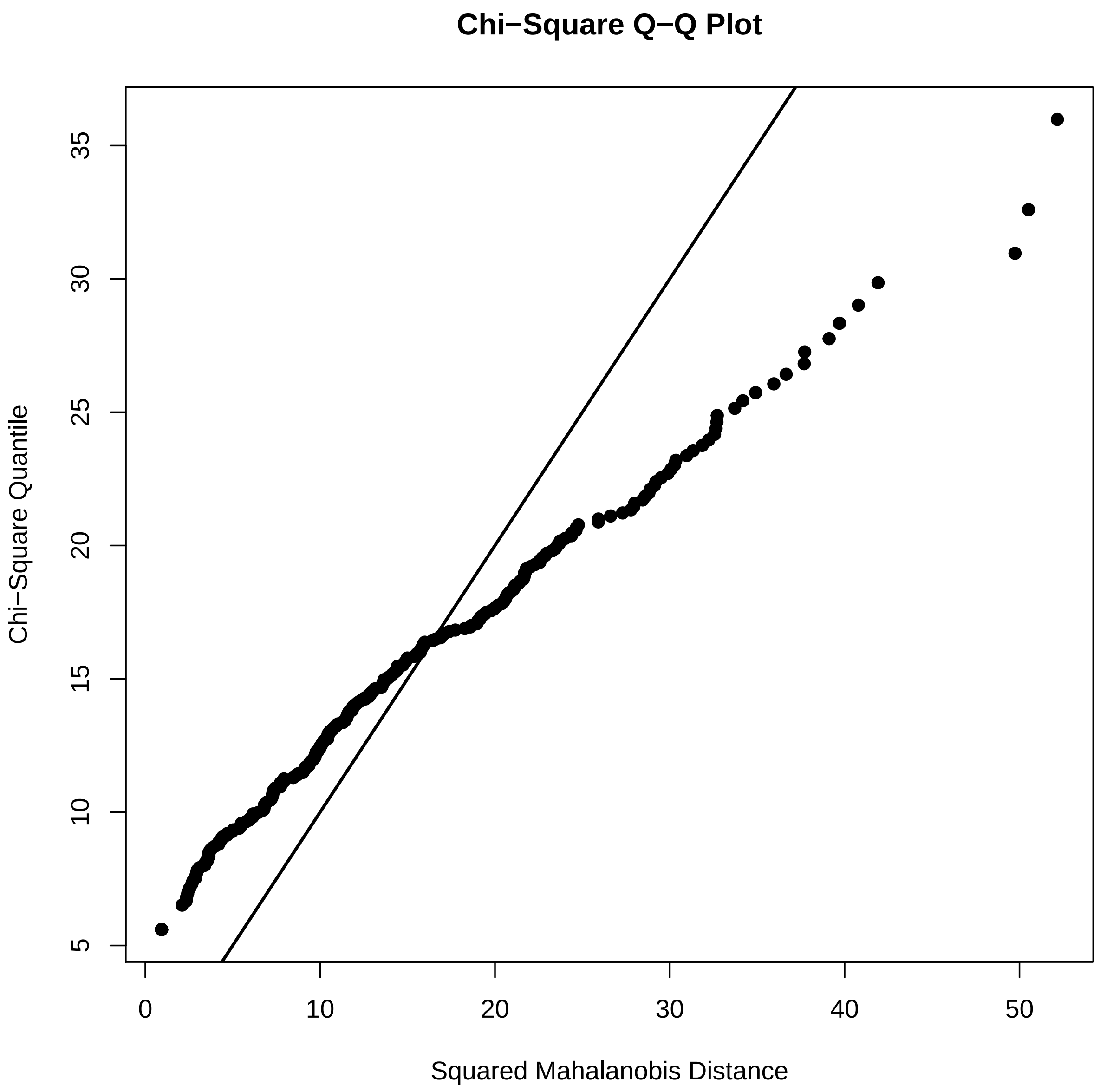

```
##    Mardia's Multivariate Normality Test
## ---------------------------------------
##    data : data.p[, 2:16]
##
##    g1p            : 31.51312
##    chi.skew       : 1475.864
##    p.value.skew   : 7.374685e-61
##
##    g2p            : 321.4878
##    z.kurtosis     : 24.67629
##    p.value.kurt   : 0
##
##    chi.small.skew : 1493.607
##    p.value.small  : 5.888681e-63
##
##    Result          : Data are not multivariate normal.
## ---------------------------------------
```

```
hzTest(data.p[,2:16], qqplot=F)
```

```
##   Henze-Zirkler's Multivariate Normality Test
## ---------------------------------------------
##   data : data.p[, 2:16]
##
##   HZ      : 1.878282
##   p-value : 0
##
##   Result  : Data are not multivariate normal.
## ---------------------------------------------
```

```
roystonTest(data.p[,2:16], qqplot=F)
```

```
##   Royston's Multivariate Normality Test
## ---------------------------------------------
##   data : data.p[, 2:16]
##
##   H       : 765.5607
##   p-value : 2.943977e-153
##
##   Result  : Data are not multivariate normal.
## ---------------------------------------------
```

```
#   Check for multivariate outliers
head(mvOutlier(data.p[,2:16], alpha=0.7, qqplot=T)$outlier, 10)
```

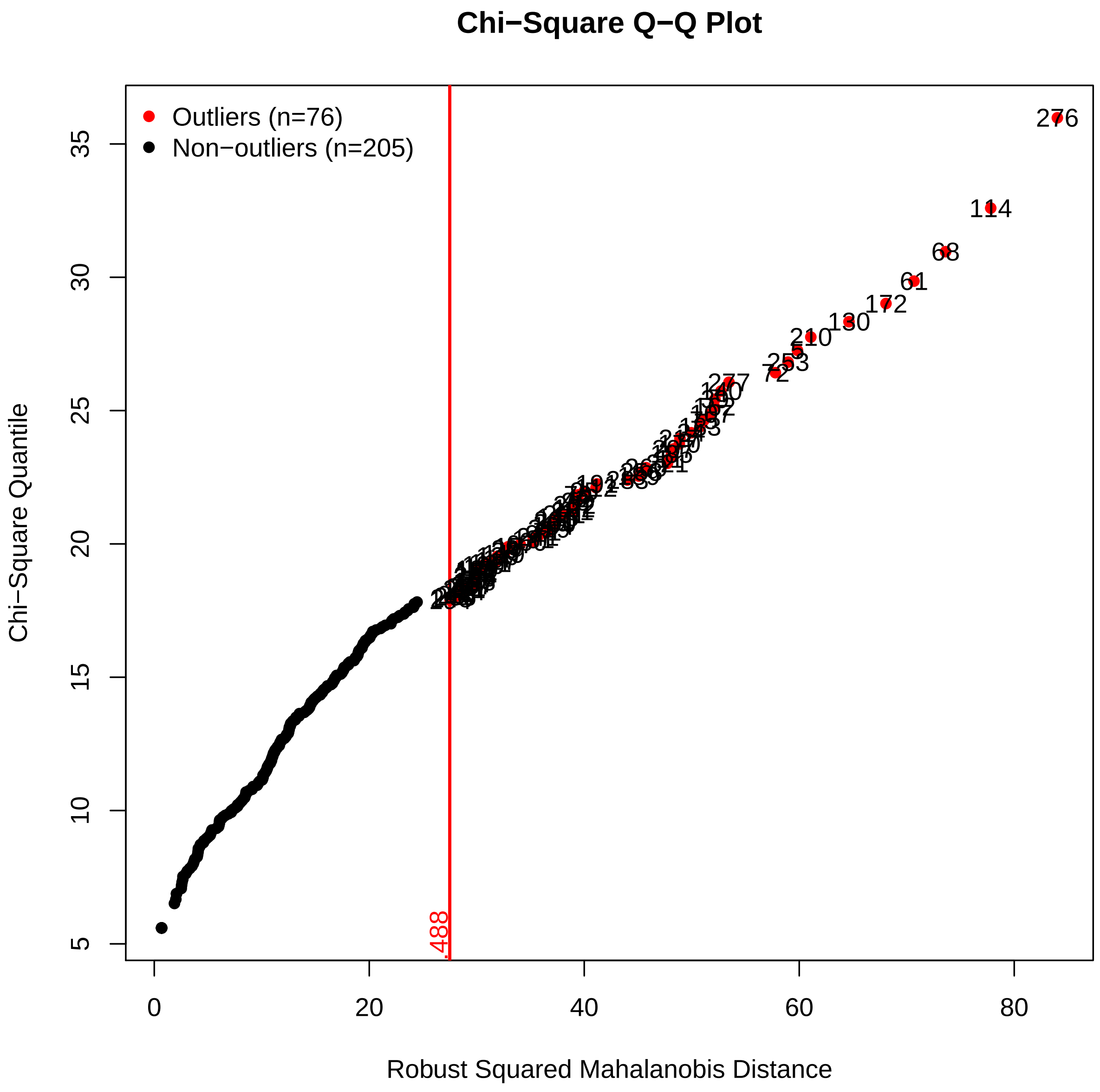


```
##     Observation Mahalanobis Distance Outlier
## 276         276               84.010    TRUE
## 114         114               77.812    TRUE
## 68           68               73.613    TRUE
## 61           61               70.675    TRUE
## 172         172               68.068    TRUE
## 130         130               64.624    TRUE
## 210         210               61.076    TRUE
## 5             5               59.834    TRUE
## 253         253               58.959    TRUE
## 72           72               57.781    TRUE
```

```
#       Loop to find rows of outlier observations for removal (warning: LONG,
```

```
#           uncomment to run)
# to.remove = as.numeric(as.character(mvOutlier(data.p[,2:16], alpha=0.7,
#                                                qqplot=F,
#                                                method="adj.quan")$outlier[1:3,1]))
# lines.to.remove = c()
# lines.progress = txtProgressBar(min=0, max=nrow(data.p), style=3, width=100)
# for (i in 1:nrow(data.p)) {
# x = as.numeric(as.character(mvOutlier(data.p[-i,2:16], alpha=0.7,
#                                        qqplot=F,
#                                        method="adj.quan")$outlier[1:8,1]))
#     if (!all(to.remove %in% x)) {
#         lines.to.remove = append(lines.to.remove, i)
#     }
#     setTxtProgressBar(lines.progress, i)
# }
# close(lines.progress)
# lines.to.remove

#       Result: 64 108 265

#       Check with results removed
head(mvOutlier(data.p[-c(64,108,265),2:16], alpha=0.7, qqplot=T,
               method="adj.quan")$outlier, 10)
```

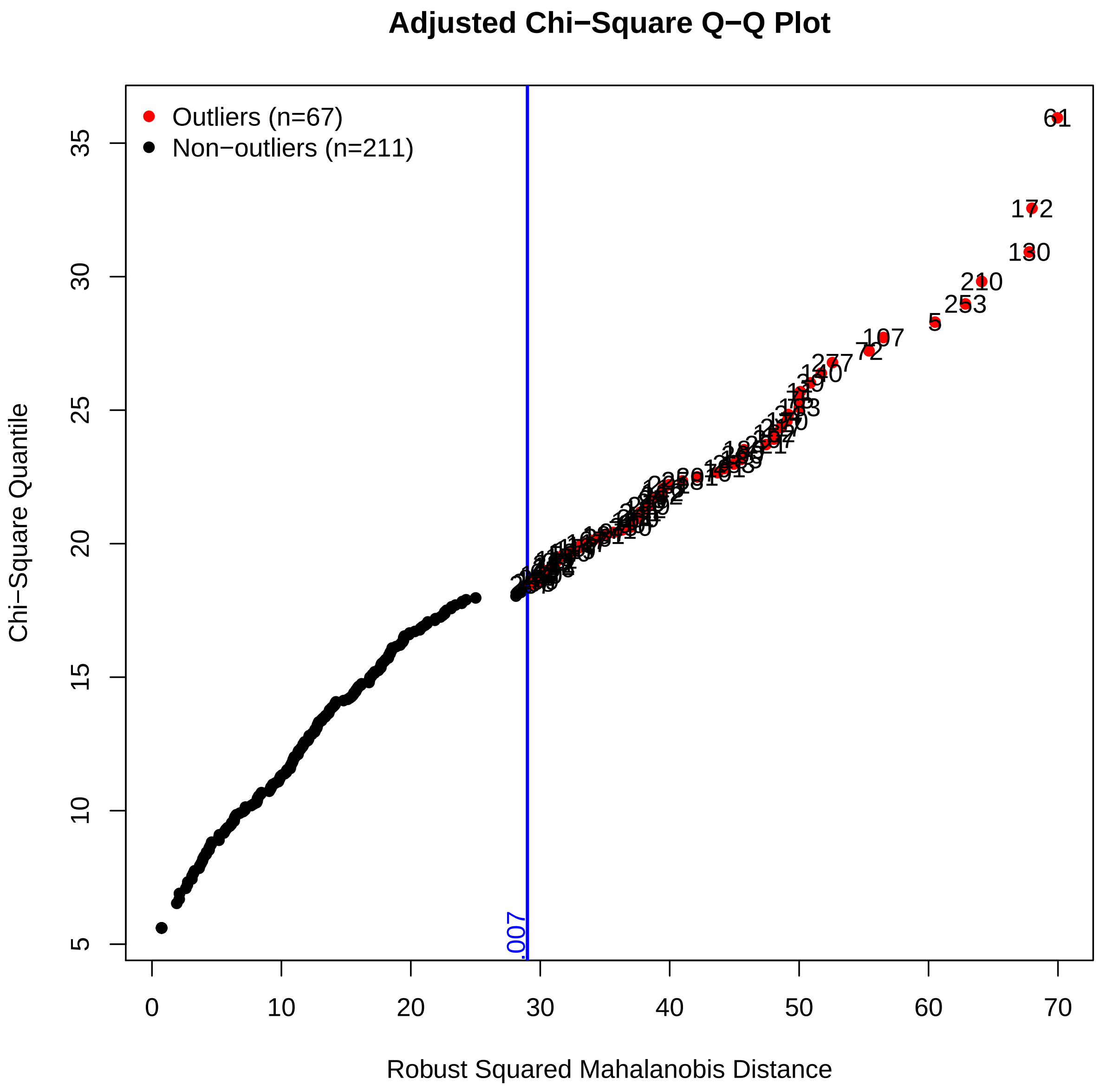


```
##     Observation Mahalanobis Distance Outlier
## 61           61               69.951    TRUE
## 172         172               67.989    TRUE
## 130         130               67.779    TRUE
## 210         210               64.104    TRUE
## 253         253               62.849    TRUE
## 5             5               60.499    TRUE
## 107         107               56.512    TRUE
## 72           72               55.398    TRUE
## 277         277               52.576    TRUE
## 140         140               51.712    TRUE
```

```
#       Identify participants
```

```
data.p[c(64,108,265),]
```

```
##          id friendly reliable ambitious sincere intelligent sociable
## 68     4.15        4        4         7       7           6        7
## 114 JH1.112        2        3         4       5           2        1
## 276 DM2.128        5        6         2       7           3        3
##     hardworking easygoing prestigious reputable confident comforting
## 68            4         5           7         7         7          7
## 114           4         5           2         5         1          7
## 276           5         2           7         5         4          5
##     pleasant interesting attractive country      accent       first   info
## 68         6           7          7      us    standard    standard gossip
## 114        3           4          2      uk nonstandard nonstandard social
## 276        6           3          3      uk nonstandard    standard social
##     age   age.o gender english
## 68   19 [18,23] female  native
## 114  21 [18,23]   male  native
## 276  20 [18,23] female  native
```

```
data.p.to.remove = as.character(data.p$id[c(64,108,265)])

#       Remove participants
data.p = data.p[!as.character(data.p$id) %in% data.p.to.remove,]
data.p.o = data.p.o[!as.character(data.p.o$id) %in% data.p.to.remove,]

#   Check for univariate normality
uniPlot(data.p[,2:16], "histogram")

#   Check skewness
sort(apply(data.p[,2:16], 2, e1071::skewness), decreasing=T)
```

```
##   confident    sociable interesting prestigious     sincere    pleasant
##  0.14615867  0.11095525  0.08127794  0.07182589  0.07130918  0.01327838
##   ambitious    friendly  comforting   easygoing  attractive intelligent
## -0.01067866 -0.05524376 -0.08458592 -0.25885992 -0.27718586 -0.36577180
##    reliable   reputable hardworking
## -0.41129870 -0.56588608 -0.74118601
```

```
#   Check kurtosis
sort(apply(data.p[,2:16], 2, e1071::kurtosis, type=2), decreasing=T)
```

```
## hardworking prestigious   ambitious   reputable intelligent  attractive
##  2.89500108  2.47034579  2.04031269  1.87415154  1.27311694  0.90267509
##    sociable interesting  comforting    friendly   easygoing    reliable
##  0.24420847  0.07131967 -0.04569917 -0.05649084 -0.09868718 -0.23499546
##     sincere    pleasant   confident
## -0.25363337 -0.45449093 -0.46965648
```

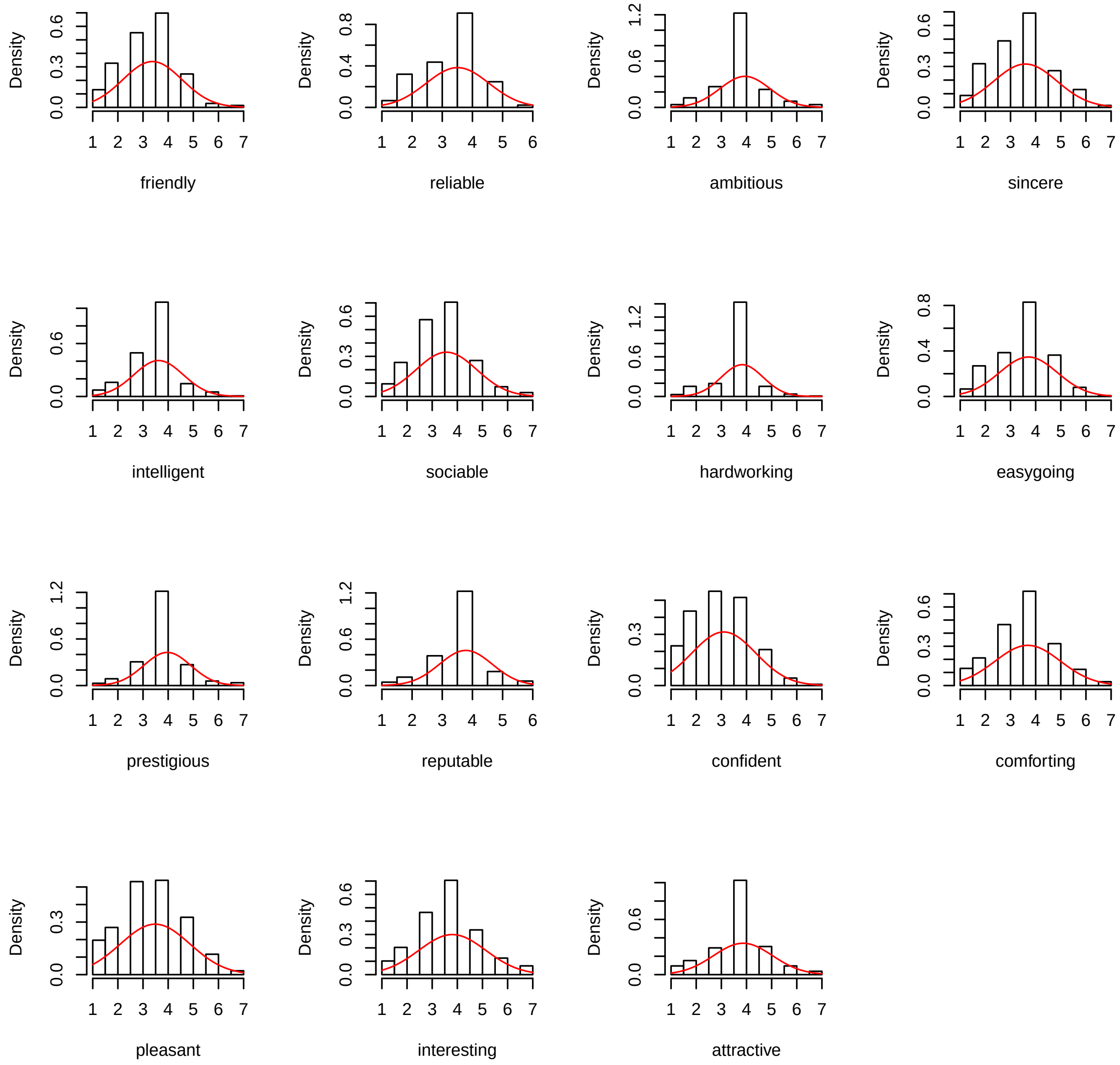


```
#   Check for linear relationships and correlations
#       Using non-ordinal dataset; lavCor fails due to empty ordinal levels
corrplot.mixed(lavCor(data.p[,2:16]), lower="ellipse", upper="number",
               order="hclust", hclust.method="ward.D",
               tl.cex=0.7, tl.pos="d", tl.col="black")
```

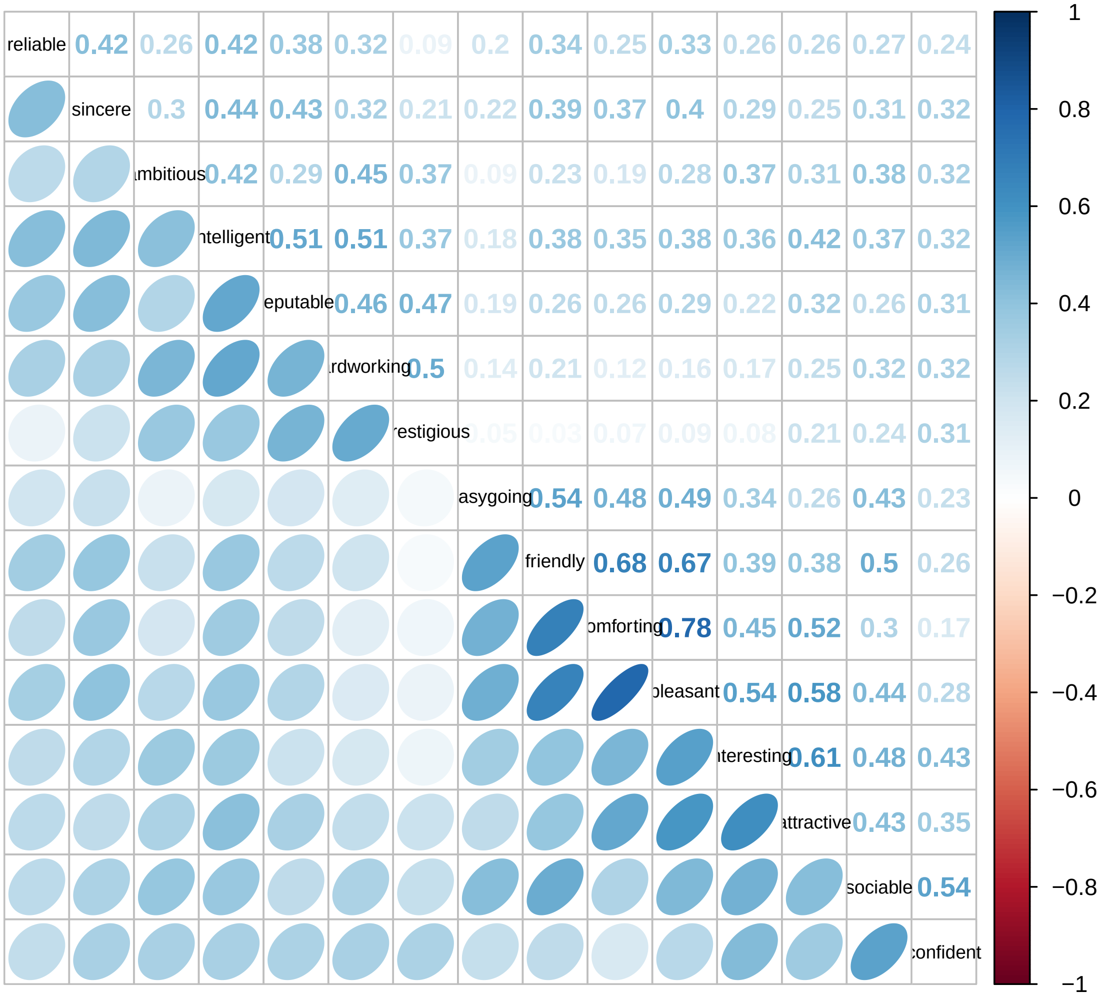


```r
#   Check factorability
KMO(cor(data.p[,2:16], method="spearman"))
```

```
## Kaiser-Meyer-Olkin factor adequacy
## Call: KMO(r = cor(data.p[, 2:16], method = "spearman"))
## Overall MSA =  0.88
## MSA for each item =
##    friendly    reliable   ambitious     sincere intelligent    sociable
##        0.88        0.91        0.88        0.91        0.93        0.87
## hardworking   easygoing prestigious   reputable   confident  comforting
##        0.87        0.88        0.79        0.89        0.89        0.86
##    pleasant interesting  attractive
##        0.87        0.89        0.87
```

```r
cortest.bartlett(cor(data.p[,2:16], method="spearman"),
                 length(unique(data.p$id)))
```

```
## $chisq
## [1] 835.4826
##
## $p.value
## [1] 3.659018e-114
##
## $df
## [1] 105
```

```r
#   Sample size
length(unique(data.p$id))
```

```
## [1] 141
```

```r
#       Minimum
ncol(data.p[,2:16]) * (ncol(data.p[,2:16]) - 1) / 2
```

```
## [1] 105
```

```r
#       Subjects-to-variables ratio
length(unique(data.p$id)) / ncol(data.p[,2:16])
```

```
## [1] 9.4
```

Exploratory Factor Analysis

```r
#   Determine number of factors
EFA.Comp.Data(data.p[,2:16], F.Max=7, Alpha=0.05, Graph=T, Spearman=T)
```

```
## Number of factors to retain:  5
```

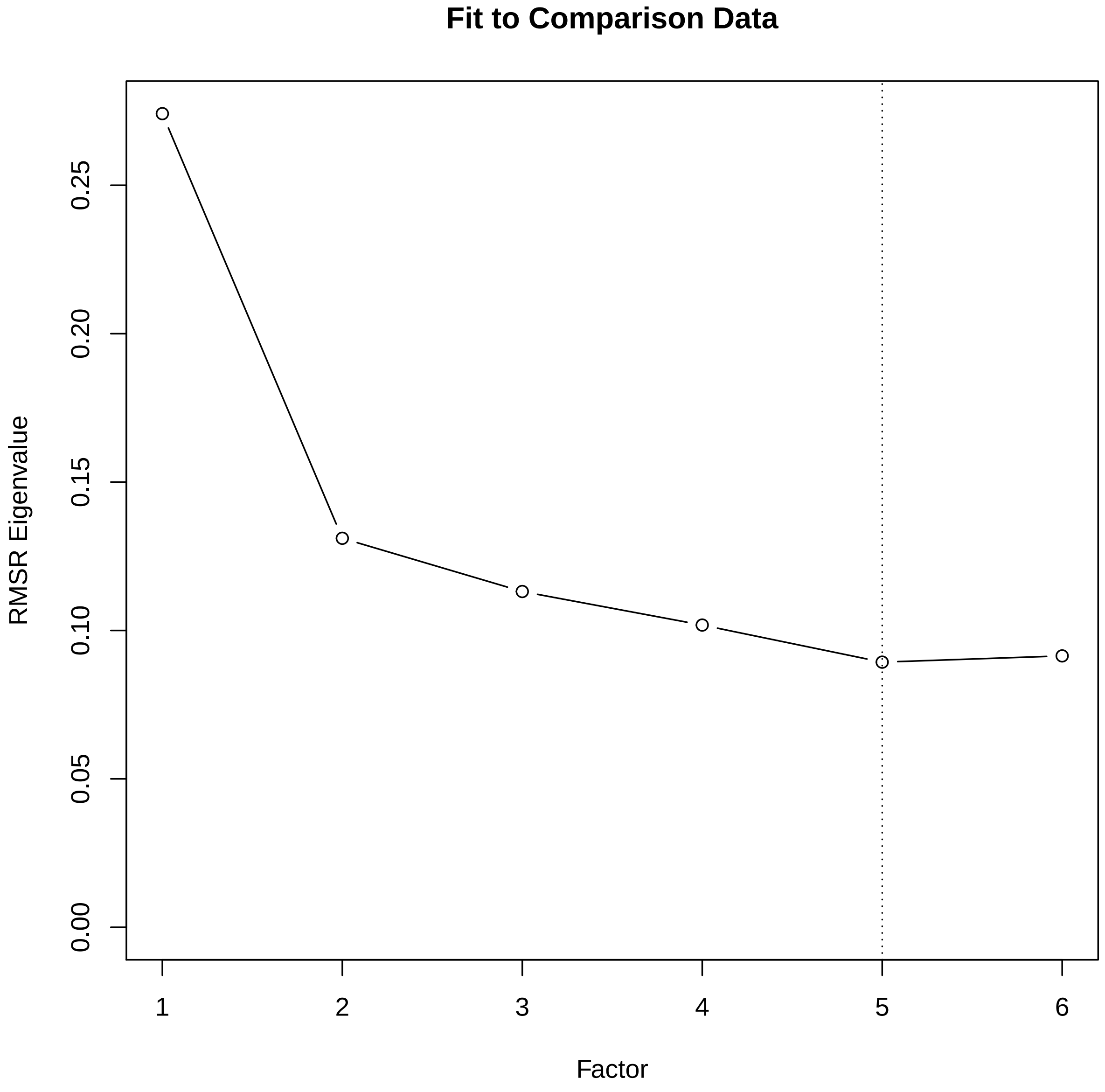

Possible overdimensionalization [see van der Eijk and Rose (2015)].

```
#   Run EFA with 5 factors
p.ea = efaUnrotate(data.p[,2:16], 5, estimator="WLSMV")
p.eao = oblqRotate(p.ea, method="oblimin")
p.eao
```

```
## Standardized Rotated Factor Loadings
##             factor1 factor2 factor3 factor4 factor5
## prestigious -0.903*
## comforting           0.921*  0.114*
## pleasant             0.707*         -0.219*
## friendly             0.649* -0.303*  0.142  -0.176*
```

```
## easygoing            0.495* -0.396*  0.132
## sociable                    -0.741* -0.158
## confident   -0.143  -0.140  -0.445* -0.260* -0.140
## interesting  0.109*         -0.216  -0.668*
## attractive           0.250*         -0.600*
## reliable     0.190*                         -0.710*
## intelligent -0.179                   -0.157* -0.573*
## sincere              0.156*                 -0.559*
## reputable   -0.300                          -0.515*
## hardworking -0.393*         -0.108          -0.448*
## ambitious   -0.221          -0.134  -0.265* -0.269*
##
## Factor Correlation
##             factor1     factor2    factor3    factor4    factor5
## factor1  1.00000000 -0.01000566  0.1646252  0.1931302  0.3874462
## factor2 -0.01000566  1.00000000 -0.3651514 -0.4086625 -0.3908013
## factor3  0.16462515 -0.36515144  1.0000000  0.3430200  0.3919922
## factor4  0.19313017 -0.40866254  0.3430200  1.0000000  0.3468018
## factor5  0.38744619 -0.39080127  0.3919922  0.3468018  1.0000000
##
## Method of rotation:  Oblimin Quartimin
## [1] "The standard errors are close but do not match with other packages. Be mindful when usin
```

```
inspect(p.ea, "rsquare")  # Communalities (r^2)
```

```
##    friendly    reliable   ambitious     sincere intelligent    sociable
##       0.708       0.454       0.350       0.421       0.560       0.730
## hardworking   easygoing prestigious   reputable   confident  comforting
##       0.514       0.442       0.816       0.487       0.438       0.861
##    pleasant interesting  attractive
##       0.773       0.681       0.616
```

```
# summary(p.eao)    # Uncomment to view test statistics (se, p, ci)
# fitMeasures(p.ea, c("chisq","df","pvalue","cfi","tli","rmsea","srmr"))
#      Uncomment to view listed fit measures

#   Run EFA with 4 factors
p.ea2 = efaUnrotate(data.p[,2:16], 4, estimator="WLSMV")
p.eao2 = oblqRotate(p.ea2, method="oblimin")
p.eao2
```

```
## Standardized Rotated Factor Loadings
##             factor1 factor2 factor3 factor4
## hardworking -0.722*         -0.110
## reputable   -0.706*  0.110
## intelligent -0.670*  0.121           0.129*
## prestigious -0.597* -0.227*
## sincere     -0.474*  0.317*
## ambitious   -0.422* -0.114  -0.167   0.240*
## reliable    -0.417*  0.253*
```

```
## friendly             0.800* -0.204* -0.116*
## comforting           0.775*  0.150*  0.219*
## pleasant             0.672*          0.315*
## easygoing            0.554* -0.308*
## sociable             0.132  -0.724*  0.127
## confident   -0.227* -0.104  -0.472*  0.220*
## attractive  -0.129*                  0.674*
## interesting          0.108  -0.243*  0.647*
##
## Factor Correlation
##            factor1    factor2    factor3    factor4
## factor1  1.0000000 -0.2839911  0.3738174 -0.3554088
## factor2 -0.2839911  1.0000000 -0.2936099  0.4768467
## factor3  0.3738174 -0.2936099  1.0000000 -0.3024718
## factor4 -0.3554088  0.4768467 -0.3024718  1.0000000
##
## Method of rotation:  Oblimin Quartimin
## [1] "The standard errors are close but do not match with other packages. Be mindful when usin
```

```
inspect(p.ea2, "rsquare")  # Communalities (r^2)
```

```
##    friendly    reliable   ambitious     sincere intelligent    sociable
##       0.730       0.286       0.360       0.387       0.577       0.735
## hardworking   easygoing prestigious   reputable   confident  comforting
##       0.532       0.422       0.373       0.504       0.448       0.766
##    pleasant interesting  attractive
##       0.777       0.636       0.633
```

```
# summary(p.eao2)    # Uncomment to view test statistics (se, p, ci)
# fitMeasures(p.ea2, c("chisq","df","pvalue","cfi","tli","rmsea","srmr"))
#       Uncomment to view listed fit measures

#   Run EFA with 3 factors
p.ea3 = efaUnrotate(data.p[,2:16], 3, estimator="WLSMV")
p.eao3 = oblqRotate(p.ea3, method="oblimin")
p.eao3
```

```
## Standardized Rotated Factor Loadings
##             factor1 factor2 factor3
## comforting   0.895*
## pleasant     0.800*          0.143*
## friendly     0.717*
## easygoing    0.482*          0.205*
## hardworking -0.116*  0.709*
## reputable    0.120   0.708*
## intelligent  0.181*  0.654*
## prestigious -0.233*  0.578*  0.138
## sincere      0.312*  0.465*
## reliable     0.253*  0.412*
## ambitious            0.383*  0.334*
```

```
## sociable     0.109           0.693*
## confident   -0.110*  0.162*  0.640*
## interesting  0.301*          0.572*
## attractive   0.329*  0.111   0.370*
##
## Factor Correlation
##           factor1   factor2   factor3
## factor1 1.0000000 0.3001387 0.4660821
## factor2 0.3001387 1.0000000 0.4845542
## factor3 0.4660821 0.4845542 1.0000000
##
## Method of rotation:  Oblimin Quartimin
## [1] "The standard errors are close but do not match with other packages. Be mindful when usin
```

```
inspect(p.ea3, "rsquare")  # Communalities (r^2)
```

```
##    friendly    reliable   ambitious     sincere intelligent    sociable
##       0.621       0.284       0.355       0.380       0.567       0.590
## hardworking   easygoing prestigious   reputable   confident  comforting
##       0.527       0.336       0.373       0.508       0.472       0.761
##    pleasant interesting  attractive
##       0.776       0.527       0.433
```

```
# summary(p.eao3)    # Uncomment to view test statistics (se, p, ci)
# fitMeasures(p.ea3, c("chisq","df","pvalue","cfi","tli","rmsea","srmr"))
#       Uncomment to view listed fit measures
```

```
#   Remove 'reliable' and repeat
EFA.Comp.Data(data.p[,c(2,4:16)], F.Max=7, Alpha=0.05, Graph=T, Spearman=T)
```

```
## Number of factors to retain:  4
```

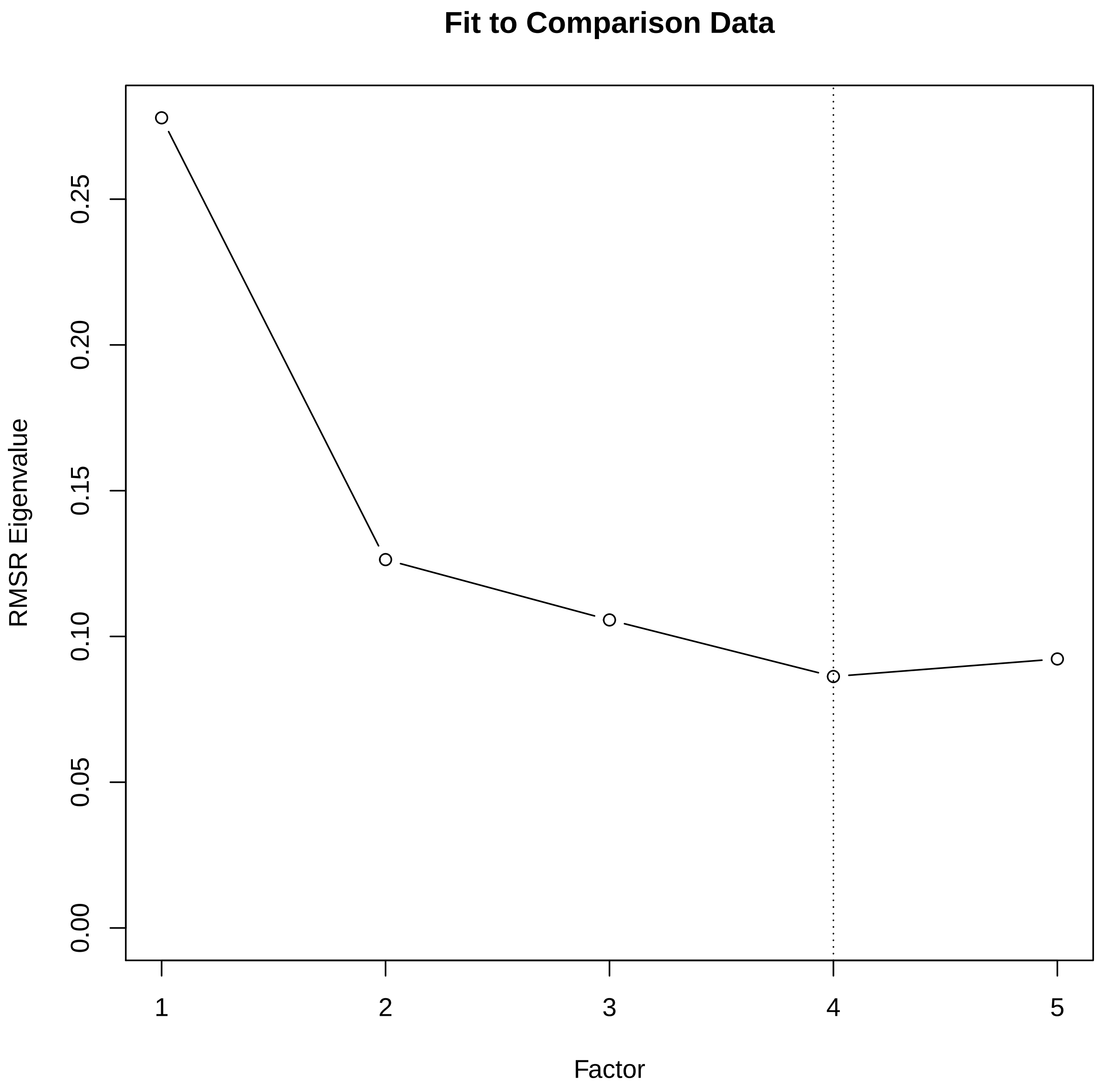


```
#   Run EFA with 4 factors
p.ea4 = efaUnrotate(data.p[,c(2,4:16)], 4, estimator="WLSMV")
p.eao4 = oblqRotate(p.ea4, method="oblimin")
p.eao4
```

```
## Standardized Rotated Factor Loadings
##             factor1 factor2 factor3 factor4
## comforting   0.844*          0.169*  0.142*
## friendly     0.801*         -0.230* -0.113*
## pleasant     0.697*                  0.282*
## easygoing    0.559*         -0.320*
## hardworking          0.734*
## prestigious -0.164*  0.690*
```

```
## reputable    0.148*  0.686*
## intelligent  0.152*  0.631*          0.135*
## ambitious            0.430* -0.117   0.265*
## sincere      0.307*  0.416*
## sociable     0.125   0.102  -0.674*  0.201
## confident   -0.104   0.251* -0.413*  0.285*
## interesting                 -0.167   0.751*
## attractive   0.140   0.145*          0.635*
##
## Factor Correlation
##            factor1    factor2    factor3    factor4
## factor1  1.0000000  0.2718299 -0.2557337  0.5103675
## factor2  0.2718299  1.0000000 -0.3132616  0.3607476
## factor3 -0.2557337 -0.3132616  1.0000000 -0.2870190
## factor4  0.5103675  0.3607476 -0.2870190  1.0000000
##
## Method of rotation:  Oblimin Quartimin
## [1] "The standard errors are close but do not match with other packages. Be mindful when usin
```

```
# inspect(p.ea4, "rsquare")  # Uncomment to view communalities (r^2)
# summary(p.eao4)    # Uncomment to view test statistics (se, p, ci)
# fitMeasures(p.ea4, c("chisq","df","pvalue","cfi","tli","rmsea","srmr"))
#       Uncomment to view listed fit measures

#   Spearman correlations with 'prestigious'
sort(cor(data.p[,2:16], method="spearman")[,9], decreasing=T)[-1]
```

```
##   reputable hardworking   ambitious intelligent   confident  attractive
##  0.44196597  0.44183131  0.36250767  0.32424627  0.27092905  0.23104377
##     sincere    sociable interesting    reliable  comforting    pleasant
##  0.22014853  0.19096221  0.10552712  0.08179552  0.07675211  0.07249824
##    friendly   easygoing
##  0.03667953  0.03483298
```

Retained:

- *hardworking*
- *reputable*
- *intelligent*
- *ambitious*

Free Listing

```
#   Example of terms given for each of three prompts before substitution
data.frame(L1=c(unlist(list.f[[1]][1])), L2=c(unlist(list.f[[1]][2]), rep(NA, 2)),
           L3=c(unlist(list.f[[1]][3]), rep(NA, 7)), stringsAsFactors=FALSE)
```

```
##                 L1          L2          L3
## 1             rank  successful  intelligent
## 2            class     wealthy    confident
```

```
## 3         standing    educated   successful
## 4          winning     skilled looked up to
## 5      first place   confident         <NA>
## 6  accomplishments      leader         <NA>
## 7     achievements  high class         <NA>
## 8            order intelligent         <NA>
## 9        hierarchy   high rank         <NA>
## 10     competition        <NA>         <NA>
## 11           order        <NA>         <NA>
```

```
#   Same terms following substitution
data.frame(L1=c(unlist(list.f.r[[1]][1])), L2=c(unlist(list.f.r[[1]][2]), rep(NA, 2)),
           L3=c(unlist(list.f.r[[1]][3]), rep(NA, 7)), stringsAsFactors=FALSE)
```

```
##                     L1                L2          L3
## 1  high social status        successful intelligent
## 2  high social status           wealthy   confident
## 3  high social status          educated  successful
## 4             awarded           skilled   admirable
## 5             awarded         confident        <NA>
## 6        accomplished            leader        <NA>
## 7        accomplished           refined        <NA>
## 8             ordered       intelligent        <NA>
## 9  high social status high social status        <NA>
## 10        competitive              <NA>        <NA>
## 11            ordered              <NA>        <NA>
```

```
#   Free list salience calculation
#       S = (sum((L - Rj + 1) / L)) / N
#       Source: Smith & Borgatti 1997
list.f.salience = data.frame(term=NA, salience=NA)
list.f.rank = data.frame(term=NA, rank=NA)
item = 1
s.n = length(list.f.r) * 3
for (i in 1:length(list.f.r)) {
    for (j in 1:3) {
        s.l = length(list.f.r[[i]][[j]])
        for (k in 1:s.l) {
            s.r = k
            s.rank = (s.l - s.r + 1) / s.l
            list.f.rank[item,] = c(list.f.r[[i]][[j]][[k]], s.rank)
            item = item + 1
        }
    }
}
list.f.salience = dplyr::summarize(group_by(list.f.rank, term),
                                   salience = sum(as.numeric(rank)) / s.n)

#   Sort by salience
```

```
list.f.salience = data.frame(list.f.salience[with(list.f.salience, order(-salience)),])
list.f.salience$term = factor(list.f.salience$term, levels=list.f.salience$term)

#   Salience deltas
list.f.salience$delta = round(c(NA, diff(list.f.salience$salience)), 4)
list.f.salience$deltadelta = round(c(NA, diff(list.f.salience$delta)), 4)
list.f.salience$propdelta = round(list.f.salience$delta /
                                      lag(list.f.salience$salience), 4)

#   Scree plot
p.list.f.salience = ggplot(list.f.salience, aes(x=term, y=salience)) +
    geom_bar(stat="identity") +
    scale_y_continuous(limits=c(0,0.4), expand=c(0,0)) +
    geom_vline(xintercept=7.5, color="#CC6677") +
    ggtitle(label="Full 303 terms") +
    labs(x="Term", y="Salience (Smith's S)") +
    theme(panel.grid.major.x = element_blank(), panel.grid.minor.x = element_blank(),
          axis.text.x=element_blank(), axis.ticks.x=element_blank())
p.list.f.salience
```

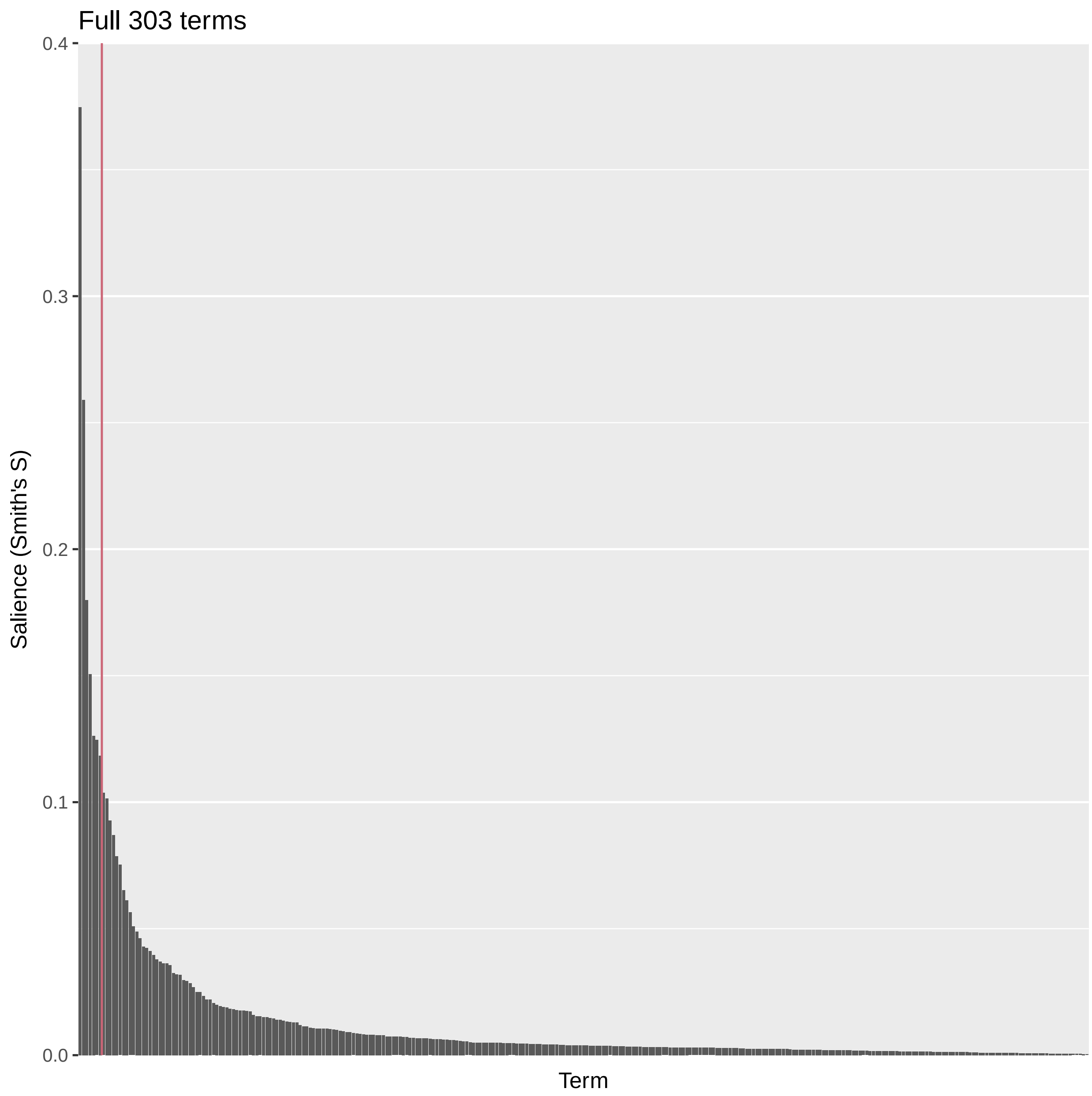


```
# ggsave("figure3a.pdf", p.list.f.salience, "pdf", width=8, height=8)

p.list.f.salience2 = ggplot(list.f.salience[1:20,], aes(x=term, y=salience)) +
    geom_bar(stat="identity") +
    scale_y_continuous(limits=c(0,0.4), expand=c(0,0)) +
    geom_vline(xintercept=7.5, color="#CC6677") +
    ggtitle(label="First 20 terms") +
    labs(x="Term", y="Salience (Smith's S)") +
    theme(axis.ticks.x=element_blank(),
          panel.grid.major.x = element_blank(), panel.grid.minor.x = element_blank(),
          axis.text.x=element_text(angle=90, hjust=0.9, vjust=0.3))
p.list.f.salience2
```

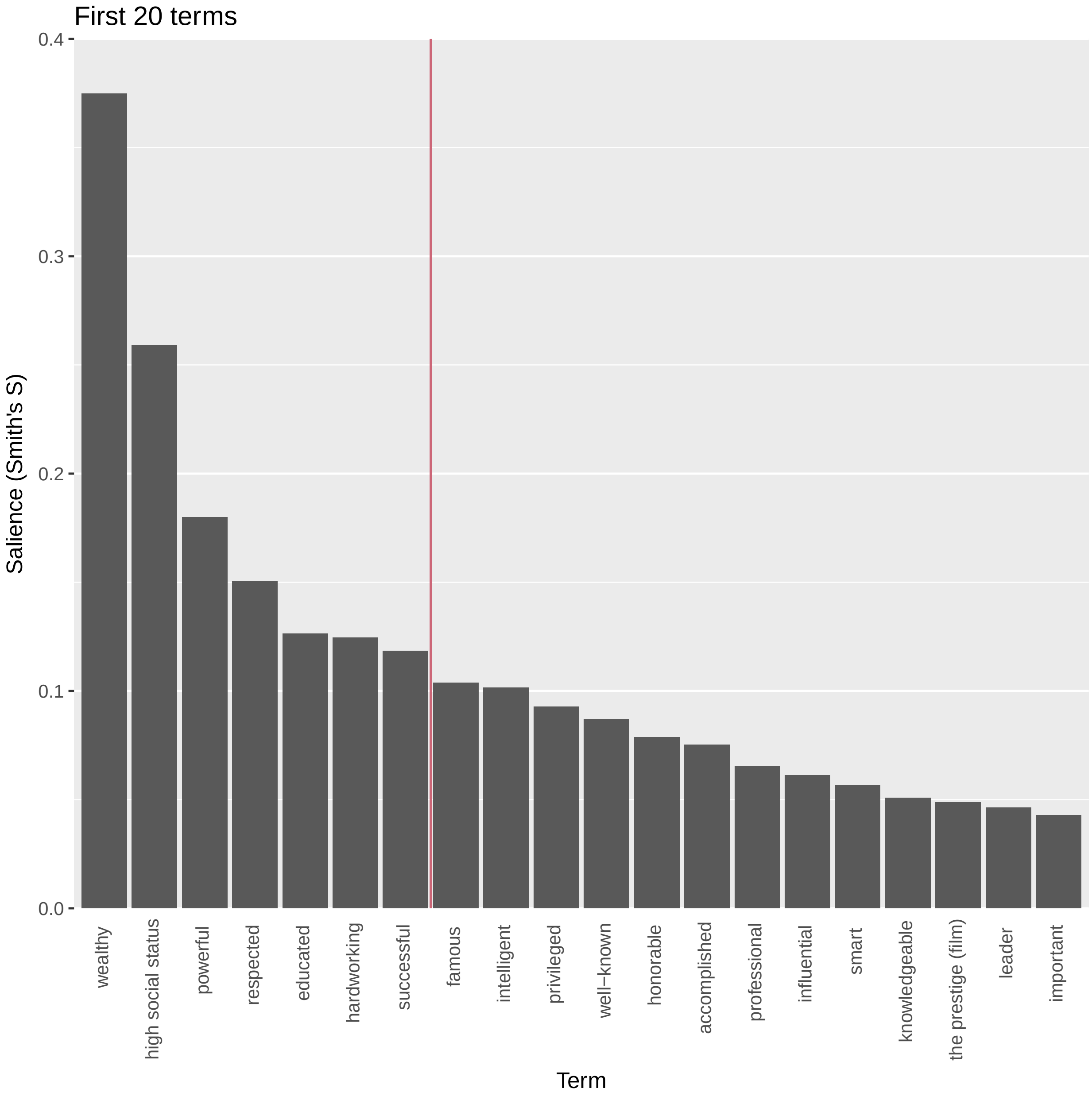


```
# ggsave("figure3b.pdf", p.list.f.salience2, "pdf", width=6, height=6)
```

Retained:

- *wealthy*
- *high social status*
- *powerful*
- *respected*
- *educated*
- *hardworking*
- *successful*

Exploratory Data Analysis

```
#   Prestige items
data.s.exp.prestige = summarySE(data.s.l[data.s.l$item %in%
                                              levels(data.s.l$item)[1:11],],
                                 measurevar="score", groupvars=c("item"))
data.s.exp.prestige$item = factor(data.s.exp.prestige$item,
                                  levels=levels(data.s.l$item)[1:11])

s.exp.prestige = ggplot(data.s.l[data.s.l$item %in% levels(data.s.l$item)[1:11],],
                        aes(x=item, y=score, fill=item)) +
    geom_violin() +
    geom_jitter(size=0.4, alpha=0.1, height=0.1, show.legend=F) +
    geom_errorbar(data=data.s.exp.prestige, aes(ymin=score-ci, ymax=score+ci),
                  width=1, size=0.5, position=position_dodge(0.9)) +
    scale_fill_manual(values=c("#332288","#6699CC","#88CCEE","#44AA99",
                               "#117733","#999933","#DDCC77","#661100",
                               "#CC6677","#882255","#AA4499")) +
    coord_cartesian(ylim=c(1,7)) +
    ggtitle("Prestige Item Distributions") +
    theme(axis.text.x=element_text(angle=-90, hjust=0, vjust=0.3))
s.exp.prestige
```

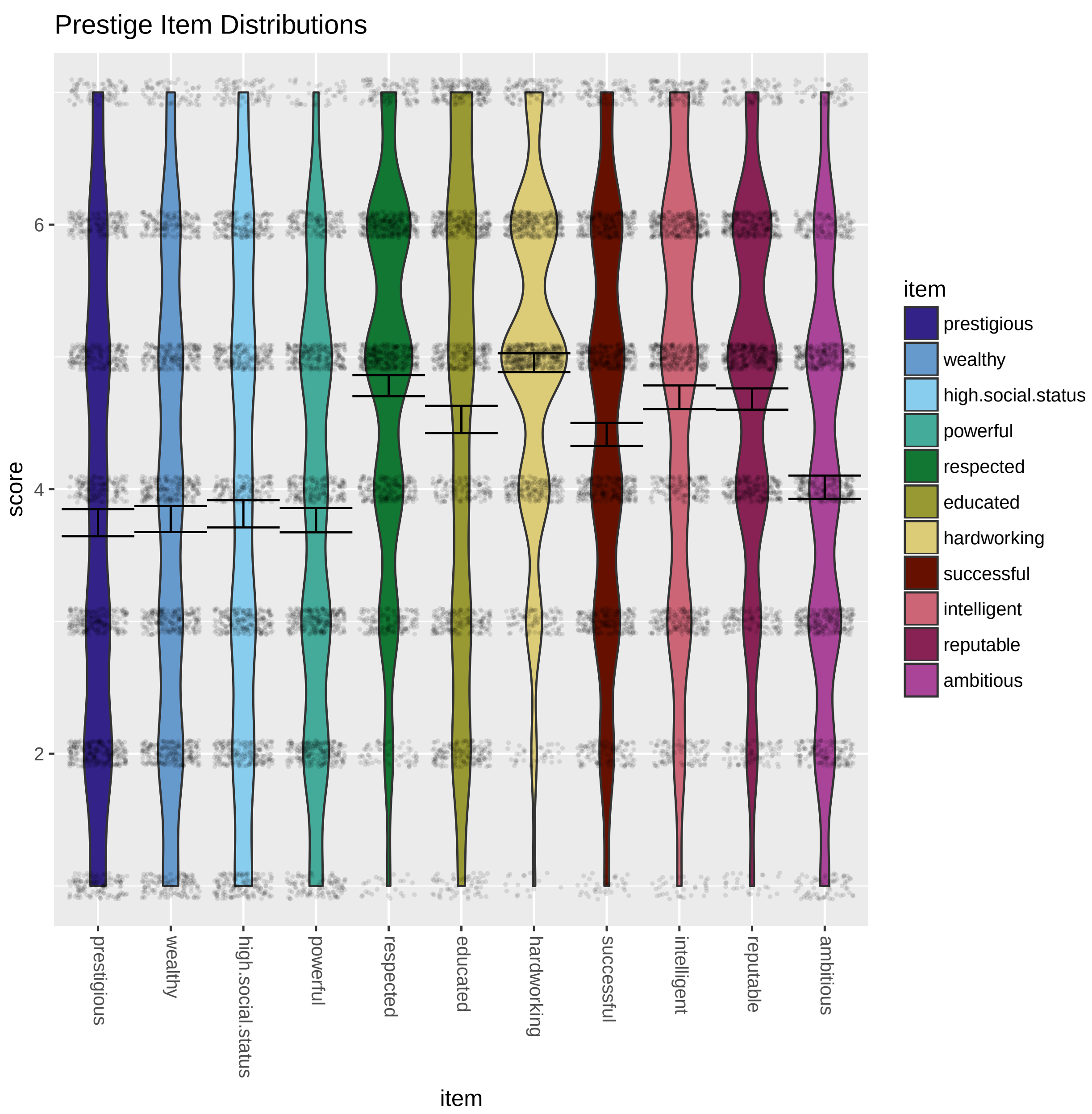


```
#   Solidarity items
data.s.exp.solidarity = summarySE(data.s.l[data.s.l$item %in%
                                              levels(data.s.l$item)[12:16],],
                                  measurevar="score", groupvars=c("item"))
data.s.exp.solidarity$item = factor(data.s.exp.solidarity$item,
                                    levels=levels(data.s.l$item)[12:16])

s.exp.solidarity = ggplot(data.s.l[data.s.l$item %in%
                                      levels(data.s.l$item)[12:16],],
                          aes(x=item, y=score, fill=item)) +
    geom_violin() +
    geom_jitter(size=0.4, alpha=0.1, height=0.1, show.legend=F) +
```

```
    geom_errorbar(data=data.s.exp.solidarity, aes(ymin=score-ci, ymax=score+ci),
                  width=1, size=0.5, position=position_dodge(0.9)) +
    scale_fill_manual(values=c("#332288","#88CCEE","#117733","#DDCC77","#CC6677")) +
    coord_cartesian(ylim=c(1,7)) +
    ggtitle("Solidarity Item Distributions") +
    theme(axis.text.x=element_text(angle=-90, hjust=0, vjust=0.3))
s.exp.solidarity
```

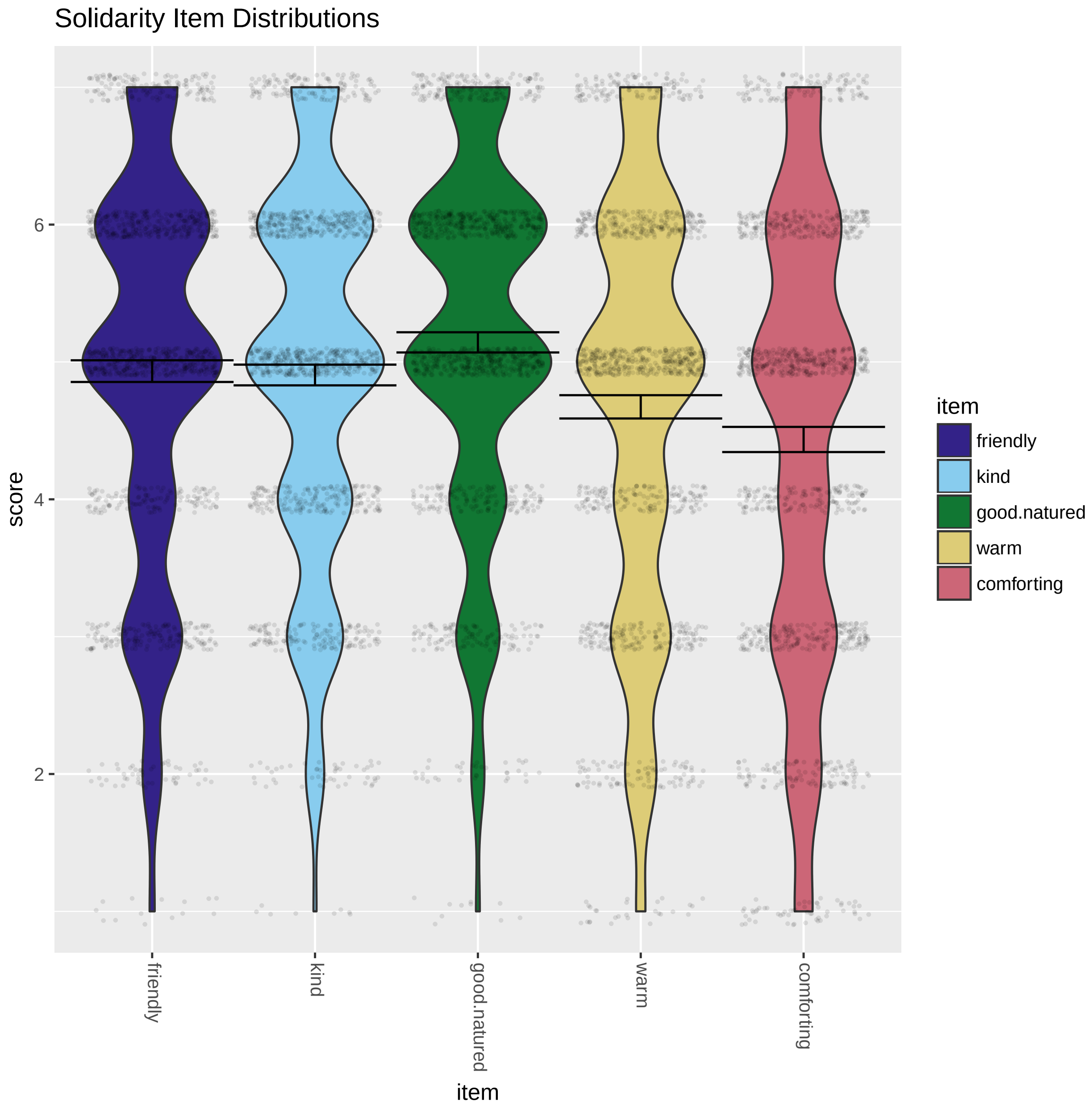


```
#   Dynamism items
data.s.exp.dynamism = summarySE(data.s.l[data.s.l$item %in%
                                          levels(data.s.l$item)[17:21],],
                                measurevar="score", groupvars=c("item"))
data.s.exp.dynamism$item = factor(data.s.exp.dynamism$item,
```

```
                                       levels=levels(data.s.l$item)[17:21])

s.exp.dynamism = ggplot(data.s.l[data.s.l$item %in% levels(data.s.l$item)[17:21],],
                        aes(x=item, y=score, fill=item)) +
    geom_violin() +
    geom_jitter(size=0.4, alpha=0.1, height=0.1, show.legend=F) +
    geom_errorbar(data=data.s.exp.dynamism, aes(ymin=score-ci, ymax=score+ci),
                  width=1, size=0.5, position=position_dodge(0.9)) +
    scale_fill_manual(values=c("#332288","#88CCEE","#117733","#DDCC77","#CC6677")) +
    coord_cartesian(ylim=c(1,7)) +
    ggtitle("Dynamism Item Distributions") +
    theme(axis.text.x=element_text(angle=-90, hjust=0, vjust=0.3))
s.exp.dynamism
```

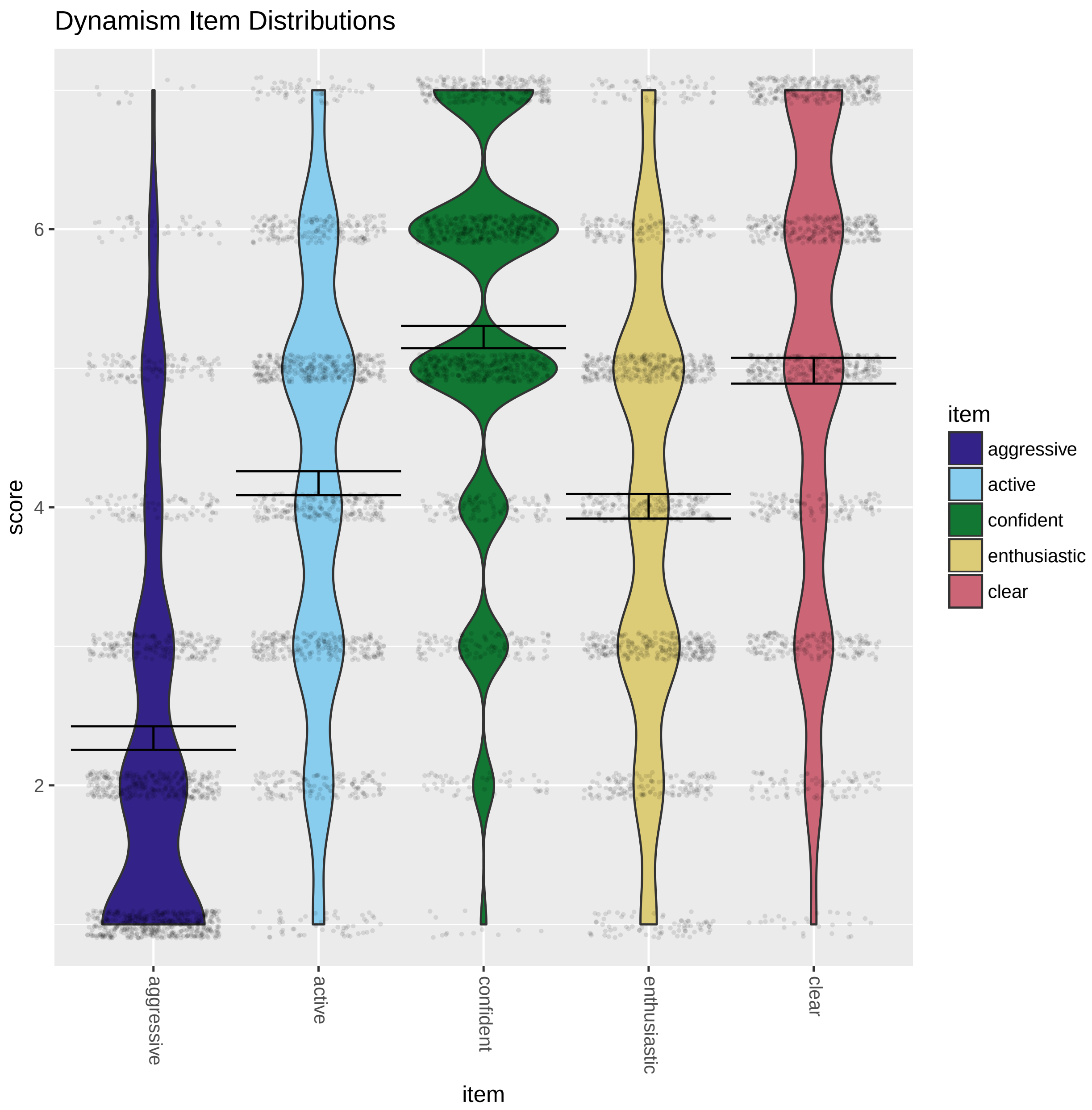


```
#   Demographic data
s.exp.country = ggplot(data.s[!duplicated(data.s[,1]),],
                       aes(x=country, fill=country)) +
    geom_bar(position=position_dodge()) +
    scale_fill_manual(values=c("#4477AA","#CC6677")) +
    ggtitle("Country") +
    xlab(NULL)

s.exp.age = ggplot(data.s[!duplicated(data.s[,1]),],
                   aes(x=age.o, fill=country)) +
    geom_bar(position=position_dodge()) +
    scale_fill_manual(values=c("#4477AA","#CC6677")) +
```

```
    ggtitle("Age") +
    xlab(NULL) +
    theme(axis.text.x=element_text(angle=-90, hjust=0, vjust=0.3))

s.exp.gender = ggplot(data.s[!duplicated(data.s[,1]),],
                      aes(x=gender, fill=country)) +
    geom_bar(position=position_dodge()) +
    scale_fill_manual(values=c("#4477AA","#CC6677")) +
    ggtitle("Gender") +
    xlab(NULL)

s.exp.ethnicity = ggplot(data.s[!duplicated(data.s[,1]),],
                         aes(x=ethnicity, fill=country)) +
    geom_bar(position=position_dodge()) +
    scale_fill_manual(values=c("#4477AA","#CC6677")) +
    ggtitle("Ethnicity") +
    xlab(NULL)

s.exp.locality = ggplot(data.s[!duplicated(data.s[,1]),],
                        aes(x=locality, fill=country)) +
    geom_bar(position=position_dodge()) +
    scale_fill_manual(values=c("#4477AA","#CC6677")) +
    ggtitle("Locality") +
    xlab(NULL)

s.exp.english = ggplot(data.s[!duplicated(data.s[,1]),],
                       aes(x=english, fill=country)) +
    geom_bar(position=position_dodge()) +
    scale_fill_manual(values=c("#4477AA","#CC6677")) +
    ggtitle("English") +
    xlab(NULL)

s.exp.education = ggplot(data.s[!duplicated(data.s[,1]),],
                         aes(x=education, fill=country)) +
    geom_bar(position=position_dodge()) +
    scale_fill_manual(values=c("#4477AA","#CC6677")) +
    ggtitle("Education") +
    xlab(NULL)

s.exp.occupation = ggplot(data.s[!duplicated(data.s[,1]),],
                          aes(x=occupation, fill=country)) +
    geom_bar(position=position_dodge()) +
    scale_fill_manual(values=c("#4477AA","#CC6677")) +
    ggtitle("Occupation") +
    xlab(NULL)

s.exp.income = ggplot(subset(data.s[!duplicated(data.s[,1]),], !is.na(income)),
                      aes(x=income, fill=country)) +
```

```
    geom_bar(position=position_dodge()) +
    scale_fill_manual(values=c("#4477AA","#CC6677")) +
    ggtitle("Income") +
    xlab(NULL)

multiplot(s.exp.country, s.exp.age, s.exp.gender, s.exp.ethnicity,
          s.exp.locality, s.exp.english, s.exp.education, s.exp.occupation,
          s.exp.income, layout=matrix(1:9, nrow=3, byrow=T))
```

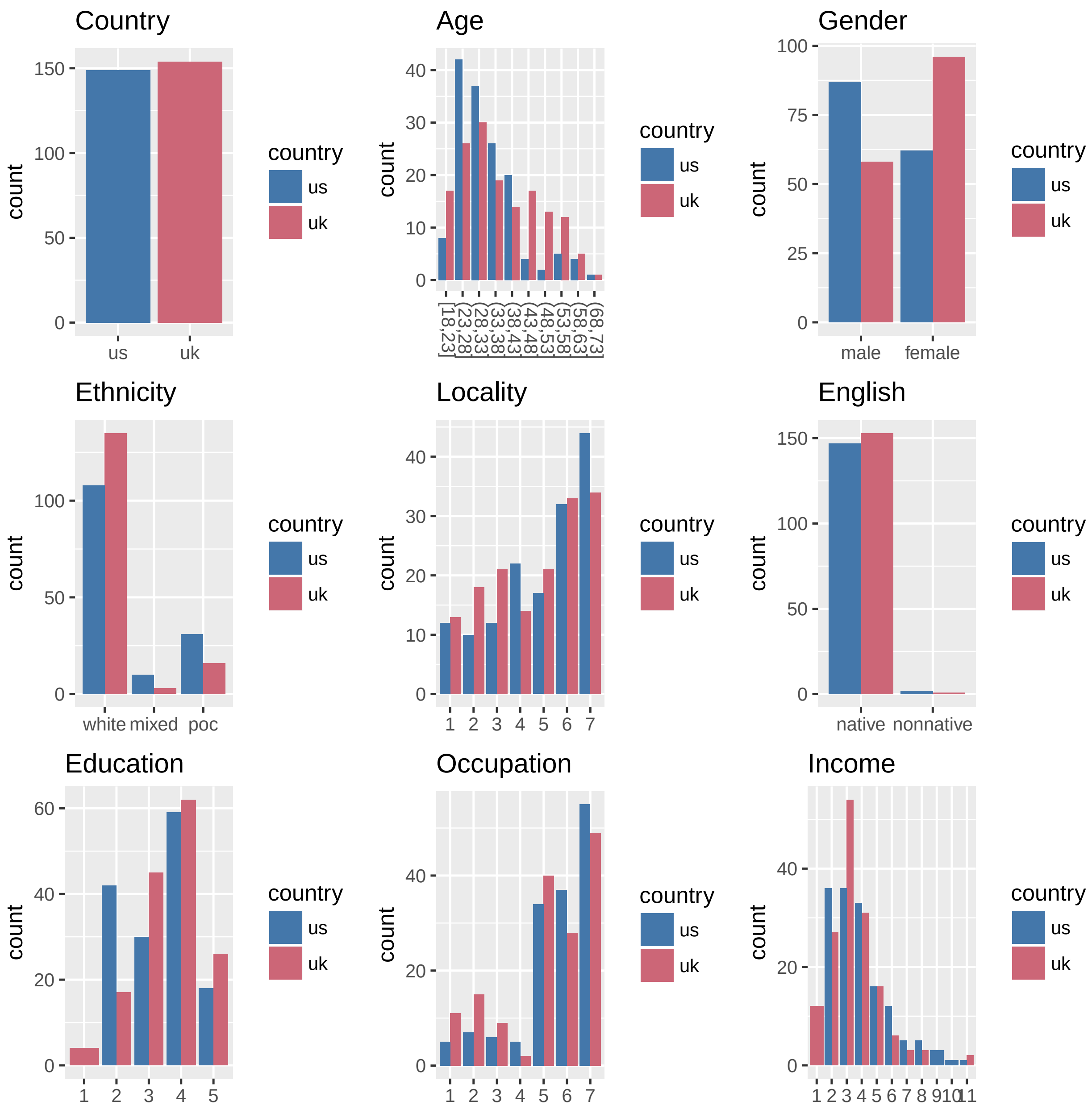

Exploratory Factor Analysis

Attitudinal Data

Checking Assumptions

```
#   Check for multivariate normality
mardiaTest(data.s[,c(2:4,6:22)], qqplot=T)
```

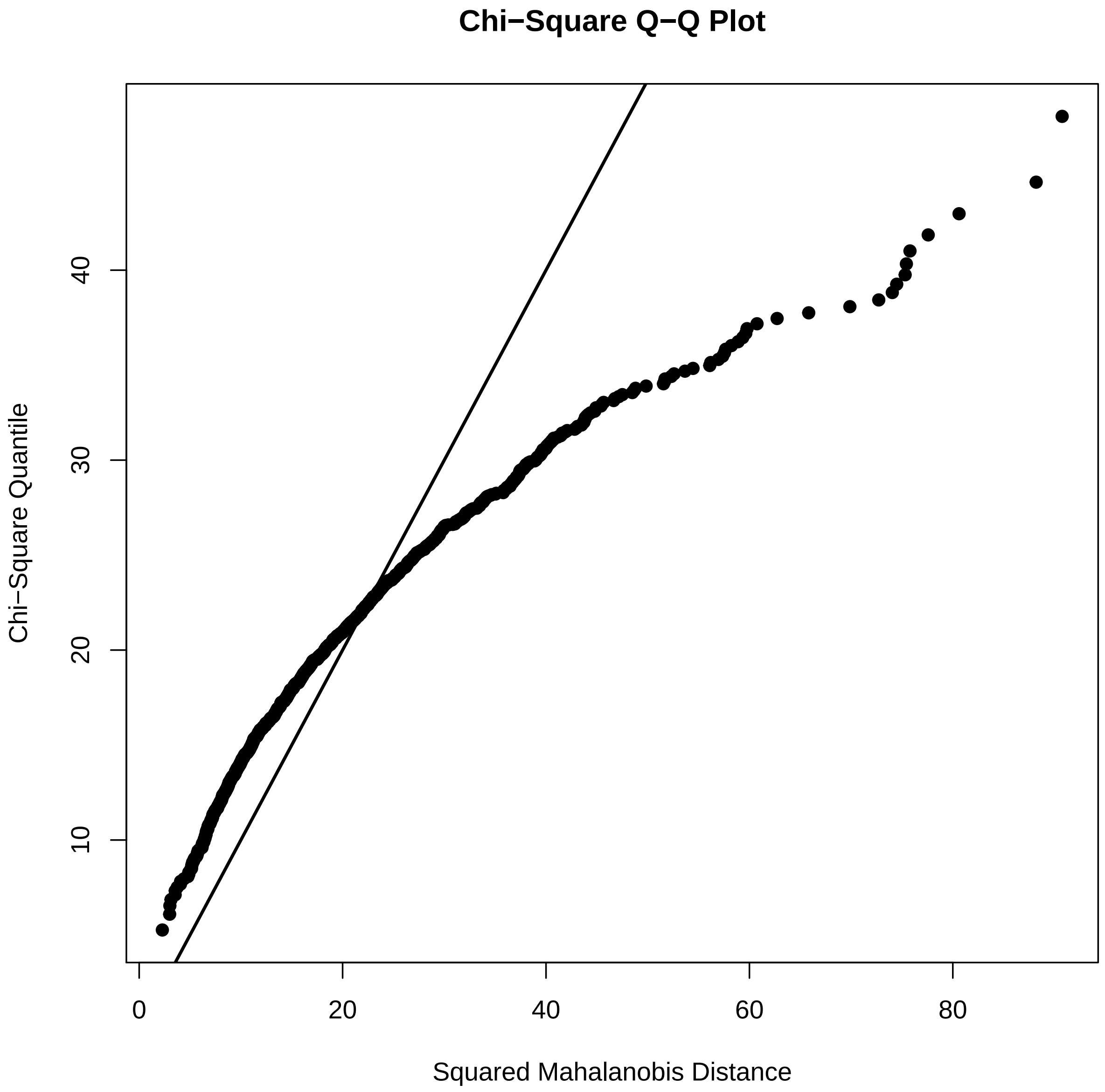


```
##    Mardia's Multivariate Normality Test
## ---------------------------------------
##    data : data.s[, c(2:4, 6:22)]
```

```
##
##    g1p            : 22.95316
##    chi.skew       : 4636.539
##    p.value.skew   : 1.064905e-306
##
##    g2p            : 543.4875
##    z.kurtosis     : 60.72504
##    p.value.kurt   : 0
##
##    chi.small.skew : 4649.111
##    p.value.small  : 1.589088e-308
##
##    Result          : Data are not multivariate normal.
## ---------------------------------------
```

```
hzTest(data.s[,c(2:4,6:22)], qqplot=F)
```

```
##   Henze-Zirkler's Multivariate Normality Test
## ---------------------------------------------
##   data : data.s[, c(2:4, 6:22)]
##
##   HZ      : 1.096427
##   p-value : 0
##
##   Result  : Data are not multivariate normal.
## ---------------------------------------------
```

```
roystonTest(data.s[,c(2:4,6:22)], qqplot=F)
```

```
##   Royston's Multivariate Normality Test
## ---------------------------------------------
##   data : data.s[, c(2:4, 6:22)]
##
##   H       : 1881.971
##   p-value : 0
##
##   Result  : Data are not multivariate normal.
## ---------------------------------------------
```

```
#   Check for multivariate outliers
head(mvOutlier(data.s[,c(2:4,6:22)], qqplot=T)$outlier, 10)
```

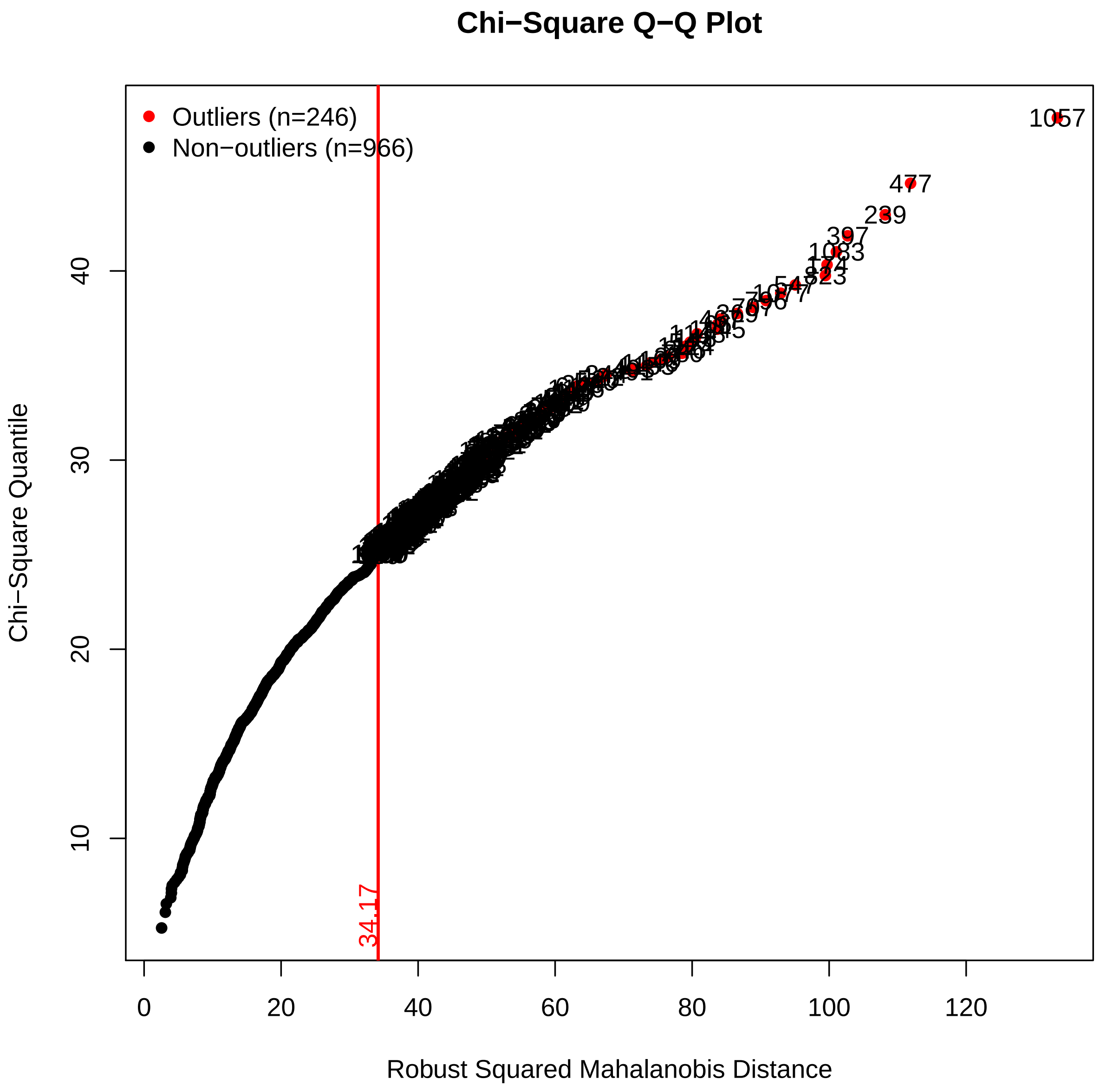


```
##      Observation Mahalanobis Distance Outlier
## 1057        1057              133.312    TRUE
## 477          477              111.890    TRUE
## 239          239              108.182    TRUE
## 397          397              102.714    TRUE
## 1083        1083              101.056    TRUE
## 174          174               99.698    TRUE
## 823          823               99.452    TRUE
## 547          547               95.056    TRUE
## 1077        1077               92.933    TRUE
## 796          796               90.794    TRUE
```

```
#       Check with most distant outlier removed
```

```r
head(mvOutlier(data.s[-c(1057),c(2:4,6:22)], qqplot=T)$outlier, 10)
```

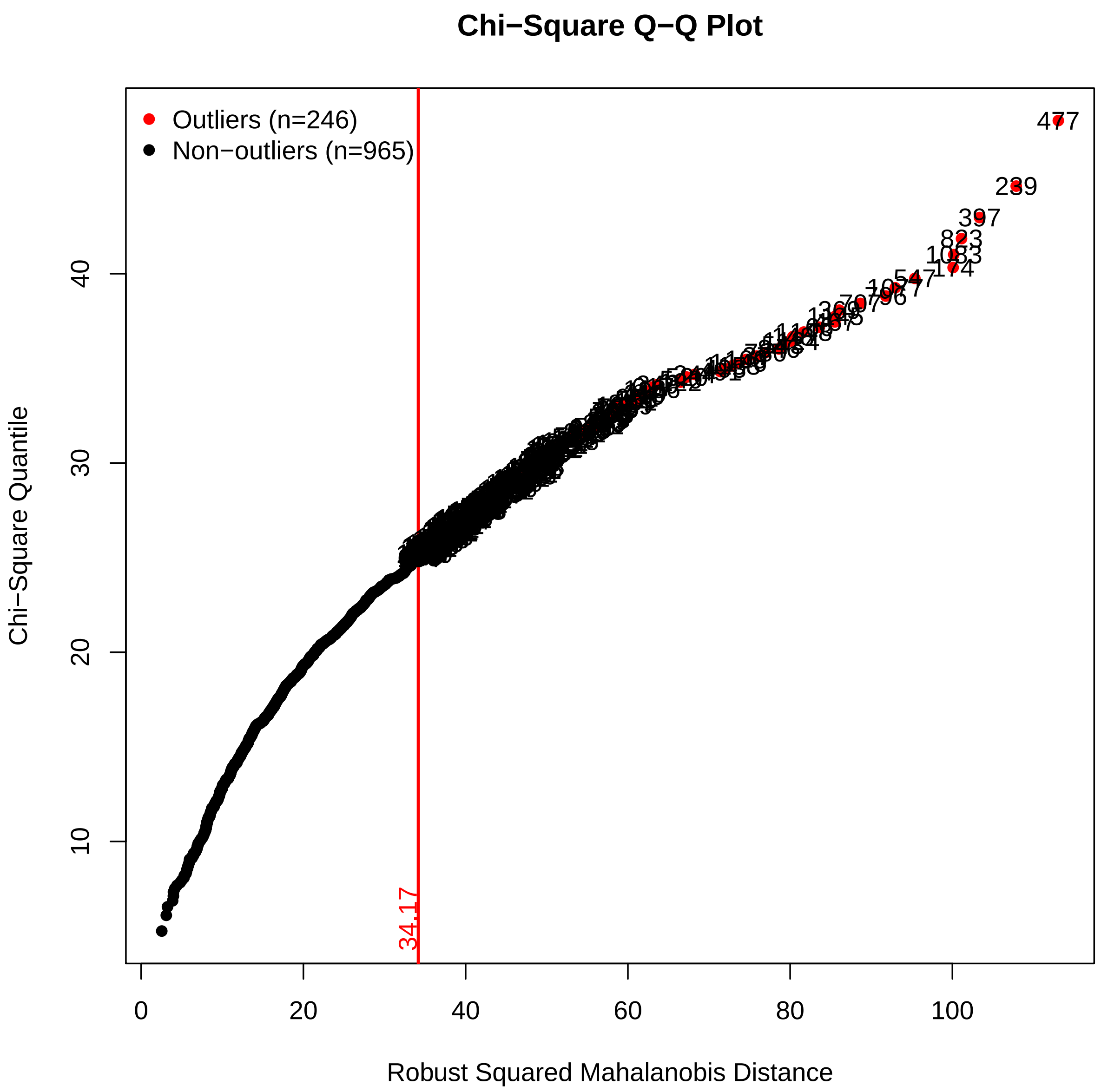


```
##      Observation Mahalanobis Distance Outlier
## 477          477              113.058    TRUE
## 239          239              107.872    TRUE
## 397          397              103.352    TRUE
## 823          823              101.125    TRUE
## 1083        1083              100.169    TRUE
## 174          174              100.090    TRUE
## 547          547               95.376    TRUE
## 1077        1077               92.958    TRUE
## 796          796               91.792    TRUE
## 707          707               88.660    TRUE
```

```
#       Identify participant
data.s[1057,]
```

```
##                id active aggressive ambitious clear comforting confident
## 1057 4785766935      1          5         1     1          7         5
##      educated enthusiastic friendly good.natured hardworking
## 1057        1            1        7            1           4
##      high.social.status intelligent kind powerful prestigious reputable
## 1057                  1           4    5        1           1         1
##      respected successful warm wealthy    accent country age   age.o
## 1057         3          3    1       1 england60      us  25 (23,28]
##      gender ethnicity locality english education occupation income
## 1057 female     white        5  native         4          1      3
```

```
data.s.to.remove = as.character(unique(data.s$id[1057]))

#       Remove participant
data.s = data.s[!as.character(data.s$id) %in% data.s.to.remove,]
data.s.o = data.s.o[!as.character(data.s.o$id) %in% data.s.to.remove,]
data.s.l = data.s.l[!as.character(data.s.l$id) %in% data.s.to.remove,]

#   Check for univariate normality
uniPlot(data.s[,c(2:4,6:22)], "histogram")
```

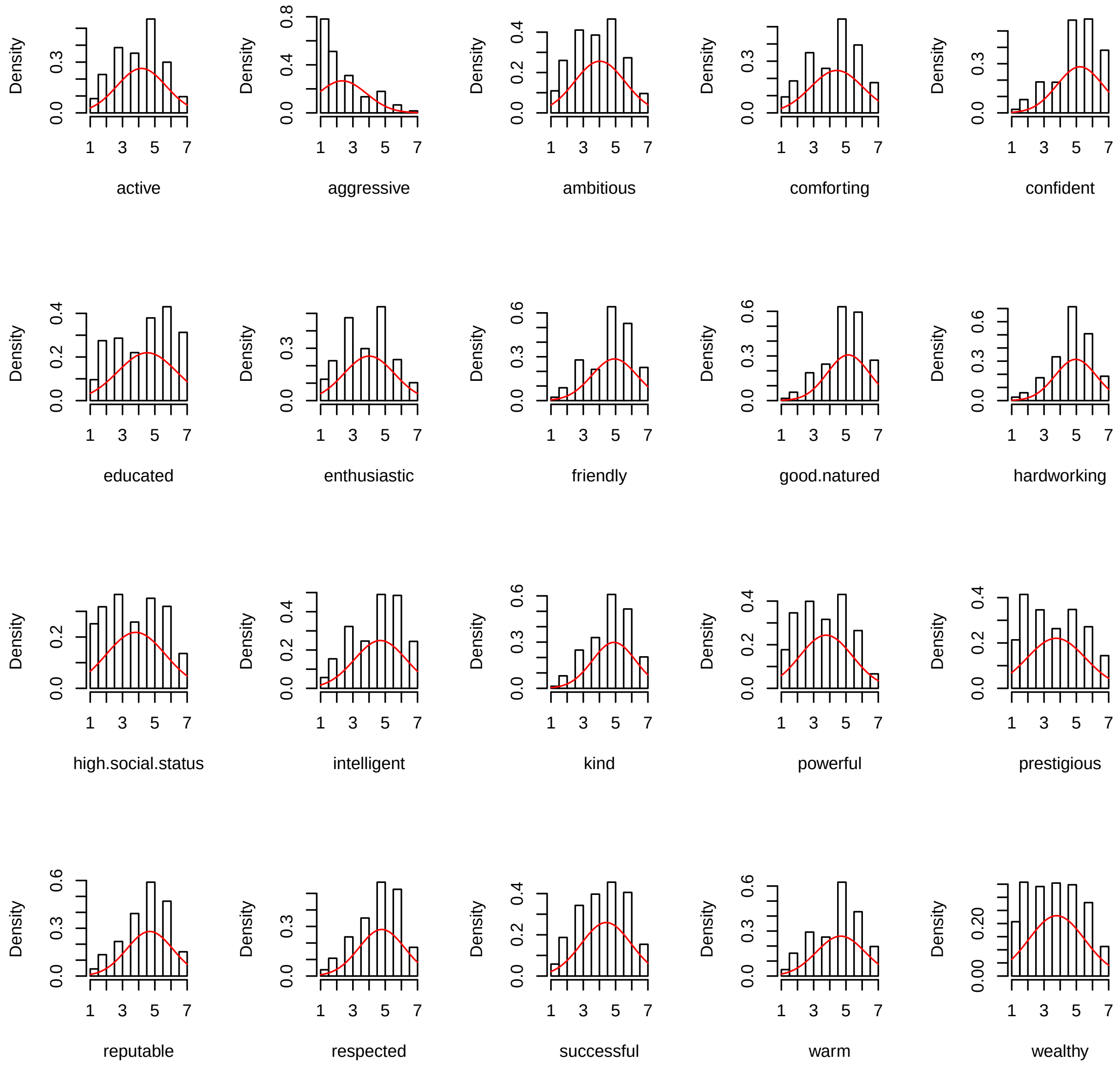


```
#   Check skewness
sort(apply(data.s[,c(2:4,6:22)], 2, e1071::skewness), decreasing=T)
```

```
##          aggressive        prestigious            wealthy
##          1.05426355         0.15508925         0.07255252
## high.social.status           powerful          ambitious
##          0.03963510         0.01558564        -0.06581404
##        enthusiastic             active         successful
##         -0.07072069        -0.19431743        -0.22430828
##            educated         comforting        intelligent
##         -0.29564471        -0.33376185        -0.42336725
##                warm               kind          reputable
##         -0.44427172        -0.44608836        -0.51838557
```

```
##          respected           friendly        hardworking
##        -0.54400273        -0.56644079        -0.63574414
##        good.natured          confident
##        -0.66516999        -0.75864126

#   Check kurtosis
sort(apply(data.s[,c(2:4,6:22)], 2, e1071::kurtosis, type=2), decreasing=T)

##        hardworking         aggressive       good.natured
##         0.39209464         0.21725606         0.13063320
##          confident          reputable          respected
##         0.05229877        -0.22943029        -0.23095621
##           friendly               kind               warm
##        -0.27147220        -0.34020059        -0.51522362
##        intelligent             active         successful
##        -0.72028226        -0.73075426        -0.74587000
##         comforting       enthusiastic          ambitious
##        -0.76184589        -0.76986973        -0.78689384
##           powerful            wealthy           educated
##        -0.99746816        -1.02775294        -1.10539166
##        prestigious high.social.status
##        -1.12278921        -1.15141466

#   Check for linear relationships and correlations
corrplot.mixed(lavCor(data.s.o[,c(2:4,6:22)]), lower="ellipse", upper="number",
               order="hclust", hclust.method="ward.D",
               tl.cex=0.7, tl.pos="d", tl.col="black")
```

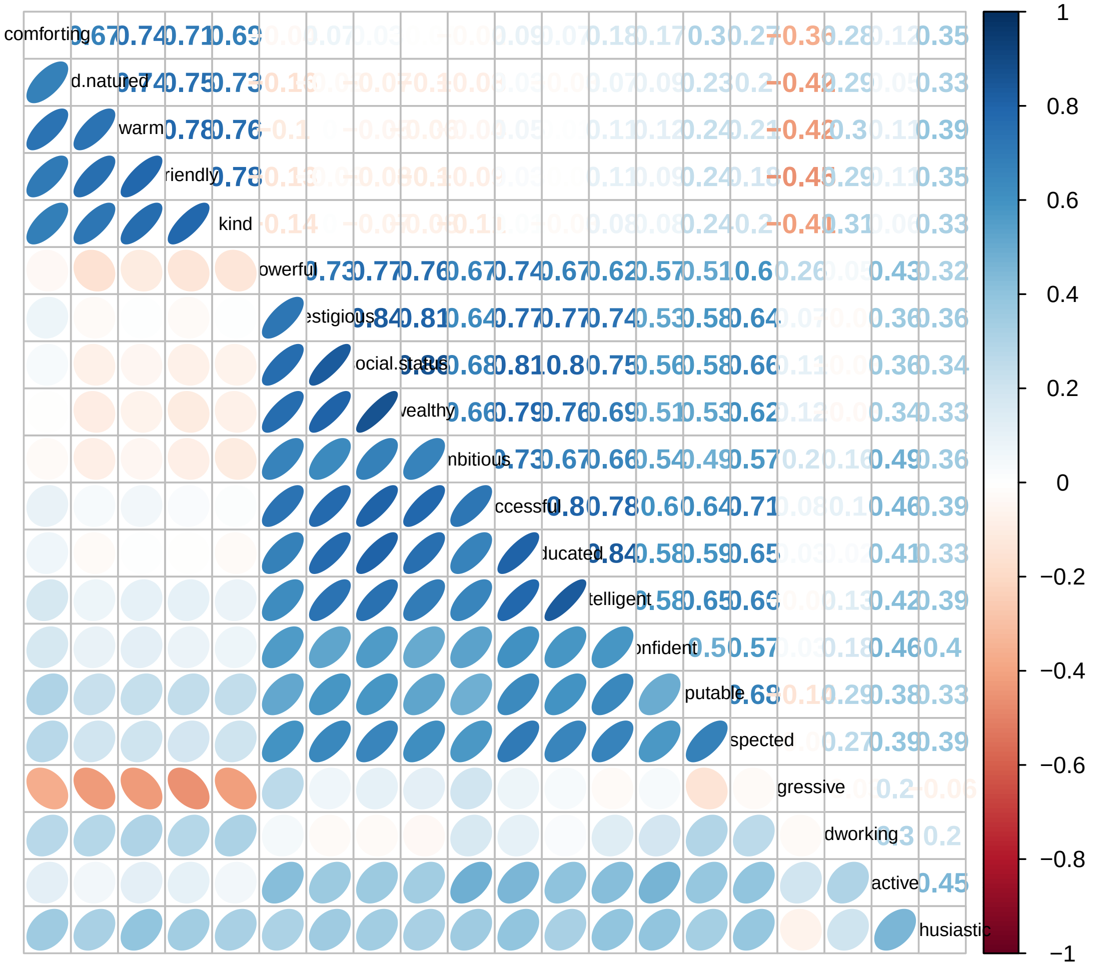

```
#   Check factorability
KMO(lavCor(data.s.o[,c(2:4,6:22)]))

## Kaiser-Meyer-Olkin factor adequacy
## Call: KMO(r = lavCor(data.s.o[, c(2:4, 6:22)]))
## Overall MSA =  0.95
## MSA for each item =
##            active      aggressive       ambitious
##              0.91            0.87            0.97
##        comforting       confident        educated
##              0.94            0.97            0.94
##      enthusiastic        friendly    good.natured
##              0.94            0.91            0.93
```

```
##        hardworking high.social.status        intelligent
##               0.81               0.95               0.94
##               kind           powerful        prestigious
##               0.92               0.96               0.97
##          reputable          respected         successful
##               0.96               0.97               0.97
##               warm            wealthy
##               0.91               0.95
```

```
cortest.bartlett(lavCor(data.s.o[,c(2:4,6:22)]), nrow(data.s.o) / 4)
```

```
## $chisq
## [1] 4954.941
##
## $p.value
## [1] 0
##
## $df
## [1] 190
```

```
#   Sample size
length(unique(data.s$id))
```

```
## [1] 302
```

```
#       Minimum
ncol(data.s[,c(2:4,6:22)]) * (ncol(data.s[,c(2:4,6:22)]) - 1) / 2
```

```
## [1] 190
```

```
#       Subjects-to-variables ratio
length(unique(data.s$id)) / ncol(data.s[,c(2:4,6:22)])
```

```
## [1] 15.1
```

Exploratory Factor Analysis

```
#   Determine number of factors
EFA.Comp.Data(data.s[,c(2:4,6:22)], F.Max=10, Graph=T, Spearman=T)
```

```
## Number of factors to retain:  6
```

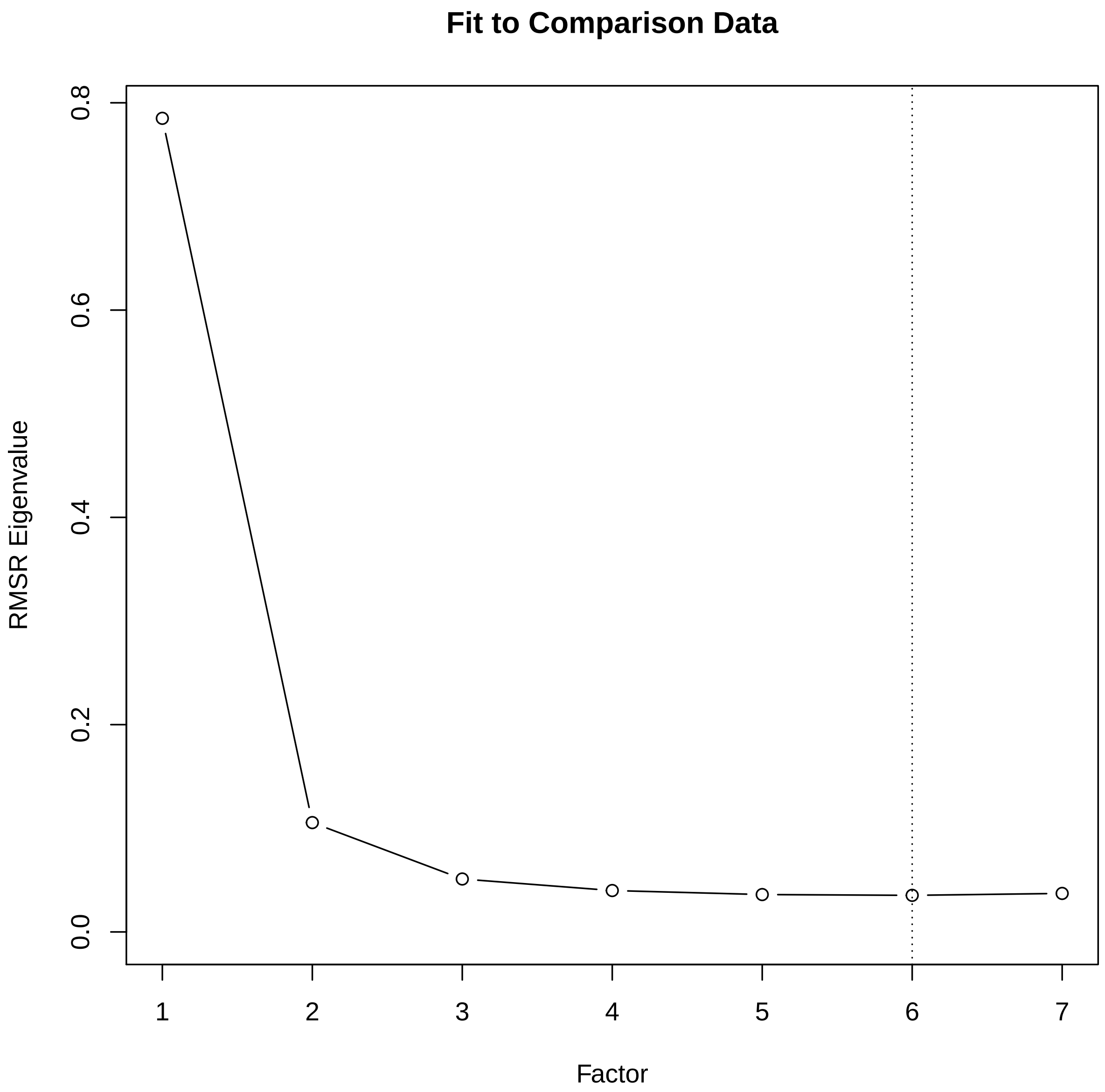

Likely overdimensionalization [see van der Eijk and Rose (2015)]. 6-factor solution is singular.

```
#   Run EFA with 5 factors
s.ea = efaUnrotate(data.s.o[,c(2:4,6:22)], 5, estimator="WLSMV")
s.eao = oblqRotate(s.ea, method="oblimin")
s.eao
```

```
## Standardized Rotated Factor Loadings
##                    factor1 factor2 factor3 factor4 factor5
## wealthy             0.928*
## high.social.status  0.841*                 -0.145
## powerful            0.819*          0.151           -0.110
## prestigious         0.745*                 -0.201
```

```
## successful          0.532*          0.110  -0.315  -0.134
## respected           0.493   0.146          -0.137  -0.393
## ambitious           0.371* -0.136   0.293  -0.247  -0.142
## warm                        0.901*
## friendly                    0.876*
## kind                        0.867*
## good.natured                0.825*
## comforting                  0.806*
## aggressive          0.239  -0.440*  0.333   0.331*
## active                              0.661  -0.194  -0.139
## enthusiastic        0.192   0.445*  0.457           0.120
## confident           0.252           0.256  -0.251  -0.171
## intelligent         0.100                  -0.770
## educated            0.220                  -0.735
## hardworking        -0.223*  0.145*  0.214          -0.562
## reputable           0.302   0.145          -0.268  -0.447*
##
## Factor Correlation
##             factor1     factor2     factor3    factor4    factor5
## factor1  1.00000000 -0.07225248  0.38948530 -0.7753126 -0.3106309
## factor2 -0.07225248  1.00000000  0.00993274 -0.1513245 -0.2997499
## factor3  0.38948530  0.00993274  1.00000000 -0.2522357 -0.3286324
## factor4 -0.77531255 -0.15132452 -0.25223571  1.0000000  0.3587661
## factor5 -0.31063087 -0.29974986 -0.32863244  0.3587661  1.0000000
##
## Method of rotation:  Oblimin Quartimin
## [1] "The standard errors are close but do not match with other packages. Be mindful when usin
```

```
inspect(s.ea, "rsquare")    # Communalities (r^2)
```

```
##             active         aggressive          ambitious
##              0.599              0.408              0.657
##         comforting          confident           educated
##              0.662              0.500              0.867
##       enthusiastic           friendly       good.natured
##              0.492              0.796              0.705
##        hardworking high.social.status        intelligent
##              0.443              0.879              0.837
##               kind           powerful        prestigious
##              0.757              0.758              0.794
##          reputable          respected         successful
##              0.669              0.711              0.819
##               warm            wealthy
##              0.791              0.848
```

```
# summary(s.eao)   # Uncomment to view test statistics (se, p, ci)
# fitMeasures(s.ea, c("chisq","df","pvalue","cfi","tli","rmsea","srmr"))
#       Uncomment to view listed fit measures

#   Run EFA with 4 factors
```

```r
s.ea2 = efaUnrotate(data.s.o[,c(2:4,6:22)], 4, estimator="WLSMV")
s.eao2 = oblqRotate(s.ea2, method="oblimin")
s.eao2
```

```
## Standardized Rotated Factor Loadings
##                    factor1 factor2 factor3 factor4
## high.social.status  0.966*                  0.121*
## wealthy             0.935*                  0.197*
## prestigious         0.922*
## educated            0.898*                 -0.234*
## successful          0.839*          0.133*
## intelligent         0.829*                 -0.294*
## powerful            0.745* -0.126*  0.192*  0.186*
## respected           0.662*  0.197*  0.184* -0.109*
## ambitious           0.623* -0.140*  0.319*
## reputable           0.596*  0.226*  0.152* -0.209*
## confident           0.510*          0.306*
## warm                        0.896*
## friendly                    0.886*
## kind                        0.869*
## good.natured                0.836*
## comforting                  0.811*
## aggressive                 -0.494*  0.368*  0.213*
## enthusiastic        0.267*  0.394*  0.331*  0.174*
## active              0.176*          0.650*
## hardworking        -0.169*  0.211*  0.503* -0.208*
##
## Factor Correlation
##             factor1     factor2     factor3     factor4
## factor1  1.00000000  0.01724318  0.41148152 -0.04344915
## factor2  0.01724318  1.00000000  0.15268665 -0.25169523
## factor3  0.41148152  0.15268665  1.00000000 -0.05082161
## factor4 -0.04344915 -0.25169523 -0.05082161  1.00000000
##
## Method of rotation:  Oblimin Quartimin
## [1] "The standard errors are close but do not match with other packages. Be mindful when usin
```

```r
inspect(s.ea2, "rsquare")    # Communalities (r^2)
```

```
##             active         aggressive          ambitious
##              0.549              0.402              0.657
##         comforting          confident           educated
##              0.662              0.502              0.849
##       enthusiastic           friendly       good.natured
##              0.438              0.795              0.705
##        hardworking high.social.status        intelligent
##              0.361              0.879              0.827
##               kind           powerful        prestigious
##              0.752              0.746              0.795
```

```
##          reputable          respected        successful
##              0.601              0.658             0.819
##               warm            wealthy
##              0.789              0.848
```

```
# summary(s.eao2)   # Uncomment to view test statistics (se, p, ci)
# fitMeasures(s.ea2, c("chisq","df","pvalue","cfi","tli","rmsea","srmr"))
#       Uncomment to view listed fit measures

#   Run EFA with 3 factors
s.ea3 = efaUnrotate(data.s.o[,c(2:4,6:22)], 3, estimator="WLSMV")
s.eao3 = oblqRotate(s.ea3, method="oblimin")
s.eao3
```

```
## Standardized Rotated Factor Loadings
##                    factor1 factor2 factor3
## high.social.status  0.976*         -0.118*
## wealthy             0.943*         -0.115*
## prestigious         0.928*         -0.105*
## educated            0.887*
## successful          0.842*          0.128*
## intelligent         0.839*  0.131*
## powerful            0.758* -0.188*  0.147*
## respected           0.673*  0.226*  0.175*
## ambitious           0.624* -0.149*  0.315*
## reputable           0.613*  0.285*  0.147*
## confident           0.513*          0.306*
## friendly                    0.890*
## warm                        0.875*
## kind                        0.868*
## good.natured                0.841*
## comforting                  0.804*
## aggressive                 -0.558*  0.346*
## active              0.163*          0.674*
## hardworking        -0.141*  0.257*  0.478*
## enthusiastic        0.257*  0.327*  0.330*
##
## Factor Correlation
##            factor1    factor2   factor3
## factor1 1.00000000 0.01325577 0.4195897
## factor2 0.01325577 1.00000000 0.1783944
## factor3 0.41958970 0.17839441 1.0000000
##
## Method of rotation:  Oblimin Quartimin
## [1] "The standard errors are close but do not match with other packages. Be mindful when usin
```

```
inspect(s.ea3, "rsquare")    # Communalities (r^2)
```

```
##             active         aggressive         ambitious
##              0.567              0.349             0.657
```

```
##         comforting          confident           educated
##              0.661              0.501              0.796
##       enthusiastic           friendly       good.natured
##              0.393              0.794              0.705
##        hardworking high.social.status        intelligent
##              0.301              0.874              0.769
##               kind           powerful        prestigious
##              0.751              0.712              0.790
##          reputable          respected         successful
##              0.574              0.651              0.817
##               warm            wealthy
##              0.782              0.822
```

```
# summary(s.eao3)   # Uncomment to view test statistics (se, p, ci)
# fitMeasures(s.ea3, c("chisq","df","pvalue","cfi","tli","rmsea","srmr"))
#       Uncomment to view listed fit measures
```

```
#   Remove 'hardworking' and repeat
EFA.Comp.Data(data.s[,c(2:4,6:11,13:22)], F.Max=7, Graph=T, Spearman=T)
```

```
## Number of factors to retain:  3
```

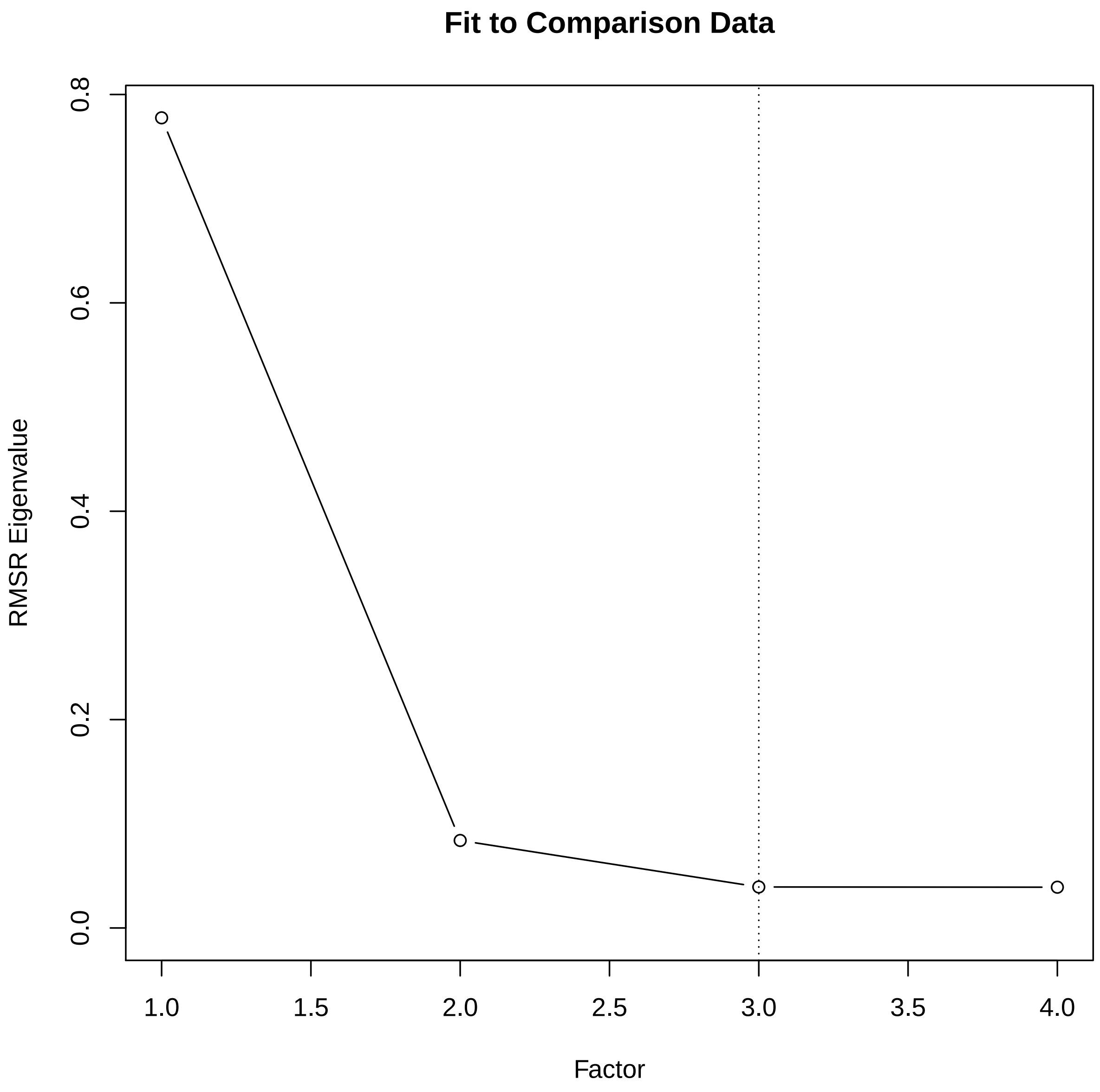


```
#   Run EFA with 3 factors
s.ea4 = efaUnrotate(data.s.o[,c(2:4,6:11,13:22)], 3, estimator="WLSMV")
s.eao4 = oblqRotate(s.ea4, method="oblimin")
s.eao4
```

```
## Standardized Rotated Factor Loadings
##                    factor1 factor2 factor3
## high.social.status  0.981*         -0.110*
## wealthy             0.947* -0.107* -0.105*
## prestigious         0.925*
## educated            0.872*
## intelligent         0.828*  0.128*
## successful          0.828*          0.138*
```

```
## powerful            0.744* -0.183*  0.157*
## respected           0.692*  0.237*  0.118*
## reputable           0.646*  0.295*
## ambitious           0.603* -0.134*  0.317*
## confident           0.487*          0.317*
## friendly                    0.890*
## warm                        0.877*
## kind                        0.870*
## good.natured                0.841*
## comforting                  0.805*
## aggressive                 -0.539*  0.355*
## active                              0.721*
## enthusiastic        0.193*  0.342*  0.401*
##
## Factor Correlation
##            factor1    factor2   factor3
## factor1 1.00000000 0.02296203 0.4834422
## factor2 0.02296203 1.00000000 0.1277011
## factor3 0.48344222 0.12770107 1.0000000
##
## Method of rotation:  Oblimin Quartimin
## [1] "The standard errors are close but do not match with other packages. Be mindful when usin
```

```
inspect(s.ea4, "rsquare")    # Communalities (r^2)
```

```
##              active         aggressive          ambitious
##               0.595              0.348              0.652
##          comforting          confident           educated
##               0.661              0.502              0.794
##        enthusiastic           friendly       good.natured
##               0.428              0.795              0.705
## high.social.status        intelligent               kind
##               0.874              0.768              0.751
##            powerful        prestigious          reputable
##               0.711              0.786              0.566
##           respected         successful               warm
##               0.643              0.817              0.784
##             wealthy
##               0.821
```

```
# summary(s.eao4)   # Uncomment to view test statistics (se, p, ci)
# fitMeasures(s.ea4, c("chisq","df","pvalue","cfi","tli","rmsea","srmr"))
#       Uncomment to view listed fit measures
```

```
#   Remove 'confident' and repeat
s.ea5 = efaUnrotate(data.s.o[,c(2:4,6,8:11,13:22)], 3, estimator="WLSMV")
s.eao5 = oblqRotate(s.ea5, method="oblimin")
s.eao5
```

```
## Standardized Rotated Factor Loadings
```

```
##                    factor1 factor2 factor3
## high.social.status  0.978*         -0.106*
## wealthy             0.940* -0.109*
## prestigious         0.920*
## educated            0.870*
## intelligent         0.827*  0.130*
## successful          0.826*          0.145*
## powerful            0.747* -0.179*  0.149*
## respected           0.703*  0.241*
## reputable           0.653*  0.298*
## ambitious           0.603* -0.127*  0.322*
## friendly                    0.890*
## warm                        0.878*
## kind                        0.869*
## good.natured                0.841*
## comforting                  0.806*
## aggressive                 -0.533*  0.367*
## active              0.103*          0.715*
## enthusiastic        0.197*  0.351*  0.398*
##
## Factor Correlation
##            factor1    factor2   factor3
## factor1 1.00000000 0.02190515 0.4725023
## factor2 0.02190515 1.00000000 0.1079256
## factor3 0.47250230 0.10792563 1.0000000
##
## Method of rotation:  Oblimin Quartimin
## [1] "The standard errors are close but do not match with other packages. Be mindful when usin
```

```
inspect(s.ea5, "rsquare")    # Communalities (r^2)
```

```
##             active         aggressive          ambitious
##              0.596              0.357              0.655
##         comforting           educated       enthusiastic
##              0.661              0.794              0.428
##           friendly       good.natured high.social.status
##              0.795              0.705              0.874
##        intelligent               kind           powerful
##              0.768              0.751              0.707
##        prestigious          reputable          respected
##              0.786              0.566              0.637
##         successful               warm            wealthy
##              0.819              0.785              0.820
```

```
# summary(s.eao5)   # Uncomment to view test statistics (se, p, ci)
# fitMeasures(s.ea5, c("chisq","df","pvalue","cfi","tli","rmsea","srmr"))
#       Uncomment to view listed fit measures

#   Remove 'ambitious' and repeat
s.ea6 = efaUnrotate(data.s.o[,c(2:3,6,8:11,13:22)], 3, estimator="WLSMV")
```

```
s.eao6 = oblqRotate(s.ea6, method="oblimin")
s.eao6
```

```
## Standardized Rotated Factor Loadings
##                     factor1 factor2 factor3
## educated             0.962*         -0.201*
## intelligent          0.921*         -0.192*
## high.social.status   0.893* -0.106*  0.105*
## successful           0.880*
## prestigious          0.861*
## wealthy              0.847* -0.127*  0.150*
## respected            0.736*  0.237*
## powerful             0.734* -0.142*  0.260*
## reputable            0.694*  0.278*
## warm                         0.893*
## friendly                     0.890*
## kind                         0.865*
## good.natured                 0.837*
## comforting                   0.808*
## enthusiastic         0.278*  0.442*  0.383*
## aggressive                  -0.441*  0.409*
## active               0.341*  0.151*  0.394*
##
## Factor Correlation
##            factor1     factor2    factor3
## factor1 1.00000000  0.05991897  0.2780254
## factor2 0.05991897  1.00000000 -0.1007078
## factor3 0.27802542 -0.10070784  1.0000000
##
## Method of rotation:  Oblimin Quartimin
## [1] "The standard errors are close but do not match with other packages. Be mindful when usin
```

```
inspect(s.ea6, "rsquare")    # Communalities (r^2)
```

```
##             active         aggressive         comforting
##              0.364              0.394              0.660
##           educated       enthusiastic           friendly
##              0.852              0.460              0.795
##       good.natured high.social.status        intelligent
##              0.705              0.862              0.806
##               kind           powerful        prestigious
##              0.749              0.727              0.781
##          reputable          respected         successful
##              0.573              0.638              0.805
##               warm            wealthy
##              0.787              0.818
```

```
# summary(s.eao6)   # Uncomment to view test statistics (se, p, ci)
# fitMeasures(s.ea6, c("chisq","df","pvalue","cfi","tli","rmsea","srmr"))
#       Uncomment to view listed fit measures
```

```r
#   Check whether 'reputable' and 'respected' are near collinearity
s.p.corr = round(lavCor(data.s.o[,c(2:3,6,8:11,13:22)]),
                 3)[c(4,8,9,11,12,13,14,15,17),c(4,8,9,11,12,13,14,15,17)]
dimnames(s.p.corr) = rep(list(c("eductd","highss","intllg","powrfl","prstgs",
                              "reptbl","rspctd","sccssf","wealth")), 2)
s.p.corr
```

```
##        eductd highss intllg powrfl prstgs reptbl rspctd sccssf wealth
## eductd  1.000  0.803  0.840  0.671  0.774  0.593  0.650  0.804  0.756
## highss  0.803  1.000  0.746  0.767  0.835  0.584  0.659  0.807  0.861
## intllg  0.840  0.746  1.000  0.621  0.738  0.648  0.663  0.780  0.694
## powrfl  0.671  0.767  0.621  1.000  0.726  0.514  0.596  0.739  0.765
## prstgs  0.774  0.835  0.738  0.726  1.000  0.581  0.644  0.774  0.808
## reptbl  0.593  0.584  0.648  0.514  0.581  1.000  0.679  0.639  0.529
## rspctd  0.650  0.659  0.663  0.596  0.644  0.679  1.000  0.709  0.618
## sccssf  0.804  0.807  0.780  0.739  0.774  0.639  0.709  1.000  0.789
## wealth  0.756  0.861  0.694  0.765  0.808  0.529  0.618  0.789  1.000
```

```r
#   Plot factor loadings
# pdf("figure4.pdf", width=8, height=8)

s.all.3d = scatterplot3d(s.eao6@loading,
                       xlim=c(-.5,1), ylim=c(-.5,1), zlim=c(-.5,1),
                       pch=16, type="h", box=F, asp=1, mar=c(5,3,0,3)+0.1,
                       color=c("#332288","#332288","#117733","#CC6677",
                               "#332288","#117733","#117733","#4477AA",
                               "#CC6677","#117733","#4477AA","#000000",
                               "#DDCC77","#DDCC77","gray","#117733",
                               "#4477AA"),
                       xlab="Factor 1", ylab="Factor 2", zlab="Factor 3",
                       angle=130)
s.all.3d.coords = s.all.3d$xyz.convert(s.eao6@loading)
rownames(s.eao6@loading)[7] = c("good-natured")
rownames(s.eao6@loading)[8] = c("high social\nstatus")
p.text.loc = list(x = mean(s.all.3d.coords$x[c(4,8,9,11,12,13,14,15,17)]),
                  y = 2.5)
s.text.loc = list(x = mean(s.all.3d.coords$x[c(3,6,7,10,16)]),
                  y = 2.5)
d.text.loc = list(x = mean(s.all.3d.coords$x[c(1,2,5)]),
                  y = 2.5)
text(s.all.3d.coords$x + c(    0, 0.05,0.225, 0.125,
                               0,-0.15, -0.3, 0.225,
                           -0.22,  0.1,    0,-0.225,
                            -0.1, -0.1,-0.25,     0,
                               0) * 1.25,
     s.all.3d.coords$y + c(  0.1,  0.1,    0,   0.1,
                             0.1, 0.07,-0.03,     0,
                            0.04,0.075,  0.1, -0.06,
                             0.1,0.125,0.025,   0.1,
```

```
                                 0.1),
     rownames(s.eao6@loading), cex=0.75, asp=1)
text(p.text.loc$x, p.text.loc$y, "Prestige", cex=1, asp=1)
text(s.text.loc$x, s.text.loc$y, "Solidarity", cex=1, asp=1)
text(d.text.loc$x, d.text.loc$y, "Dynamism", cex=1, asp=1)
```

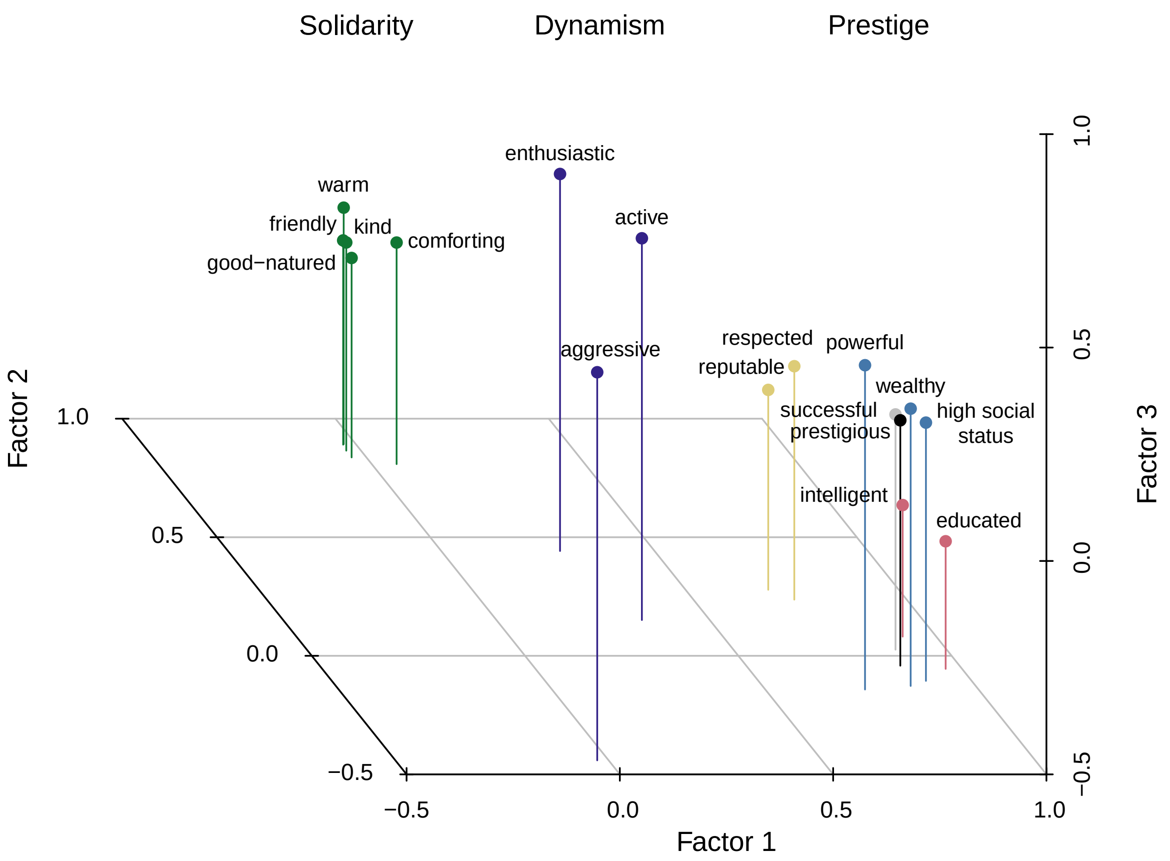


```
# dev.off()
```

Internal Prestige Domain

```
#   Determine number of factors
EFA.Comp.Data(data.s[,c(8,13,14,16,18:20,22)], F.Max=5, Graph=T, Spearman=T)
```

```
## Number of factors to retain:  3
```

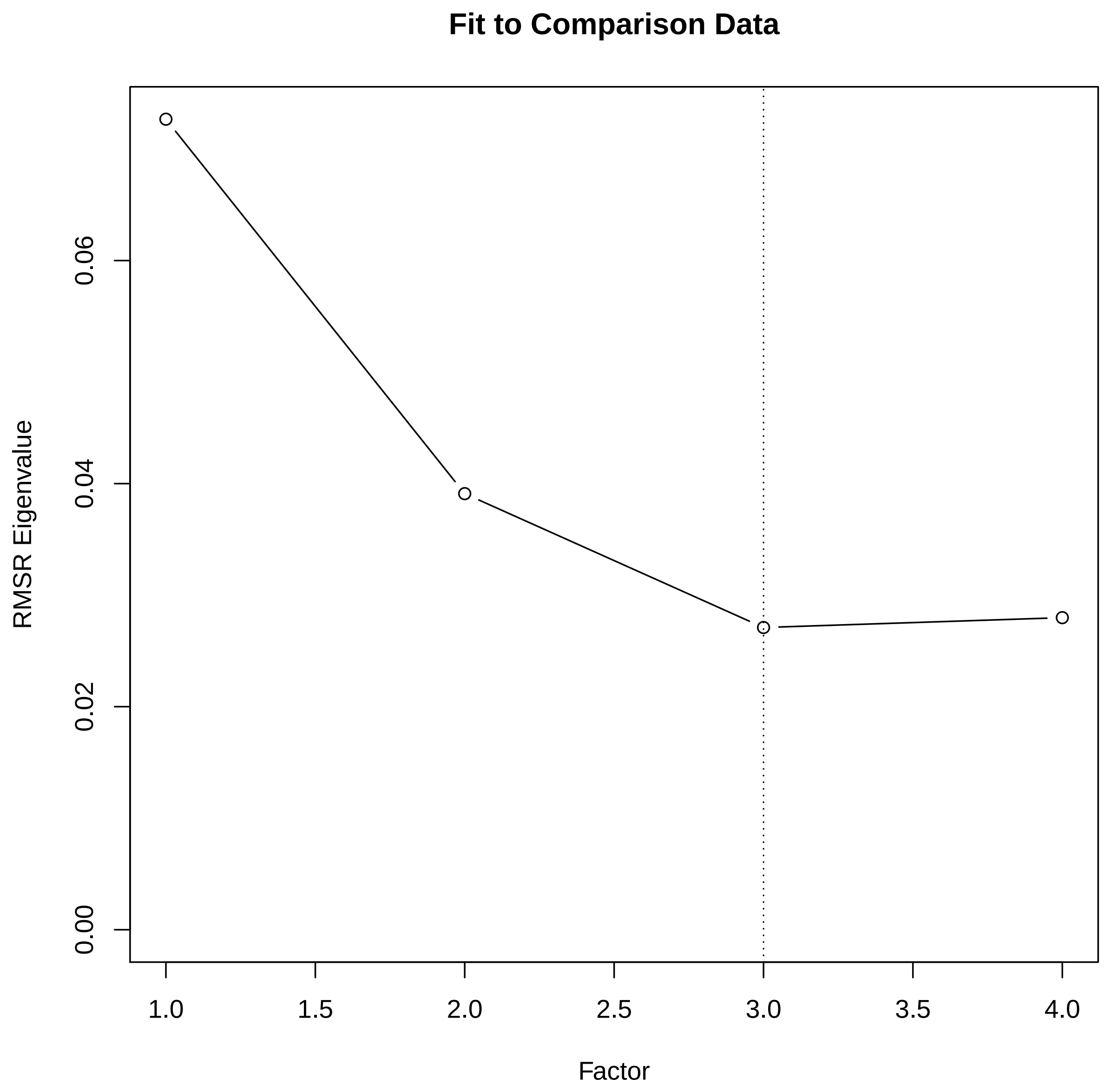


```
#   Run EFA with 3 factors
s.p.ea = efaUnrotate(data.s.o[,c(8,13,14,16,18:20,22)], 3, estimator="WLSMV")
s.p.eao = oblqRotate(s.p.ea, method="oblimin")
s.p.eao
```

```
## Standardized Rotated Factor Loadings
##                     factor1 factor2 factor3
## wealthy              0.933*                
## powerful             0.801*  0.143*        
## high.social.status   0.762*          0.178*
## successful           0.439*  0.253*  0.286*
```

```
## reputable                   0.755*  0.113*
## respected           0.178*  0.720*
## intelligent                 0.142*  0.849*
## educated            0.164*          0.833*
##
## Factor Correlation
##           factor1   factor2   factor3
## factor1 1.0000000 0.7046726 0.8267031
## factor2 0.7046726 1.0000000 0.7762098
## factor3 0.8267031 0.7762098 1.0000000
##
## Method of rotation:  Oblimin Quartimin
## [1] "The standard errors are close but do not match with other packages. Be mindful when usin
```

```
inspect(s.p.ea, "rsquare")    # Communalities (r^2)
```

```
##           educated high.social.status        intelligent
##              0.868              0.865              0.839
##           powerful          reputable          respected
##              0.698              0.657              0.716
##         successful            wealthy
##              0.814              0.864
```

```
# summary(s.p.eao)   # Uncomment to view test statistics (se, p, ci)
# fitMeasures(s.p.ea, c("chisq","df","pvalue","cfi","tli","rmsea","srmr"))
#      Uncomment to view listed fit measures
```

Successful is a good general predictor of prestige, but does not partition well into one factor. Given our criteria, we eliminate it due to the small gap between its primary loading and cross-loadings.

```
#   Remove 'successful' and repeat
EFA.Comp.Data(data.s[,c(8,13,14,16,18,19,22)], F.Max=5, Graph=T, Spearman=T)
```

```
## Number of factors to retain:  5
```

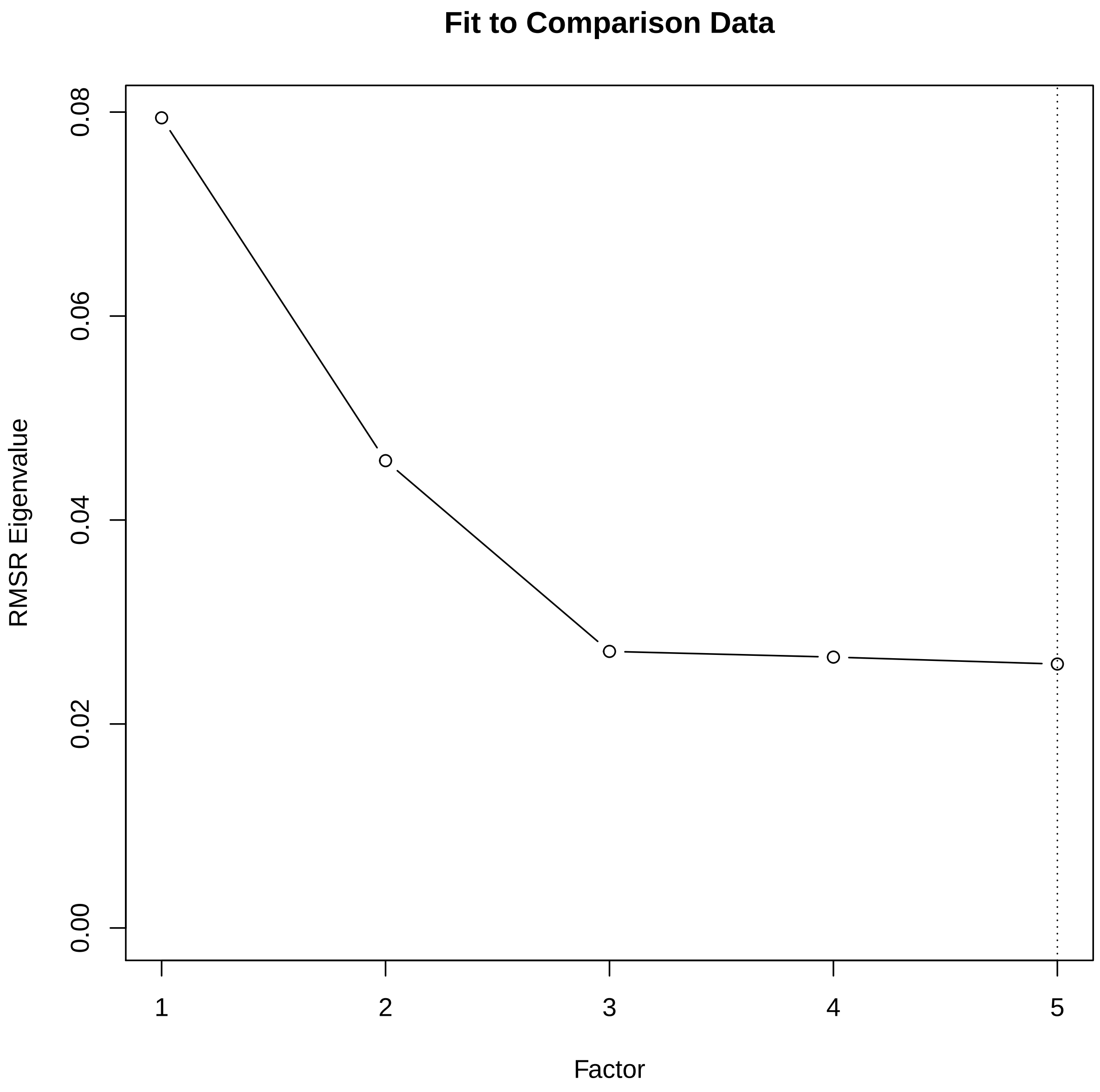

Likely overdimensionalization. 5- and 4-factor solutions fail.

```
#   Run EFA with 3 factors
s.p.ea2 = efaUnrotate(data.s.o[,c(8,13,14,16,18,19,22)], 3, estimator="WLSMV")
s.p.eao2 = oblqRotate(s.p.ea2, method="oblimin")
summary(s.p.eao2)   # Test statistics (se, p, ci)
```

```
## Standardized Rotated Factor Loadings
##                     factor1 factor2 factor3
## wealthy              0.935*
## powerful             0.819*  0.112*
## high.social.status   0.771*          0.166*
## reputable                    0.824*
```

```
## respected           0.238*  0.590*
## educated            0.108           0.893*
## intelligent                 0.146*  0.845*
##
## Factor Correlation
##           factor1   factor2   factor3
## factor1 1.0000000 0.6688528 0.8418781
## factor2 0.6688528 1.0000000 0.7507437
## factor3 0.8418781 0.7507437 1.0000000
##
## Method of rotation:  Oblimin Quartimin
##
## Test Statistics for Standardized Rotated Factor Loadings
##        lhs op               rhs std.loading    se      z     p ci.lower
## 1  factor1 =~          educated       0.108 0.086  1.253 0.210   -0.061
## 2  factor1 =~ high.social.status       0.771 0.032 24.059 0.000    0.708
## 3  factor1 =~       intelligent      -0.061 0.041 -1.510 0.131   -0.141
## 4  factor1 =~          powerful       0.819 0.032 25.929 0.000    0.757
## 5  factor1 =~         reputable      -0.010 0.021 -0.456 0.648   -0.051
## 6  factor1 =~         respected       0.238 0.050  4.807 0.000    0.141
## 7  factor1 =~           wealthy       0.935 0.027 34.625 0.000    0.882
## 8  factor2 =~          educated      -0.064 0.014 -4.473 0.000   -0.092
## 9  factor2 =~ high.social.status       0.027 0.022  1.223 0.221   -0.016
## 10 factor2 =~       intelligent       0.146 0.063  2.322 0.020    0.023
## 11 factor2 =~          powerful       0.112 0.034  3.307 0.001    0.045
## 12 factor2 =~         reputable       0.824 0.027 30.649 0.000    0.771
## 13 factor2 =~         respected       0.590 0.077  7.703 0.000    0.440
## 14 factor2 =~           wealthy      -0.035 0.020 -1.729 0.084   -0.076
## 15 factor3 =~          educated       0.893 0.081 11.090 0.000    0.735
## 16 factor3 =~ high.social.status       0.166 0.034  4.818 0.000    0.098
## 17 factor3 =~       intelligent       0.845 0.083 10.166 0.000    0.682
## 18 factor3 =~          powerful      -0.079 0.029 -2.754 0.006   -0.136
## 19 factor3 =~         reputable       0.048 0.034  1.392 0.164   -0.020
## 20 factor3 =~         respected       0.056 0.063  0.883 0.377   -0.068
## 21 factor3 =~           wealthy       0.019 0.030  0.641 0.521   -0.040
##    ci.upper
## 1     0.276
## 2     0.834
## 3     0.018
## 4     0.880
## 5     0.032
## 6     0.335
## 7     0.988
## 8    -0.036
## 9     0.070
## 10    0.270
## 11    0.178
## 12    0.877
```

```
## 13    0.741
## 14    0.005
## 15    1.051
## 16    0.233
## 17    1.008
## 18   -0.023
## 19    0.116
## 20    0.179
## 21    0.079
```

```
inspect(s.p.ea2, "rsquare")    # Communalities (r^2)
```

```
##           educated high.social.status        intelligent
##              0.880              0.872              0.826
##           powerful          reputable          respected
##              0.688              0.729              0.668
##            wealthy
##              0.862
```

```
fitMeasures(s.p.ea2, c("chisq","df","pvalue",
                       "cfi","tli","rmsea","srmr"))   #       Fit measures
```

```
##  chisq     df pvalue    cfi    tli  rmsea   srmr
##  0.553  3.000  0.907  1.000  1.000  0.000  0.002
```

```
#   Prepare factor loadings plot
s.p.3d = scatterplot3d(s.p.eao2@loading, lab=c(6,6), lab.z=6,
                       xlim=c(-.2,1), ylim=c(-.2,1), zlim=c(-.2,1))
env = environment(s.p.3d[[1]])
xmin = get("x.min", env=env) * get("x.scal", env=env)
xmax = get("x.max", env=env) * get("x.scal", env=env)
xmean = mean(c(xmin, xmax))
xnew = 2*xmean - s.p.eao2@loading[,1]
```

```
#   Plot factor loadings
# pdf("figure1.pdf", width=8, height=8)

s.p.3d = scatterplot3d(xnew, s.p.eao2@loading[,2], s.p.eao2@loading[,3],
                       xlim=c(-.2,1), ylim=c(-.2,1), zlim=c(-.2,1),
                       pch=16, type="h", box=F, asp=1, mar=c(5,3,0,3)+0.1,
                       color=c("#CC6677","#4477AA","#CC6677",
                               "#4477AA","#DDCC77","#DDCC77","#4477AA"),
                       xlab="Factor 1", ylab="Factor 2", zlab="Factor 3",
                       angle=130, lab=c(6,6), lab.z=6,
                       x.ticklabs=c("1.0","0.8","0.6","0.4","0.2","0.0","-0.2"))
s.p.3d.coords = s.p.3d$xyz.convert(xnew, s.p.eao2@loading[,2], s.p.eao2@loading[,3])
rownames(s.p.eao2@loading)[2] = c("high social status")
text(s.p.3d.coords$x + c(-0.55, rep(0,2), 0.5, rep(0,2), -0.1),
     s.p.3d.coords$y + c(0.05, rep(0.2,2), 0.05, rep(0.2,2), 0.225),
     rownames(s.p.eao2@loading), cex=0.75, asp=1)
p.p.text.loc = list(x = mean(s.p.3d.coords$x[c(2,4,7)]),
```

```
                  y = 6)
p.r.text.loc = list(x = mean(s.p.3d.coords$x[c(5,6)]),
                  y = 5.97)
p.i.text.loc = list(x = mean(s.p.3d.coords$x[c(1,3)]),
                  y = 6)
text(p.p.text.loc$x, p.p.text.loc$y, "Position", cex=1, asp=1)
text(p.r.text.loc$x, p.r.text.loc$y, "Reputation", cex=1, asp=1)
text(p.i.text.loc$x, p.i.text.loc$y, "Information", cex=1, asp=1)
```

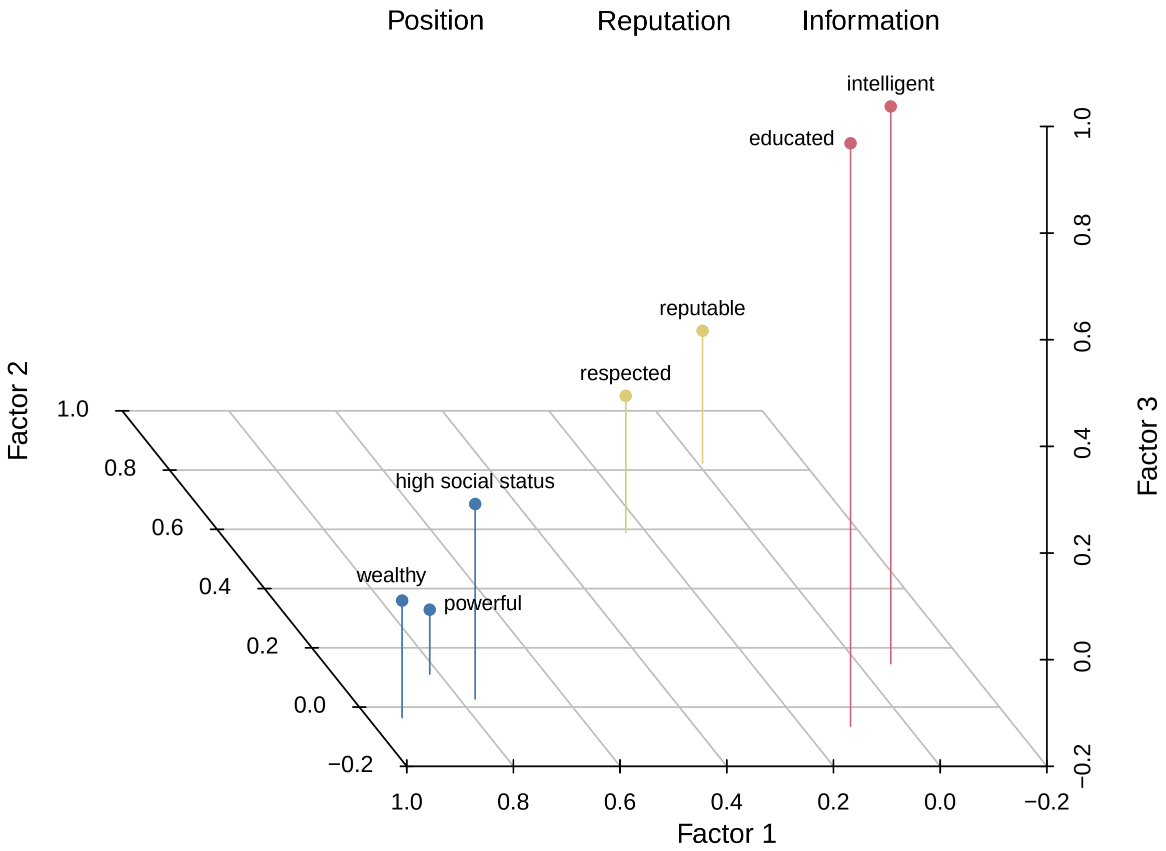


```
# dev.off()
```

Position-Reputation-Information Prestige Factor Structure

- **Position**

  - *wealthy*
  - *powerful*
  - *high social status*
- **Reputation**
  - *reputable*
  - *respected*
- **Information**
  - *educated*
  - *intelligent*

Triad Data

Checking Assumptions

Note: The concept of normality does not apply to dichotomous variables. The tetrachoric correlation assumes that the latent continuous versions of the dichotomous variables are bivariate-normally distributed (Hershberger 2005).

```
#   Check for linear relationships and correlations
corrplot.mixed(triad.cor, lower="ellipse", upper="number",
               order="hclust", hclust.method="ward.D",
               tl.cex=0.7, tl.pos="d", tl.col="black")
```

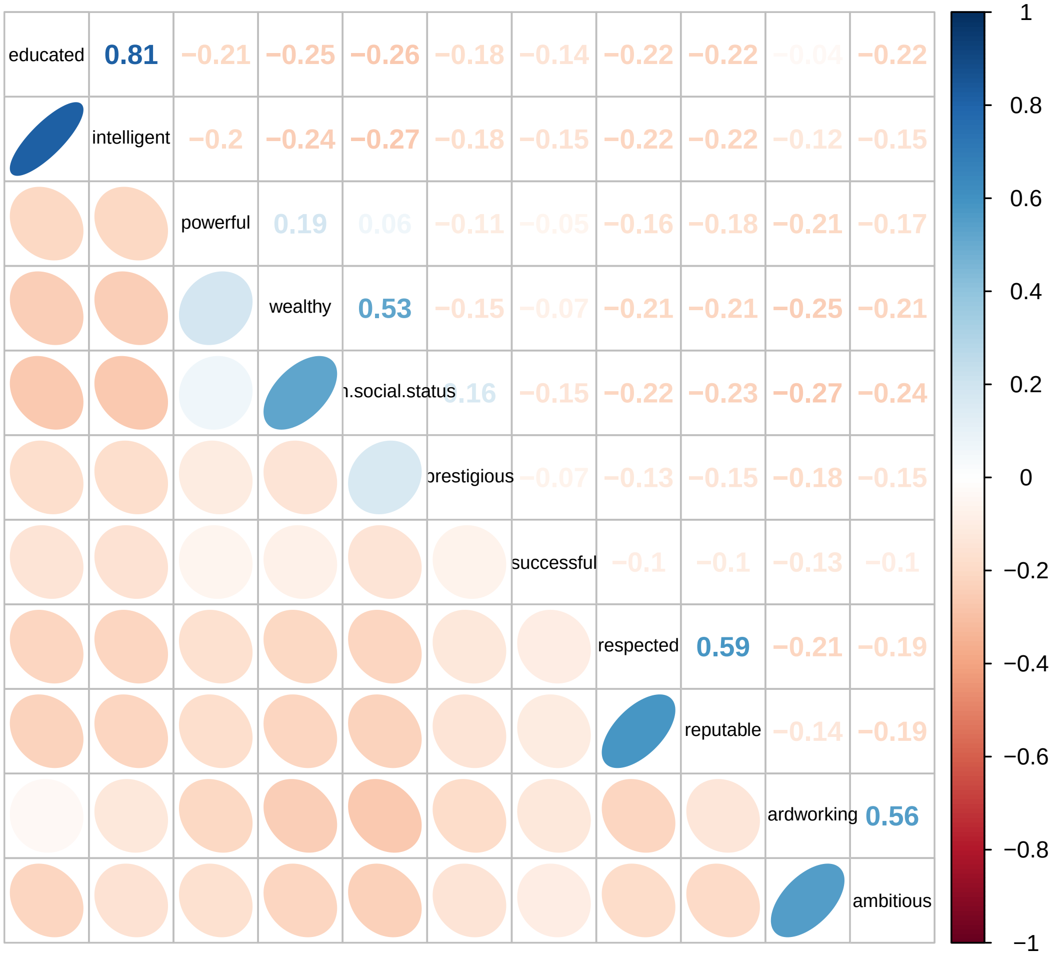


```
#   Check factorability
KMO(triad.cor[-1,-1])
```

```
## Kaiser-Meyer-Olkin factor adequacy
## Call: KMO(r = triad.cor[-1, -1])
## Overall MSA =  0.36
## MSA for each item =
##           wealthy high.social.status           powerful
##              0.59               0.31               0.16
##         respected           educated        hardworking
##              0.38               0.53               0.38
##        successful        intelligent          reputable
##              0.08               0.51               0.40
```

```
##          ambitious
##               0.34
```

```
cortest.bartlett(triad.cor[-1,-1], length(unique(data.t$id)))
```

```
## $chisq
## [1] 1255.215
##
## $p.value
## [1] 1.666607e-233
##
## $df
## [1] 45
```

```
#   Sample size
length(unique(data.t$id))
```

```
## [1] 308
```

```
#       Minimum
ncol(data.t[4:13]) * (ncol(data.t[4:13]) - 1) / 2
```

```
## [1] 45
```

```
#       Subjects-to-variables ratio
length(unique(data.t$id)) / ncol(data.t[4:13])
```

```
## [1] 30.8
```

Exploratory Factor Analysis (Prestige Domain Only)

```
#   Determine number of factors
fa.parallel(triad.cor[-1,-1], n.obs=308, fm="wls", fa="fa")
```

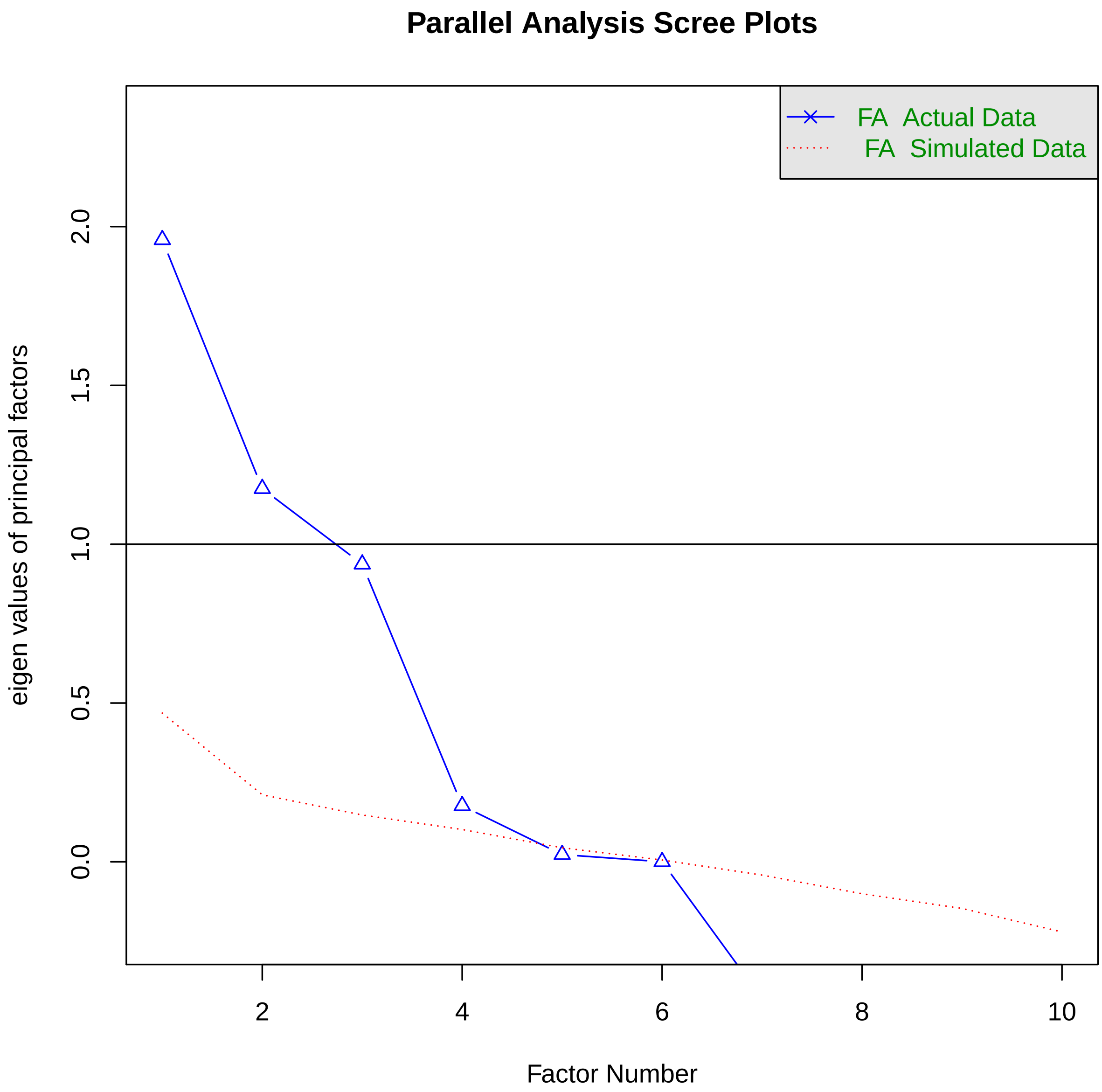


```
## Parallel analysis suggests that the number of factors =  4  and the number of components =  N
```

```
#   Run EFA with 4 factors
t.fa = fa(triad.cor[-1,-1], nfactors=4, n.obs=308, fm="wls", rotate="oblimin")
print.psych(t.fa, sort=T, cut=0.1, digits=3)
```

```
## Factor Analysis using method =  wls
## Call: fa(r = triad.cor[-1, -1], nfactors = 4, n.obs = 308, rotate = "oblimin",
##     fm = "wls")
## Standardized loadings (pattern matrix) based upon correlation matrix
##                      item   WLS1   WLS2   WLS3   WLS4    h2     u2  com
## educated                5  0.958                      0.932 0.0678 1.01
## intelligent             8  0.940                      0.901 0.0988 1.01
```

```
## respected             4 -0.107  0.767 -0.116        0.642 0.3580 1.09
## reputable             9 -0.105  0.764               0.622 0.3777 1.06
## powerful              3 -0.221 -0.244 -0.243        0.143 0.8567 3.01
## ambitious            10 -0.156 -0.120  0.774        0.654 0.3455 1.13
## hardworking           6                0.757        0.585 0.4152 1.02
## high.social.status    2 -0.204 -0.220 -0.226  0.569 0.595 0.4048 1.94
## successful            7 -0.248 -0.265 -0.263 -0.524 0.296 0.7042 2.54
## wealthy               1 -0.236 -0.276 -0.269  0.423 0.493 0.5068 3.18
##
##                        WLS1  WLS2  WLS3  WLS4
## SS loadings           2.071 1.476 1.474 0.844
## Proportion Var        0.207 0.148 0.147 0.084
## Cumulative Var        0.207 0.355 0.502 0.586
## Proportion Explained  0.353 0.252 0.251 0.144
## Cumulative Proportion 0.353 0.605 0.856 1.000
##
##  With factor correlations of
##        WLS1   WLS2   WLS3   WLS4
## WLS1  1.000 -0.088 -0.008 -0.162
## WLS2 -0.088  1.000 -0.055 -0.205
## WLS3 -0.008 -0.055  1.000 -0.217
## WLS4 -0.162 -0.205 -0.217  1.000
##
## Mean item complexity =  1.7
## Test of the hypothesis that 4 factors are sufficient.
##
## The degrees of freedom for the null model are  45  and the objective function was  4.145 with
## The degrees of freedom for the model are 11  and the objective function was  1.209
##
## The root mean square of the residuals (RMSR) is  0.048
## The df corrected root mean square of the residuals is  0.097
##
## The harmonic number of observations is  308 with the empirical chi square  64.095  with prob
## The total number of observations was  308  with Likelihood Chi Square =  362.969  with prob <
##
## Tucker Lewis Index of factoring reliability =  -0.2007
## RMSEA index =  0.3266  and the 90 % confidence intervals are  0.2948 0.3518
## BIC =  299.938
## Fit based upon off diagonal values = 0.966
```

```
#   Remove 'successful' and repeat
fa.parallel(triad.cor[c(2:7,9:11),c(2:7,9:11)], n.obs=308, fm="wls", fa="fa")
```

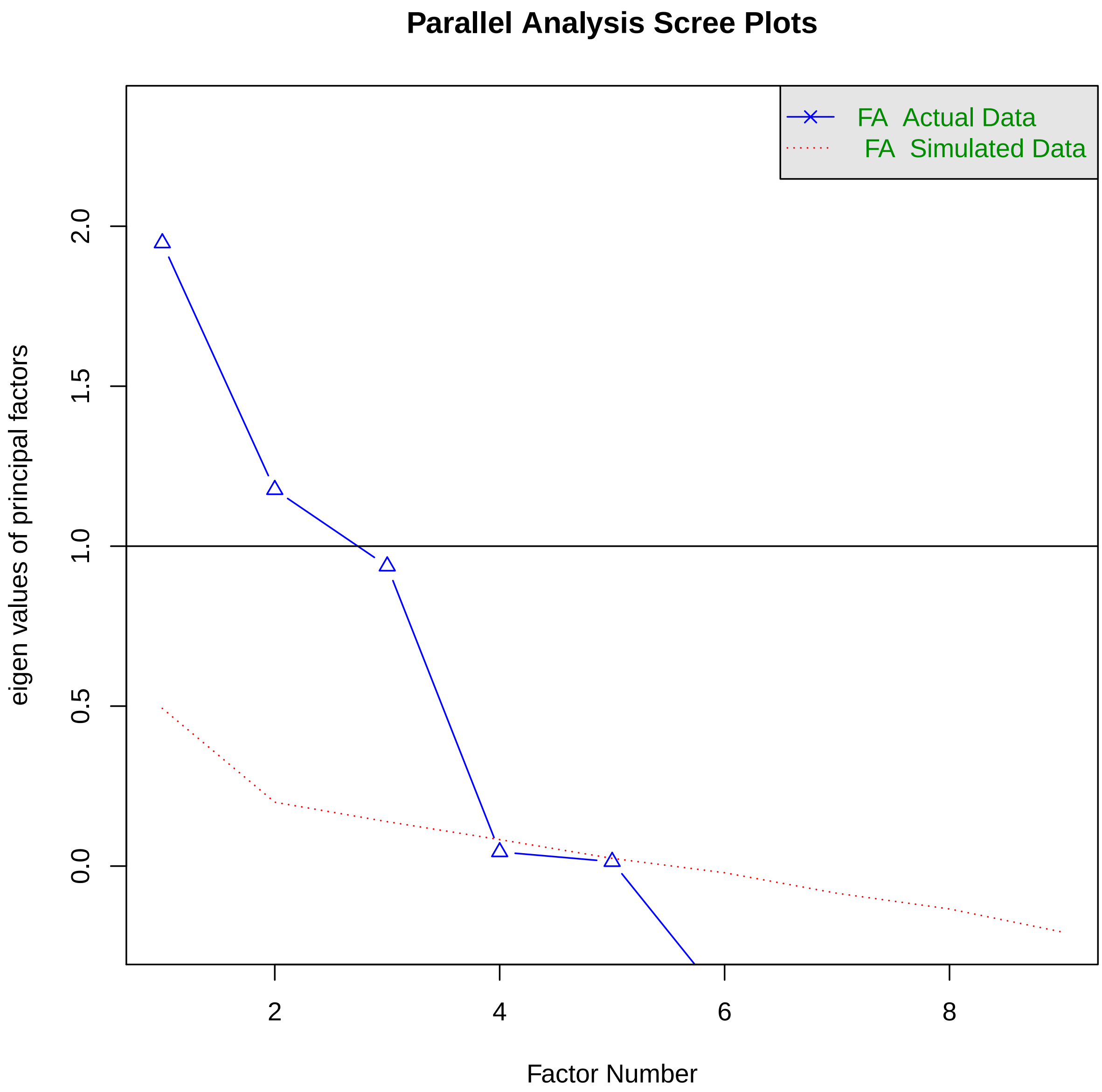


```
## Parallel analysis suggests that the number of factors =  3  and the number of components =  N
```

Likely underdimensionalization. 3-factor solution results in mostly negative loadings.

```
#   Run EFA with 4 factors
t.fa2 = fa(triad.cor[c(2:7,9:11),c(2:7,9:11)], nfactors=4, n.obs=308, fm="wls",
           rotate="oblimin")
print.psych(t.fa2, sort=T, cut=0.1, digits=3)
```

```
## Factor Analysis using method =  wls
## Call: fa(r = triad.cor[c(2:7, 9:11), c(2:7, 9:11)], nfactors = 4, n.obs = 308,
##     rotate = "oblimin", fm = "wls")
## Standardized loadings (pattern matrix) based upon correlation matrix
##                    item   WLS1   WLS3   WLS2   WLS4    h2      u2   com
```

```
## educated              5  0.924                         0.859 0.14053 1.03
## intelligent           7  0.884                         0.786 0.21374 1.03
## ambitious             9 -0.222 -0.798                  0.665 0.33488 1.19
## hardworking           6        -0.704        -0.123 0.518 0.48164 1.12
## high.social.status    2 -0.271  0.307 -0.722 -0.130 0.687 0.31332 1.74
## reputable             8 -0.284  0.334  0.587 -0.159 0.601 0.39893 2.28
## respected             4 -0.278  0.372  0.587 -0.146 0.619 0.38088 2.34
## wealthy               1 -0.228  0.249 -0.571        0.470 0.53016 1.73
## powerful              3                       0.989 0.999 0.00125 1.03
##
##                        WLS1  WLS3  WLS2  WLS4
## SS loadings           1.983 1.556 1.563 1.102
## Proportion Var        0.220 0.173 0.174 0.122
## Cumulative Var        0.220 0.393 0.567 0.689
## Proportion Explained  0.320 0.251 0.252 0.178
## Cumulative Proportion 0.320 0.570 0.822 1.000
##
##  With factor correlations of
##        WLS1   WLS3   WLS2   WLS4
## WLS1  1.000 -0.100  0.006 -0.062
## WLS3 -0.100  1.000 -0.008  0.031
## WLS2  0.006 -0.008  1.000 -0.184
## WLS4 -0.062  0.031 -0.184  1.000
##
## Mean item complexity =  1.5
## Test of the hypothesis that 4 factors are sufficient.
##
## The degrees of freedom for the null model are  36  and the objective function was  3.505 with
## The degrees of freedom for the model are 6  and the objective function was  0.121
##
## The root mean square of the residuals (RMSR) is  0.018
## The df corrected root mean square of the residuals is  0.044
##
## The harmonic number of observations is  308 with the empirical chi square  7.126  with prob <
## The total number of observations was  308  with Likelihood Chi Square =  36.352  with prob <
##
## Tucker Lewis Index of factoring reliability =  0.821
## RMSEA index =  0.13  and the 90 % confidence intervals are  0.0901 0.17
## BIC =  1.972
## Fit based upon off diagonal values = 0.996
## Measures of factor score adequacy
##                                                   WLS1  WLS3  WLS2  WLS4
## Correlation of scores with factors              0.956 0.897 0.897 0.999
## Multiple R square of scores with factors        0.913 0.804 0.805 0.998
## Minimum correlation of possible factor scores   0.826 0.608 0.611 0.997
```

```
#   Remove 'hardworking' and 'ambitious' and repeat
#       (Sequential removal in either order has same result)
```

```r
fa.parallel(triad.cor[c(2:6,9,10),c(2:6,9,10)], n.obs=308, fm="wls", fa="fa")
```

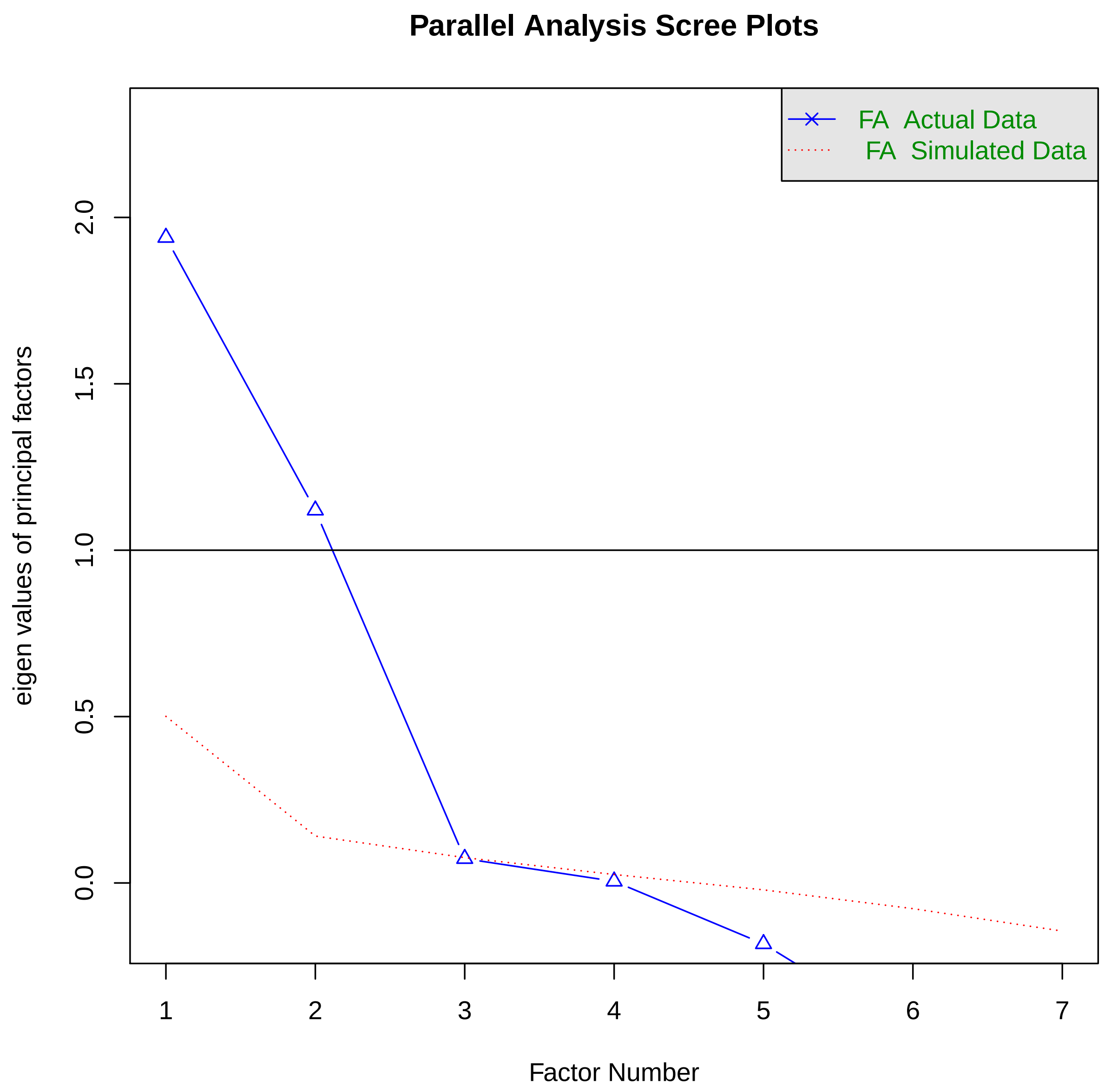


```
## Parallel analysis suggests that the number of factors =  2  and the number of components =  N
```

Likely underdimensionalization. 2-factor solution results in mostly negative loadings.

```r
#   Run EFA with 3 factors
t.fa3 = fa(triad.cor[c(2:6,9,10),c(2:6,9,10)], nfactors=3, n.obs=308, fm="wls",
           rotate="oblimin")
print.psych(t.fa3, sort=T, cut=0.1, digits=3)
```

```
## Factor Analysis using method =  wls
## Call: fa(r = triad.cor[c(2:6, 9, 10), c(2:6, 9, 10)], nfactors = 3,
##     n.obs = 308, rotate = "oblimin", fm = "wls")
```

```
##
##  Warning: A Heywood case was detected.
## Standardized loadings (pattern matrix) based upon correlation matrix
##                    item   WLS1   WLS2   WLS3    h2       u2  com
## educated              5  0.902               0.829 0.171062 1.00
## intelligent           6  0.878               0.795 0.204617 1.00
## reputable             7         0.765        0.614 0.385965 1.01
## respected             4         0.723        0.556 0.443804 1.02
## powerful              3 -0.374 -0.381 -0.142 0.176 0.824155 2.27
## high.social.status    2                1.001 1.000 0.000193 1.00
## wealthy               1 -0.236 -0.248  0.383 0.344 0.656219 2.45
##
##                        WLS1  WLS2  WLS3
## SS loadings           1.786 1.321 1.206
## Proportion Var        0.255 0.189 0.172
## Cumulative Var        0.255 0.444 0.616
## Proportion Explained  0.414 0.306 0.280
## Cumulative Proportion 0.414 0.720 1.000
##
##  With factor correlations of
##        WLS1   WLS2  WLS3
## WLS1  1.000 -0.236 -0.29
## WLS2 -0.236  1.000 -0.29
## WLS3 -0.290 -0.290  1.00
##
## Mean item complexity =  1.4
## Test of the hypothesis that 3 factors are sufficient.
##
## The degrees of freedom for the null model are  21  and the objective function was  2.314 with
## The degrees of freedom for the model are 3  and the objective function was  0.007
##
## The root mean square of the residuals (RMSR) is  0.011
## The df corrected root mean square of the residuals is  0.029
##
## The harmonic number of observations is  308 with the empirical chi square  1.571  with prob <
## The total number of observations was  308  with Likelihood Chi Square =  2.258  with prob <
##
## Tucker Lewis Index of factoring reliability =  1.0077
## RMSEA index =  0  and the 90 % confidence intervals are  0 0.0865
## BIC =  -14.932
## Fit based upon off diagonal values = 0.999
```

```
#   Remove 'powerful' and repeat (due to Heywood case)
t.fa4 = fa(triad.cor[c(2,3,5,6,9,10),c(2,3,5,6,9,10)], nfactors=3, n.obs=308,
          fm="wls", rotate="oblimin")
print.psych(t.fa4, sort=T, cut=0.1, digits=3)
```

```
## Factor Analysis using method =  wls
## Call: fa(r = triad.cor[c(2, 3, 5, 6, 9, 10), c(2, 3, 5, 6, 9, 10)],
```

```
##     nfactors = 3, n.obs = 308, rotate = "oblimin", fm = "wls")
## Standardized loadings (pattern matrix) based upon correlation matrix
##                   item   WLS1   WLS2   WLS3    h2    u2  com
## intelligent          5  0.962               0.891 0.109 1.00
## educated             4  0.832               0.749 0.251 1.01
## reputable            6         0.820        0.655 0.345 1.00
## respected            3         0.705        0.532 0.468 1.01
## wealthy              1                0.776 0.571 0.429 1.01
## high.social.status   2                0.657 0.497 0.503 1.04
##
##                        WLS1  WLS2  WLS3
## SS loadings           1.641 1.193 1.061
## Proportion Var        0.274 0.199 0.177
## Cumulative Var        0.274 0.472 0.649
## Proportion Explained  0.421 0.306 0.272
## Cumulative Proportion 0.421 0.728 1.000
##
##  With factor correlations of
##        WLS1   WLS2   WLS3
## WLS1  1.000 -0.303 -0.374
## WLS2 -0.303  1.000 -0.369
## WLS3 -0.374 -0.369  1.000
##
## Mean item complexity =  1
## Test of the hypothesis that 3 factors are sufficient.
##
## The degrees of freedom for the null model are  15  and the objective function was  2.174 with
## The degrees of freedom for the model are 0  and the objective function was  0
##
## The root mean square of the residuals (RMSR) is  0
## The df corrected root mean square of the residuals is  NA
##
## The harmonic number of observations is  308 with the empirical chi square  0  with prob <  NA
## The total number of observations was  308  with Likelihood Chi Square =  0.001  with prob <
##
## Tucker Lewis Index of factoring reliability =  -Inf
## Fit based upon off diagonal values = 1
## Measures of factor score adequacy
##                                                   WLS1  WLS2  WLS3
## Correlation of scores with factors             0.959 0.879 0.856
## Multiple R square of scores with factors       0.920 0.772 0.732
## Minimum correlation of possible factor scores  0.840 0.544 0.464
```

Cluster Analysis

Attitudinal Data

Checking Assumptions

```
#   Check clustering of Manhattan distance matrix
plot(cmdscale(dist(t(data.s.o[,c(2:4,6:22)]), "manhattan"), 2), type="n",
     xlab="Dimension 1", ylab="Dimension 2", main ="Multidimensional Scaling Plot")
text(cmdscale(dist(t(data.s.o[,c(2:4,6:22)]), "manhattan"), 2),
     labels=rownames(cmdscale(dist(t(data.s.o[,c(2:4,6:22)]), "manhattan"), 2)))
```

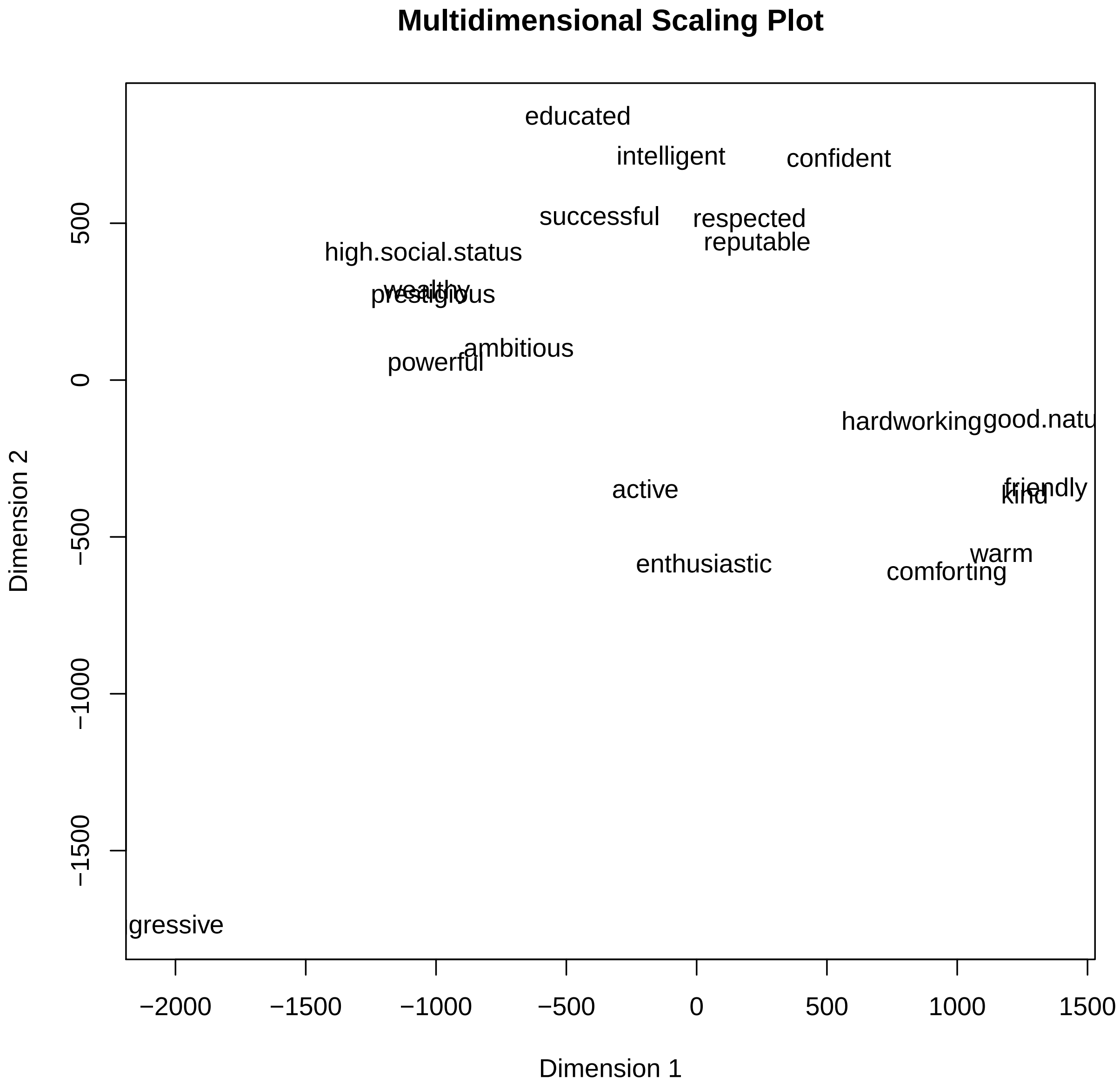

Partitioning Around Medoids

```
#   Check whether more than 1 cluster is needed
#       (Subsequent PAM analyses integrate this step)
dudahart2(t(data.s[,c(2:4,6:22)]),
          pam(t(data.s[,c(2:4,6:22)]), 2, metric="manhattan")$clustering)
```

```
## $p.value
## [1] 0
##
## $dh
## [1] 0.6916442
##
## $compare
## [1] 0.9713662
##
## $cluster1
## [1] FALSE
##
## $alpha
## [1] 0.001
##
## $z
## [1] 3.090232
```

```
#   Determine number of clusters
a.pamk = pamk(t(data.s[,c(2:4,6:22)]), 2:9, metric="manhattan")
a.pamk[2:3]
```

```
## $nc
## [1] 3
##
## $crit
## [1] 0.0000000 0.3023491 0.3165899 0.2225254 0.2442202 0.2194406 0.1852707
## [8] 0.1735111 0.1753493
```

```
#   Run PAM with 3 clusters
a.pam = pam(t(data.s[,c(2:4,6:22)]), 3, metric="manhattan")
summary(a.pam)[6:7]
```

```
## $clusinfo
##      size max_diss   av_diss diameter separation
## [1,]   13     1652 1112.2308     2131       1462
## [2,]    1        0    0.0000        0       2262
## [3,]    6     1359  816.1667     1646       1462
##
## $silinfo
## $silinfo$widths
##                       cluster neighbor   sil_width
## high.social.status          1        3 0.452726429
## wealthy                     1        2 0.430096559
```

```
## prestigious              1         3 0.419979886
## successful               1         3 0.397993172
## educated                 1         3 0.397326402
## ambitious                1         3 0.370609210
## powerful                 1         2 0.370210728
## intelligent              1         3 0.310737387
## active                   1         3 0.154800104
## respected                1         3 0.152838650
## reputable                1         3 0.105027300
## confident                1         3 0.017841768
## enthusiastic             1         3 0.008110642
## aggressive               2         1 0.000000000
## friendly                 3         1 0.541292135
## kind                     3         1 0.531422052
## warm                     3         1 0.510053840
## good.natured             3         1 0.505936238
## comforting               3         1 0.414480580
## hardworking              3         1 0.240314619
##
## $silinfo$clus.avg.widths
## [1] 0.2760229 0.0000000 0.4572499
##
## $silinfo$avg.width
## [1] 0.3165899
```

```
plot(a.pam, which.plots=2)
```

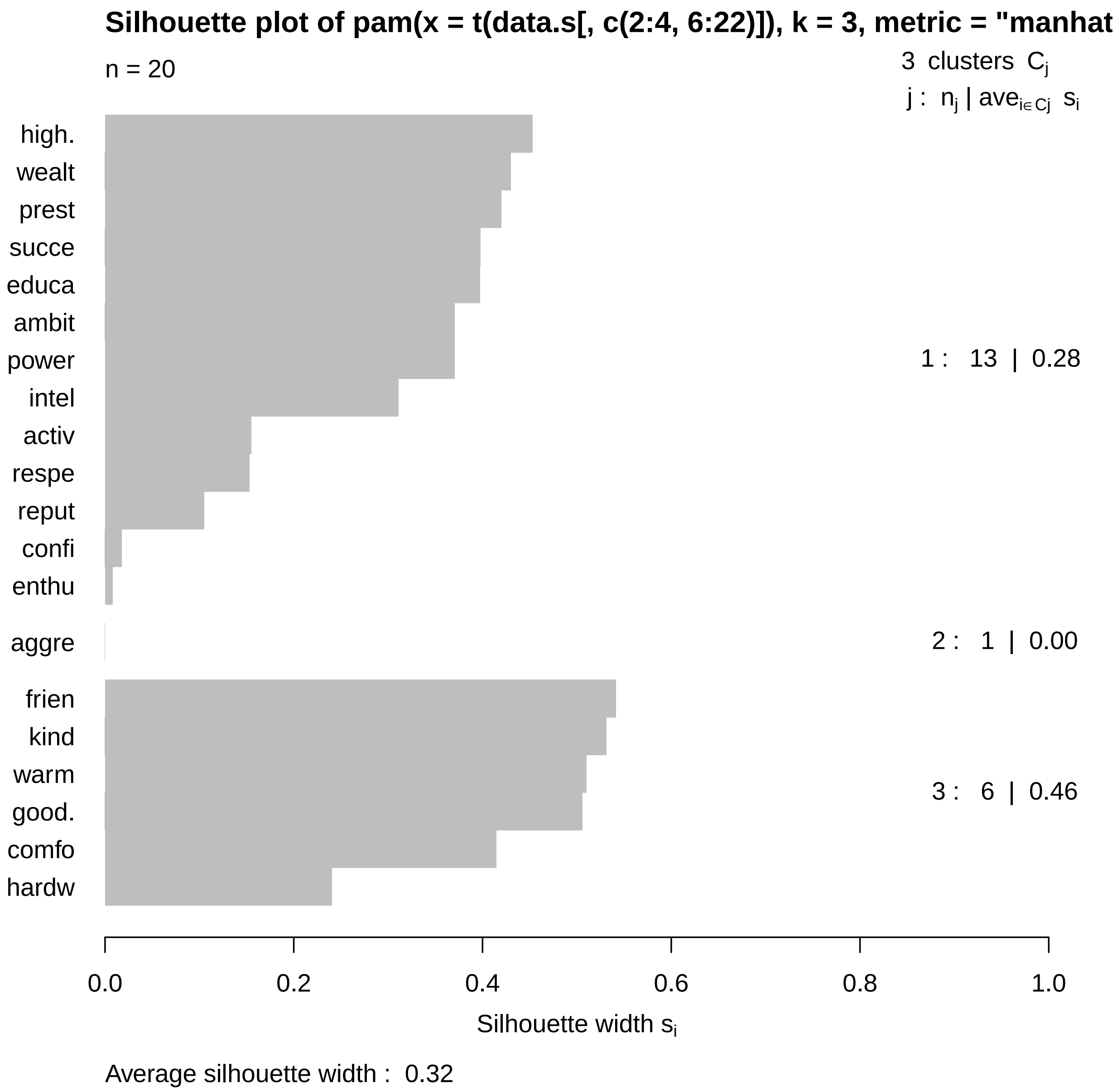


```
#   Remove 'aggressive' and repeat
a.pamk2 = pamk(t(data.s[,c(2,4,6:22)]), 1:10, metric="manhattan")
a.pamk2[2:3]
```

```
## $nc
## [1] 2
##
## $crit
##  [1] 0.0000000 0.3357046 0.2342372 0.2570739 0.2309902 0.1950218 0.1826433
##  [8] 0.1845782 0.1862534 0.1421999
```

```
#   Run PAM with 2 clusters
a.pam2 = pam(t(data.s[,c(2,4,6:22)]), 2, metric="manhattan")
```

```r
summary(a.pam2)[6:7]
```

```
## $clusinfo
##      size max_diss   av_diss diameter separation
## [1,]   13     1652 1112.2308     2131       1462
## [2,]    6     1359  816.1667     1646       1462
##
## $silinfo
## $silinfo$widths
##                     cluster neighbor   sil_width
## high.social.status        1        2 0.452726429
## wealthy                   1        2 0.436419878
## prestigious               1        2 0.419979886
## powerful                  1        2 0.410476585
## successful                1        2 0.397993172
## educated                  1        2 0.397326402
## ambitious                 1        2 0.370609210
## intelligent               1        2 0.310737387
## active                    1        2 0.154800104
## respected                 1        2 0.152838650
## reputable                 1        2 0.105027300
## confident                 1        2 0.017841768
## enthusiastic              1        2 0.008110642
## friendly                  2        1 0.541292135
## kind                      2        1 0.531422052
## warm                      2        1 0.510053840
## good.natured              2        1 0.505936238
## comforting                2        1 0.414480580
## hardworking               2        1 0.240314619
##
## $silinfo$clus.avg.widths
## [1] 0.2796067 0.4572499
##
## $silinfo$avg.width
## [1] 0.3357046
```

```r
plot(a.pam2, which.plots=2)
```

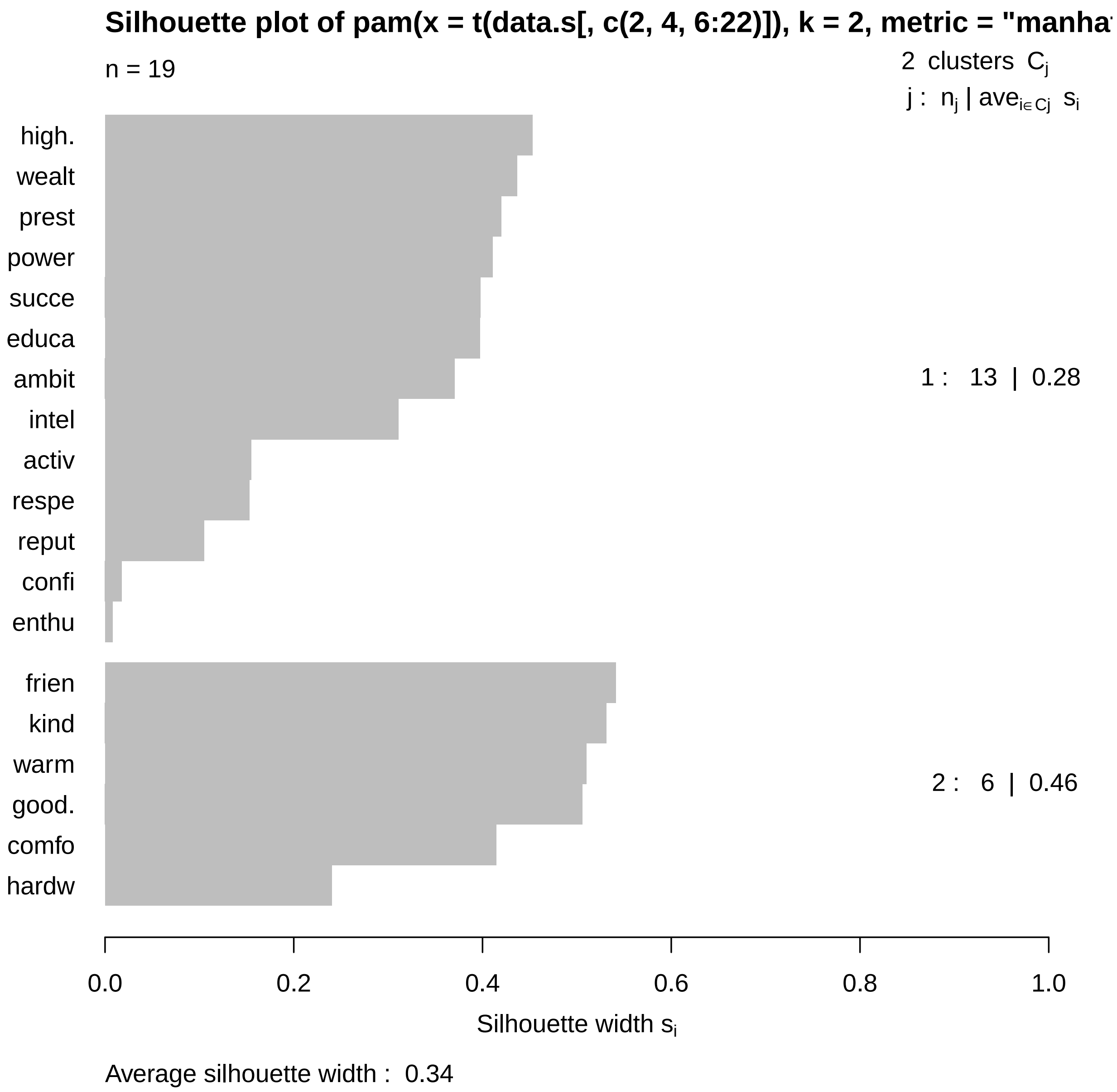


```
#   Remove 'hardworking' and repeat
a.pamk3 = pamk(t(data.s[,c(2,4,6:11,13:22)]), 1:10, metric="manhattan")
a.pamk3[2:3]

## $nc
## [1] 2
##
## $crit
##  [1] 0.0000000 0.3619950 0.2534559 0.2788504 0.2527778 0.2183691 0.2158282
##  [8] 0.2175965 0.1500999 0.1296384

#   Run PAM with 2 clusters
a.pam3 = pam(t(data.s[,c(2,4,6:11,13:22)]), 2, metric="manhattan")
```

```r
summary(a.pam3)[6:7]
```

```
## $clusinfo
##      size max_diss  av_diss diameter separation
## [1,]   13     1652 1112.231     2131       1599
## [2,]    5     1095  695.200     1265       1599
##
## $silinfo
## $silinfo$widths
##                     cluster neighbor    sil_width
## high.social.status        1        2  0.458346448
## wealthy                   1        2  0.443492696
## prestigious               1        2  0.423819800
## powerful                  1        2  0.422918523
## successful                1        2  0.409412476
## educated                  1        2  0.404080014
## ambitious                 1        2  0.388092288
## intelligent               1        2  0.320962480
## active                    1        2  0.176134442
## respected                 1        2  0.169215545
## reputable                 1        2  0.121942591
## confident                 1        2  0.036660661
## enthusiastic              1        2 -0.003223519
## friendly                  2        1  0.593164794
## kind                      2        1  0.576825409
## warm                      2        1  0.566833315
## good.natured              2        1  0.540302222
## comforting                2        1  0.466929939
##
## $silinfo$clus.avg.widths
## [1] 0.2901426 0.5488111
##
## $silinfo$avg.width
## [1] 0.361995
```

```r
plot(a.pam3, which.plots=2)
```

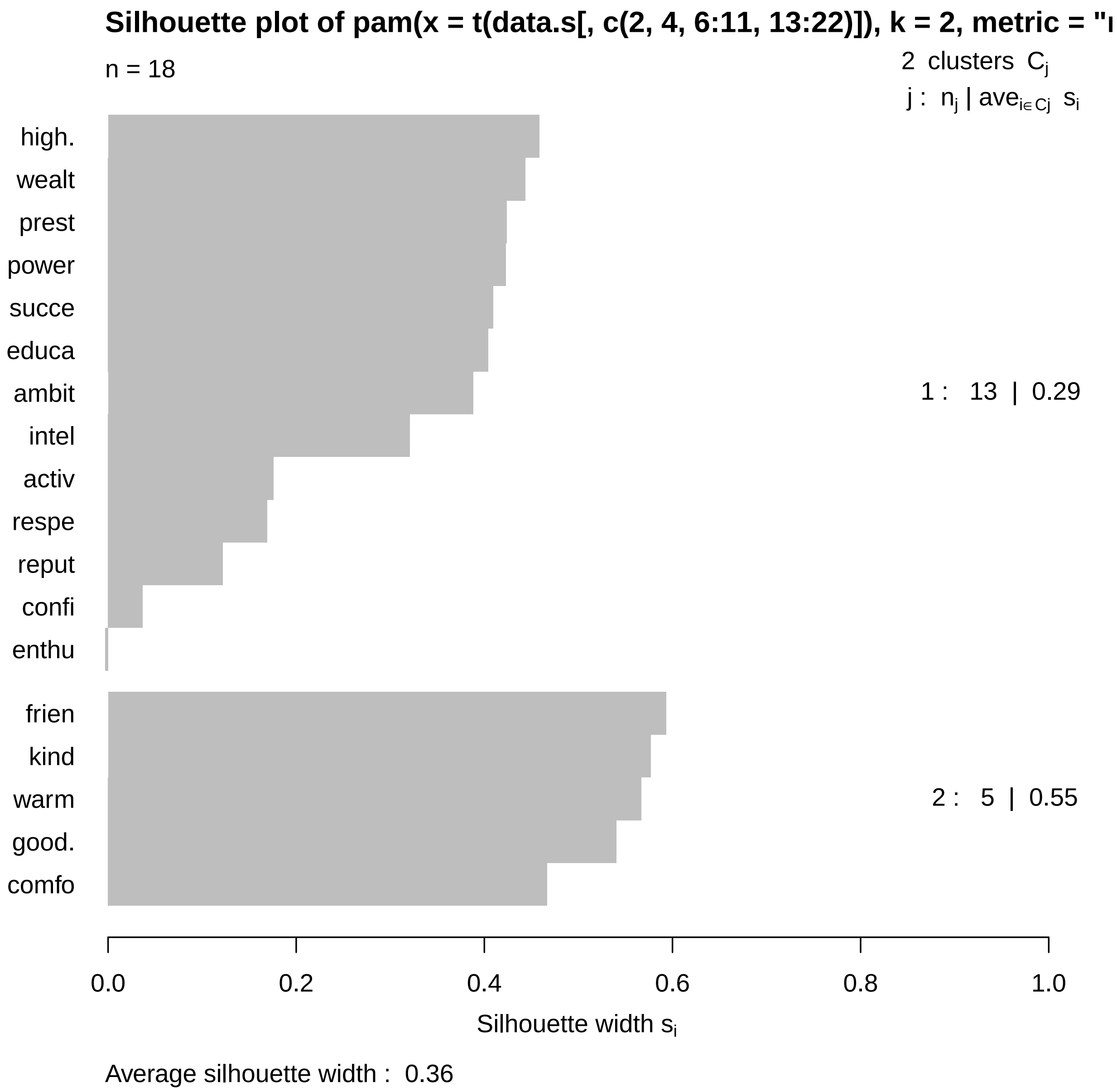


```
#   Remove 'enthusiastic' and repeat
a.pamk4 = pamk(t(data.s[,c(2,4,6:8,10,11,13:22)]), 1:10, metric="manhattan")
a.pamk4[2:3]
```

```
## $nc
## [1] 2
##
## $crit
##  [1] 0.0000000 0.3953822 0.2810903 0.2825911 0.2389058 0.2299334 0.2318057
##  [8] 0.1589293 0.1372642 0.1146208
```

```
#   Run PAM with 2 clusters
a.pam4 = pam(t(data.s[,c(2,4,6:8,10,11,13:22)]), 2, metric="manhattan")
```

```r
summary(a.pam4)[6:7]

## $clusinfo
##      size max_diss av_diss diameter separation
## [1,]   12     1491 1067.25     2131       1599
## [2,]    5     1095  695.20     1265       1599
##
## $silinfo
## $silinfo$widths
##                   cluster neighbor  sil_width
## high.social.status      1        2 0.47588491
## wealthy                 1        2 0.45994205
## prestigious             1        2 0.43882963
## powerful                1        2 0.43630177
## successful              1        2 0.42933402
## educated                1        2 0.42701157
## ambitious               1        2 0.39961216
## intelligent             1        2 0.34056614
## respected               1        2 0.18615809
## active                  1        2 0.16814216
## reputable               1        2 0.13859639
## confident               1        2 0.04622739
## friendly                2        1 0.59841337
## kind                    2        1 0.58207662
## warm                    2        1 0.57433618
## good.natured            2        1 0.54481798
## comforting              2        1 0.47524674
##
## $silinfo$clus.avg.widths
## [1] 0.3288839 0.5549782
##
## $silinfo$avg.width
## [1] 0.3953822

plot(a.pam4, which.plots=2)
```

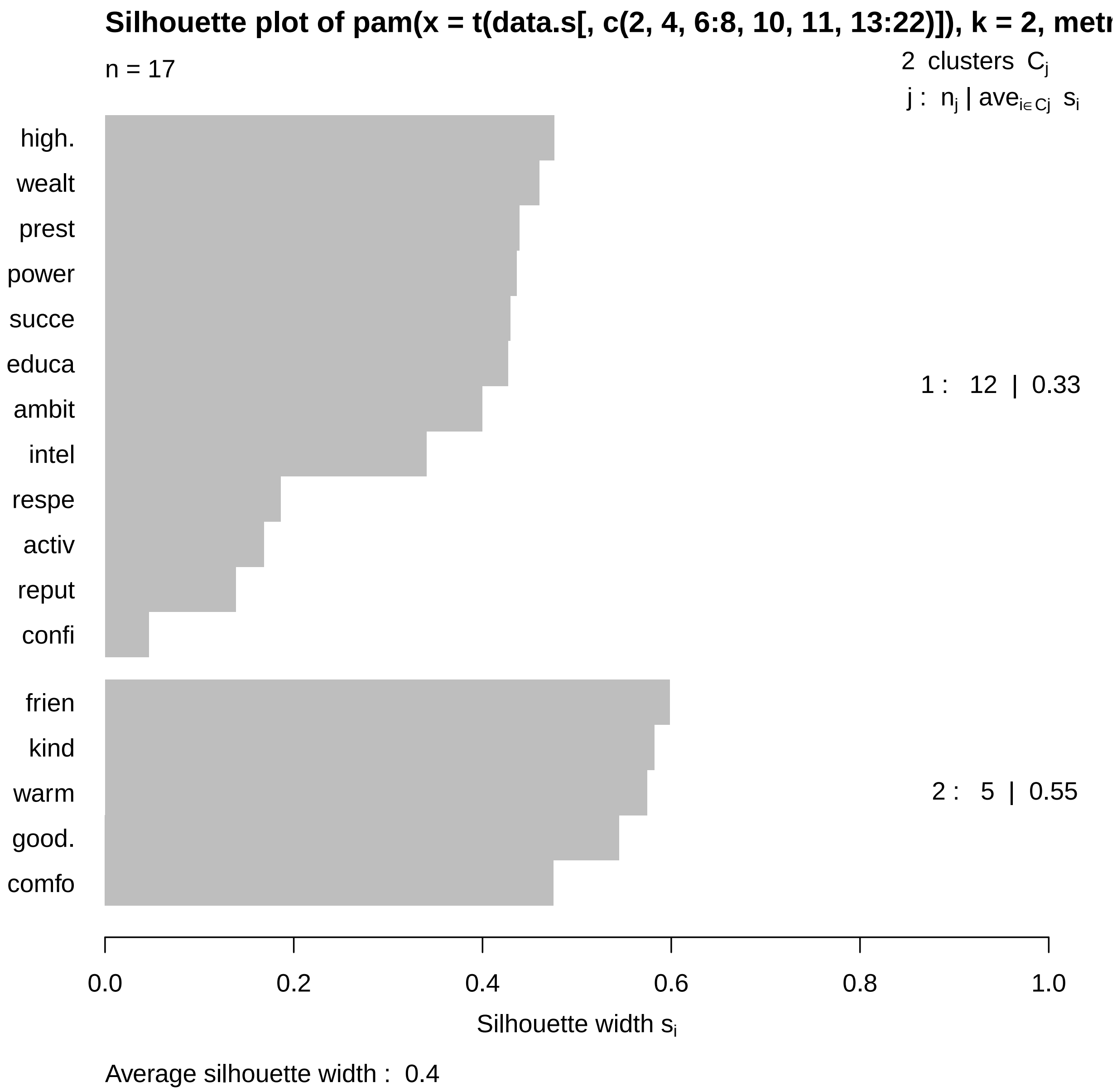


```
#   Remove 'confident' and repeat
a.pamk5 = pamk(t(data.s[,c(2,4,6,8,10,11,13:22)]), 1:10, metric="manhattan")
a.pamk5[2:3]
```

```
## $nc
## [1] 2
##
## $crit
##  [1] 0.0000000 0.4284709 0.2911180 0.2937192 0.2443043 0.2462936 0.1688624
##  [8] 0.1458432 0.1217846 0.1067901
```

As before, optimum average silhouette width is reached with 2 clusters, with 3-cluster and 4-cluster groupings of roughly equivalent secondary values.

```r
#   Run PAM with 2 clusters
a.pam5 = pam(t(data.s[,c(2,4,6,8,10,11,13:22)]), 2, metric="manhattan")
summary(a.pam5)[6:7]
```

```
## $clusinfo
##      size max_diss av_diss diameter separation
## [1,]   11     1491  1033.0     1794       1599
## [2,]    5     1095   695.2     1265       1599
##
## $silinfo
## $silinfo$widths
##                    cluster neighbor sil_width
## high.social.status       1        2 0.5032263
## wealthy                  1        2 0.4885259
## prestigious              1        2 0.4678708
## powerful                 1        2 0.4605444
## successful               1        2 0.4430448
## educated                 1        2 0.4367289
## ambitious                1        2 0.4189237
## intelligent              1        2 0.3425775
## respected                1        2 0.1777018
## active                   1        2 0.1723473
## reputable                1        2 0.1363857
## friendly                 2        1 0.6054891
## kind                     2        1 0.5885351
## warm                     2        1 0.5800038
## good.natured             2        1 0.5540971
## comforting               2        1 0.4795321
##
## $silinfo$clus.avg.widths
## [1] 0.3679888 0.5615314
##
## $silinfo$avg.width
## [1] 0.4284709
```

```r
plot(a.pam5, which.plots=2)
```

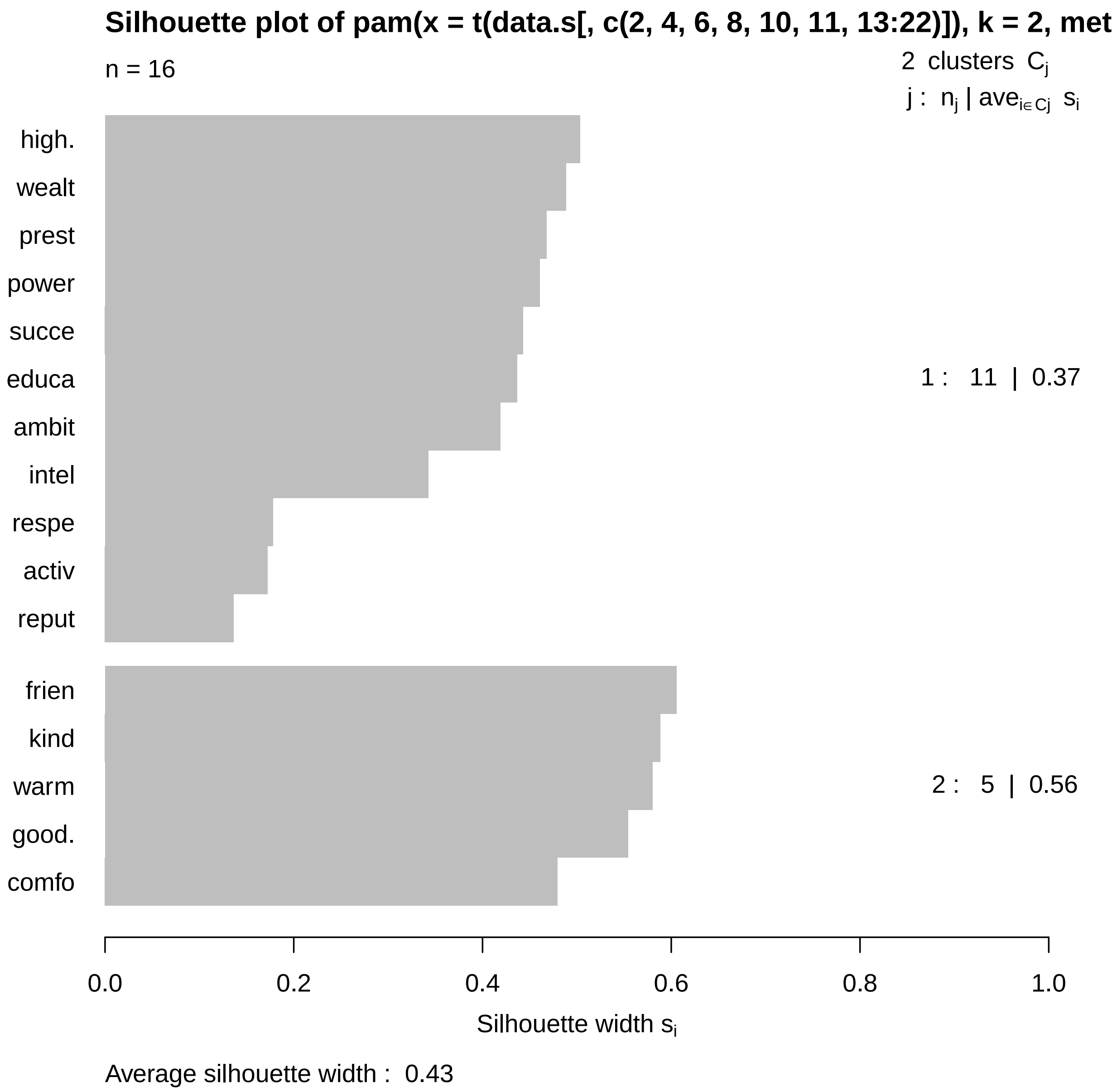


```
#   Plot color-coded silhouette plot
a.pam5.sil = silhouette(a.pam5)
rownames(a.pam5.sil)[1] = "high social\nstatus"
rownames(a.pam5.sil)[15] = "good-natured"
attr(a.pam5.sil, "Ordered") = T
attr(a.pam5.sil, "class") = "silhouette"

par(mar=.1+c(4,7,2,2))
plot(a.pam5.sil, main="", max.strlen=40, do.n.k=F,
     col=c("#4477AA","#4477AA","black","#4477AA","gray",
           "#CC6677","gray","#CC6677","#DDCC77","#332288","#DDCC77",
           "#117733","#117733","#117733","#117733","#117733"))
```

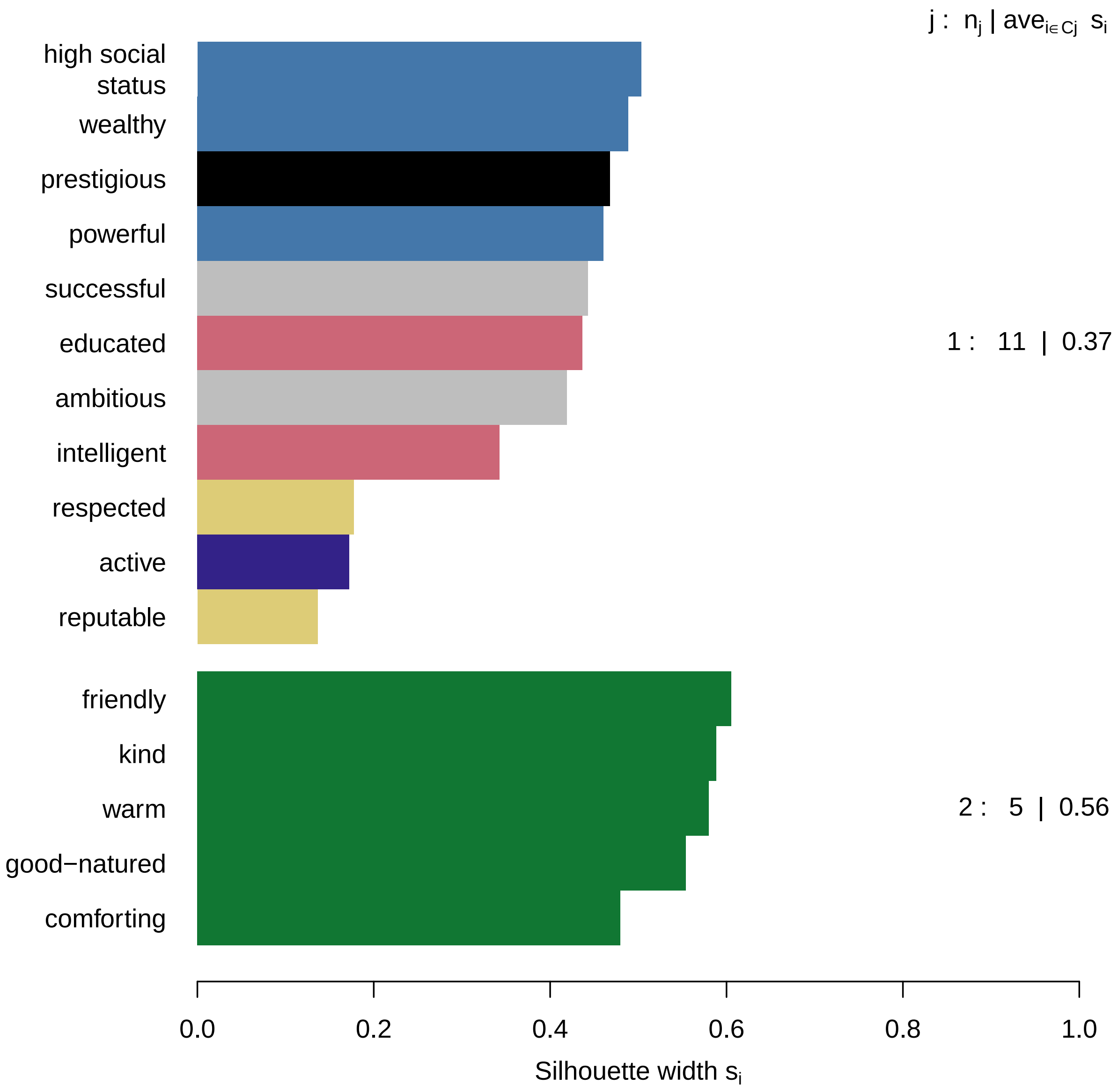


```
#   Plot color-coded multidimensional scaling plot
a.pam5.mds = cmdscale(as.dist(as.matrix(a.pam5$diss)))
a.pam5.mds = a.pam5.mds[c(7,16,11,10,14,4,2,8,13,1,12,5,9,15,6,3),]
rownames(a.pam5.mds)[1] = "high social status"
rownames(a.pam5.mds)[15] = "good-natured"
a.pam5.mds = scales::rescale(a.pam5.mds, c(-.75,.75))

par(pty="s")
plot(a.pam5.mds, type="n", asp=1, xlim=c(-1,1), ylim=c(-1,1),
     xlab="PC1", ylab="PC2")
text(a.pam5.mds[,1], a.pam5.mds[,2], labels=rownames(a.pam5.mds), cex=1,
     col=c("#4477AA","#4477AA","black","#4477AA","gray",
```

```
        "#CC6677","gray","#CC6677","#DDCC77","#332288","#DDCC77",
        "#117733","#117733","#117733","#117733","#117733"))
```

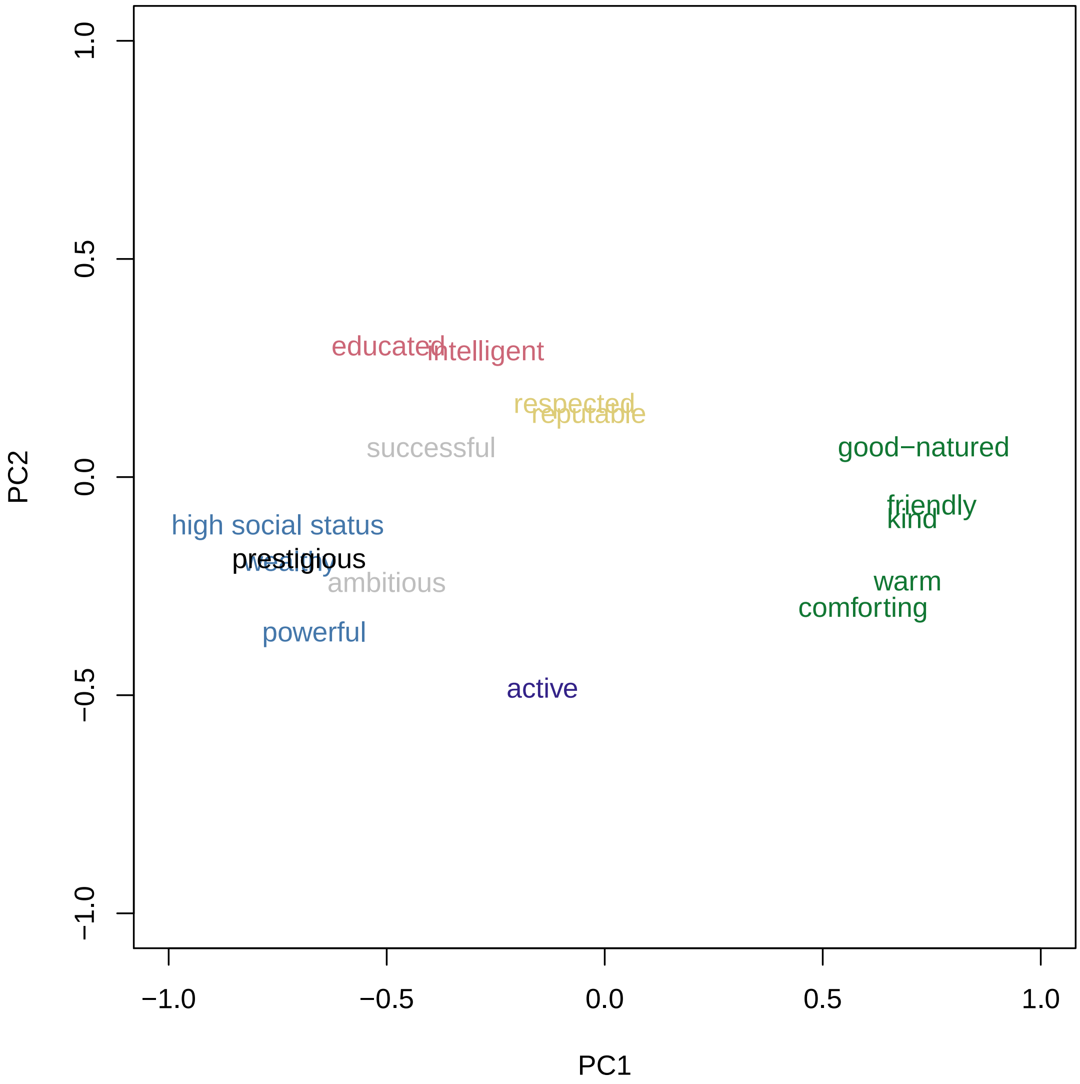


```
#   Combined plot for prestige, solidarity, and dynamism domains
# pdf("figure5.pdf", width=12, height=6)

par(mfrow=c(1,2))

par(pty="m", mar=.1+c(4,7,2,2))
plot(a.pam5.sil, main="", max.strlen=40, do.n.k=F,
     col=c("#4477AA","#4477AA","black","#4477AA","gray",
        "#CC6677","gray","#CC6677","#DDCC77","#332288","#DDCC77",
        "#117733","#117733","#117733","#117733","#117733"))
```

```
par(pty="s")
plot(a.pam5.mds, type="n", asp=1, xlim=c(-1,1), ylim=c(-1,1), xlab="PC1", ylab="PC2")
text(a.pam5.mds[,1], a.pam5.mds[,2], labels=rownames(a.pam5.mds), cex=1,
     col=c("#4477AA","#4477AA","black","#4477AA","gray",
           "#CC6677","gray","#CC6677","#DDCC77","#332288","#DDCC77",
           "#117733","#117733","#117733","#117733","#117733"))
```

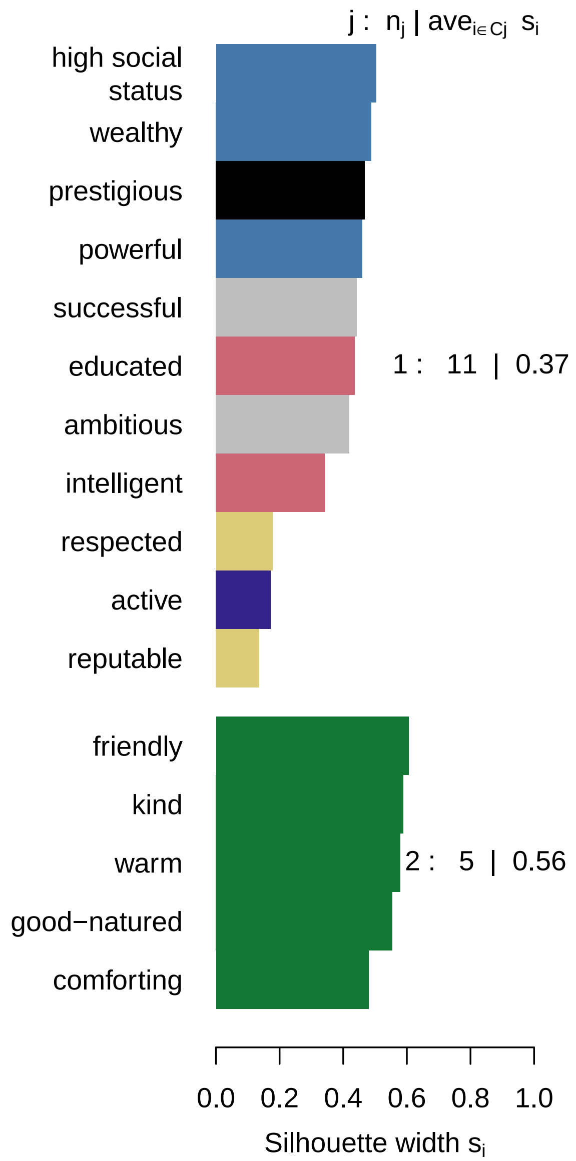

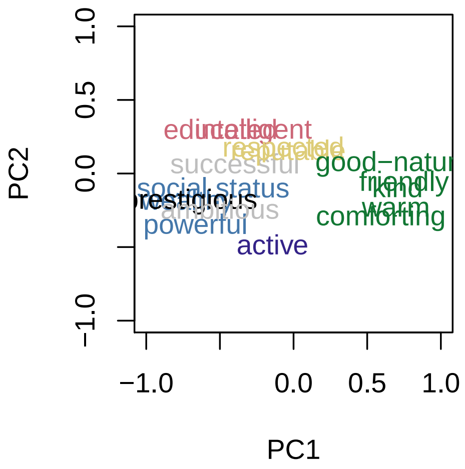


```
# dev.off()
```

Internal Prestige Domain

```r
#   Determine number of clusters
a.p.pamk = pamk(t(data.s[,c(2,4,8,13:14,16:20,22)]), 1:9, metric="manhattan")
a.p.pamk[2:3]
```

```
## $nc
## [1] 3
##
## $crit
## [1] 0.00000000 0.18750441 0.19370643 0.16097934 0.16387290 0.13039051
## [7] 0.09539619 0.07358597 0.04879458
```

```r
#   Run PAM with 3 clusters
a.p.pam = pam(t(data.s[,c(2,4,8,13:14,16:20,22)]), 3, metric="manhattan")
summary(a.p.pam)[6:7]
```

```
## $clusinfo
##      size max_diss   av_diss diameter separation
## [1,]    1        0    0.0000        0       1430
## [2,]    6     1130 866.1667     1515       1115
## [3,]    4     1043 680.2500     1135       1115
##
## $silinfo
## $silinfo$widths
##                    cluster neighbor   sil_width
## active                   1        2  0.00000000
## intelligent              2        3  0.26364100
## respected                2        1  0.25195638
## reputable                2        1  0.22165928
## educated                 2        3  0.11705737
## successful               2        3  0.11067380
## ambitious                2        3 -0.07979671
## wealthy                  3        2  0.34612157
## high.social.status       3        2  0.32905930
## prestigious              3        2  0.31126908
## powerful                 3        2  0.25912962
##
## $silinfo$clus.avg.widths
## [1] 0.0000000 0.1475319 0.3113949
##
## $silinfo$avg.width
## [1] 0.1937064
```

```r
plot(a.p.pam, which.plots=2)
```

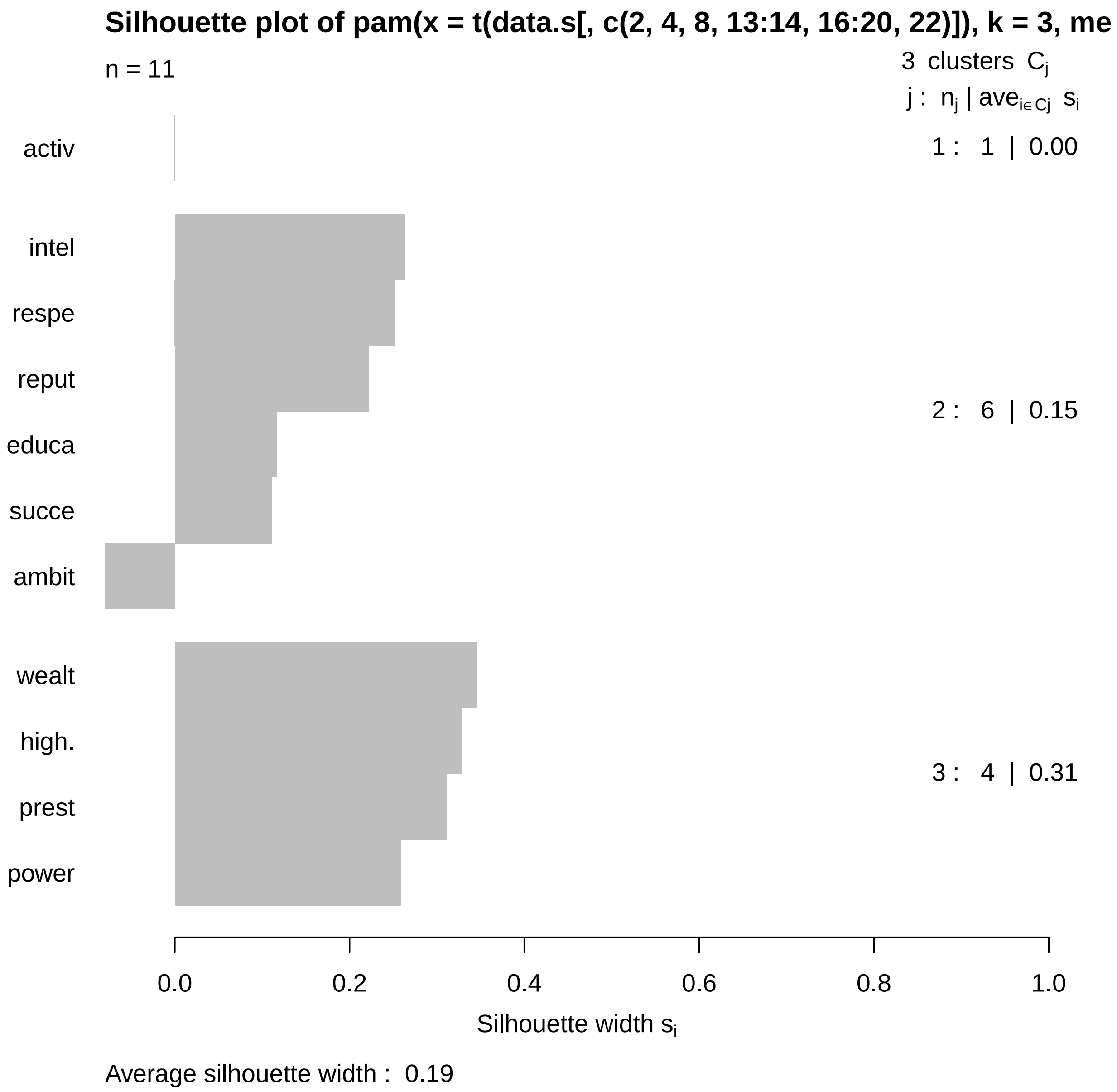


```
#   Remove 'active' and 'ambitious' and repeat
#       (Sequential removal in either order has same result)
#       'Prestigious' is also removed since all remaining items are within the
#           prestige domain
a.p.pamk2 = pamk(t(data.s[,c(8,13:14,16,18:20,22)]), 1:7, metric="manhattan")
a.p.pamk2[2:3]
```

```
## $nc
## [1] 2
##
## $crit
## [1] 0.00000000 0.27114892 0.22157011 0.16772404 0.11929514 0.09069578
## [7] 0.05529316
```

```
#   Run PAM with 2 clusters
a.p.pam2a = pam(t(data.s[,c(8,13:14,16,18:20,22)]), 2, metric="manhattan")
summary(a.p.pam2a)[6:7]
```

```
## $clusinfo
##      size max_diss av_diss diameter separation
## [1,]    5     1163   812.2     1353       1115
## [2,]    3      997   597.0     1043       1115
##
## $silinfo
## $silinfo$widths
##                    cluster neighbor sil_width
## intelligent              1        2 0.3104483
## respected                1        2 0.2994681
## reputable                1        2 0.2892959
## educated                 1        2 0.1544659
## successful               1        2 0.1026163
## wealthy                  2        1 0.3706957
## high.social.status       2        1 0.3308320
## powerful                 2        1 0.3113692
##
## $silinfo$clus.avg.widths
## [1] 0.2312589 0.3376323
##
## $silinfo$avg.width
## [1] 0.2711489
```

```
#   Run PAM with 3 clusters
a.p.pam2b = pam(t(data.s[,c(8,13:14,16,18:20,22)]), 3, metric="manhattan")
summary(a.p.pam2b)[6:7]
```

```
## $clusinfo
##      size max_diss  av_diss diameter separation
## [1,]    3      965 600.3333      983       1038
## [2,]    3      997 597.0000     1043       1115
## [3,]    2      984 492.0000      984       1038
##
## $silinfo
## $silinfo$widths
##                    cluster neighbor sil_width
## educated                 1        3 0.3067835
## intelligent              1        3 0.2030973
## successful               1        3 0.1014760
## wealthy                  2        1 0.3099152
## powerful                 2        1 0.2717753
## high.social.status       2        1 0.2558736
## reputable                3        1 0.1903456
## respected                3        1 0.1332942
##
```

```
## $silinfo$clus.avg.widths
## [1] 0.2037856 0.2791881 0.1618199
##
## $silinfo$avg.width
## [1] 0.2215701
```

```
#   Plot silhouette plots
par(mfrow=c(1,2))
plot(a.p.pam2a, which.plots=2)
plot(a.p.pam2b, which.plots=2)
```

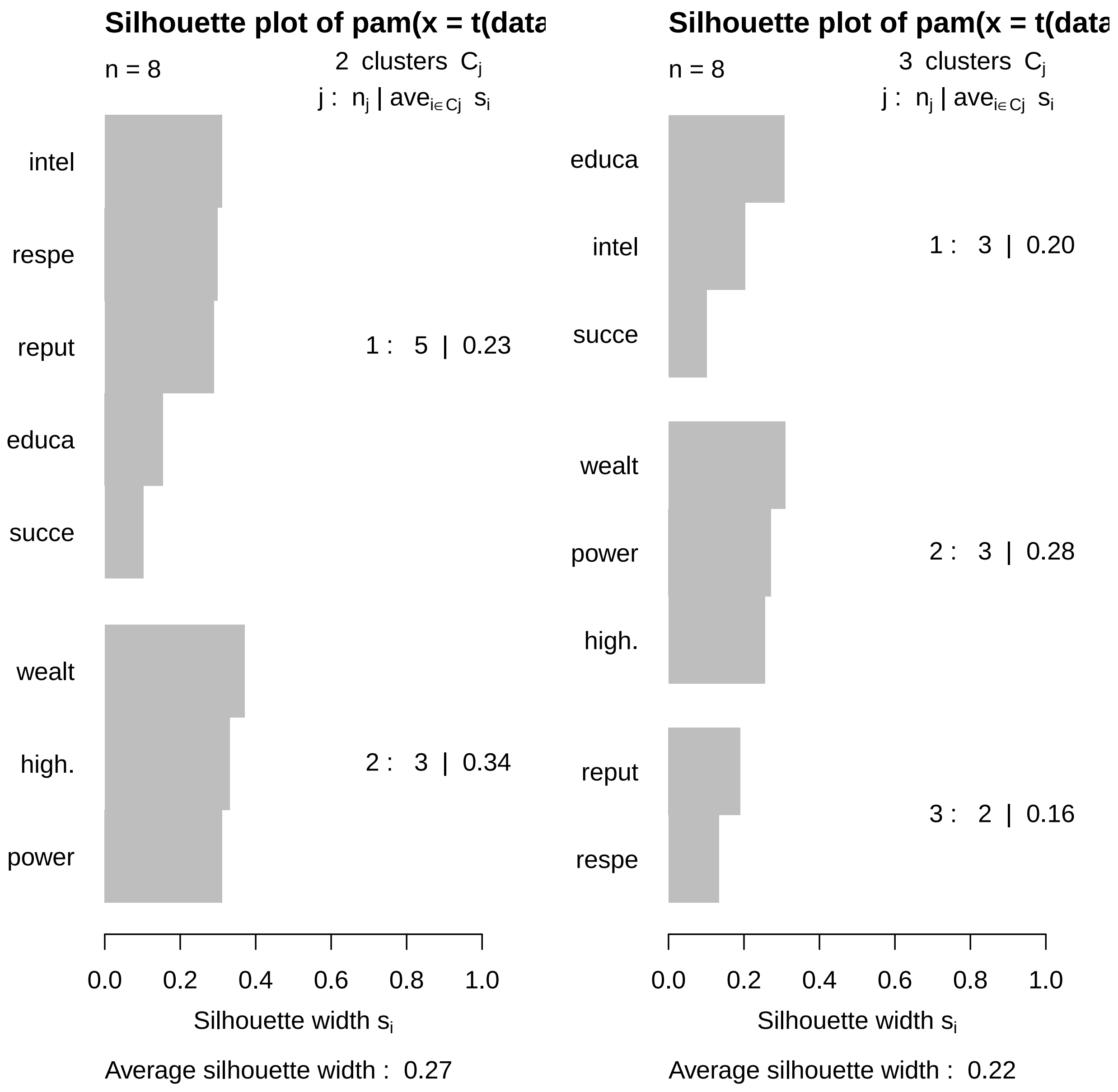

Though the silhouette width for *successful* is slightly above 0.10 in both cases, its removal leads to an improvement in structure (silhouette widths).

```r
#   Remove 'successful' and repeat
a.p.pamk3 = pamk(t(data.s[,c(8,13:14,16,18,19,22)]), 1:6, metric="manhattan")
a.p.pamk3[2:3]
```

```
## $nc
## [1] 2
##
## $crit
## [1] 0.00000000 0.29957234 0.28189891 0.20276792 0.14434215 0.06319218
```

```r
#   Run PAM with 2 clusters
a.p.pam3a = pam(t(data.s[,c(8,13:14,16,18,19,22)]), 2, metric="manhattan")
summary(a.p.pam3a)[6:7]
```

```
## $clusinfo
##      size max_diss av_diss diameter separation
## [1,]    4     1163     774     1353       1194
## [2,]    3      997     597     1043       1194
##
## $silinfo
## $silinfo$widths
##                     cluster neighbor sil_width
## intelligent               1        2 0.2990718
## respected                 1        2 0.2868085
## reputable                 1        2 0.2836676
## educated                  1        2 0.1220502
## wealthy                   2        1 0.4018036
## high.social.status        2        1 0.3608212
## powerful                  2        1 0.3427835
##
## $silinfo$clus.avg.widths
## [1] 0.2478995 0.3684694
##
## $silinfo$avg.width
## [1] 0.2995723
```

```r
#   Run PAM with 3 clusters
a.p.pam3b = pam(t(data.s[,c(8,13:14,16,18,19,22)]), 3, metric="manhattan")
summary(a.p.pam3b)[6:7]
```

```
## $clusinfo
##      size max_diss av_diss diameter separation
## [1,]    2      836     418      836       1097
## [2,]    3      997     597     1043       1194
## [3,]    2      984     492      984       1097
##
## $silinfo
## $silinfo$widths
##                     cluster neighbor sil_width
## educated                  1        3 0.3628049
```

```
## intelligent              1        3 0.2601770
## wealthy                  2        1 0.3524946
## powerful                 2        1 0.3209055
## high.social.status       2        1 0.2901855
## reputable                3        1 0.2178060
## respected                3        1 0.1689189
##
## $silinfo$clus.avg.widths
## [1] 0.3114909 0.3211952 0.1933625
##
## $silinfo$avg.width
## [1] 0.2818989
```

```
#   Plot silhouette plots
par(mfrow=c(1,2))
plot(a.p.pam3a, which.plots=2)
plot(a.p.pam3b, which.plots=2)
```

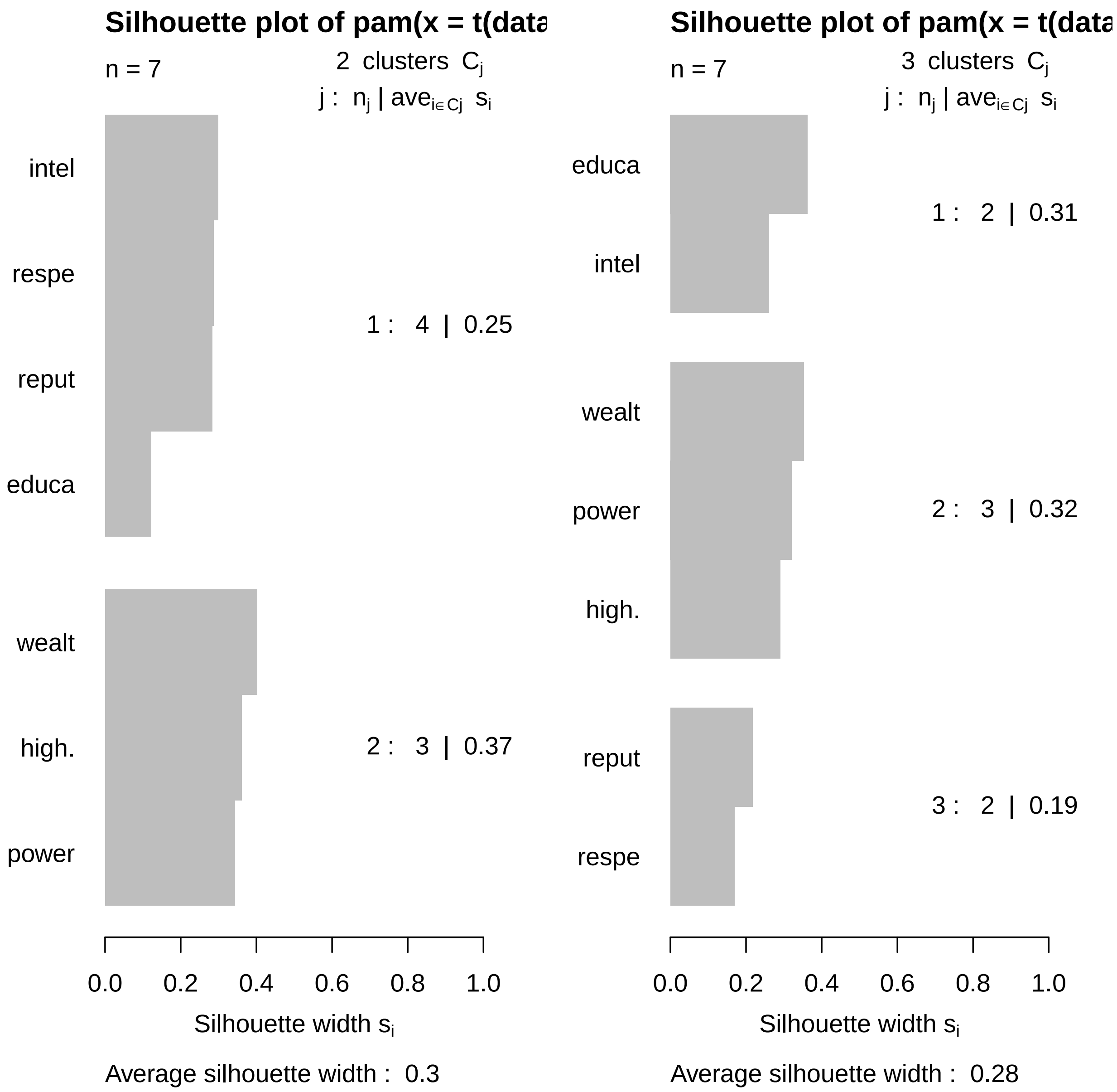


```
#   Evaluate cluster validation statistics
#       2-cluster solution
cluster.stats(dist(t(data.s[,c(8,13:14,16,18,19,22)]), "manhattan"),
              a.p.pam3a$clustering)

## $n
## [1] 7
##
## $cluster.number
## [1] 2
##
## $cluster.size
## [1] 4 3
```

```
##
## $min.cluster.size
## [1] 3
##
## $noisen
## [1] 0
##
## $diameter
## [1] 1353 1043
##
## $average.distance
## [1] 1117.3333  944.6667
##
## $median.distance
## [1] 1130  997
##
## $separation
## [1] 1194 1194
##
## $average.toother
## [1] 1495.333 1495.333
##
## $separation.matrix
##      [,1] [,2]
## [1,]    0 1194
## [2,] 1194    0
##
## $ave.between.matrix
##          [,1]     [,2]
## [1,]    0.000 1495.333
## [2,] 1495.333    0.000
##
## $average.between
## [1] 1495.333
##
## $average.within
## [1] 1059.778
##
## $n.between
## [1] 12
##
## $n.within
## [1] 9
##
## $max.diameter
## [1] 1353
##
## $min.separation
```

```
## [1] 1194
##
## $within.cluster.ss
## [1] 2821393
##
## $clus.avg.silwidths
##         1         2
## 0.2478995 0.3684694
##
## $avg.silwidth
## [1] 0.2995723
##
## $g2
## NULL
##
## $g3
## NULL
##
## $pearsongamma
## [1] 0.816113
##
## $dunn
## [1] 0.8824834
##
## $dunn2
## [1] 1.338305
##
## $entropy
## [1] 0.6829081
##
## $wb.ratio
## [1] 0.7087234
##
## $ch
## [1] 4.475973
##
## $cwidegap
## [1] 1097  997
##
## $widestgap
## [1] 1097
##
## $sindex
## [1] 1194
##
## $corrected.rand
## NULL
##
```

```
## $vi
## NULL
```

```
#       3-cluster solution
cluster.stats(dist(t(data.s[,c(8,13:14,16,18,19,22)]), "manhattan"),
              a.p.pam3b$clustering)
```

```
## $n
## [1] 7
##
## $cluster.number
## [1] 3
##
## $cluster.size
## [1] 2 3 2
##
## $min.cluster.size
## [1] 2
##
## $noisen
## [1] 0
##
## $diameter
## [1]  836 1043  984
##
## $average.distance
## [1] 836.0000 944.6667 984.0000
##
## $median.distance
## [1] 836 997 984
##
## $separation
## [1] 1097 1194 1097
##
## $average.toother
## [1] 1324.200 1495.333 1447.000
##
## $separation.matrix
##      [,1] [,2] [,3]
## [1,]    0 1194 1097
## [2,] 1194    0 1543
## [3,] 1097 1543    0
##
## $ave.between.matrix
##      [,1]     [,2]     [,3]
## [1,]    0 1393.000 1221.000
## [2,] 1393    0.000 1597.667
## [3,] 1221 1597.667    0.000
##
```

```
## $average.between
## [1] 1426.75
##
## $average.within
## [1] 930.8
##
## $n.between
## [1] 16
##
## $n.within
## [1] 5
##
## $max.diameter
## [1] 1043
##
## $min.separation
## [1] 1097
##
## $within.cluster.ss
## [1] 1737674
##
## $clus.avg.silwidths
##         1         2         3
## 0.3114909 0.3211952 0.1933625
##
## $avg.silwidth
## [1] 0.2818989
##
## $g2
## NULL
##
## $g3
## NULL
##
## $pearsongamma
## [1] 0.7997933
##
## $dunn
## [1] 1.051774
##
## $dunn2
## [1] 1.240854
##
## $entropy
## [1] 1.078992
##
## $wb.ratio
## [1] 0.6523918
```

```
##
## $ch
## [1] 4.154306
##
## $cwidegap
## [1] 836 997 984
##
## $widestgap
## [1] 997
##
## $sindex
## [1] 1097
##
## $corrected.rand
## NULL
##
## $vi
## NULL
```

```
#   Plot 3-cluster silhouette plot
a.p.pam3b.sil = silhouette(a.p.pam3b)
a.p.pam3b.sil = a.p.pam3b.sil[c(3,4,5,6,7,1,2),]
a.p.pam3b.sil[,1] = c(1,1,1,2,2,3,3)
a.p.pam3b.sil[,2] = c(3,3,3,3,3,2,2)
rownames(a.p.pam3b.sil)[3] = "high social\nstatus"
attr(a.p.pam3b.sil, "Ordered") = T
attr(a.p.pam3b.sil, "class") = "silhouette"

par(mar=.1+c(4,7,2,2))
plot(a.p.pam3b.sil, main="", max.strlen=40, do.n.k=F,
     col=c("#4477AA","#DDCC77","#CC6677"))
```

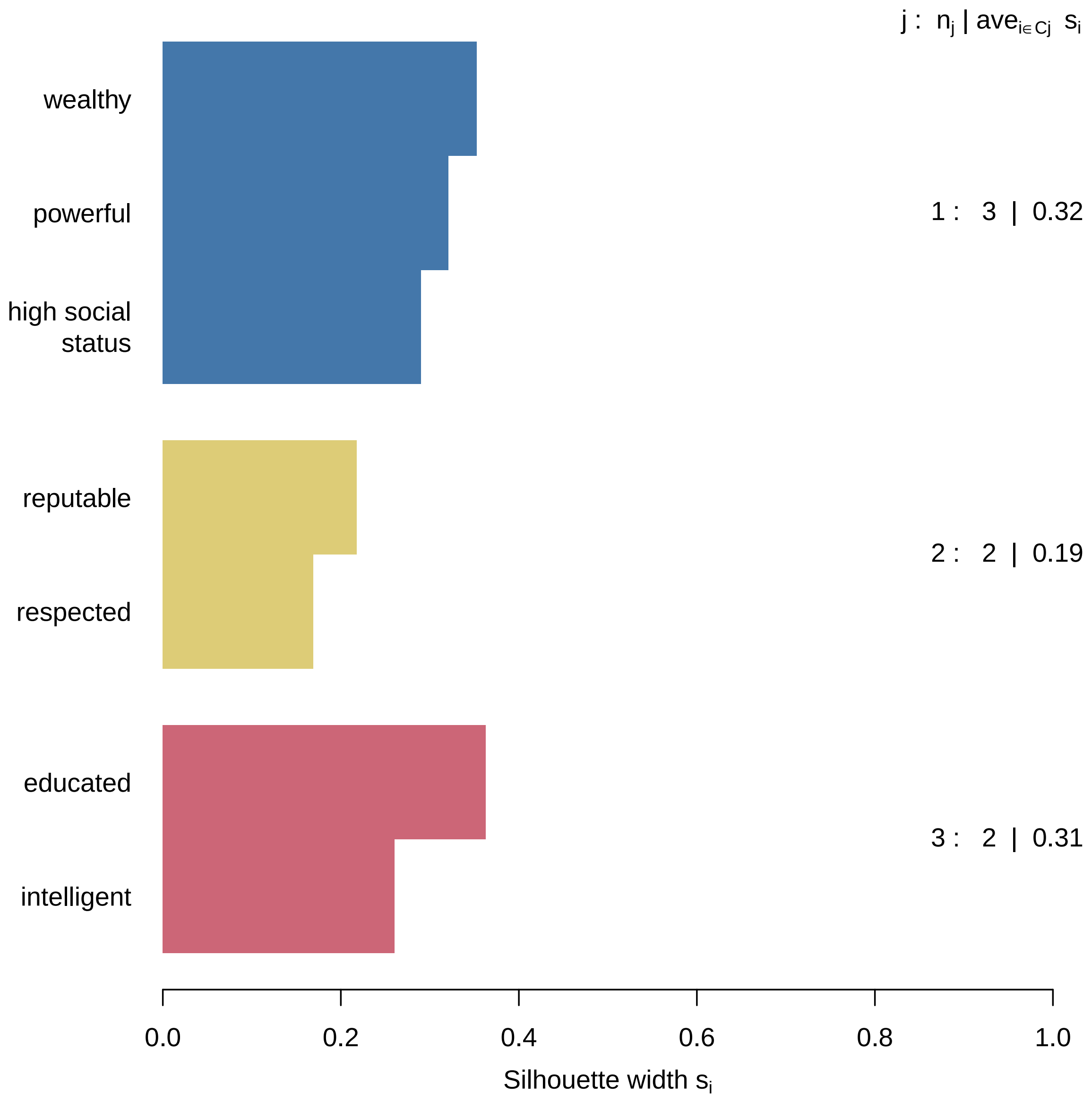


```
#   Plot multidimensional scaling plot
a.p.pam3b.mds = cmdscale(as.dist(as.matrix(a.p.pam3b$diss)))
a.p.pam3b.mds = a.p.pam3b.mds[c(7,4,2,5,6,1,3),]
rownames(a.p.pam3b.mds)[3] = "high social status"
a.p.pam3b.mds = a.p.pam3b.mds / 1000

par(pty="s")
plot(a.p.pam3b.mds, type="n", asp=1, xlim=c(-1,1), ylim=c(-1,1),
     xlab="PC1", ylab="PC2")
text(a.p.pam3b.mds[,1], a.p.pam3b.mds[,2], labels=rownames(a.p.pam3b.mds), cex=1,
     col=c(rep("#4477AA",3), rep("#DDCC77",2), rep("#CC6677",2)))
```

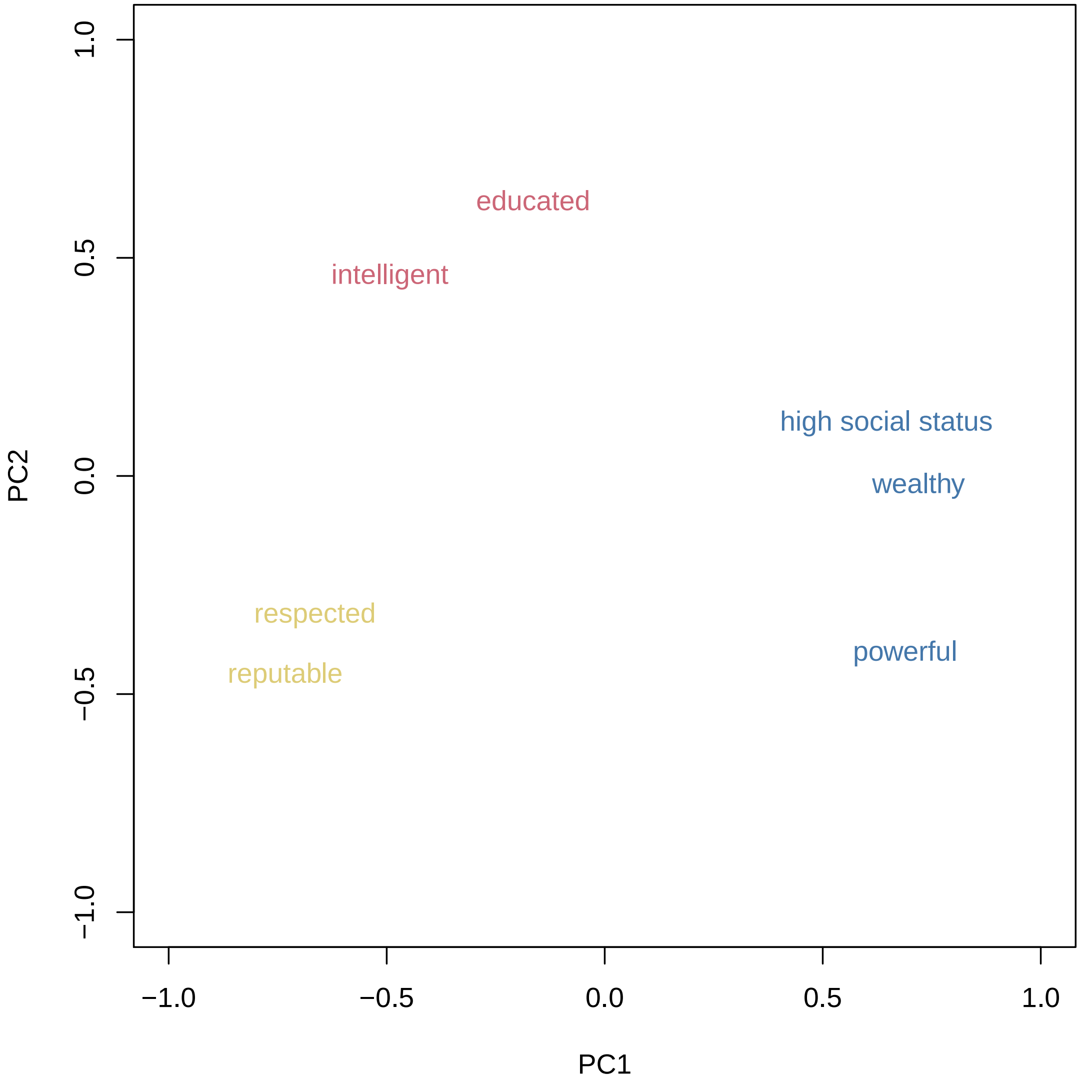

Triad Data

```
#   Convert triad correlation matrix to distance matrix
triad.dist = cor2dist(triad.cor)
round(as.dist(triad.dist), 3)
```

```
##                      prestigious wealthy high.social.status powerful
## wealthy                    1.516
## high.social.status         1.294   0.970
## powerful                   1.487   1.273              1.370
## respected                  1.501   1.554              1.559    1.524
```

```
## educated                  1.536   1.579              1.588    1.553
## hardworking               1.538   1.579              1.592    1.553
## successful                1.462   1.465              1.515    1.451
## intelligent               1.536   1.577              1.591    1.551
## reputable                 1.513   1.557              1.566    1.533
## ambitious                 1.515   1.557              1.572    1.527
##                     respected educated hardworking successful intelligent
## wealthy
## high.social.status
## powerful
## respected
## educated                1.560
## hardworking             1.556    1.439
## successful              1.483    1.513       1.503
## intelligent             1.559    0.609       1.500      1.519
## reputable               0.906    1.563       1.509      1.486       1.560
## ambitious               1.542    1.562       0.941      1.482       1.519
##                     reputable
## wealthy
## high.social.status
## powerful
## respected
## educated
## hardworking
## successful
## intelligent
## reputable
## ambitious               1.543
```

Checking Assumptions

```
#   Check clustering of distance matrix
plot(cmdscale(triad.dist, 2), type="n", xlab="Dimension 1", ylab="Dimension 2",
     main ="Multidimensional Scaling")
text(cmdscale(triad.dist, 2), labels=rownames(cmdscale(triad.dist, 2)))
```

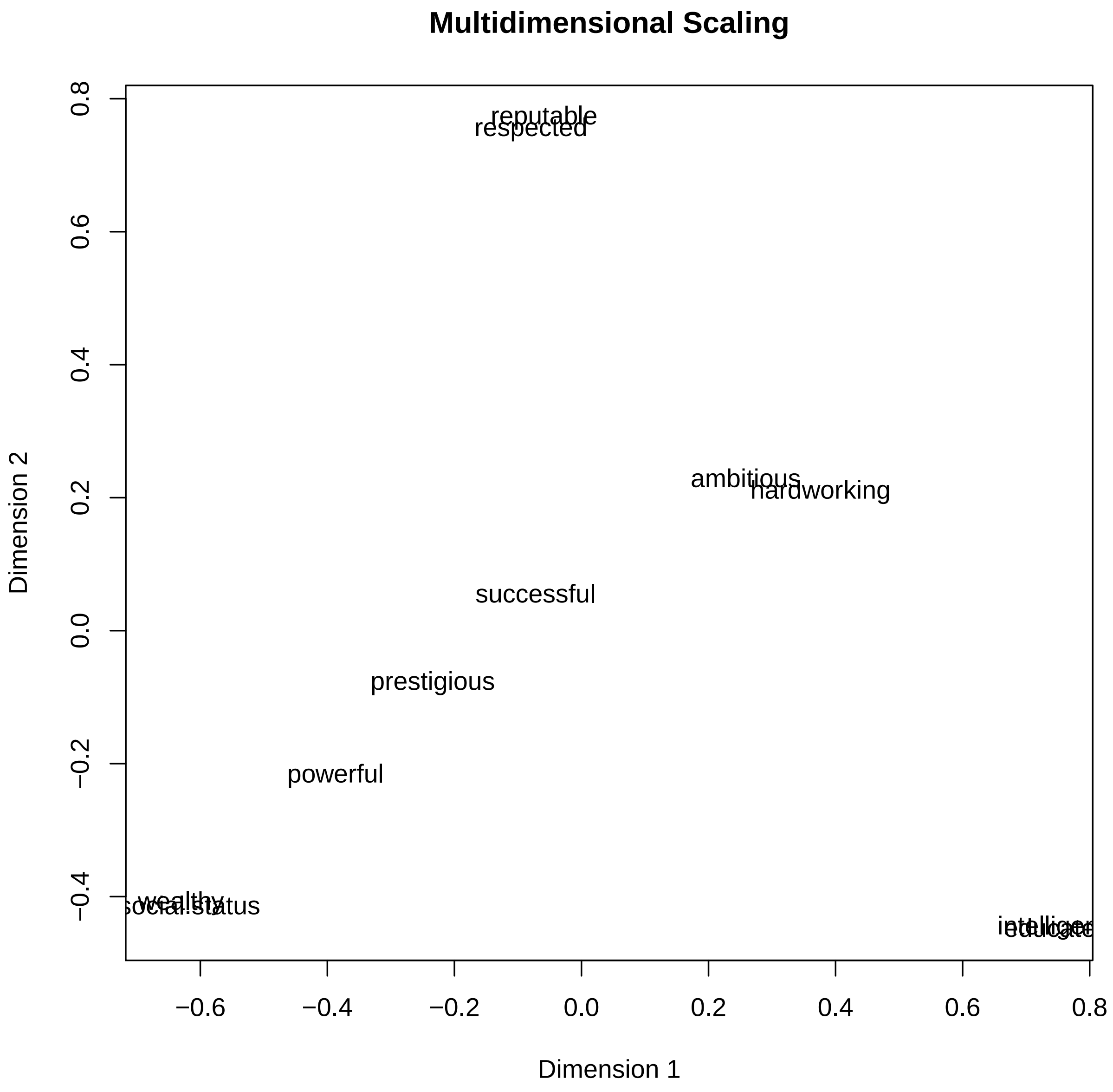

Partitioning Around Medoids (Prestige Domain Only)

```
#   Determine number of clusters
t.pamk = pamk(triad.dist[-1,-1], 2:9, diss=T)
t.pamk[2:3]
```

```
## $nc
## [1] 4
##
## $crit
## [1] 0.00000000 0.09961604 0.13373844 0.32469222 0.32467341 0.32245992
## [7] 0.26951644 0.19503530 0.11713497
```

```r
#   Run PAM with 4 clusters
t.pam = pam(triad.dist[-1,-1], 4, diss=T)
summary(t.pam)[6:7]
```

```
## $clusinfo
##      size  max_diss   av_diss  diameter separation
## [1,]    4 1.4653485 0.9272064 1.5150617   1.481778
## [2,]    2 0.9062835 0.4531418 0.9062835   1.483216
## [3,]    2 0.6085704 0.3042852 0.6085704   1.439266
## [4,]    2 0.9406949 0.4703475 0.9406949   1.439266
##
## $silinfo
## $silinfo$widths
##                      cluster neighbor   sil_width
## wealthy                    1        2 0.205385167
## high.social.status         1        2 0.177743733
## powerful                   1        2 0.107097317
## successful                 1        2 0.004885164
## respected                  2        1 0.407749938
## reputable                  2        4 0.406208611
## intelligent                3        4 0.596762138
## educated                   3        4 0.594412048
## ambitious                  4        1 0.386849277
## hardworking                4        3 0.359828824
##
## $silinfo$clus.avg.widths
## [1] 0.1237778 0.4069793 0.5955871 0.3733391
##
## $silinfo$avg.width
## [1] 0.3246922
```

```r
plot(t.pam, which.plots=2)
```

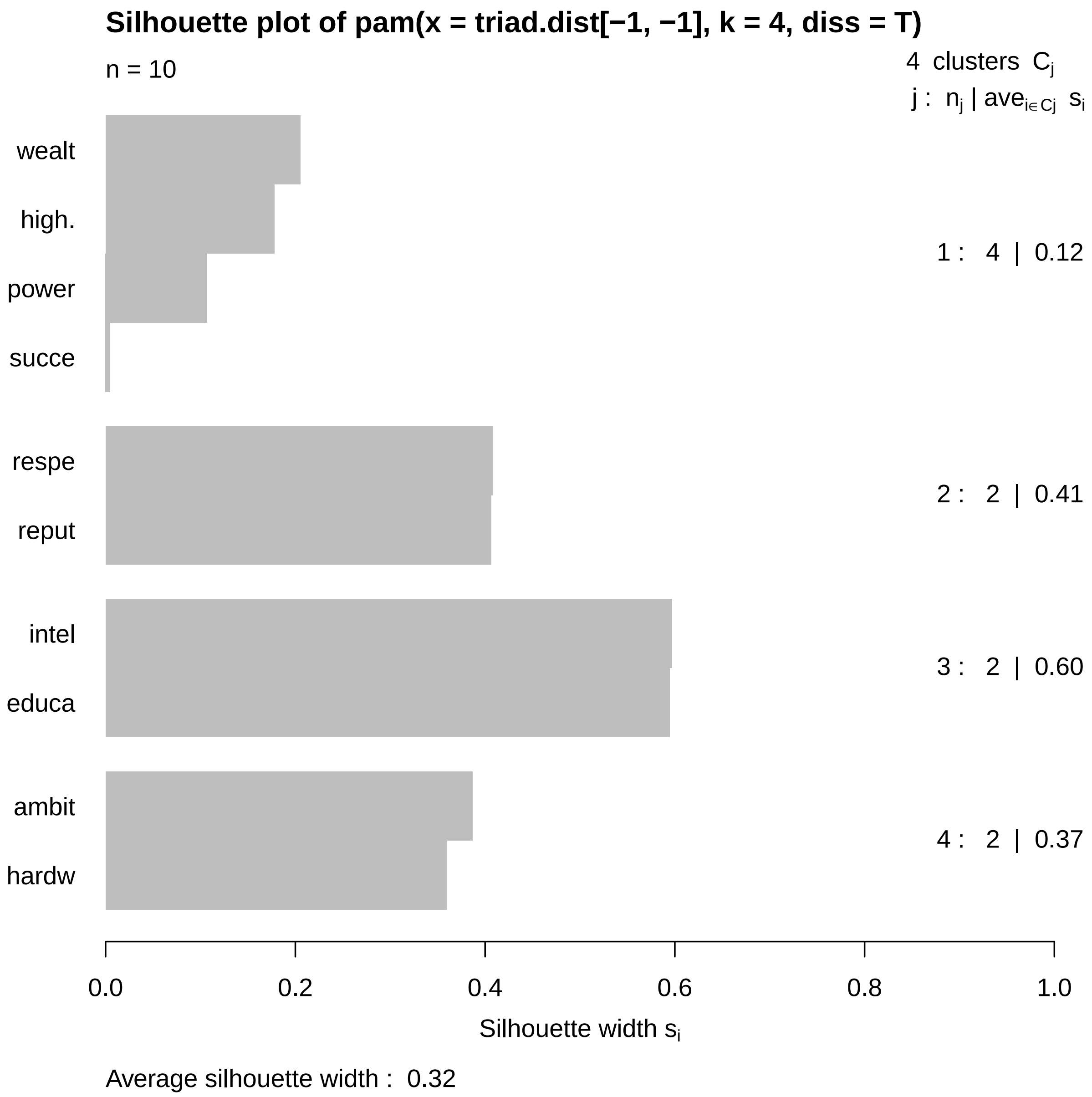


```
#   Remove 'successful' and repeat
t.pamk2 = pamk(triad.dist[c(2:7,9:11),c(2:7,9:11)], 2:8, diss=T)
t.pamk2[2:3]
```

```
## $nc
## [1] 4
##
## $crit
## [1] 0.0000000 0.1116944 0.1691533 0.3806584 0.3639776 0.3051515 0.2195737
## [8] 0.1301500
```

```
#   Run PAM with 4 clusters
t.pam2 = pam(triad.dist[c(2:7,9:11),c(2:7,9:11)], 4, diss=T)
```

```r
summary(t.pam2)[6:7]
```

```
## $clusinfo
##      size  max_diss   av_diss  diameter separation
## [1,]    3 1.2730888 0.7478257 1.3699592   1.523932
## [2,]    2 0.9062835 0.4531418 0.9062835   1.509250
## [3,]    2 0.6085704 0.3042852 0.6085704   1.439266
## [4,]    2 0.9406949 0.4703475 0.9406949   1.439266
##
## $silinfo
## $silinfo$widths
##                    cluster neighbor sil_width
## wealthy                  1        2 0.2790035
## high.social.status       1        2 0.2512992
## powerful                 1        2 0.1354056
## respected                2        1 0.4137547
## reputable                2        4 0.4062086
## intelligent              3        4 0.5967621
## educated                 3        4 0.5944120
## ambitious                4        3 0.3892508
## hardworking              4        3 0.3598288
##
## $silinfo$clus.avg.widths
## [1] 0.2219028 0.4099817 0.5955871 0.3745398
##
## $silinfo$avg.width
## [1] 0.3806584
```

```r
plot(t.pam2, which.plots=2)
```

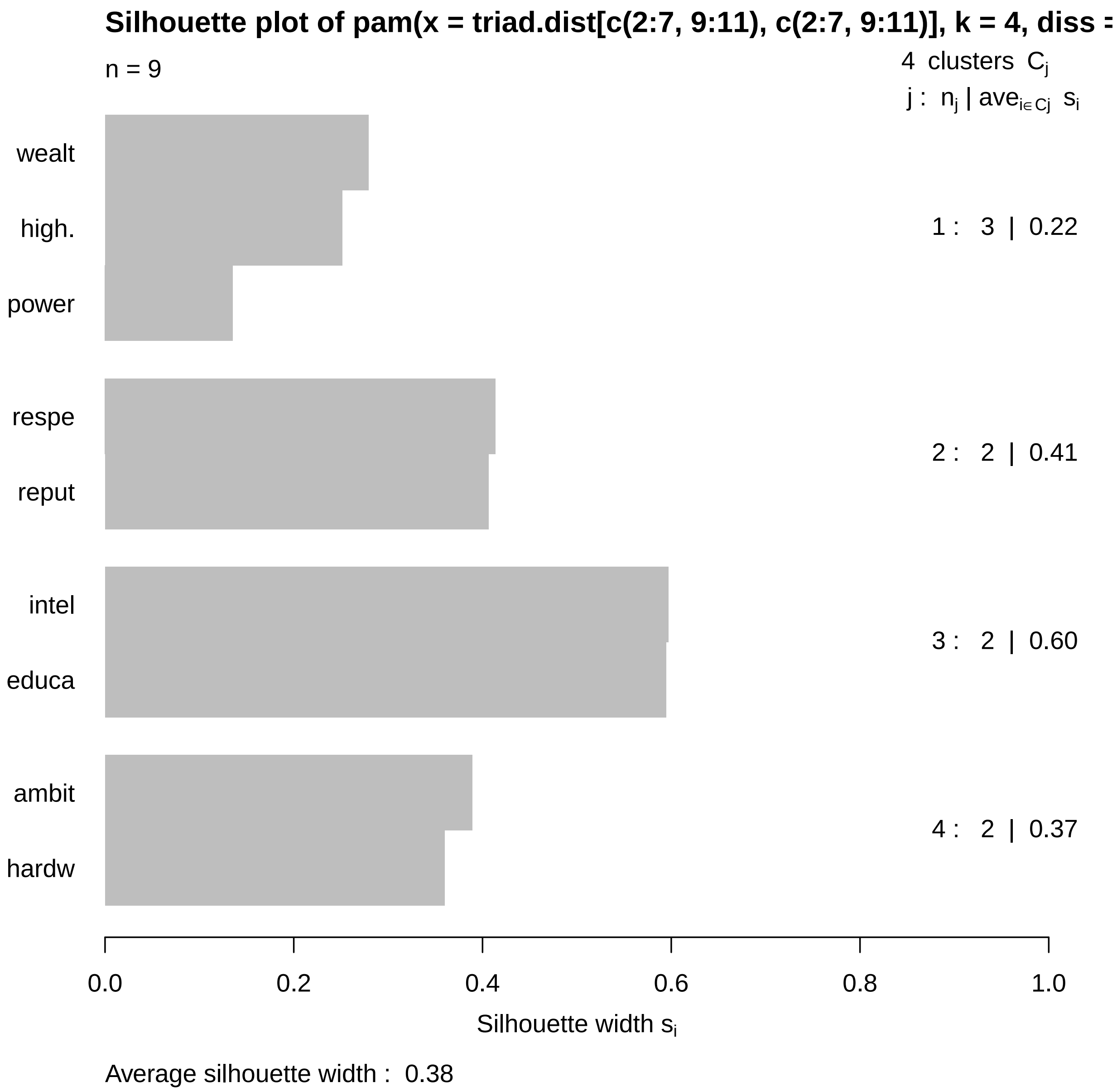

Analyses of the attitudinal data showed that *hardworking* has poor fit within the prestige domain, so we remove it here.

```
#   Remove 'hardworking' and repeat
t.pamk3 = pamk(triad.dist[c(2:6,9:11),c(2:6,9:11)], 2:7, diss=T)
t.pamk3[2:3]
```

```
## $nc
## [1] 4
##
## $crit
## [1] 0.0000000 0.1512716 0.2650205 0.3373963 0.3188783 0.2526989 0.1509321
```

```
#   Run PAM with 4 clusters
```

```
t.pam3 = pam(triad.dist[c(2:6,9:11),c(2:6,9:11)], 4, diss=T)
summary(t.pam3)[6:7]
```

```
## $clusinfo
##      size  max_diss   av_diss  diameter separation
## [1,]    3 1.2730888 0.7478257 1.3699592   1.523932
## [2,]    2 0.9062835 0.4531418 0.9062835   1.523932
## [3,]    2 0.6085704 0.3042852 0.6085704   1.518800
## [4,]    1 0.0000000 0.0000000 0.0000000   1.518800
##
## $silinfo
## $silinfo$widths
##                    cluster neighbor sil_width
## wealthy                  1        2 0.2790035
## high.social.status       1        2 0.2512992
## powerful                 1        4 0.1344120
## reputable                2        4 0.4127555
## respected                2        4 0.4121302
## educated                 3        2 0.6102614
## intelligent              3        4 0.5993083
## ambitious                4        3 0.0000000
##
## $silinfo$clus.avg.widths
## [1] 0.2215716 0.4124429 0.6047849 0.0000000
##
## $silinfo$avg.width
## [1] 0.3373963
```

```
plot(t.pam3, which.plots=2)
```

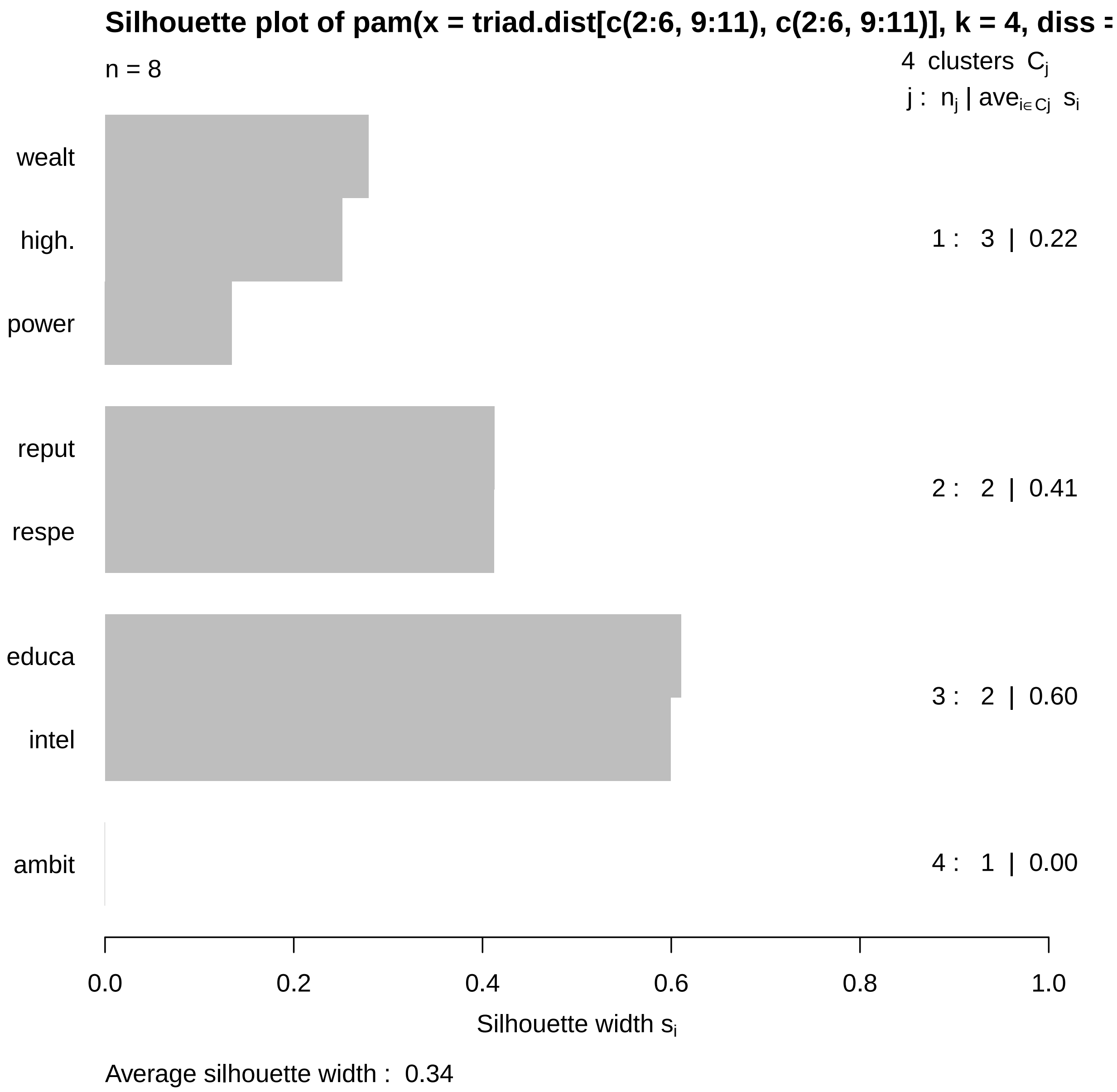


```
#   Remove 'ambitious' and repeat
t.pamk4 = pamk(triad.dist[c(2:6,9:10),c(2:6,9:10)], 2:6, diss=T)
t.pamk4[2:3]
```

```
## $nc
## [1] 3
##
## $crit
## [1] 0.0000000 0.2597561 0.3879568 0.3656060 0.2899724 0.1736675
```

```
#   Run PAM with 3 clusters
t.pam4 = pam(triad.dist[c(2:6,9:10),c(2:6,9:10)], 3, diss=T)
summary(t.pam4)[6:7]
```

```
## $clusinfo
##      size  max_diss   av_diss  diameter separation
## [1,]    3 1.2730888 0.7478257 1.3699592   1.523932
## [2,]    2 0.9062835 0.4531418 0.9062835   1.523932
## [3,]    2 0.6085704 0.3042852 0.6085704   1.550592
##
## $silinfo
## $silinfo$widths
##                    cluster neighbor sil_width
## wealthy                  1        2 0.2790035
## high.social.status       1        2 0.2512992
## powerful                 1        2 0.1354056
## reputable                2        1 0.4161493
## respected                2        1 0.4137547
## educated                 3        2 0.6102614
## intelligent              3        2 0.6098236
##
## $silinfo$clus.avg.widths
## [1] 0.2219028 0.4149520 0.6100425
##
## $silinfo$avg.width
## [1] 0.3879568
```

```
plot(t.pam4, which.plots=2)
```

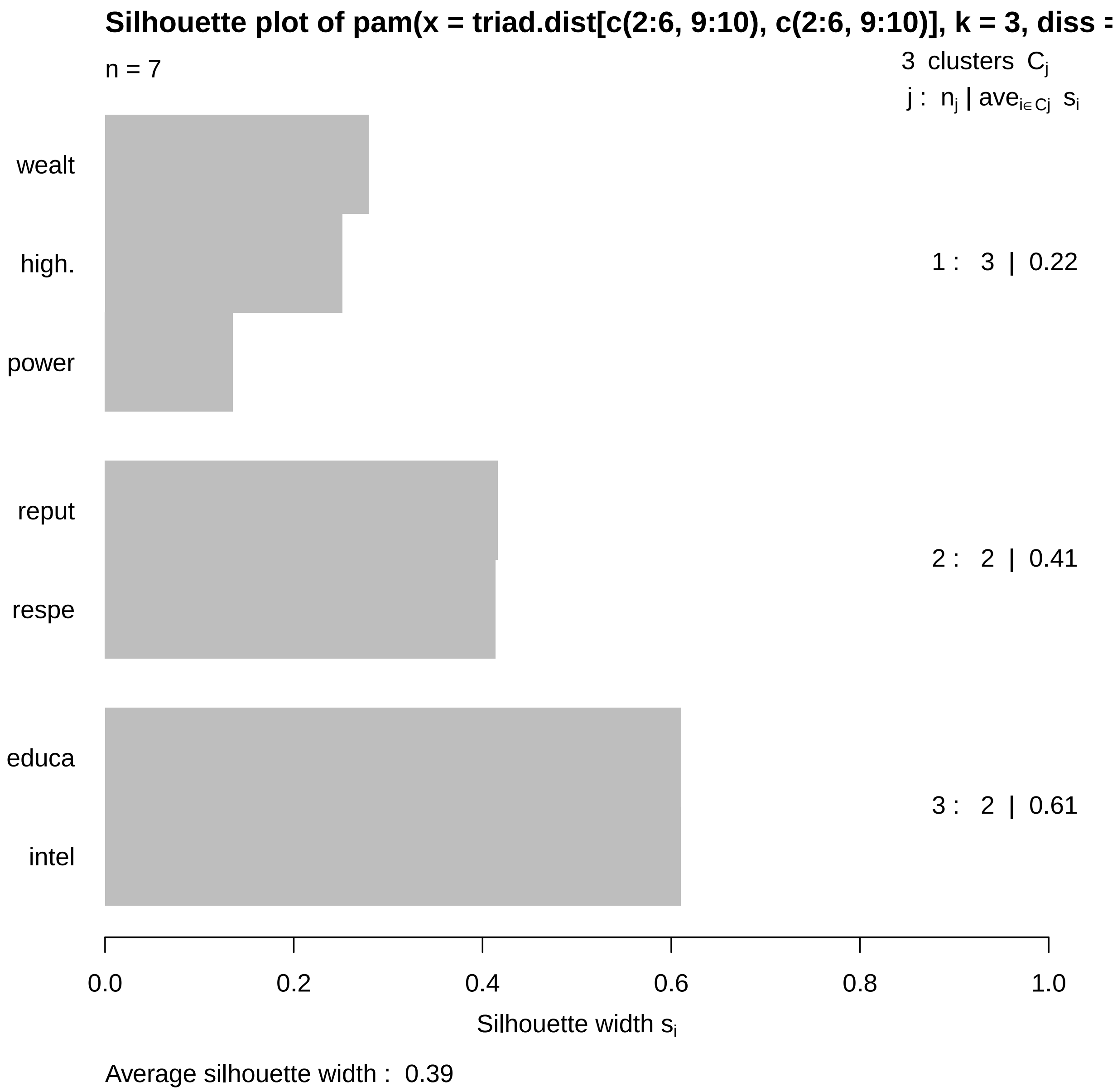


```
#   Evaluate cluster validation statistics
#       3-cluster solution
cluster.stats(triad.dist[c(2:6,9:10),c(2:6,9:10)],
              t.pam4$clustering)

## $n
## [1] 7
##
## $cluster.number
## [1] 3
##
## $cluster.size
## [1] 3 2 2
```

```
##
## $min.cluster.size
## [1] 2
##
## $noisen
## [1] 0
##
## $diameter
## [1] 1.3699592 0.9062835 0.6085704
##
## $average.distance
## [1] 1.2044788 0.9062835 0.6085704
##
## $median.distance
## [1] 1.2730888 0.9062835 0.6085704
##
## $separation
## [1] 1.523932 1.523932 1.550592
##
## $average.toother
## [1] 1.561142 1.553692 1.568164
##
## $separation.matrix
##          [,1]     [,2]     [,3]
## [1,] 0.000000 1.523932 1.550592
## [2,] 1.523932 0.000000 1.559031
## [3,] 1.550592 1.559031 0.000000
##
## $ave.between.matrix
##          [,1]     [,2]     [,3]
## [1,] 0.000000 1.549082 1.573202
## [2,] 1.549082 0.000000 1.560607
## [3,] 1.573202 1.560607 0.000000
##
## $average.between
## [1] 1.561008
##
## $average.within
## [1] 1.025658
##
## $n.between
## [1] 16
##
## $n.within
## [1] 5
##
## $max.diameter
## [1] 1.369959
```

```
##
## $min.separation
## [1] 1.523932
##
## $within.cluster.ss
## [1] 2.075586
##
## $clus.avg.silwidths
##         1         2         3
## 0.2219028 0.4149520 0.6100425
##
## $avg.silwidth
## [1] 0.3879568
##
## $g2
## NULL
##
## $g3
## NULL
##
## $pearsongamma
## [1] 0.8625466
##
## $dunn
## [1] 1.112393
##
## $dunn2
## [1] 1.286102
##
## $entropy
## [1] 1.078992
##
## $wb.ratio
## [1] 0.6570484
##
## $ch
## [1] 4.142641
##
## $cwidegap
## [1] 1.2730888 0.9062835 0.6085704
##
## $widestgap
## [1] 1.273089
##
## $sindex
## [1] 1.523932
##
## $corrected.rand
```

```
## NULL
##
## $vi
## NULL
```

```
#       4-cluster solution including 'hardworking' and 'ambitious'
cluster.stats(triad.dist[c(2:7,9:11),c(2:7,9:11)],
              t.pam2$clustering)
```

```
## $n
## [1] 9
##
## $cluster.number
## [1] 4
##
## $cluster.size
## [1] 3 2 2 2
##
## $min.cluster.size
## [1] 2
##
## $noisen
## [1] 0
##
## $diameter
## [1] 1.3699592 0.9062835 0.6085704 0.9406949
##
## $average.distance
## [1] 1.2044788 0.9062835 0.6085704 0.9406949
##
## $median.distance
## [1] 1.2730888 0.9062835 0.6085704 0.9406949
##
## $separation
## [1] 1.523932 1.509250 1.439266 1.439266
##
## $average.toother
## [1] 1.561808 1.549102 1.550071 1.539192
##
## $separation.matrix
##          [,1]     [,2]     [,3]     [,4]
## [1,] 0.000000 1.523932 1.550592 1.526736
## [2,] 1.523932 0.000000 1.559031 1.509250
## [3,] 1.550592 1.559031 0.000000 1.439266
## [4,] 1.526736 1.509250 1.439266 0.000000
##
## $ave.between.matrix
##          [,1]     [,2]     [,3]     [,4]
## [1,] 0.000000 1.549082 1.573202 1.563139
```

```
## [2,] 1.549082 0.000000 1.560607 1.537627
## [3,] 1.573202 1.560607 0.000000 1.504837
## [4,] 1.563139 1.537627 1.504837 0.000000
##
## $average.between
## [1] 1.550827
##
## $average.within
## [1] 1.011498
##
## $n.between
## [1] 30
##
## $n.within
## [1] 6
##
## $max.diameter
## [1] 1.369959
##
## $min.separation
## [1] 1.439266
##
## $within.cluster.ss
## [1] 2.51804
##
## $clus.avg.silwidths
##         1         2         3         4
## 0.2219028 0.4099817 0.5955871 0.3745398
##
## $avg.silwidth
## [1] 0.3806584
##
## $g2
## NULL
##
## $g3
## NULL
##
## $pearsongamma
## [1] 0.8844058
##
## $dunn
## [1] 1.050591
##
## $dunn2
## [1] 1.249368
##
## $entropy
```

```
## [1] 1.368922
##
## $wb.ratio
## [1] 0.6522308
##
## $ch
## [1] 4.120888
##
## $cwidegap
## [1] 1.2730888 0.9062835 0.6085704 0.9406949
##
## $widestgap
## [1] 1.273089
##
## $sindex
## [1] 1.439266
##
## $corrected.rand
## NULL
##
## $vi
## NULL
```

```
#   Plot color-coded silhouette plot
t.pam4.sil = silhouette(t.pam4)
rownames(t.pam4.sil)[2] = "high social\nstatus"
attr(t.pam4.sil, "Ordered") = T
attr(t.pam4.sil, "class") = "silhouette"

par(mar=.1+c(4,7,2,2))
plot(t.pam4.sil, main="", max.strlen=40, do.n.k=F,
     col=c("#4477AA","#DDCC77","#CC6677"))
```

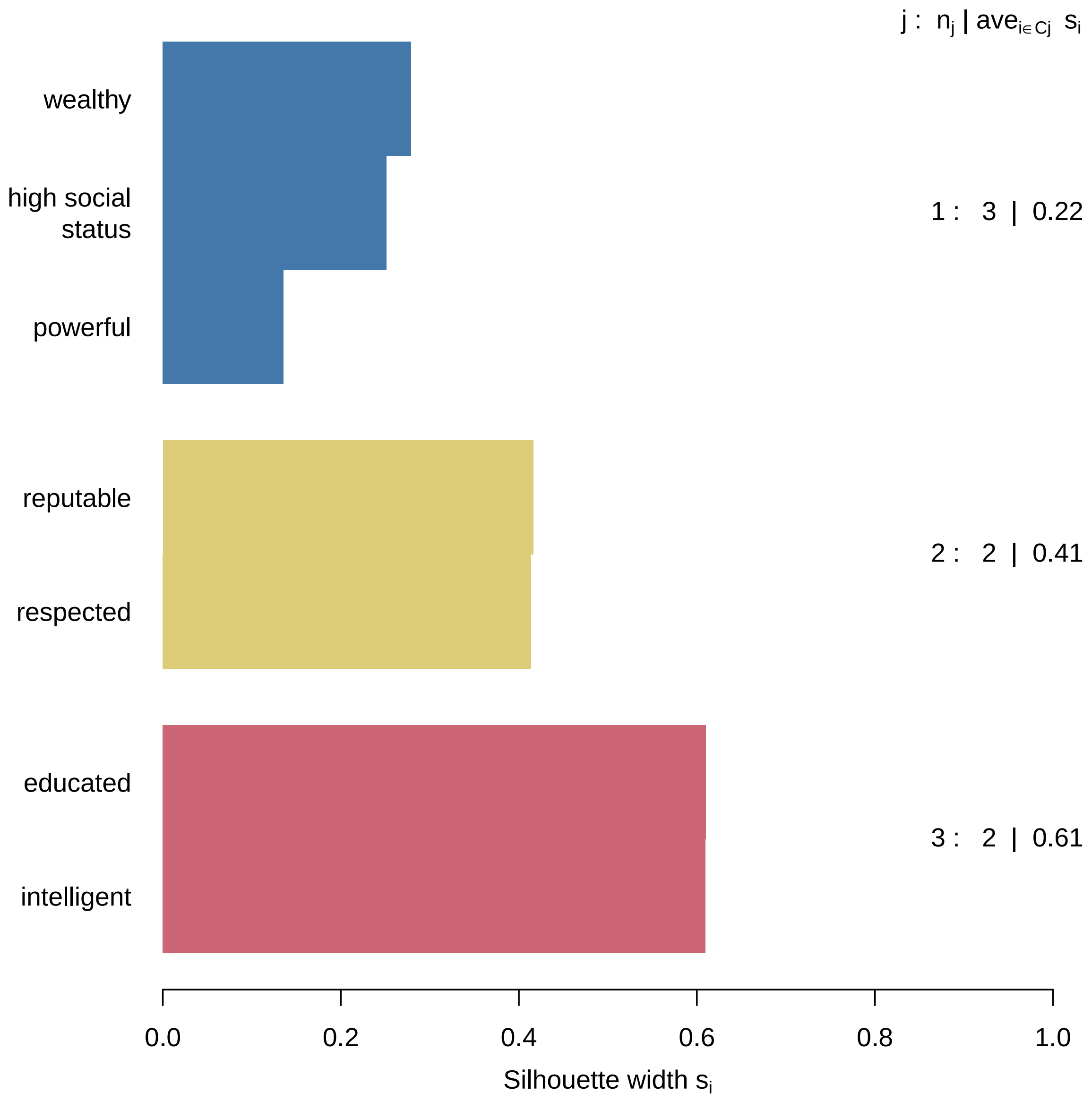


```
#   Plot color-coded multidimensional scaling plot
t.pam4.mds = cmdscale(as.dist(triad.dist[c(2:6,9:10),c(2:6,9:10)]))
t.pam4.mds = t.pam4.mds[c(1,2,3,7,4,5,6),]
rownames(t.pam4.mds)[2] = "high social status"

par(pty="s")
plot(t.pam4.mds, type="n", asp=1, xlim=c(-1,1), ylim=c(-1,1), xlab="PC1", ylab="PC2")
text(t.pam4.mds[,1], t.pam4.mds[,2], labels=rownames(t.pam4.mds), cex=1,
     col=c(rep("#4477AA",3), rep("#DDCC77",2), rep("#CC6677",2)))
```

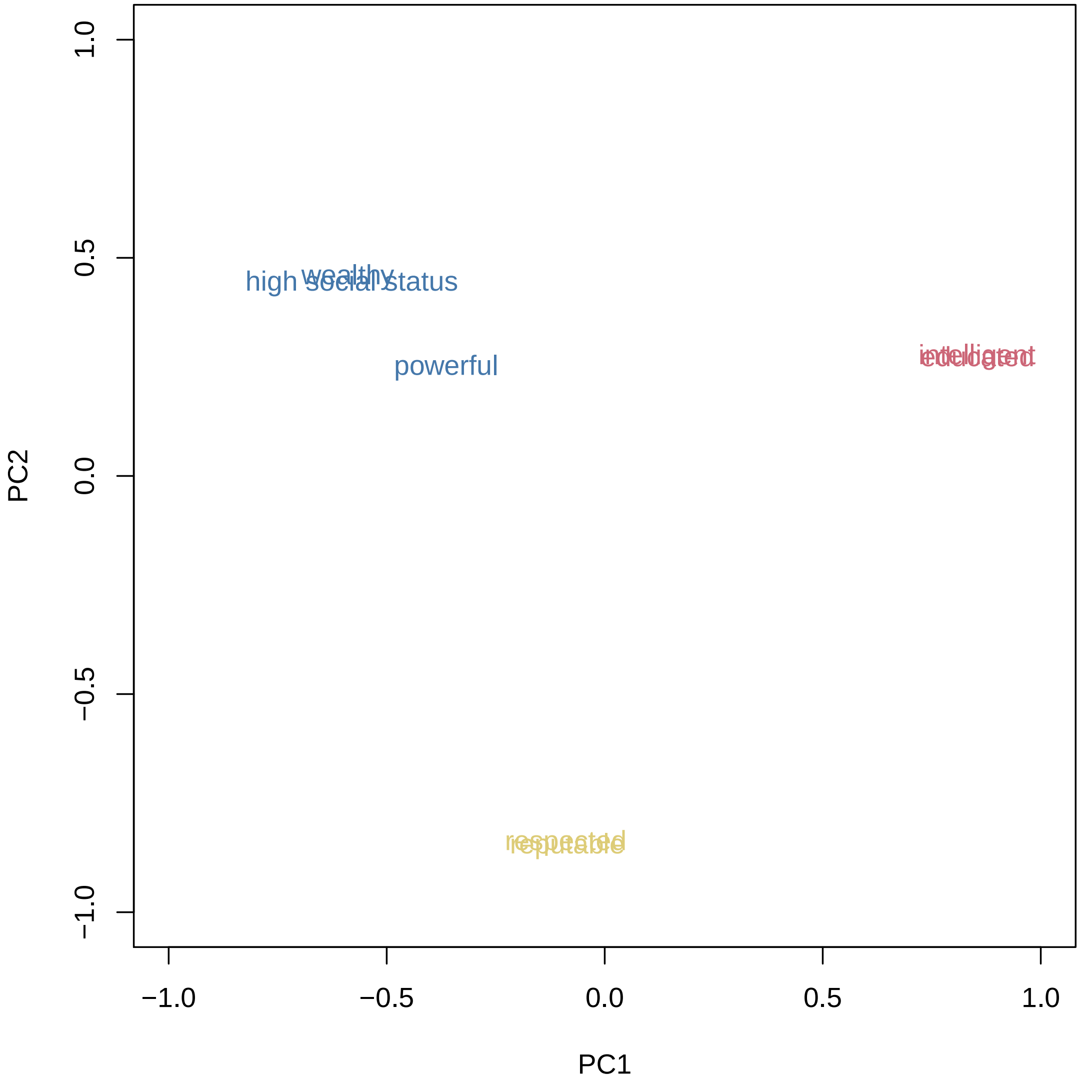


```
#   Combined plot for prestige domain from attitudinal and triad data
# pdf("figure6.pdf", width=12, height=9)

par(mfrow=c(2,2))

par(pty="m", mar=.1+c(4,7,2,2))
plot(a.p.pam3b.sil, main="", max.strlen=40, do.n.k=F,
     col=c("#4477AA","#DDCC77","#CC6677"))

par(pty="s")
plot(a.p.pam3b.mds, type="n", asp=1, xlim=c(-1,1), ylim=c(-1,1),
     xlab="PC1", ylab="PC2")
```

```
text(a.p.pam3b.mds[,1], a.p.pam3b.mds[,2], labels=rownames(a.p.pam3b.mds), cex=1,
     col=c(rep("#4477AA",3), rep("#DDCC77",2), rep("#CC6677",2)))

par(pty="m", mar=.1+c(4,7,2,2))
plot(t.pam4.sil, main="", max.strlen=40, do.n.k=F,
     col=c("#4477AA","#DDCC77","#CC6677"))

par(pty="s")
plot(t.pam4.mds, type="n", asp=1, xlim=c(-1,1), ylim=c(-1,1), xlab="PC1", ylab="PC2")
text(t.pam4.mds[,1], t.pam4.mds[,2], labels=rownames(t.pam4.mds), cex=1,
     col=c(rep("#4477AA",3), rep("#DDCC77",2), rep("#CC6677",2)))
```

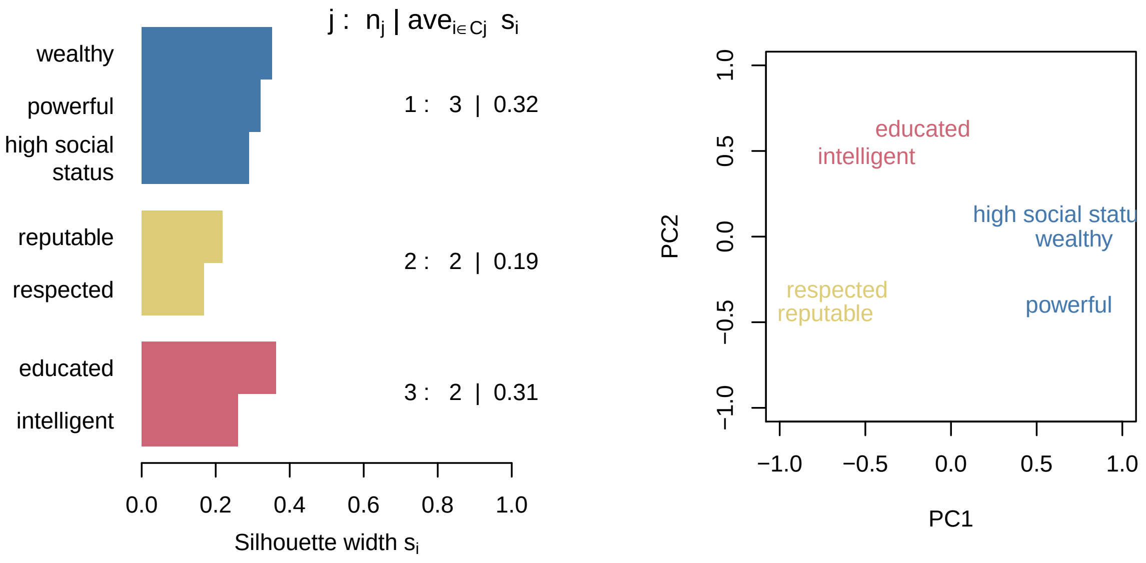

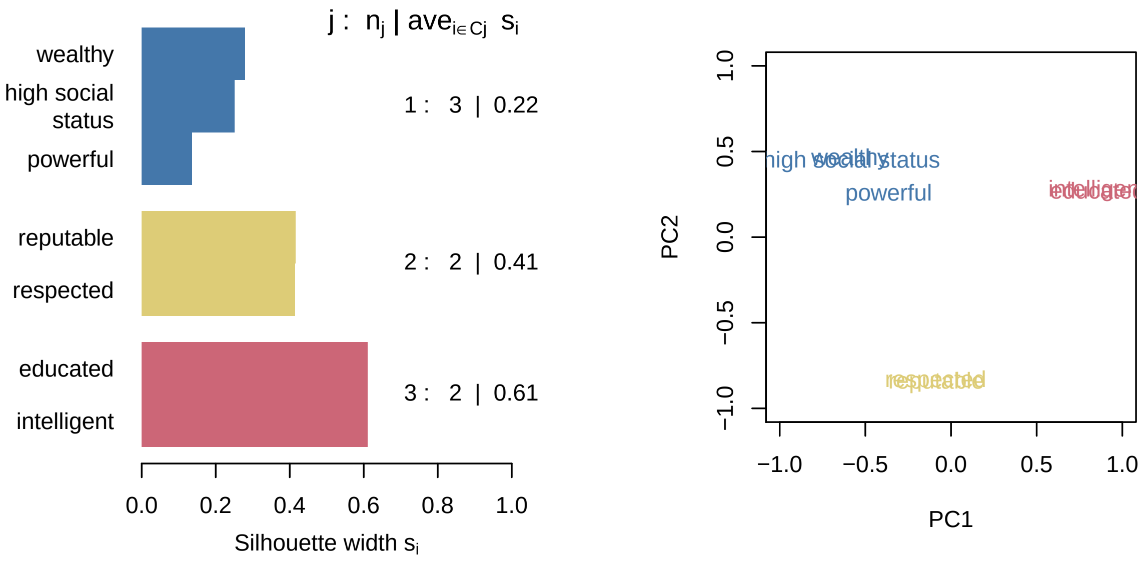


```
# dev.off()
```

STUDY 2: SCALE EVALUATION

Exploratory Data Analysis

```
#   Combine scale construction (data.s) and scale evaluation (data.c) data sets
data.all = bind_rows(cbind(study = "con", data.s),
                     cbind(study = "eval", data.c))
data.all = data.all[,c("study", colnames(data.c))]
data.all$study = factor(data.all$study, levels=c("con","eval"))
data.all$id = as.factor(data.all$id)
data.all$accent = factor(data.all$accent, levels=levels(data.c$accent))
data.all$age.o = ordered(data.all$age.o, levels=levels(data.c$age.o))
data.all$gender = factor(data.all$gender, levels=levels(data.c$gender))

#       Ordered
data.all.o = data.all
for (i in 3:26) {
    data.all.o[,i] = ordered(as.integer(unlist(data.all.o[,i])))
}

#       Long
data.all.l = gather(data.all, "item", "score", active:wealthy)
data.all.l$item = factor(data.all.l$item, levels=levels(data.c.l$item))

#       Pre-emptively remove data.c outliers (we find these outliers later,
#           in "Checking Assumptions" of Exploratory Factory Analysis)
data.all.to.remove = as.character(unique(data.c$id[c(80,738,839,1125,1184,1871)]))
data.all = data.all[!as.character(data.all$id) %in% data.all.to.remove,]
data.all.o = data.all.o[!as.character(data.all.o$id) %in% data.all.to.remove,]
data.all.l = data.all.l[!as.character(data.all.l$id) %in% data.all.to.remove,]
```

The three additional *prestige* variables are left off of these plots, since they did not appear in the original data.

```
#   Prestige items
data.all.exp.prestige = summarySE(data.all.l[data.all.l$item %in%
                                      levels(data.all.l$item)[1:11],],
                                  measurevar="score", groupvars=c("item","study"))
data.all.exp.prestige$item = factor(data.all.exp.prestige$item,
                                    levels=levels(data.all.l$item)[1:11])

s.exp.prestige = ggplot(data.all.l[data.all.l$item %in%
                                       levels(data.all.l$item)[1:11],],
                        aes(x=item, y=score, fill=study)) +
    geom_violin() +
    geom_jitter(size=0.4, alpha=0.1, height=0.1, show.legend=F) +
    geom_errorbar(data=data.all.exp.prestige, aes(ymin=score-ci, ymax=score+ci),
```

```
                  width=1, size=0.5, position=position_dodge(0.9)) +
    scale_fill_manual(values=c("#4477AA","#CC6677")) +
    coord_cartesian(ylim=c(1,7)) +
    ggtitle("'Prestige' Item Distributions") +
    theme(axis.text.x=element_text(angle=-90, hjust=0, vjust=0.3))
s.exp.prestige
```

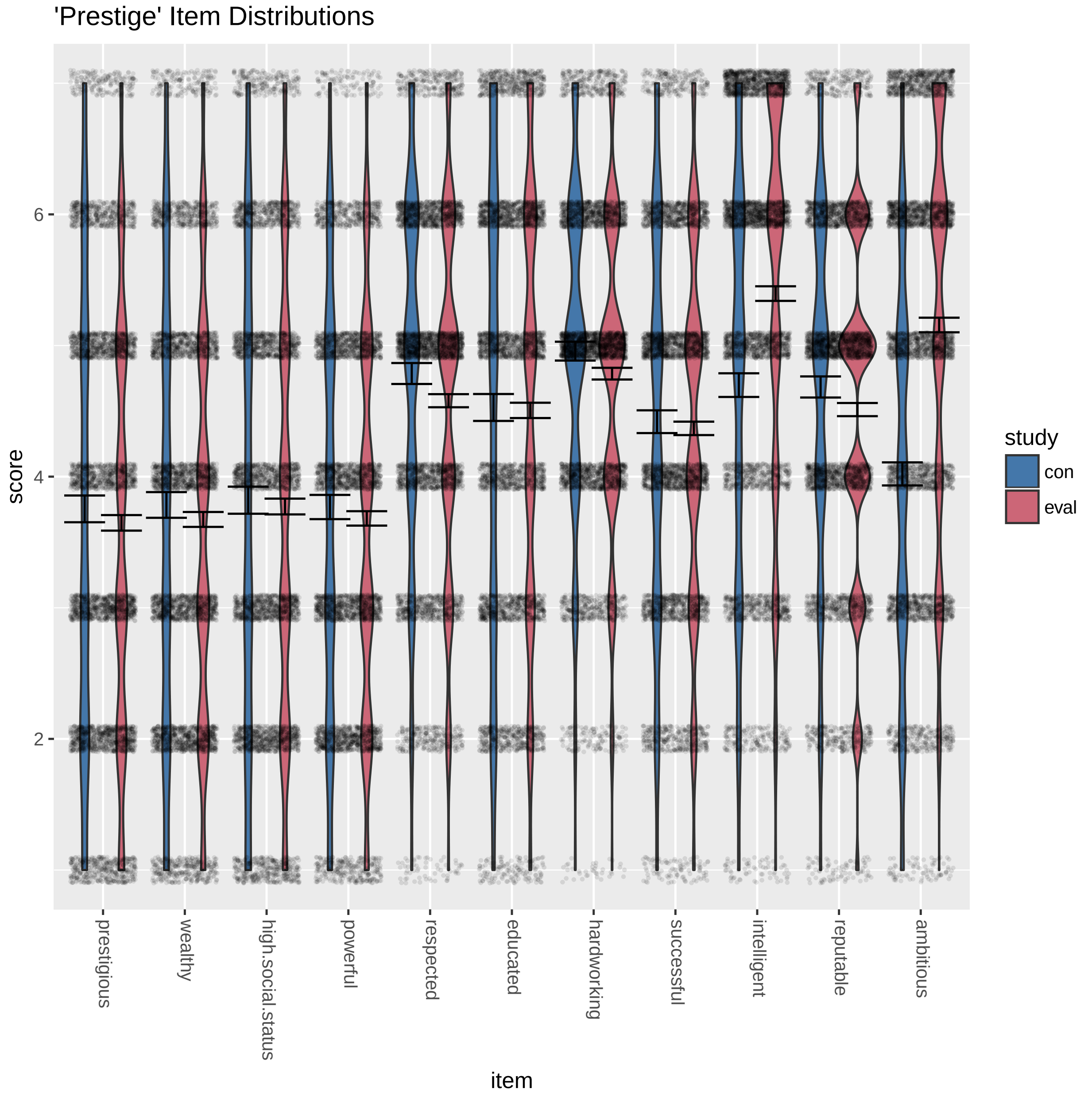


```
#   Solidarity items
data.all.exp.solidarity = summarySE(data.all.l[data.all.l$item %in%
                                          levels(data.all.l$item)[15:19],],
                              measurevar="score", groupvars=c("item","study"))
data.all.exp.solidarity$item = factor(data.all.exp.solidarity$item,
                              levels=levels(data.all.l$item)[15:19])
```

```
s.exp.solidarity = ggplot(data.all.l[data.all.l$item %in%
                                      levels(data.all.l$item)[15:19],],
                          aes(x=item, y=score, fill=study)) +
    geom_violin() +
    geom_jitter(size=0.4, alpha=0.1, height=0.1, show.legend=F) +
    geom_errorbar(data=data.all.exp.solidarity, aes(ymin=score-ci, ymax=score+ci),
                  width=1, size=0.5, position=position_dodge(0.9)) +
    scale_fill_manual(values=c("#4477AA","#CC6677")) +
    coord_cartesian(ylim=c(1,7)) +
    ggtitle("'Solidarity' Item Distributions") +
    theme(axis.text.x=element_text(angle=-90, hjust=0, vjust=0.3))
s.exp.solidarity
```

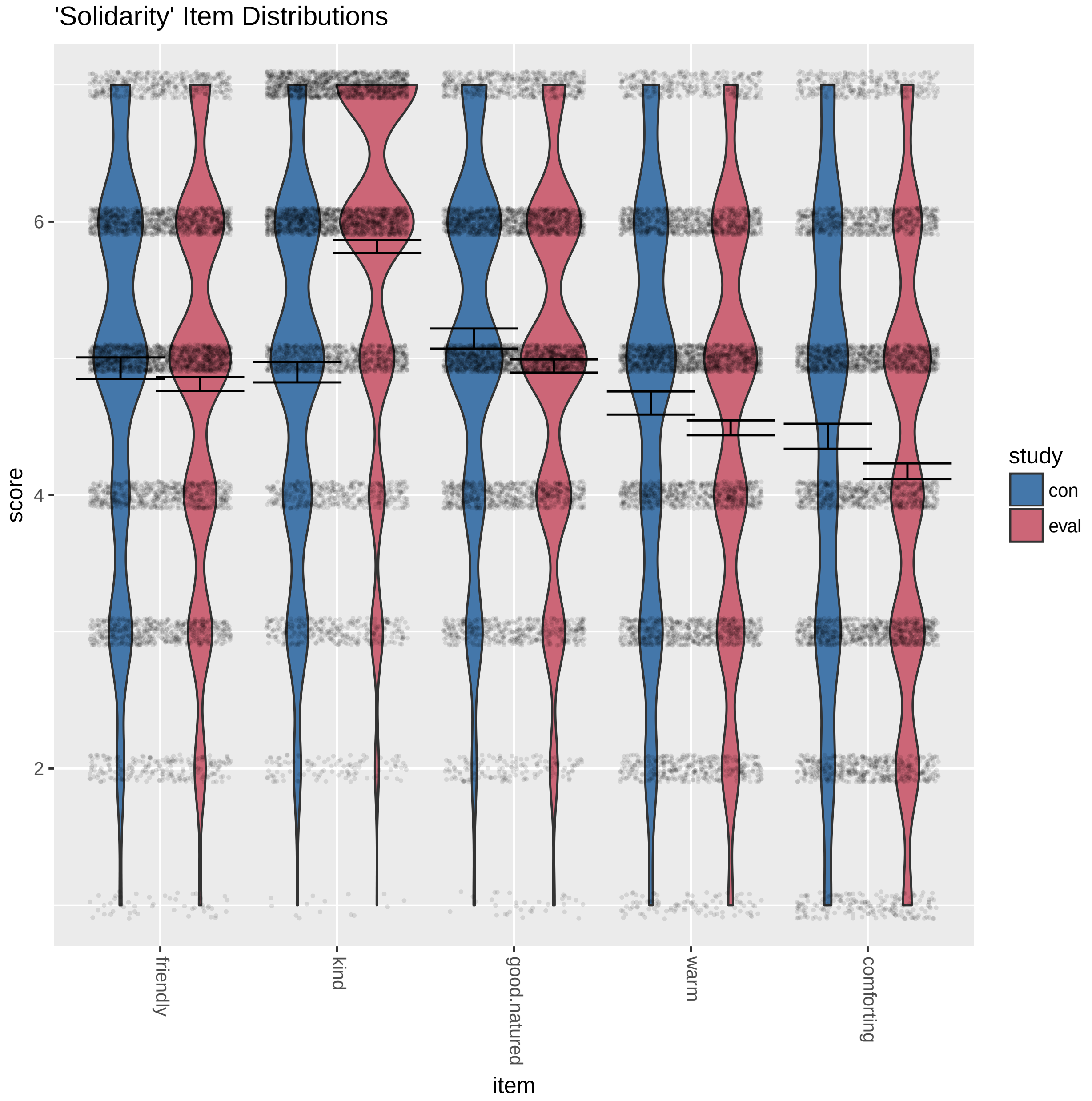


```
#   Dynamism items
data.all.exp.dynamism = summarySE(data.all.l[data.all.l$item %in%
                                              levels(data.all.l$item)[20:24],],
                                  measurevar="score", groupvars=c("item","study"))
data.all.exp.dynamism$item = factor(data.all.exp.dynamism$item,
                                    levels=levels(data.all.l$item)[20:24])

s.exp.dynamism = ggplot(data.all.l[data.all.l$item %in%
                                   levels(data.all.l$item)[20:24],],
                        aes(x=item, y=score, fill=study)) +
    geom_violin() +
    geom_jitter(size=0.4, alpha=0.1, height=0.1, show.legend=F) +
```

```
    geom_errorbar(data=data.all.exp.dynamism, aes(ymin=score-ci, ymax=score+ci),
                  width=1, size=0.5, position=position_dodge(0.9)) +
    scale_fill_manual(values=c("#4477AA","#CC6677")) +
    coord_cartesian(ylim=c(1,7)) +
    ggtitle("'Dynamism' Item Distributions") +
    theme(axis.text.x=element_text(angle=-90, hjust=0, vjust=0.3))
s.exp.dynamism
```

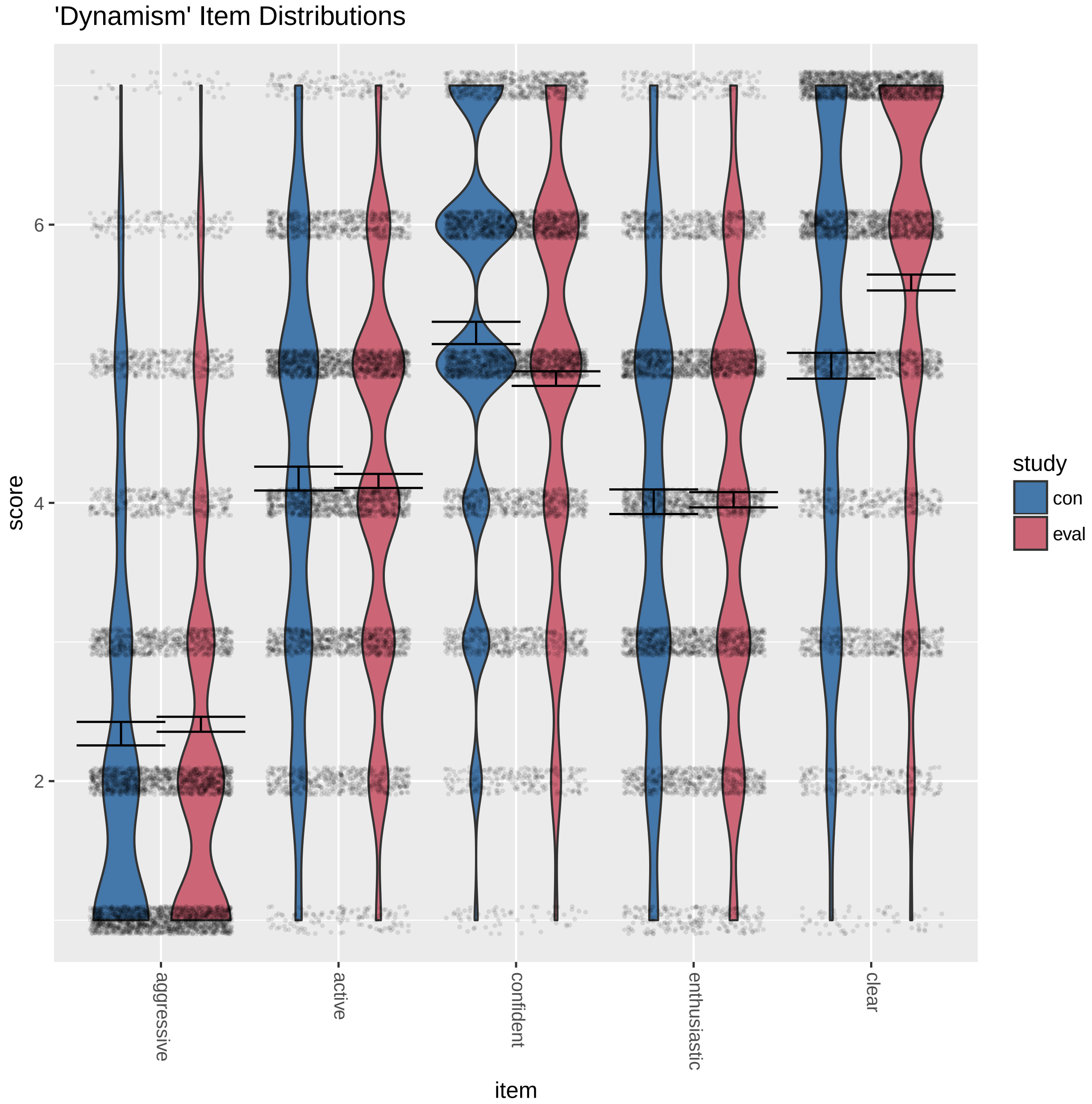


```
#   Permutation test of independence of attitudinal variables by study
c.att.ipt = independence_test(active+aggressive+ambitious+clear+comforting+
                              confident+educated+enthusiastic+friendly+
                              good.natured+hardworking+high.social.status+
                              intelligent+kind+powerful+prestigious+reputable+
```

```
                              respected+successful+warm+wealthy ~ study,
                              data=data.all.o)
c.att.ipt
```

```
##
##  Asymptotic General Independence Test
##
## data:  active (ordered), aggressive (ordered), ambitious (ordered), clear (ordered), comforti
## maxT = 20.428, p-value < 2.2e-16
## alternative hypothesis: two.sided
```

```
#   Pairwise permutation tests of attitudinal variables by study
c.att.ppt = data.frame(item=factor(), comparison=factor(), p.val=numeric())
for (i in 1:length(levels(data.s.l$item))) {
    temp.ppt = pairwisePermutationTest(as.formula(paste(levels(data.s.l$item)[i],
                                                         "~study")),
                                       data=data.all.o)
    temp = data.frame(item=levels(data.s.l$item)[i],
                      comparison=temp.ppt$Comparison,
                      p.val=as.numeric(temp.ppt$p.value))
    c.att.ppt = rbind(c.att.ppt, temp)
}
c.att.ppt$p.adj = p.adjust(c.att.ppt$p.val, "fdr")
c.att.ppt$sig = symnum(c.att.ppt$p.adj, cutpoints = c(0, 0.001, 0.01, 0.05, 1),
                       symbols = c("***", "**", "*", " "))
c.att.ppt$p.val = formatC(c.att.ppt$p.val, format="f", digits=4)
c.att.ppt$p.adj = formatC(c.att.ppt$p.adj, format="f", digits=4)

c.att.ppt
```

```
##                  item     comparison  p.val  p.adj sig
## 1         prestigious con - eval = 0 0.0642 0.0963
## 2             wealthy con - eval = 0 0.0460 0.0743
## 3   high.social.status con - eval = 0 0.4100 0.4783
## 4           powerful con - eval = 0 0.1016 0.1422
## 5          respected con - eval = 0 0.0000 0.0000 ***
## 6           educated con - eval = 0 0.6984 0.7651
## 7         hardworking con - eval = 0 0.0001 0.0001 ***
## 8          successful con - eval = 0 0.3053 0.3771
## 9         intelligent con - eval = 0 0.0000 0.0000 ***
## 10          reputable con - eval = 0 0.0003 0.0006 ***
## 11          ambitious con - eval = 0 0.0000 0.0000 ***
## 12           friendly con - eval = 0 0.0155 0.0272   *
## 13               kind con - eval = 0 0.0000 0.0000 ***
## 14        good.natured con - eval = 0 0.0000 0.0000 ***
## 15               warm con - eval = 0 0.0004 0.0008 ***
## 16          comforting con - eval = 0 0.0000 0.0000 ***
## 17          aggressive con - eval = 0 0.1846 0.2423
## 18              active con - eval = 0 0.7287 0.7651
```

```
## 19          confident con - eval = 0 0.0000 0.0000 ***
## 20       enthusiastic con - eval = 0 0.7874 0.7874
## 21              clear con - eval = 0 0.0000 0.0000 ***
```

```
#   Demographic data
#       Scale construction study in teal, scale construction study in orange
# pdf("figure7.pdf", width=8, height=8)

c.exp.country = ggplot(data.all[!duplicated(data.all[,2]),],
                       aes(x=country, fill=study)) +
    geom_bar(position=position_dodge()) +
    scale_fill_manual(values=c("#57A3AD","#DEA73A")) +
    ggtitle("Country") +
    xlab(NULL) +
    ylab(NULL) +
    guides(fill=F)

c.exp.age = ggplot(subset(data.all[!duplicated(data.all[,2]),], !is.na(age.o)),
                   aes(x=age.o, fill=study)) +
    geom_bar(position=position_dodge()) +
    scale_fill_manual(values=c("#57A3AD","#DEA73A")) +
    ggtitle("Age") +
    xlab(NULL) +
    ylab(NULL) +
    guides(fill=F) +
    theme(axis.text.x=element_text(angle=-90, hjust=0, vjust=0.3))

c.exp.gender = ggplot(subset(data.all[!duplicated(data.all[,2]),], !is.na(gender)),
                      aes(x=gender, fill=study)) +
    geom_bar(position=position_dodge()) +
    scale_fill_manual(values=c("#57A3AD","#DEA73A")) +
    ggtitle("Gender") +
    xlab(NULL) +
    ylab(NULL) +
    guides(fill=F)

c.exp.ethnicity = ggplot(subset(data.all[!duplicated(data.all[,2]),],
                                !is.na(ethnicity)),
                         aes(x=ethnicity, fill=study)) +
    geom_bar(position=position_dodge()) +
    scale_fill_manual(values=c("#57A3AD","#DEA73A")) +
    ggtitle("Ethnicity") +
    xlab(NULL) +
    ylab(NULL) +
    guides(fill=F)

c.exp.locality = ggplot(subset(data.all[!duplicated(data.all[,2]),],
                               !is.na(locality)),
```

```
                        aes(x=locality, fill=study)) +
    geom_bar(position=position_dodge()) +
    scale_fill_manual(values=c("#57A3AD","#DEA73A")) +
    ggtitle("Locality") +
    xlab(NULL) +
    ylab(NULL) +
    guides(fill=F)

c.exp.english = ggplot(subset(data.all[!duplicated(data.all[,2]),],
                              !is.na(english)),
                       aes(x=english, fill=study)) +
    geom_bar(position=position_dodge()) +
    scale_fill_manual(values=c("#57A3AD","#DEA73A")) +
    ggtitle("English") +
    xlab(NULL) +
    ylab(NULL) +
    guides(fill=F)

c.exp.education = ggplot(subset(data.all[!duplicated(data.all[,2]),],
                                !is.na(education)),
                         aes(x=education, fill=study)) +
    geom_bar(position=position_dodge()) +
    scale_fill_manual(values=c("#57A3AD","#DEA73A")) +
    ggtitle("Education") +
    xlab(NULL) +
    ylab(NULL) +
    guides(fill=F)

c.exp.occupation = ggplot(subset(data.all[!duplicated(data.all[,2]),],
                                 !is.na(occupation)),
                          aes(x=occupation, fill=study)) +
    geom_bar(position=position_dodge()) +
    scale_fill_manual(values=c("#57A3AD","#DEA73A")) +
    ggtitle("Occupation") +
    xlab(NULL) +
    ylab(NULL) +
    guides(fill=F)

c.exp.income = ggplot(subset(data.all[!duplicated(data.all[,2]),], !is.na(income)),
                      aes(x=income, fill=study)) +
    geom_bar(position=position_dodge()) +
    scale_fill_manual(values=c("#57A3AD","#DEA73A")) +
    ggtitle("Income") +
    xlab(NULL) +
    ylab(NULL) +
    guides(fill=F)

multiplot(c.exp.country, c.exp.age, c.exp.gender,
```

```
       c.exp.ethnicity, c.exp.locality, c.exp.english,
       c.exp.education, c.exp.occupation, c.exp.income,
       layout=matrix(1:9, nrow=3, byrow=T))
```

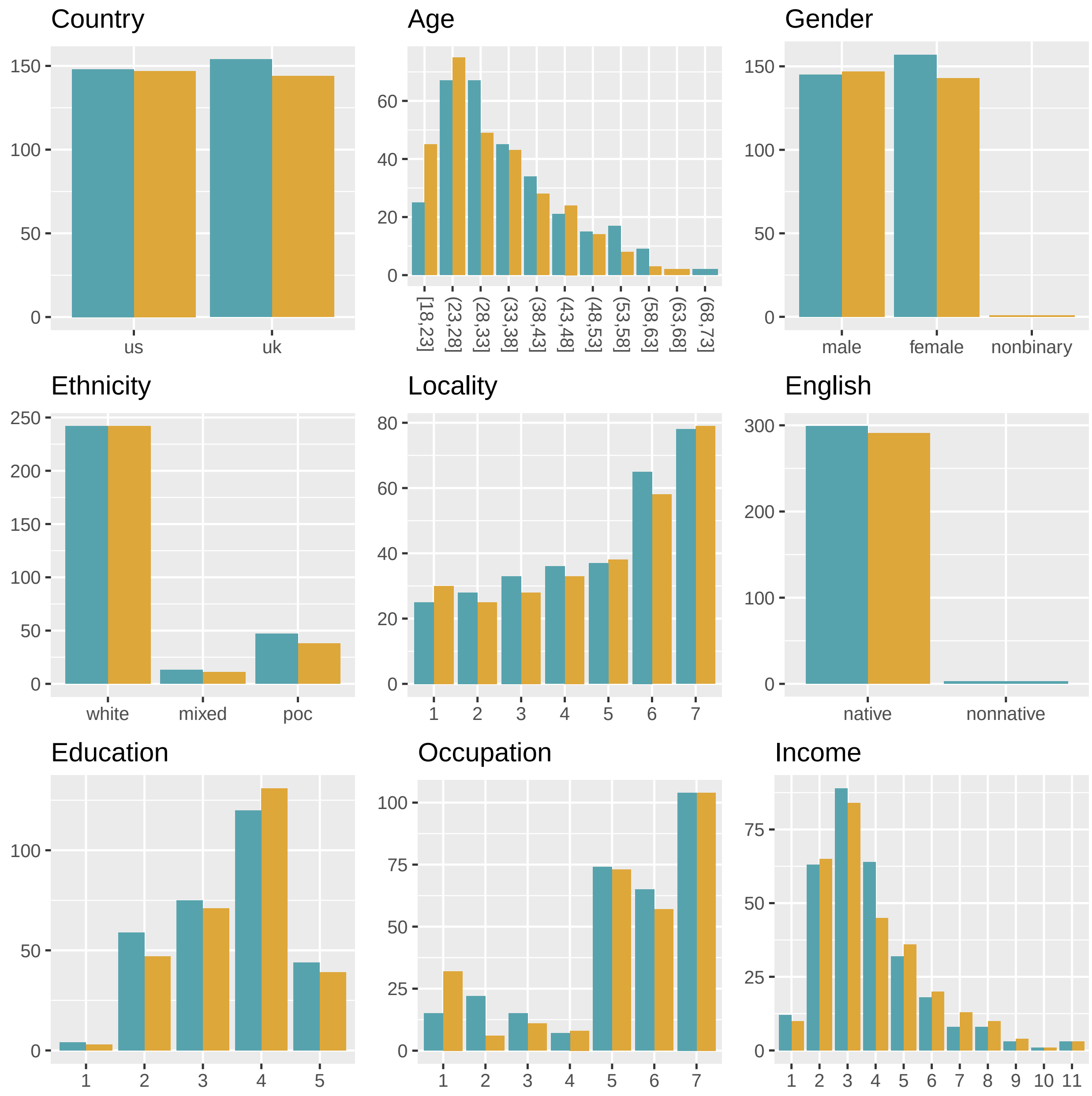


```
# dev.off()

#   Permutation test of independence of demographic variables by study
c.demo.ipt = independence_test(country+age.o+gender+ethnicity+locality+education+
                                   occupation+income ~ study,
                        data=data.all.o)
c.demo.ipt

##
##  Asymptotic General Independence Test
```

```
##
## data:  country, age.o (ordered), gender, ethnicity, locality (ordered), education (ordered),
## maxT = 8.2215, p-value = 5.995e-15
## alternative hypothesis: two.sided
```

```
#   Pairwise permutation tests of demographic variables by study
c.demo.ppt = data.frame(item=factor(), comparison=factor(), p.val=numeric())
for (i in 1:length(colnames(data.all)[c(28,30:33,35:37)])) {
    temp.ppt = pairwisePermutationTest(as.formula(paste(colnames(data.all)[c(28,30:33,35:37)][i]
                                                                   "~study")),
                                       data=data.all.o)
    temp = data.frame(item=colnames(data.all)[c(28,30:33,35:37)][i],
                      comparison=temp.ppt$Comparison,
                      p.val=as.numeric(temp.ppt$p.value))
    c.demo.ppt = rbind(c.demo.ppt, temp)
}
c.demo.ppt$p.adj = p.adjust(c.demo.ppt$p.val, "fdr")
c.demo.ppt$sig = symnum(c.demo.ppt$p.adj, cutpoints = c(0, 0.001, 0.01, 0.05, 1),
                       symbols = c("***", "**", "*", " "))
c.demo.ppt$p.val = formatC(c.demo.ppt$p.val, format="f", digits=4)
c.demo.ppt$p.adj = formatC(c.demo.ppt$p.adj, format="f", digits=4)

c.demo.ppt
```

```
##          item     comparison  p.val  p.adj sig
## 1    country con - eval = 0 0.3780 0.4320
## 2      age.o con - eval = 0 0.0000 0.0000 ***
## 3     gender con - eval = 0 0.0844 0.1126
## 4  ethnicity con - eval = 0 0.0491 0.0785
## 5   locality con - eval = 0 0.7897 0.7897
## 6  education con - eval = 0 0.0365 0.0731
## 7 occupation con - eval = 0 0.0000 0.0000 ***
## 8     income con - eval = 0 0.0349 0.0731
```

Exploratory Factor Analysis

Checking Assumptions

```
#   Check for multivariate normality
mardiaTest(data.c[,c(25,17,14,19,20,9,15)], qqplot=T)
```

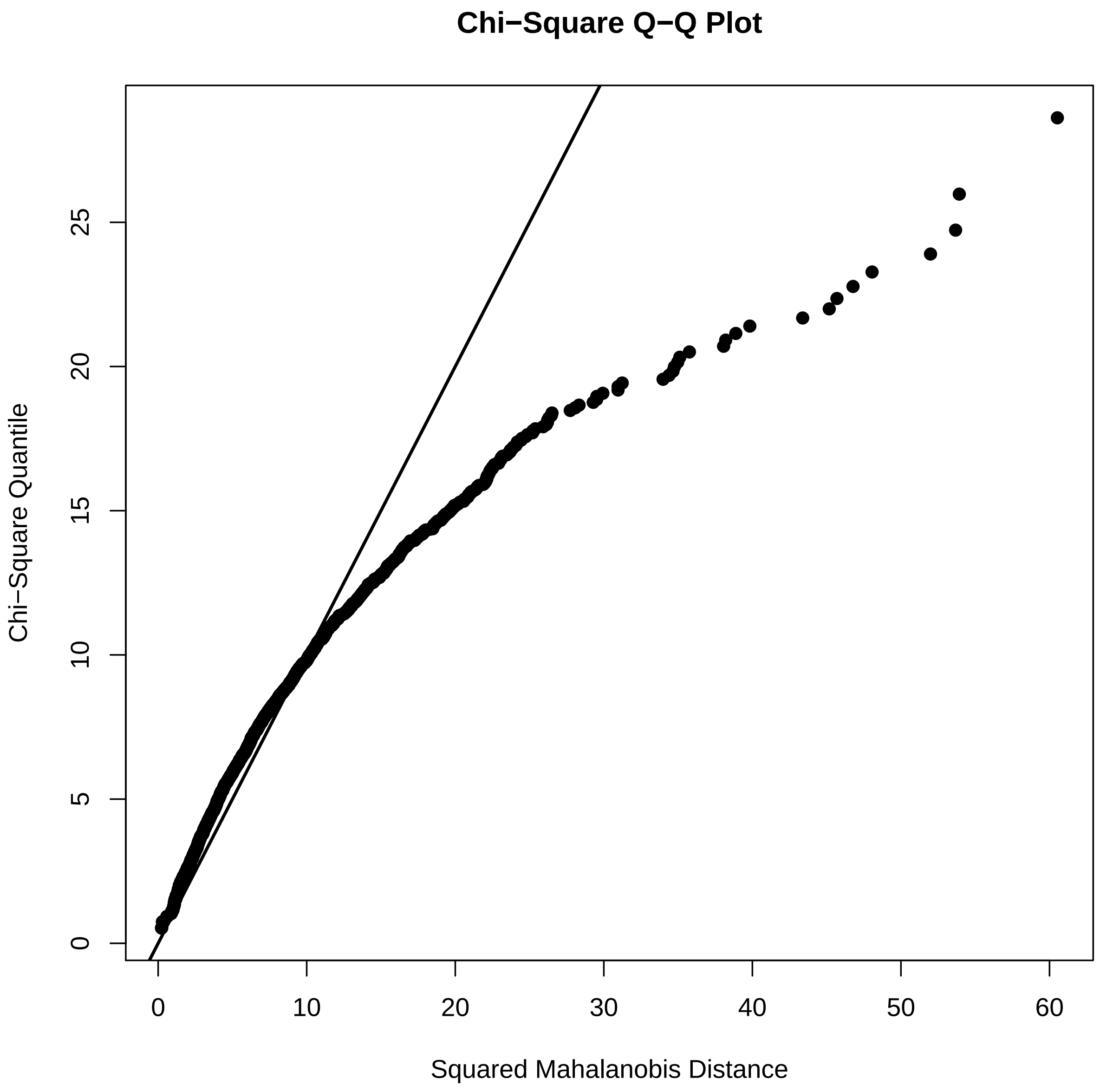


```
##    Mardia's Multivariate Normality Test
## ---------------------------------------
##    data : data.c[, c(25, 17, 14, 19, 20, 9, 15)]
##
##    g1p            : 2.444025
##    chi.skew       : 1201.645
##    p.value.skew   : 3.168192e-197
##
##    g2p            : 81.29621
##    z.kurtosis     : 44.26463
##    p.value.kurt   : 0
##
```

```
##    chi.small.skew : 1203.173
##    p.value.small  : 1.55467e-197
##
##    Result          : Data are not multivariate normal.
## ---------------------------------------
```

```
hzTest(data.c[,c(25,17,14,19,20,9,15)], qqplot=F)
```

```
##   Henze-Zirkler's Multivariate Normality Test
## ---------------------------------------------
##   data : data.c[, c(25, 17, 14, 19, 20, 9, 15)]
##
##   HZ      : 4.266756
##   p-value : 0
##
##   Result  : Data are not multivariate normal.
## ---------------------------------------------
```

```
# roystonTest(data.c[,c(25,17,14,19,20,9,15)], qqplot=F)    # Too many observations

#   Check for multivariate outliers
head(mvOutlier(data.c[,c(25,17,14,19,20,9,15)], qqplot=T)$outlier, 10)
```

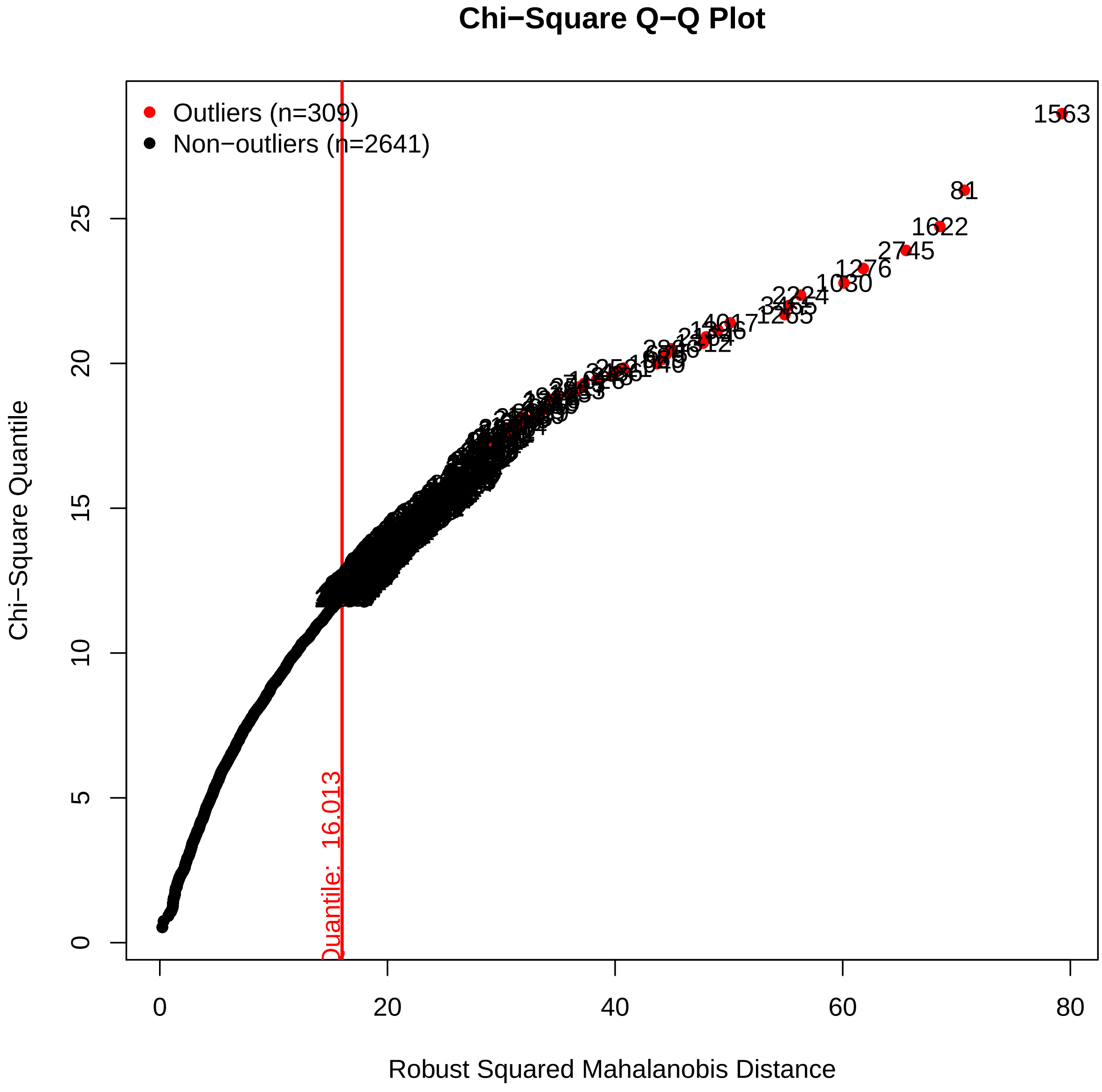


```
##      Observation Mahalanobis Distance Outlier
## 1563        1563               79.263    TRUE
## 81            81               70.685    TRUE
## 1622        1622               68.536    TRUE
## 2745        2745               65.574    TRUE
## 1276        1276               61.804    TRUE
## 1030        1030               60.113    TRUE
## 2224        2224               56.292    TRUE
## 3465        3465               55.266    TRUE
## 1265        1265               54.916    TRUE
## 4017        4017               50.107    TRUE
```

```
#       Loop to find rows of outlier observations for removal (warning: LONG,
```

```
#           uncomment to run)
# to.remove = as.numeric(as.character(mvOutlier(data.c[,c(25,17,14,19,20,9,15)],
#                                                qqplot=T)$outlier[1:6,1]))
# lines.to.remove = c()
# lines.progress = txtProgressBar(min=0, max=nrow(data.c), style=3, width=100)
# for (i in 1:nrow(data.c)) {
# x = as.numeric(as.character(mvOutlier(data.c[-i,c(25,17,14,19,20,9,15)],
#                                        qqplot=F)$outlier[1:10,1]))
#     if (!all(to.remove %in% x)) {
#         lines.to.remove = append(lines.to.remove, i)
#     }
#     setTxtProgressBar(lines.progress, i)
# }
# close(lines.progress)
# lines.to.remove

#       Result: 80  738  839 1125 1184 1871

#       Check with results removed
head(mvOutlier(data.c[-c(80,738,839,1125,1184,1871),c(25,17,14,19,20,9,15)],
               qqplot=T)$outlier, 10)
```

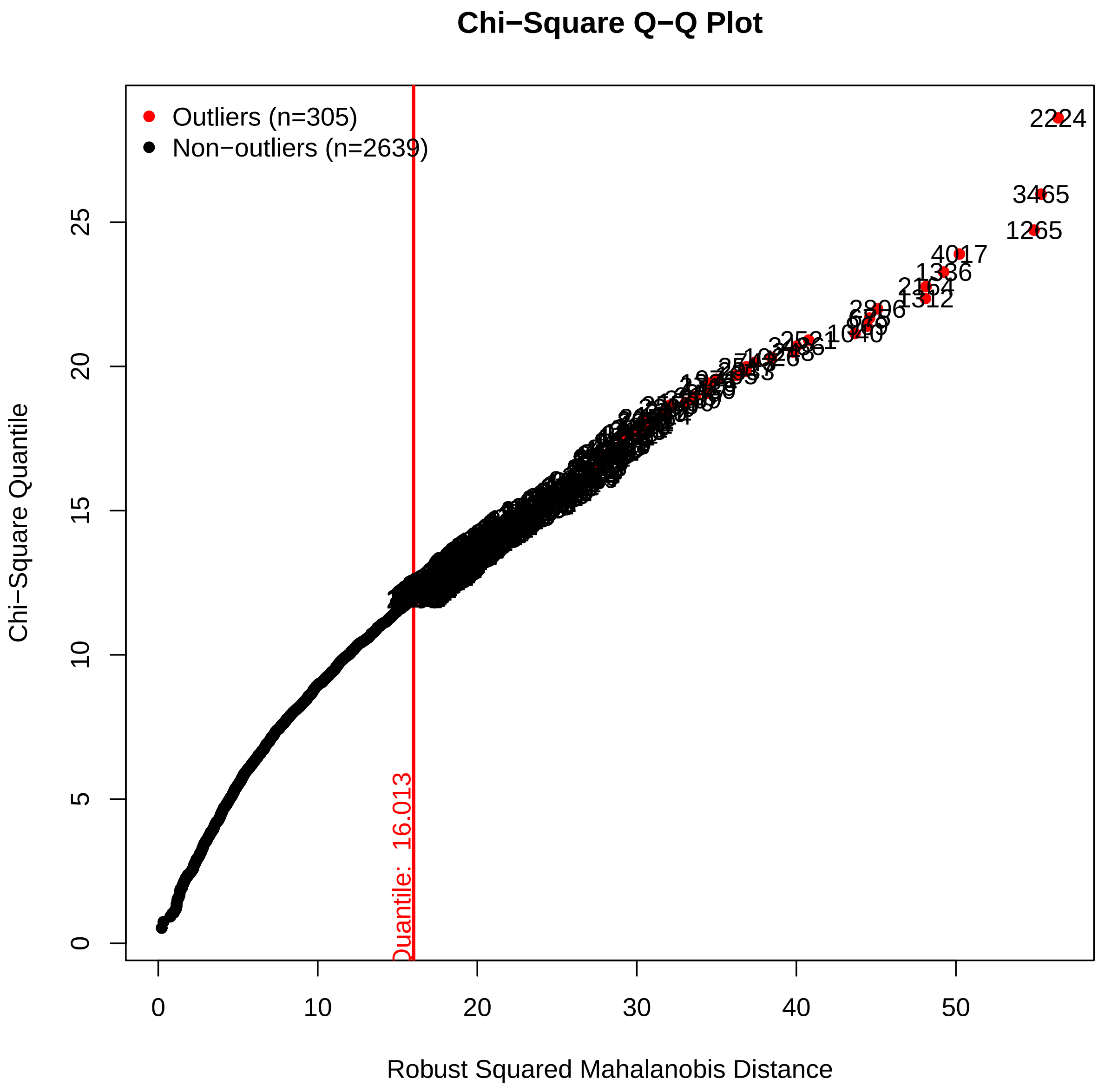


```
##      Observation Mahalanobis Distance Outlier
## 2224        2224               56.407    TRUE
## 3465        3465               55.336    TRUE
## 1265        1265               54.898    TRUE
## 4017        4017               50.219    TRUE
## 1336        1336               49.236    TRUE
## 2164        2164               48.136    TRUE
## 1312        1312               48.090    TRUE
## 2806        2806               45.091    TRUE
## 675          675               44.586    TRUE
## 969          969               44.430    TRUE

#       Identify participants
```

```r
data.c[c(80,738,839,1125,1184,1871),]
```

```
##              id active aggressive ambitious clear comforting confident
## 81   4803513020      6          4         6     3          3         7
## 1030 4803481471      2          3         2     7          5         4
## 1276 4803502390      1          1         7     7          1         1
## 1563 4803510410      1          7         1     7          1         7
## 1622 4803481471      3          6         1     3          6         2
## 2745 4803513020      5          5         6     5          2         1
##      driven educated enthusiastic friendly good.natured hardworking
## 81        2        1            3        2            1           6
## 1030      5        6            2        6            3           2
## 1276      1        1            1        4            4           7
## 1563      4        4            7        4            1           1
## 1622      5        3            5        2            6           6
## 2745      7        4            3        2            5           5
##      high.social.status intelligent kind powerful prestigious reputable
## 81                    6           7    5        2           6         4
## 1030                  2           3    2        7           4         6
## 1276                  1           7    7        4           1         1
## 1563                  1           1    1        7           7         4
## 1622                  1           2    4        2           5         5
## 2745                  6           1    3        2           7         2
##      respected skilled successful talented warm wealthy          accent
## 81           7       5          6        5    3       1        colorado
## 1030         2       4          6        4    3       6      illinois10
## 1276         7       6          1        1    1       1 northcarolina14
## 1563         7       1          7        1    1       7 northcarolina10
## 1622         7       4          3        6    4       7 northcarolina10
## 2745         6       2          4        3    2       1        england60
##      country age   age.o gender ethnicity locality english education
## 81        us  27 (23,28] female     mixed        5  native         2
## 1030      us  32 (28,33] female       poc        6  native         3
## 1276      us  26 (23,28]   male       poc        4  native         3
## 1563      us  28 (23,28] female     white        5  native         3
## 1622      us  32 (28,33] female       poc        6  native         3
## 2745      us  27 (23,28] female     mixed        5  native         2
##      occupation income
## 81            6      4
## 1030          2      2
## 1276          1      2
## 1563          5      4
## 1622          2      2
## 2745          6      4
```

```r
data.c.to.remove = as.character(unique(data.c$id[c(80,738,839,1125,1184,1871)]))

#       Remove participants
data.c = data.c[!as.character(data.c$id) %in% data.c.to.remove,]
```

```r
data.c.o = data.c.o[!as.character(data.c.o$id) %in% data.c.to.remove,]
data.c.l = data.c.l[!as.character(data.c.l$id) %in% data.c.to.remove,]

#   Check for univariate normality
uniPlot(data.c[,c(25,17,14,19,20,9,15)], "histogram")

#   Check skewness
sort(apply(data.c[,c(25,17,14,19,20,9,15)], 2, e1071::skewness), decreasing=T)
```

```
##             wealthy            powerful high.social.status
##           0.1738016           0.1278680          0.1232289
##            educated           respected          reputable
##          -0.2910235          -0.3570799         -0.4149637
##         intelligent
##          -0.8228329
```

```r
#   Check kurtosis
sort(apply(data.c[,c(25,17,14,19,20,9,15)], 2, e1071::kurtosis, type=2),
     decreasing=T)
```

```
##         intelligent           reputable          respected
##          -0.2175272          -0.3506247         -0.4292984
##            powerful             wealthy           educated
##          -0.7678839          -0.7901175         -0.8330473
## high.social.status
##          -0.9337776
```

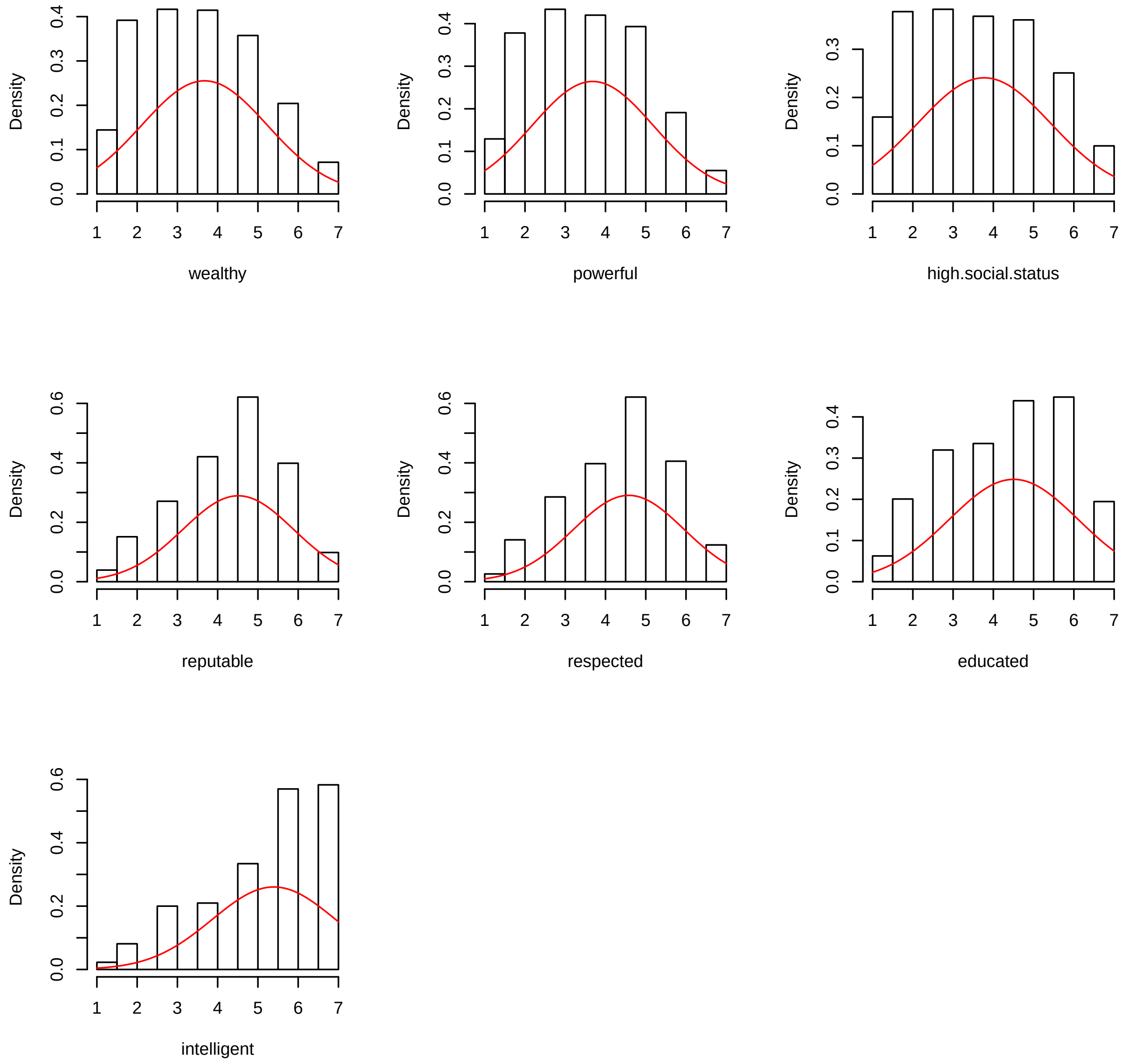


```
#   Check for linear relationships and correlations
corrplot.mixed(lavCor(data.c[,c(25,17,14,19,20,9,15)]), lower="ellipse",
               upper="number", order="hclust", hclust.method="ward.D",
               tl.cex=0.7, tl.pos="d", tl.col="black")
```

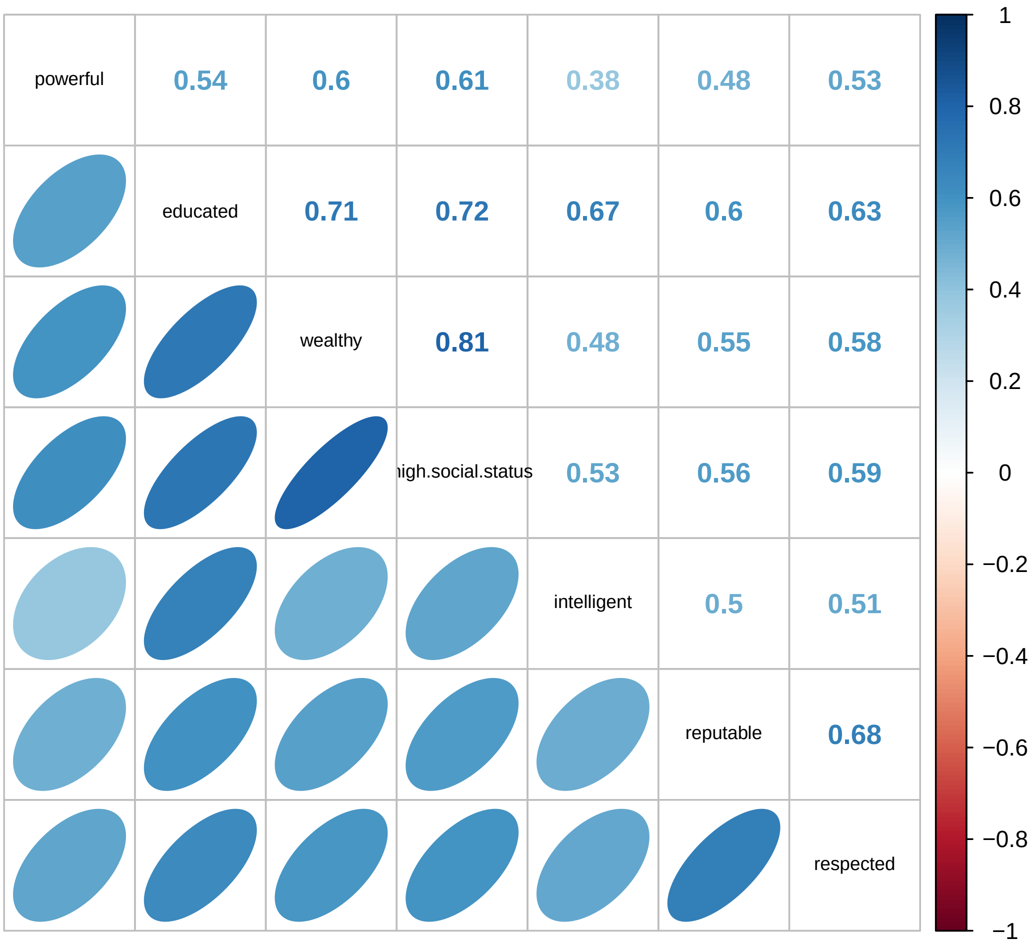


```
#   Check factorability
KMO(lavCor(data.c.o[,c(25,17,14,19,20,9,15)]))
```

```
## Kaiser-Meyer-Olkin factor adequacy
## Call: KMO(r = lavCor(data.c.o[, c(25, 17, 14, 19, 20, 9, 15)]))
## Overall MSA =  0.89
## MSA for each item =
##            wealthy           powerful high.social.status
##               0.87               0.95               0.87
##          reputable          respected           educated
##               0.90               0.90               0.89
##        intelligent
##               0.88
```

```r
cortest.bartlett(lavCor(data.c.o[,c(25,17,14,19,20,9,15)]), nrow(data.c.o) / 4)
```

```
## $chisq
## [1] 3645.93
##
## $p.value
## [1] 0
##
## $df
## [1] 21
```

```r
#   Sample size
length(unique(data.c$id))
```

```
## [1] 291
```

```r
#       Minimum
ncol(data.c[,c(25,17,14,19,20,9,15)]) *
    (ncol(data.c[,c(25,17,14,19,20,9,15)]) - 1) / 2
```

```
## [1] 21
```

```r
#       Subjects-to-variables ratio
length(unique(data.c$id)) / ncol(data.c[,c(25,17,14,19,20,9,15)])
```

```
## [1] 41.57143
```

Exploratory Factor Analysis

```r
#   Determine number of factors
EFA.Comp.Data(data.c[,c(2:4,6:25)], F.Max=10, Graph=T, Spearman=T)
```

```
## Number of factors to retain:  5
```

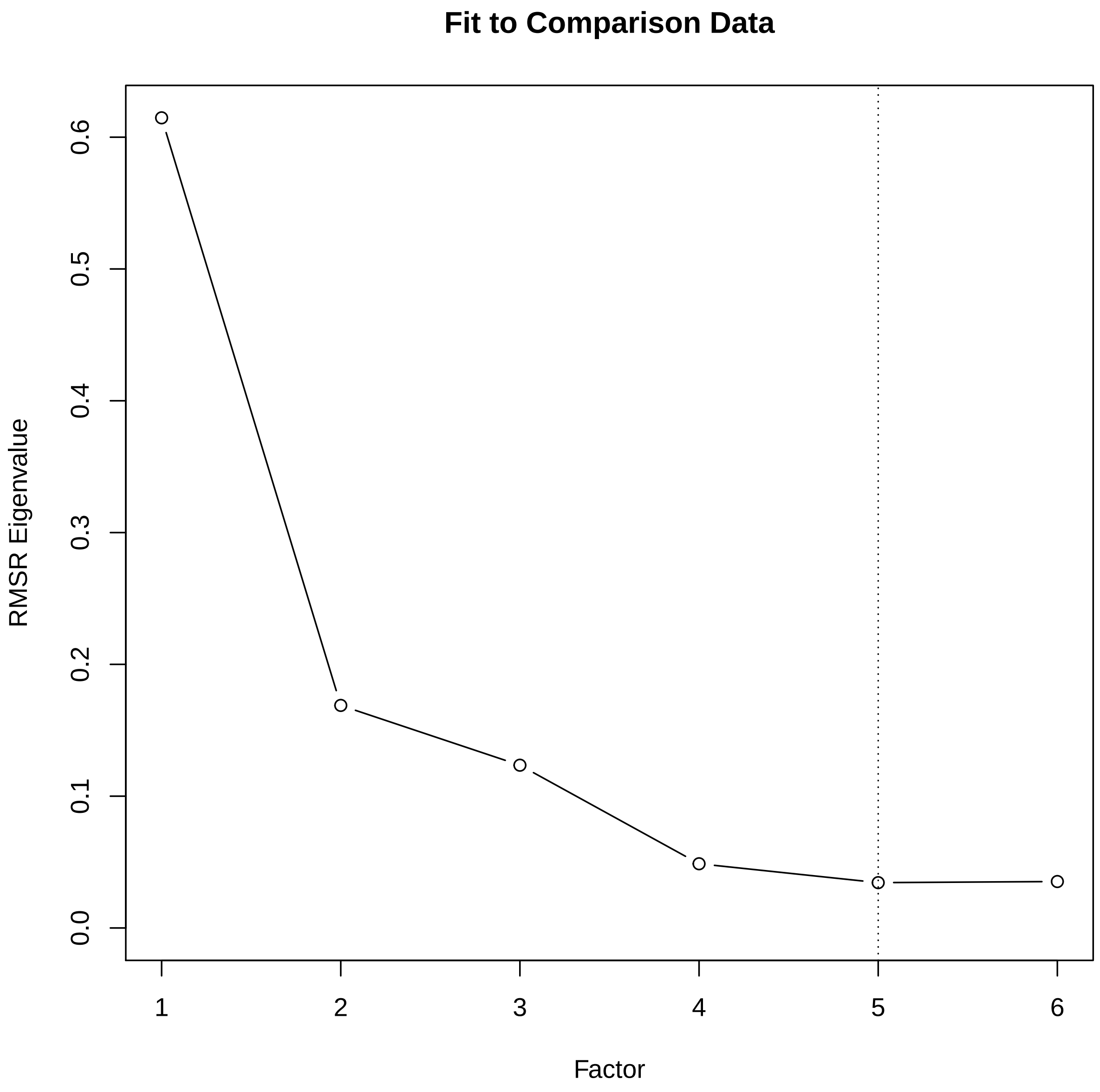

Possible overdimensionalization.

```
#   Run EFA with 5 factors
c.ea = efaUnrotate(data.c.o[,c(2:4,6:25)], 5, estimator="WLSMV")
c.eao = oblqRotate(c.ea, method="oblimin")
c.eao
```

```
## Standardized Rotated Factor Loadings
##                     factor1 factor2 factor3 factor4 factor5
## high.social.status  0.918*
## wealthy             0.916*
## prestigious         0.864*
## educated            0.612*                   0.168* -0.294*
```

```
## successful          0.597*          0.134*  0.239* -0.139*
## powerful            0.496*          0.295*  0.172*
## talented            0.416*  0.196*  0.231*  0.246*
## warm                        0.911*
## friendly                    0.858*
## comforting          0.160*  0.823*
## good.natured       -0.113*  0.793*          0.127*
## aggressive                 -0.471*  0.382*  0.121*  0.396*
## active                              0.676*
## enthusiastic        0.153*  0.423*  0.516* -0.150*
## driven              0.206*          0.514*  0.183* -0.122*
## confident           0.211*          0.404*  0.209* -0.171*
## respected           0.414*  0.171*          0.497*
## hardworking        -0.287*  0.205*  0.301*  0.467*
## reputable           0.390*  0.178*          0.454*
## skilled             0.267*  0.116*  0.224*  0.422*
## intelligent         0.272* -0.120*          0.153* -0.673*
## ambitious           0.157* -0.114*  0.317*         -0.590*
## kind               -0.217*  0.477* -0.105*         -0.568*
##
## Factor Correlation
##             factor1     factor2     factor3    factor4    factor5
## factor1  1.00000000  0.04463605  0.45659361  0.5371285 -0.3428777
## factor2  0.04463605  1.00000000  0.08022421  0.2724055 -0.3206624
## factor3  0.45659361  0.08022421  1.00000000  0.4814221 -0.1341422
## factor4  0.53712849  0.27240553  0.48142206  1.0000000 -0.3126912
## factor5 -0.34287767 -0.32066243 -0.13414219 -0.3126912  1.0000000
##
## Method of rotation:  Oblimin Quartimin
## [1] "The standard errors are close but do not match with other packages. Be mindful when usin
```

```
inspect(c.ea, "rsquare")    # Communalities (r^2)
```

```
##              active          aggressive           ambitious
##               0.515               0.567               0.609
##          comforting           confident              driven
##               0.677               0.567               0.618
##            educated        enthusiastic            friendly
##               0.781               0.495               0.790
##        good.natured         hardworking high.social.status
##               0.720               0.430               0.845
##         intelligent                kind            powerful
##               0.767               0.682               0.598
##         prestigious           reputable           respected
##               0.787               0.661               0.736
##             skilled          successful            talented
##               0.669               0.793               0.663
##                warm             wealthy
##               0.804               0.822
```

```r
# summary(c.eao)   # Uncomment to view test statistics (se, p, ci)
# fitMeasures(c.ea, c("chisq","df","pvalue","cfi","tli","rmsea","srmr"))
#       Uncomment to view listed fit measures

#   Run EFA with 4 factors
c.ea2 = efaUnrotate(data.c.o[,c(2:4,6:25)], 4, estimator="WLSMV")
c.eao2 = oblqRotate(c.ea2, method="oblimin")
c.eao2
```

```
## Standardized Rotated Factor Loadings
##                    factor1 factor2 factor3 factor4
## high.social.status  0.951*
## wealthy             0.950*
## prestigious         0.913*
## educated            0.685*          0.300*
## successful          0.683*          0.149* -0.211*
## respected           0.569*  0.268*  0.108* -0.190*
## powerful            0.552*                 -0.352*
## reputable           0.535*  0.266*         -0.190*
## talented            0.496*  0.230*         -0.316*
## warm                        0.911*
## friendly                    0.871*
## comforting          0.178*  0.833*
## good.natured                0.819*
## intelligent         0.329* -0.118*  0.694*
## kind               -0.219*  0.469*  0.576*  0.136*
## ambitious           0.165* -0.133*  0.573* -0.291*
## active                                     -0.682*
## driven              0.244*          0.124* -0.586*
## hardworking        -0.142*  0.301*  0.113* -0.507*
## confident           0.261*          0.180* -0.486*
## aggressive                 -0.445* -0.393* -0.473*
## enthusiastic                0.370*         -0.411*
## skilled             0.399*  0.195*  0.116* -0.401*
##
## Factor Correlation
##             factor1     factor2    factor3    factor4
## factor1  1.00000000  0.09225484  0.3740807 -0.5418862
## factor2  0.09225484  1.00000000  0.3522282 -0.1276881
## factor3  0.37408070  0.35222817  1.0000000 -0.1976308
## factor4 -0.54188619 -0.12768813 -0.1976308  1.0000000
##
## Method of rotation:  Oblimin Quartimin
## [1] "The standard errors are close but do not match with other packages. Be mindful when usin
```

```r
inspect(c.ea2, "rsquare")    # Communalities (r^2)
```

```
##             active         aggressive          ambitious
##              0.479              0.569              0.580
##         comforting          confident             driven
```

```
##              0.678              0.567              0.616
##          educated      enthusiastic          friendly
##              0.781              0.409              0.791
##       good.natured        hardworking high.social.status
##              0.716              0.368              0.838
##        intelligent               kind          powerful
##              0.774              0.681              0.598
##        prestigious          reputable         respected
##              0.785              0.618              0.676
##            skilled         successful          talented
##              0.649              0.791              0.663
##               warm            wealthy
##              0.799              0.818
```

```
# summary(c.eao2)   # Uncomment to view test statistics (se, p, ci)
# fitMeasures(c.ea2, c("chisq","df","pvalue","cfi","tli","rmsea","srmr"))
#       Uncomment to view listed fit measures
```

```
#   Remove 'aggressive' and repeat
EFA.Comp.Data(data.c[,c(2,4,6:25)], F.Max=10, Graph=T, Spearman=T)
```

```
## Number of factors to retain:  6
```

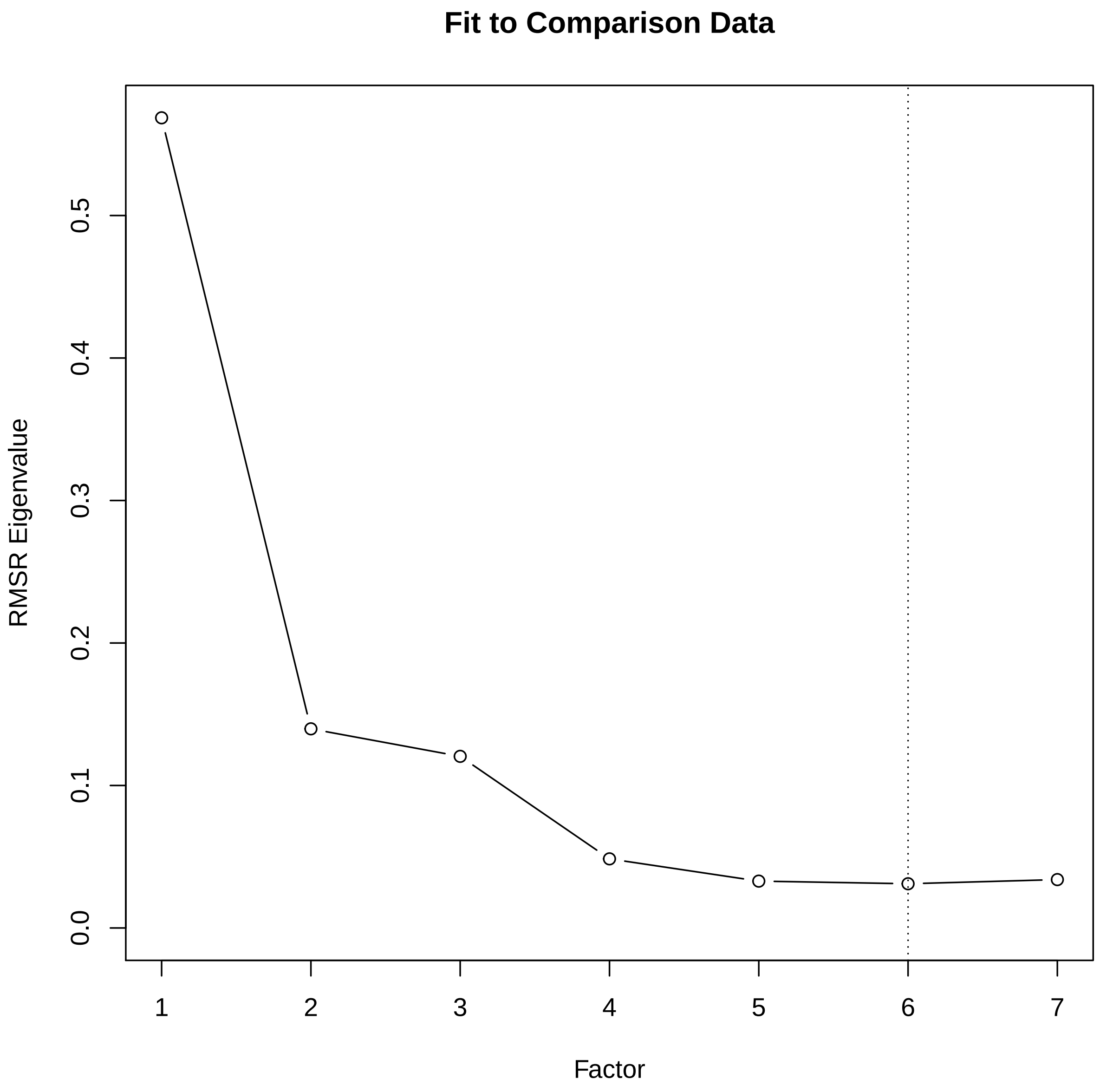

Likely overdimensionalization.

```
#   Run EFA with 5 factors
c.ea3 = efaUnrotate(data.c.o[,c(2,4,6:25)], 5, estimator="WLSMV")
c.eao3 = oblqRotate(c.ea3, method="oblimin")
c.eao3
```

```
## Standardized Rotated Factor Loadings
##                    factor1 factor2 factor3 factor4 factor5
## high.social.status  0.894*
## wealthy             0.890*
## prestigious         0.842*
## educated            0.580*          0.143*          -0.325*
```

```
## successful          0.562*          0.227* -0.160* -0.160*
## powerful            0.472*          0.163* -0.321*
## talented            0.385*  0.173*  0.234* -0.268*
## warm                        0.909*
## friendly                    0.864*
## comforting          0.170*  0.826*
## good.natured       -0.111*  0.797*  0.106*
## kind               -0.232*  0.542*          0.123* -0.513*
## respected           0.393*  0.173*  0.479*
## hardworking        -0.312*  0.167*  0.443* -0.355*
## reputable           0.370*  0.178*  0.437*
## skilled             0.235*          0.401* -0.269*
## active                                     -0.723*
## driven              0.163*          0.161* -0.560* -0.112*
## enthusiastic        0.133*  0.386* -0.169* -0.530*
## confident           0.177*          0.174* -0.429* -0.180*
## intelligent         0.224*                         -0.749*
## ambitious           0.111*                 -0.301* -0.588*
##
## Factor Correlation
##            factor1    factor2    factor3    factor4    factor5
## factor1  1.0000000  0.0341752  0.5095735 -0.4970526 -0.4128210
## factor2  0.0341752  1.0000000  0.3025706 -0.2053081 -0.2546777
## factor3  0.5095735  0.3025706  1.0000000 -0.4898072 -0.3780643
## factor4 -0.4970526 -0.2053081 -0.4898072  1.0000000  0.3094205
## factor5 -0.4128210 -0.2546777 -0.3780643  0.3094205  1.0000000
##
## Method of rotation:  Oblimin Quartimin
## [1] "The standard errors are close but do not match with other packages. Be mindful when usin
```

```
inspect(c.ea3, "rsquare")    # Communalities (r^2)
```

```
##              active          ambitious         comforting
##               0.519              0.598              0.678
##           confident             driven           educated
##               0.565              0.623              0.781
##        enthusiastic           friendly       good.natured
##               0.497              0.794              0.720
##         hardworking high.social.status        intelligent
##               0.438              0.845              0.813
##                kind           powerful        prestigious
##               0.610              0.585              0.787
##           reputable          respected            skilled
##               0.660              0.736              0.671
##          successful           talented               warm
##               0.793              0.664              0.804
##             wealthy
##               0.821
```

```r
# summary(c.eao3)   # Uncomment to view test statistics (se, p, ci)
# fitMeasures(c.ea3, c("chisq","df","pvalue","cfi","tli","rmsea","srmr"))
#       Uncomment to view listed fit measures

#   Run EFA with 4 factors
c.ea4 = efaUnrotate(data.c.o[,c(2,4,6:25)], 4, estimator="WLSMV")
c.eao4 = oblqRotate(c.ea4, method="oblimin")
c.eao4
```

```
## Standardized Rotated Factor Loadings
##                    factor1 factor2 factor3 factor4
## high.social.status  0.928*
## wealthy             0.928*
## prestigious         0.890*
## educated            0.641*          0.320*
## successful          0.635*          0.159* -0.240*
## respected           0.533*  0.263*  0.122* -0.204*
## powerful            0.512*                 -0.382*
## reputable           0.500*  0.259*  0.111* -0.205*
## talented            0.449*  0.200*         -0.358*
## warm                        0.905*
## friendly                    0.871*
## comforting          0.191*  0.836*
## good.natured                0.818*
## kind               -0.235*  0.538*  0.514*  0.137*
## intelligent         0.259*          0.761*
## ambitious                  -0.105*  0.554* -0.295*
## active                                     -0.752*
## driven              0.174*                 -0.651*
## hardworking        -0.187*  0.250*         -0.545*
## confident           0.202*          0.168* -0.518*
## enthusiastic                0.323*         -0.456*
## skilled             0.346*  0.161*  0.108* -0.437*
##
## Factor Correlation
##             factor1     factor2    factor3    factor4
## factor1  1.00000000  0.07310025  0.4281994 -0.5914088
## factor2  0.07310025  1.00000000  0.2720342 -0.2541151
## factor3  0.42819940  0.27203419  1.0000000 -0.3665712
## factor4 -0.59140879 -0.25411510 -0.3665712  1.0000000
##
## Method of rotation:  Oblimin Quartimin
## [1] "The standard errors are close but do not match with other packages. Be mindful when usin
```

```r
inspect(c.ea4, "rsquare")    # Communalities (r^2)
```

```
##             active          ambitious         comforting
##              0.487              0.568              0.679
##          confident             driven           educated
##              0.565              0.623              0.780
```

```
##       enthusiastic          friendly       good.natured
##              0.412             0.794              0.716
##        hardworking high.social.status        intelligent
##              0.369             0.838              0.826
##               kind          powerful        prestigious
##              0.606             0.586              0.785
##          reputable         respected            skilled
##              0.618             0.677              0.648
##         successful          talented               warm
##              0.791             0.664              0.799
##            wealthy
##              0.818
```

```
# summary(c.eao4)   # Uncomment to view test statistics (se, p, ci)
# fitMeasures(c.ea4, c("chisq","df","pvalue","cfi","tli","rmsea","srmr"))
#       Uncomment to view listed fit measures
```

```
#   Remove 'talented', 'driven', and 'skilled', and repeat
EFA.Comp.Data(data.c[,c(2,4,6:7,9:20,22,24:25)], F.Max=10, Graph=T, Spearman=T)
```

```
## Number of factors to retain:  5
```

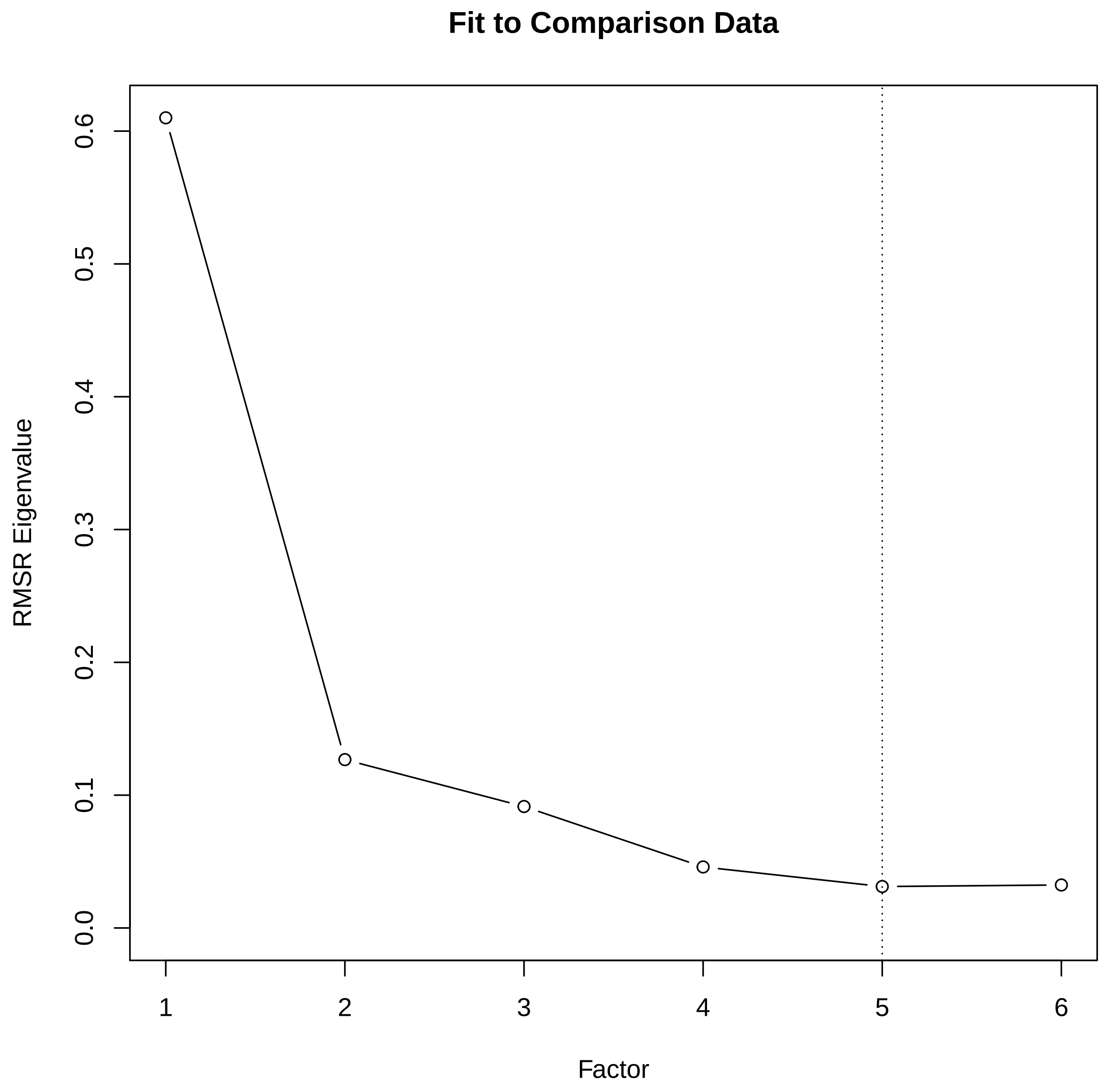


```
#   Run EFA with 5 factors
c.ea5 = efaUnrotate(data.c[,c(2,4,6:7,9:20,22,24:25)], 5, estimator="WLSMV")
c.eao5 = oblqRotate(c.ea5, method="oblimin")
c.eao5
```

```
## Standardized Rotated Factor Loadings
##                     factor1 factor2 factor3 factor4 factor5
## high.social.status   0.877*
## wealthy              0.871*
## prestigious          0.856*
## educated             0.548*           0.102*         -0.332*
## successful           0.533*           0.233* -0.144* -0.177*
## powerful             0.470* -0.118*  0.184* -0.294*
```

```
## warm                         0.881*
## friendly                     0.853*
## comforting          0.150*  0.796*
## good.natured                 0.779*
## kind               -0.208*  0.549*          0.123* -0.410*
## respected           0.354*  0.162*  0.479*         -0.106*
## reputable           0.341*  0.181*  0.404*
## hardworking        -0.263*  0.169*  0.401* -0.322*
## active                                     -0.689*
## enthusiastic        0.130*  0.348* -0.146* -0.509*
## confident           0.165*          0.181* -0.412* -0.184*
## intelligent         0.155*                         -0.744*
## ambitious           0.105*                 -0.222* -0.574*
##
## Factor Correlation
##             factor1     factor2    factor3    factor4    factor5
## factor1  1.00000000  0.05216044  0.5106525 -0.4571536 -0.4892874
## factor2  0.05216044  1.00000000  0.2938408 -0.2371475 -0.2243038
## factor3  0.51065252  0.29384079  1.0000000 -0.4371824 -0.3963016
## factor4 -0.45715360 -0.23714745 -0.4371824  1.0000000  0.3107074
## factor5 -0.48928743 -0.22430376 -0.3963016  0.3107074  1.0000000
##
## Method of rotation:  Oblimin Quartimin
## [1] "The standard errors are close but do not match with other packages. Be mindful when usin
```

```
inspect(c.ea5, "rsquare")    # Communalities (r^2)
```

```
##            active          ambitious         comforting
##             0.499              0.527              0.636
##         confident           educated       enthusiastic
##             0.529              0.737              0.476
##          friendly       good.natured        hardworking
##             0.756              0.671              0.380
## high.social.status        intelligent               kind
##             0.813              0.733              0.481
##          powerful        prestigious          reputable
##             0.558              0.769              0.621
##         respected         successful               warm
##             0.708              0.757              0.769
##           wealthy
##             0.783
```

```
# summary(c.eao5)   # Uncomment to view test statistics (se, p, ci)
# fitMeasures(c.ea5, c("chisq","df","pvalue","cfi","tli","rmsea","srmr"))
#       Uncomment to view listed fit measures
```

Confirmatory Factor Analysis

```
#   Define CFA model

#       Standard PRI model
cfa.model1 = "
prestige             =~ position + reputation + information

position             =~ wealthy + powerful + high.social.status
reputation           =~ reputable + respected
information          =~ educated + intelligent
"
#       Model of PRI subscales and other domains
#           (for discriminant validity assessment)
cfa.model2 = "
position             =~ wealthy + powerful + high.social.status
reputation           =~ reputable + respected
information          =~ educated + intelligent

solidarity           =~ friendly + kind + good.natured + warm + comforting
dynamism             =~ aggressive + active + confident + enthusiastic
"

#       Model of other domains only
#           (for criterion validity comparative study)
cfa.model3 = "
solidarity           =~ friendly + kind + good.natured + warm + comforting
dynamism             =~ active + aggressive + confident + enthusiastic
"
```

Measurement Invariance

Country

```
permuteMeasEq(20, "mgcfa", cfa(cfa.model1, data=data.c,
                               group="country",
                               estimator="WLSMV"),
              AFIs=c("chisq","CFI","RMSEA"), showProgress=F)
```

```
## Omnibus p value based on parametric chi-squared difference test:
##
## Chisq diff    Df diff Pr(>Chisq)
##     27.710     22.000      0.185
##
##
## Omnibus p values based on nonparametric permutation method:
##
```

```
##       AFI.Difference p.value
## chisq         27.710       1
## cfi            1.000       1
## rmsea          0.013       1
```

```
measurementInvariance(cfa.model1, data.c,
                      group="country",
                      estimator="WLSMV", strict=T)
```

```
##
## Measurement invariance models:
##
## Model 1 : fit.configural
## Model 2 : fit.loadings
## Model 3 : fit.intercepts
## Model 4 : fit.residuals
## Model 5 : fit.means
##
## Scaled Chi Square Difference Test (method = "satorra.bentler.2001")
##
##                 Df AIC BIC   Chisq Chisq diff Df diff Pr(>Chisq)
## fit.configural 22          27.710
## fit.loadings   28          56.869     23.471       6  0.0006531 ***
## fit.intercepts 31          78.586     62.435       3  1.774e-13 ***
## fit.residuals  38          88.053     16.974       7  0.0175658 *
## fit.means      42         185.326     33.668       4  8.717e-07 ***
## ---
## Signif. codes:  0 '***' 0.001 '**' 0.01 '*' 0.05 '.' 0.1 ' ' 1
##
##
## Fit measures:
##
##                 cfi.scaled rmsea.scaled cfi.scaled.delta rmsea.scaled.delta
## fit.configural      0.982        0.065               NA                 NA
## fit.loadings        0.985        0.053            0.002              0.011
## fit.intercepts      0.978        0.061            0.007              0.008
## fit.residuals       0.977        0.056            0.001              0.005
## fit.means           0.967        0.064            0.010              0.008
```

Age

```
permuteMeasEq(20, "mgcfa", cfa(cfa.model1, data=data.c,
                               group="age.o",
                               estimator="WLSMV"),
              AFIs=c("chisq","CFI","RMSEA"), showProgress=F)
```

```
## Omnibus p value based on parametric chi-squared difference test:
##
## Chisq diff    Df diff Pr(>Chisq)
```

```
##     40.722    110.000      1.000
##
##
## Omnibus p values based on nonparametric permutation method:
##
##       AFI.Difference p.value
## chisq         40.722    0.85
## cfi            1.000    1.00
## rmsea          0.000    1.00
```

```
measurementInvariance(cfa.model1, data.c,
                      group="age.o",
                      estimator="WLSMV", strict=T)
```

```
##
## Measurement invariance models:
##
## Model 1 : fit.configural
## Model 2 : fit.loadings
## Model 3 : fit.intercepts
## Model 4 : fit.residuals
## Model 5 : fit.means
##
## Scaled Chi Square Difference Test (method = "satorra.bentler.2001")
##
##                 Df AIC BIC   Chisq Chisq diff Df diff Pr(>Chisq)
## fit.configural 110          40.722
## fit.loadings   164         119.742     40.486      54     0.9135
## fit.intercepts 191         169.791     82.107      27  1.804e-07 ***
## fit.residuals  254         234.753     70.416      63     0.2434
## fit.means      290         529.653     84.137      36  1.001e-05 ***
## ---
## Signif. codes:  0 '***' 0.001 '**' 0.01 '*' 0.05 '.' 0.1 ' ' 1
##
##
## Fit measures:
##
##                cfi.scaled rmsea.scaled cfi.scaled.delta rmsea.scaled.delta
## fit.configural      0.986        0.058               NA                 NA
## fit.loadings        0.993        0.034            0.007              0.024
## fit.intercepts      0.987        0.044            0.007              0.010
## fit.residuals       0.981        0.045            0.005              0.001
## fit.means           0.959        0.061            0.022              0.017
```

```
#   Compare item loadings between groups
cfa.age = cfa(cfa.model1, data=data.c,
            group="age.o", group.label=levels(data.c$age.o),
            estimator="WLSMV")
partab.age = parameterEstimates(cfa.age)
```

```
partab.age = partab.age[partab.age$lhs != "prestige" & partab.age$op == "=~",]
partab.age$rhs = factor(partab.age$rhs, ordered=T, levels=partab.age$rhs[1:7])

ggplot(partab.age, aes(x=rhs, y=est, fill=as.factor(group))) +
    geom_bar(position="dodge", stat="identity") +
    geom_errorbar(aes(ymin=ci.lower, ymax=ci.upper),
                width=0.5, position=position_dodge(0.9)) +
    scale_fill_manual(values=c("#332288","#88CCEE","#44AA99","#117733","#999933",
                               "#DDCC77","#661100","#CC6677","#882255","#AA4499"))
```

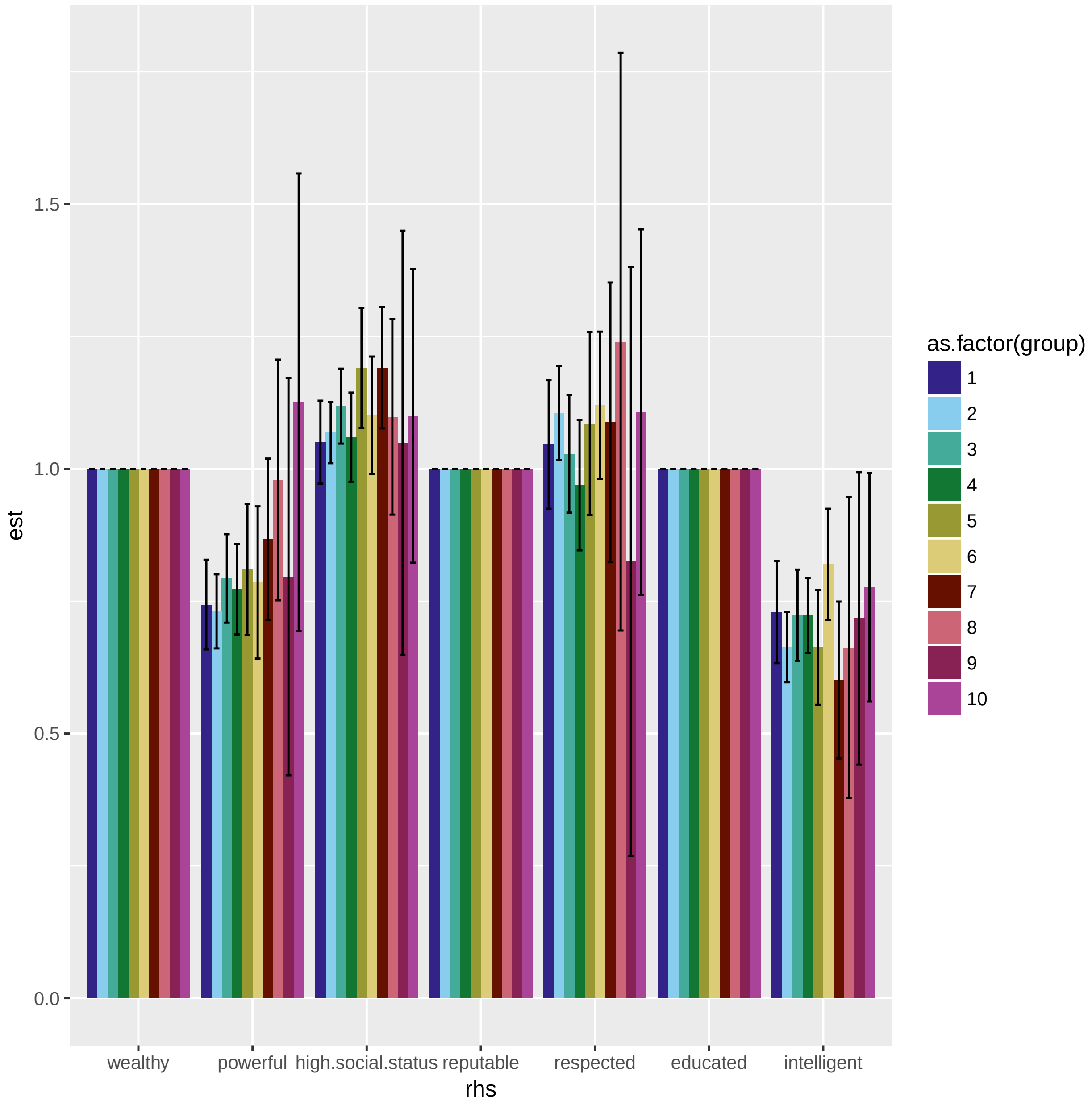

Gender *Note:* There were not enough participants of nonbinary gender to test that level, so only males and females are included.

```
permuteMeasEq(20, "mgcfa", cfa(cfa.model1,
                              data=data.c[data.c$gender != "nonbinary",],
                              group="age.o",
                              estimator="WLSMV"),
              AFIs=c("chisq","CFI","RMSEA"), showProgress=F)
```

```
## Omnibus p value based on parametric chi-squared difference test:
##
## Chisq diff    Df diff Pr(>Chisq)
##     41.076    110.000      1.000
##
##
## Omnibus p values based on nonparametric permutation method:
##
##       AFI.Difference p.value
## chisq         41.076     0.8
## cfi            1.000     1.0
## rmsea          0.000     1.0
```

```
measurementInvariance(cfa.model1, data.c[data.c$gender != "nonbinary",],
                      group="age.o",
                      estimator="WLSMV", strict=T)
```

```
##
## Measurement invariance models:
##
## Model 1 : fit.configural
## Model 2 : fit.loadings
## Model 3 : fit.intercepts
## Model 4 : fit.residuals
## Model 5 : fit.means
##
## Scaled Chi Square Difference Test (method = "satorra.bentler.2001")
##
##                 Df AIC BIC   Chisq Chisq diff Df diff Pr(>Chisq)
## fit.configural 110          41.076
## fit.loadings   164         120.071     40.466      54     0.9138
## fit.intercepts 191         171.440     84.305      27  8.274e-08 ***
## fit.residuals  254         235.414     69.371      63     0.2714
## fit.means      290         534.947     85.403      36  6.793e-06 ***
## ---
## Signif. codes:  0 '***' 0.001 '**' 0.01 '*' 0.05 '.' 0.1 ' ' 1
##
##
## Fit measures:
##
##                cfi.scaled rmsea.scaled cfi.scaled.delta rmsea.scaled.delta
## fit.configural      0.986        0.058               NA                 NA
## fit.loadings        0.993        0.034            0.007              0.025
```

```
## fit.intercepts       0.986        0.044            0.007                 0.010
## fit.residuals        0.981        0.045            0.005                 0.001
## fit.means            0.959        0.062            0.022                 0.017
```

```
#   Compare item loadings between groups
cfa.g = cfa(cfa.model1, data=data.c[data.c$gender != "nonbinary",],
            group="gender", group.label=c("male","female"),
            estimator="WLSMV")
partab.g = parameterEstimates(cfa.g)
partab.g = partab.g[partab.g$lhs != "prestige" & partab.g$op == "=~",]
partab.g$rhs = factor(partab.g$rhs, ordered=T, levels=partab.g$rhs[1:7])

ggplot(partab.g, aes(x=rhs, y=est, fill=as.factor(group))) +
    geom_bar(position="dodge", stat="identity") +
    geom_errorbar(aes(ymin=ci.lower, ymax=ci.upper),
                width=0.5, position=position_dodge(0.9)) +
    scale_fill_manual(values=c("#4477AA","#CC6677"))
```

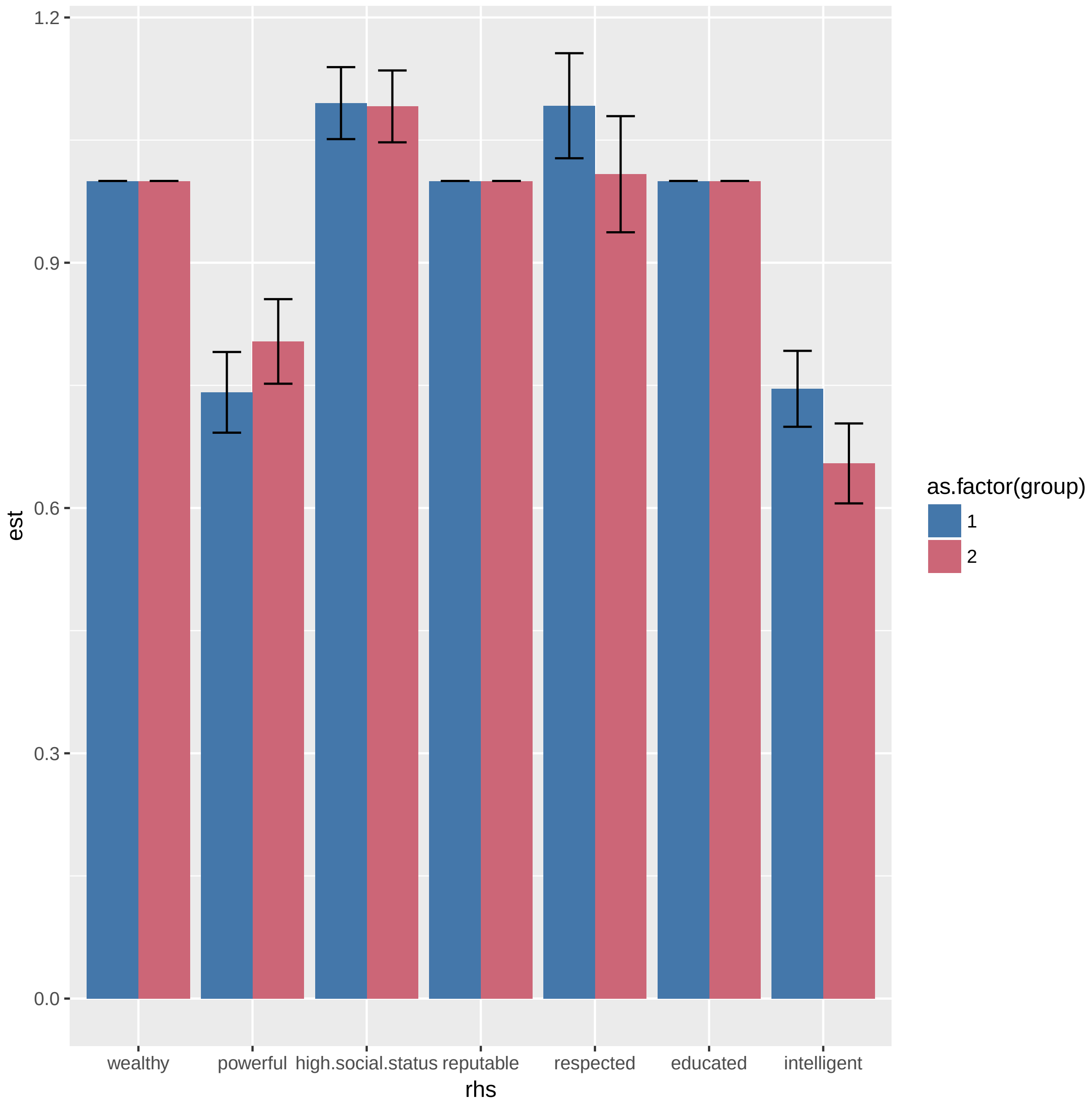

Ethnicity

```
permuteMeasEq(20, "mgcfa", cfa(cfa.model1, data=data.c,
                               group="ethnicity",
                               estimator="WLSMV"),
              AFIs=c("chisq","CFI","RMSEA"), showProgress=F)
```

```
## Omnibus p value based on parametric chi-squared difference test:
##
## Chisq diff    Df diff Pr(>Chisq)
##     33.671     33.000      0.435
##
```

```
##
## Omnibus p values based on nonparametric permutation method:
##
##       AFI.Difference p.value
## chisq         33.671    0.05
## cfi            1.000    0.05
## rmsea          0.005    0.05
```

```
measurementInvariance(cfa.model1, data.c,
                      group="ethnicity",
                      estimator="WLSMV", strict=T)
```

```
##
## Measurement invariance models:
##
## Model 1 : fit.configural
## Model 2 : fit.loadings
## Model 3 : fit.intercepts
## Model 4 : fit.residuals
## Model 5 : fit.means
##
## Scaled Chi Square Difference Test (method = "satorra.bentler.2001")
##
##                Df AIC BIC  Chisq Chisq diff Df diff Pr(>Chisq)
## fit.configural 33         33.671
## fit.loadings   45         56.086    13.3571      12     0.3436
## fit.intercepts 51         72.995    27.9548       6  9.582e-05 ***
## fit.residuals  65         79.081     7.6665      14     0.9060
## fit.means      73         93.456     4.4325       8     0.8161
## ---
## Signif. codes:  0 '***' 0.001 '**' 0.01 '*' 0.05 '.' 0.1 ' ' 1
##
##
## Fit measures:
##
##                cfi.scaled rmsea.scaled cfi.scaled.delta rmsea.scaled.delta
## fit.configural      0.985        0.060               NA                 NA
## fit.loadings        0.992        0.037            0.007              0.022
## fit.intercepts      0.989        0.041            0.003              0.004
## fit.residuals       0.990        0.035            0.001              0.006
## fit.means           0.992        0.029            0.003              0.007
```

```
#   Compare item loadings between groups
cfa.e = cfa(cfa.model1, data=data.c,
            group="ethnicity", group.label=c("white","mixed","poc"),
            estimator="WLSMV")
partab.e = parameterEstimates(cfa.e)
partab.e = partab.e[partab.e$lhs != "prestige" & partab.e$op == "=~",]
partab.e$rhs = factor(partab.e$rhs, ordered=T, levels=partab.e$rhs[1:7])
```

```
ggplot(partab.e, aes(x=rhs, y=est, fill=as.factor(group))) +
    geom_bar(position="dodge", stat="identity") +
    geom_errorbar(aes(ymin=ci.lower, ymax=ci.upper),
                  width=0.5, position=position_dodge(0.9)) +
    scale_fill_manual(values=c("#4477AA","#DDCC77","#CC6677"))
```

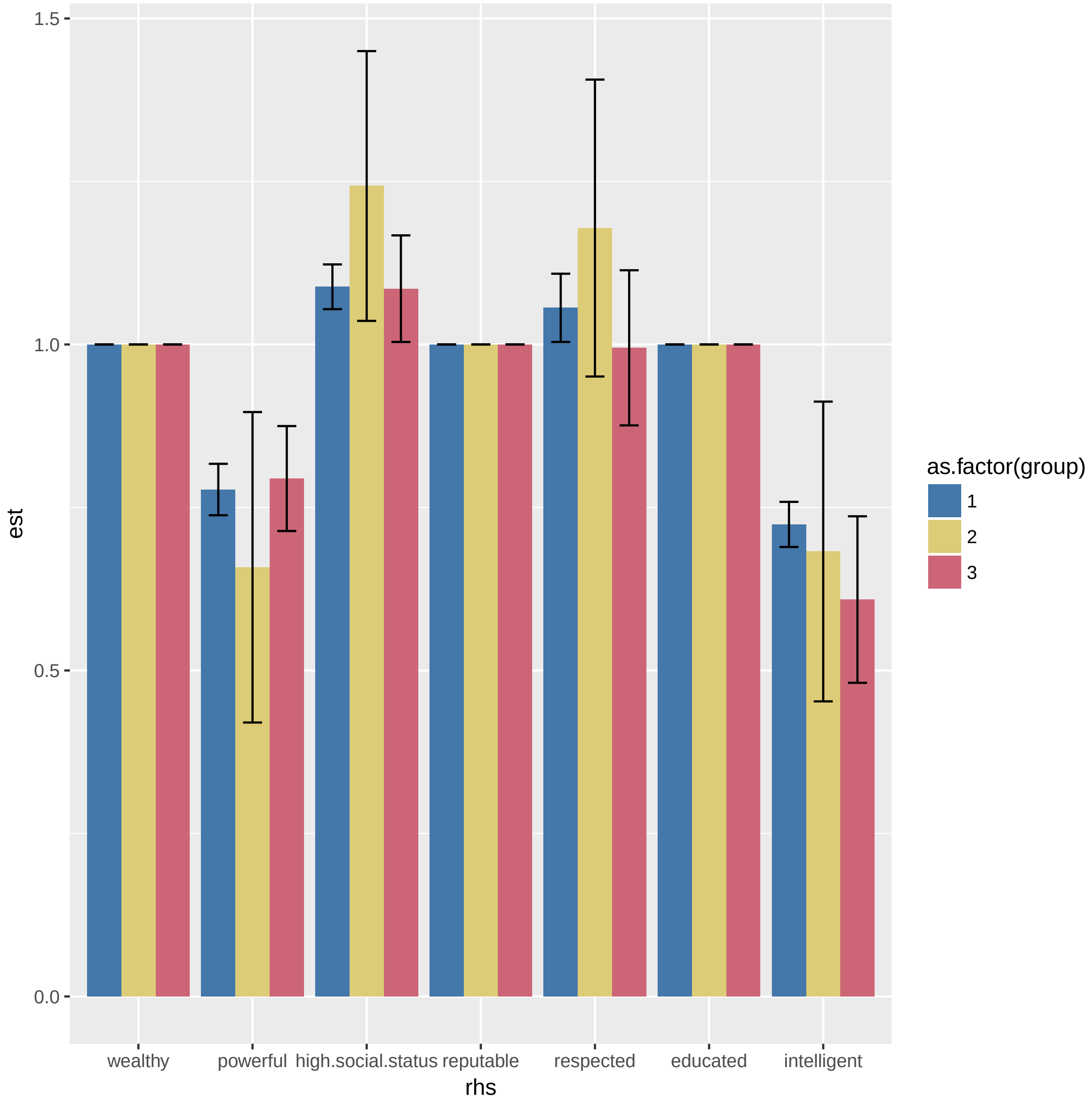

Locality

```
permuteMeasEq(20, "mgcfa", cfa(cfa.model1, data=data.c,
                              group="locality",
                              estimator="WLSMV"),
```

```
            AFIs=c("chisq","CFI","RMSEA"), showProgress=F)
```

```
## Omnibus p value based on parametric chi-squared difference test:
##
## Chisq diff    Df diff Pr(>Chisq)
##     47.175     77.000      0.997
##
##
## Omnibus p values based on nonparametric permutation method:
##
##       AFI.Difference p.value
## chisq         47.175       0
## cfi            1.000       1
## rmsea          0.000       1
```

```
measurementInvariance(cfa.model1, data.c,
                      group="locality",
                      estimator="WLSMV", strict=T)
```

```
##
## Measurement invariance models:
##
## Model 1 : fit.configural
## Model 2 : fit.loadings
## Model 3 : fit.intercepts
## Model 4 : fit.residuals
## Model 5 : fit.means
##
## Scaled Chi Square Difference Test (method = "satorra.bentler.2001")
##
##                 Df AIC BIC   Chisq Chisq diff Df diff Pr(>Chisq)
## fit.configural  77          47.175
## fit.loadings   113         109.234     39.151      36    0.33026
## fit.intercepts 131         122.994     27.762      18    0.06577 .
## fit.residuals  173         157.327     45.278      42    0.33682
## fit.means      197         182.466      7.232      24    0.99961
## ---
## Signif. codes:  0 '***' 0.001 '**' 0.01 '*' 0.05 '.' 0.1 ' ' 1
##
##
## Fit measures:
##
##                cfi.scaled rmsea.scaled cfi.scaled.delta rmsea.scaled.delta
## fit.configural      0.977        0.075               NA                 NA
## fit.loadings        0.988        0.044            0.011              0.030
## fit.intercepts      0.987        0.044            0.002              0.000
## fit.residuals       0.984        0.042            0.003              0.002
## fit.means           0.991        0.030            0.007              0.012
```

```
#   Compare item loadings between groups
```

```
cfa.l = cfa(cfa.model1, data=data.c,
            group="locality", group.label=levels(data.c$locality),
            estimator="WLSMV")
partab.l = parameterEstimates(cfa.l)
partab.l = partab.l[partab.l$lhs != "prestige" & partab.l$op == "=~",]
partab.l$rhs = factor(partab.l$rhs, ordered=T, levels=partab.l$rhs[1:7])

ggplot(partab.l, aes(x=rhs, y=est, fill=as.factor(group))) +
    geom_bar(position="dodge", stat="identity") +
    geom_errorbar(aes(ymin=ci.lower, ymax=ci.upper),
                width=0.5, position=position_dodge(0.9)) +
    scale_fill_manual(values=c("#332288","#88CCEE","#44AA99","#117733","#DDCC77",
                               "#CC6677","#AA4499"))
```

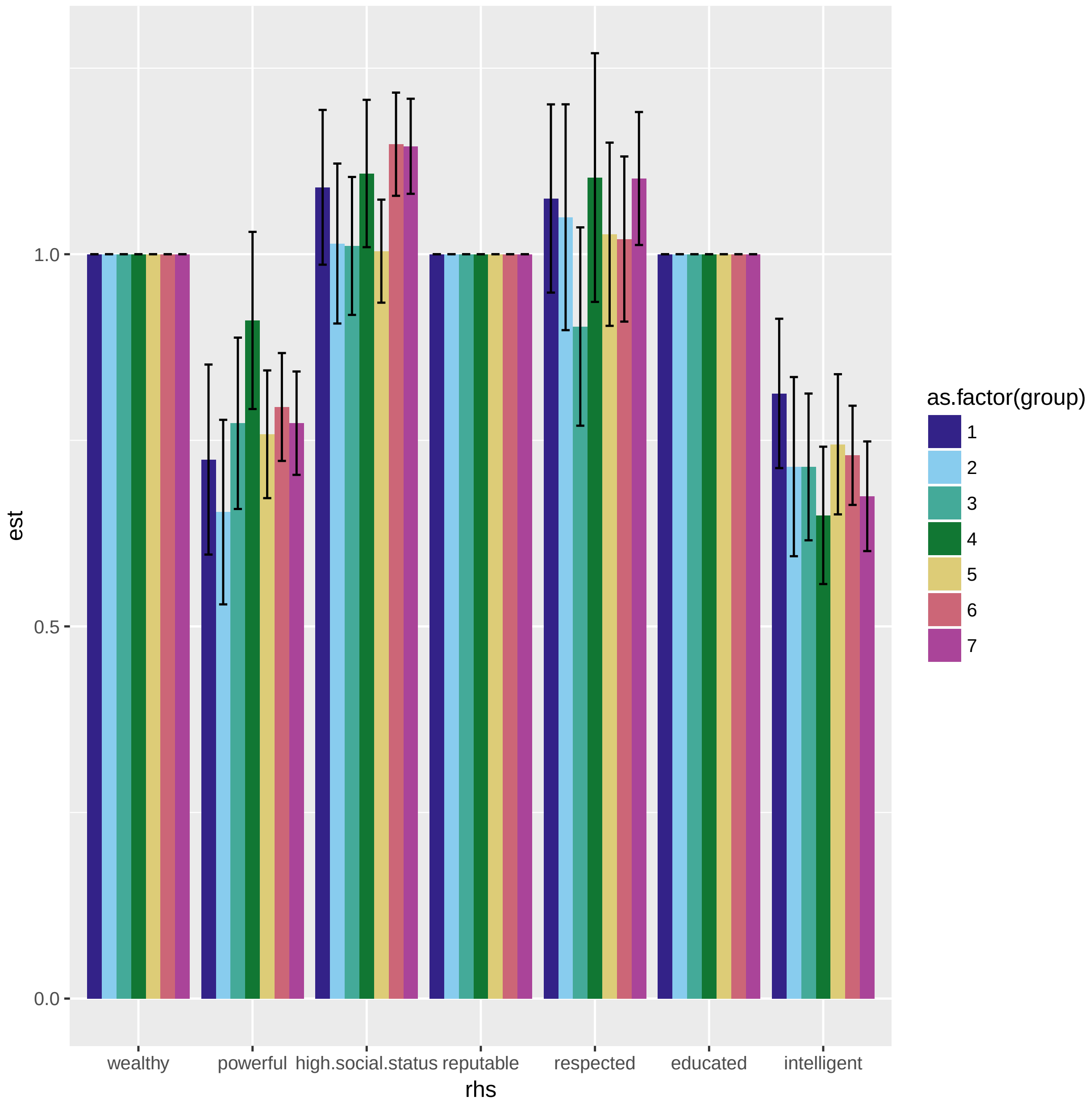

Education

Note: There were not enough participants of the lowest education level to test, so it is excluded.

```
permuteMeasEq(20, "mgcfa", cfa(cfa.model1, data=data.c[data.c$education != "1",],
                               group="education",
                               estimator="WLSMV"),
              AFIs=c("chisq","CFI","RMSEA"), showProgress=F)
```

```
## Omnibus p value based on parametric chi-squared difference test:
##
## Chisq diff    Df diff Pr(>Chisq)
```

```
##     36.397     44.000      0.785
##
##
## Omnibus p values based on nonparametric permutation method:
##
##       AFI.Difference p.value
## chisq         36.397       0
## cfi            1.000       1
## rmsea          0.000       1
```

```
measurementInvariance(cfa.model1, data.c[data.c$education != "1",],
                      group="education",
                      estimator="WLSMV", strict=T)
```

```
##
## Measurement invariance models:
##
## Model 1 : fit.configural
## Model 2 : fit.loadings
## Model 3 : fit.intercepts
## Model 4 : fit.residuals
## Model 5 : fit.means
##
## Scaled Chi Square Difference Test (method = "satorra.bentler.2001")
##
##                 Df AIC BIC   Chisq Chisq diff Df diff Pr(>Chisq)
## fit.configural  44          36.397
## fit.loadings    62          79.246     27.071      18    0.07767 .
## fit.intercepts  71          87.986     18.824       9    0.02673 *
## fit.residuals   92         104.887     23.317      21    0.32733
## fit.means      104         138.085     10.057      12    0.61095
## ---
## Signif. codes:  0 '***' 0.001 '**' 0.01 '*' 0.05 '.' 0.1 ' ' 1
##
##
## Fit measures:
##
##                cfi.scaled rmsea.scaled cfi.scaled.delta rmsea.scaled.delta
## fit.configural      0.980        0.071               NA                 NA
## fit.loadings        0.989        0.045            0.009              0.026
## fit.intercepts      0.987        0.044            0.001              0.000
## fit.residuals       0.986        0.040            0.001              0.004
## fit.means           0.989        0.034            0.002              0.006
```

```
#   Compare item loadings between groups
cfa.ed = cfa(cfa.model1, data=data.c[data.c$education != "1",],
            group="education", group.label=2:5,
            estimator="WLSMV")
partab.ed = parameterEstimates(cfa.ed)
```

```
partab.ed = partab.ed[partab.ed$lhs != "prestige" & partab.ed$op == "=~",]
partab.ed$rhs = factor(partab.ed$rhs, ordered=T, levels=partab.ed$rhs[1:7])

ggplot(partab.ed, aes(x=rhs, y=est, fill=as.factor(group))) +
    geom_bar(position="dodge", stat="identity") +
    geom_errorbar(aes(ymin=ci.lower, ymax=ci.upper),
                  width=0.5, position=position_dodge(0.9)) +
    scale_fill_manual(values=c("#4477AA","#117733","#DDCC77","#CC6677"))
```

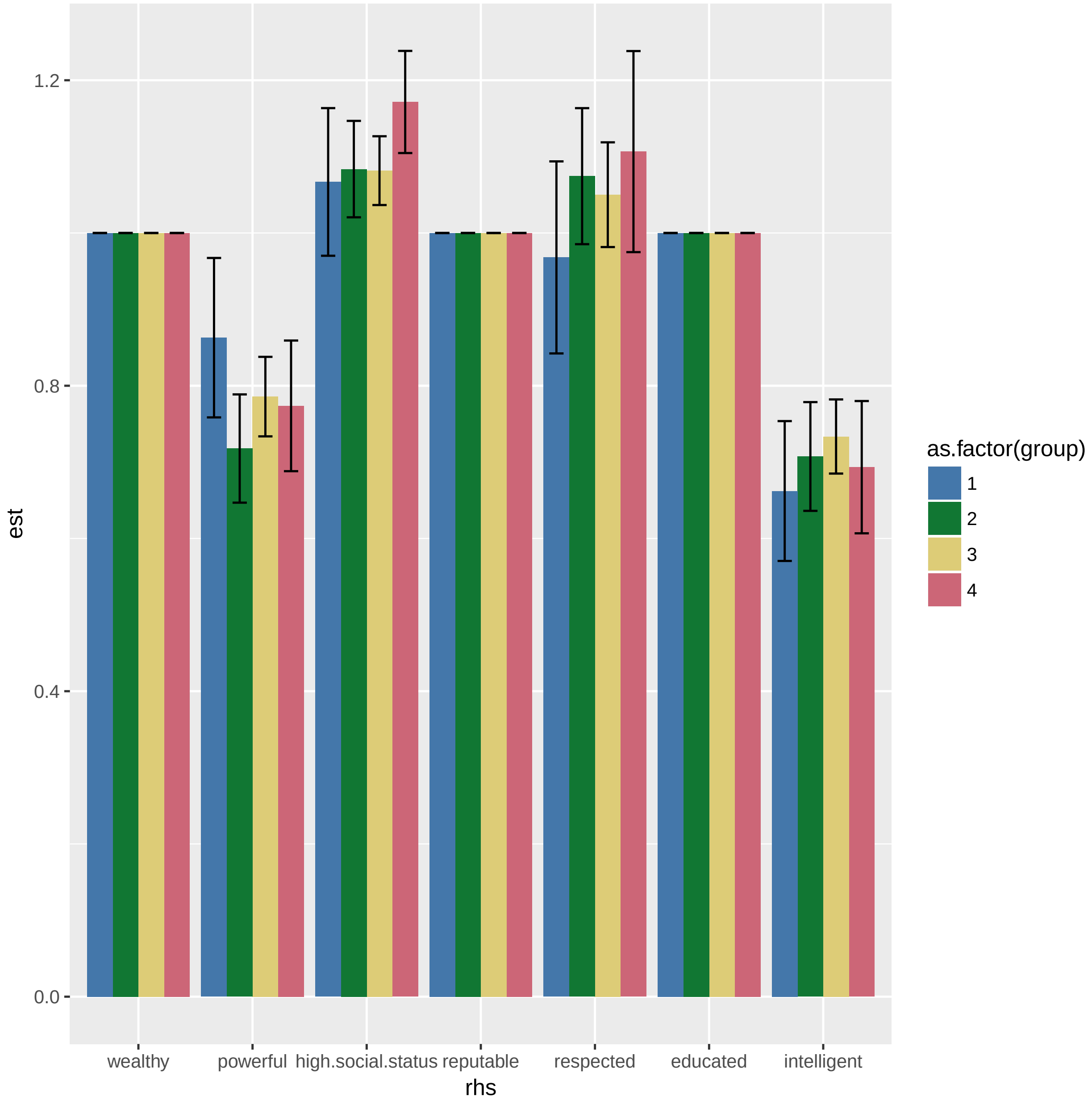

Occupation

```r
permuteMeasEq(20, "mgcfa", cfa(cfa.model1, data=data.c,
                              group="occupation",
                              estimator="WLSMV"),
              AFIs=c("chisq","CFI","RMSEA"), showProgress=F)
```

```
## Omnibus p value based on parametric chi-squared difference test:
##
## Chisq diff    Df diff Pr(>Chisq)
##     44.257     77.000      0.999
##
##
## Omnibus p values based on nonparametric permutation method:
##
##       AFI.Difference p.value
## chisq         44.257       0
## cfi            1.000       1
## rmsea          0.000       1
```

```r
measurementInvariance(cfa.model1, data.c,
                      group="occupation",
                      estimator="WLSMV", strict=T)
```

```
##
## Measurement invariance models:
##
## Model 1 : fit.configural
## Model 2 : fit.loadings
## Model 3 : fit.intercepts
## Model 4 : fit.residuals
## Model 5 : fit.means
##
## Scaled Chi Square Difference Test (method = "satorra.bentler.2001")
##
##                 Df AIC BIC   Chisq Chisq diff Df diff Pr(>Chisq)
## fit.configural  77          44.257
## fit.loadings   113         140.238     51.124      36   0.048804 *
## fit.intercepts 131         162.791     36.160      18   0.006731 **
## fit.residuals  173         186.012     26.103      42   0.974065
## fit.means      197         253.791     21.859      24   0.587700
## ---
## Signif. codes:  0 '***' 0.001 '**' 0.01 '*' 0.05 '.' 0.1 ' ' 1
##
##
## Fit measures:
##
##                cfi.scaled rmsea.scaled cfi.scaled.delta rmsea.scaled.delta
## fit.configural      0.985        0.061               NA                 NA
## fit.loadings        0.986        0.049            0.001              0.012
## fit.intercepts      0.984        0.049            0.003              0.001
```

```
## fit.residuals       0.984        0.043            0.000              0.006
## fit.means           0.984        0.040            0.000              0.003
```

Income

Note: There were not enough participants in the uppermost 3 levels to test, so they are excluded.

```
permuteMeasEq(20, "mgcfa", cfa(cfa.model1, data=data.c[data.c$income != "9" &
                                            data.c$income != "10" &
                                            data.c$income != "11",],
                               group="occupation",
                               estimator="WLSMV"),
              AFIs=c("chisq","CFI","RMSEA"), showProgress=F)
```

```
## Omnibus p value based on parametric chi-squared difference test:
##
## Chisq diff    Df diff Pr(>Chisq)
##     43.413     77.000      0.999
##
##
## Omnibus p values based on nonparametric permutation method:
##
##       AFI.Difference p.value
## chisq         43.413       0
## cfi            1.000       1
## rmsea          0.000       1
```

```
measurementInvariance(cfa.model1, data.c[data.c$income != "9" &
                                            data.c$income != "10" &
                                            data.c$income != "11",],
                      group="occupation",
                      estimator="WLSMV", strict=T)
```

```
##
## Measurement invariance models:
##
## Model 1 : fit.configural
## Model 2 : fit.loadings
## Model 3 : fit.intercepts
## Model 4 : fit.residuals
## Model 5 : fit.means
##
## Scaled Chi Square Difference Test (method = "satorra.bentler.2001")
##
##                 Df AIC BIC   Chisq Chisq diff Df diff Pr(>Chisq)
## fit.configural  77          43.413
## fit.loadings   113         137.990     50.399      36   0.056078 .
## fit.intercepts 131         159.801     34.879      18   0.009791 **
```

```
## fit.residuals  173         182.461     25.447      42   0.979514
## fit.means      197         246.164     20.588      24   0.662912
## ---
## Signif. codes:  0 '***' 0.001 '**' 0.01 '*' 0.05 '.' 0.1 ' ' 1
##
##
## Fit measures:
##
##                cfi.scaled rmsea.scaled cfi.scaled.delta rmsea.scaled.delta
## fit.configural      0.986        0.060               NA                 NA
## fit.loadings        0.986        0.049            0.001              0.012
## fit.intercepts      0.984        0.049            0.003              0.001
## fit.residuals       0.984        0.043            0.000              0.006
## fit.means           0.985        0.039            0.001              0.003
```

Confirmatory Factor Analysis

```
#   Determine weights for complex survey design using rough estimates
#       from census data

#   US Ethnicity
#       Source: https://factfinder.census.gov/faces/tableservices/jsf/pages/productview.xhtml?pi
#       us white: 233657078
#       us poc: 75148137
#       us mixed: 9752947
#   UK Ethnicity
#       Source: http://www.ons.gov.uk/ons/rel/census/2011-census/key-statistics-and-quick-statis
#       uk white: 55010359
#       uk poc: 6921590
#       uk mixed: 1250229
#   Ethnicity Probabilities
#       ((0.5 * (x / 318558162)) + (0.5 * (y / 63182178)))
#       white: 0.8020729
#       poc: 0.1727253
#       mixed: 0.0252018
#   Ethnicity Weights
#       (pop prob / sample prob), e.g.
#        0.8020729 / (nrow(data.c[!duplicated(data.c$id) &
#                                 data.c$ethnicity == "white",]) /
#                         nrow(data.c[!duplicated(data.c$id),]))
#       white: 0.9644761
#       poc: 1.322712
#       mixed: 0.6667022

#   US Locality
#       Source: https://www.census.gov/geo/reference/ua/urban-rural-2010.html
#       us locality 1: ~59492267 (rural)
```

```
#       us locality 2: ~9777049
#       us locality 3: ~9777049
#       us locality 4: ~9777049
#       us locality 5: ~73307374
#       us locality 6: ~73307374
#       us locality 7: ~73307374
#   UK Locality
#       Source: https://www.gov.uk/government/publications/rural-population-and-migration/rural-
#       uk locality 1: ~192085 (sparse rural town)
#       uk locality 2: ~298045 (sparse rural village)
#       uk locality 3: ~4811871 (rural town)
#       uk locality 4: ~3958891 (rural village)
#       uk locality 5: ~1948518 (urban minor conurbation)
#       uk locality 6: ~19415739 (urban major conurbation)
#       uk locality 7: ~23691469 (urban city and town)
#   Locality Probabilities
#       ((0.5 * (x / 308745536)) + (0.5 * (y / 54316618)))
#       locality 1: 0.09811334
#       locality 2: 0.0185771
#       locality 3: 0.06012816
#       locality 4: 0.05227623
#       locality 5: 0.1366548
#       locality 6: 0.2974455
#       locality 7: 0.3368049
#   Locality Weights
#       (pop prob / sample prob), e.g.
#        0.09811334 / (nrow(data.c[!duplicated(data.c$id) &
#                                  data.c$locality == "1",]) /
#                          nrow(data.c[!duplicated(data.c$id),]))
#       locality 1: 0.9516994
#       locality 2: 0.2162374
#       locality 3: 0.6249034
#       locality 4: 0.4609813
#       locality 5: 1.046488
#       locality 6: 1.4923565
#       locality 7: 1.240636

#   US Education
#       Source: https://factfinder.census.gov/faces/tableservices/jsf/pages/productview.xhtml?pi
#       us education 1: 32732542
#       us education 2: 112573532
#       us education 3: 31488942
#       us education 4: 42249955
#       us education 5: 23786225
#   UK Education
#       Source: https://www.nomisweb.co.uk/census/2011/QS501UK/view/2092957697?cols=measures
#       uk education 1: 14529079
#       uk education 2: 7223057
```

```
#       uk education 3: 15690573
#       uk education 4: ~6932341.5
#       uk education 5: ~6932341.5
#   Education Probabilities
#       ((0.5 * (x / 242831196)) + (0.5 * (y / 51307392)))
#       education 1: 0.2089863
#       education 2: 0.3021838
#       education 3: 0.2177446
#       education 4: 0.1545514
#       education 5: 0.1165338
#   Education Weights
#       (pop prob / sample prob), e.g.
#        0.2089863 / (nrow(data.c[!duplicated(data.c$id) &
#                                 data.c$education == "1",]) /
#                          nrow(data.c[!duplicated(data.c$id),]))
#       education 1: 20.27167
#       education 2: 1.870968
#       education 3: 0.8924462
#       education 4: 0.3433165
#       education 5: 0.8695214

data.c$w.ethnicity = ifelse(data.c$ethnicity == "white", 0.9644761,
                       ifelse(data.c$ethnicity == "poc", 1.322712,
                                                          0.6667022))
data.c$w.locality = ifelse(data.c$locality == "1", 0.9516994,
                    ifelse(data.c$locality == "2", 0.2162374,
                    ifelse(data.c$locality == "3", 0.6249034,
                    ifelse(data.c$locality == "4", 0.4609813,
                    ifelse(data.c$locality == "5", 1.046488,
                    ifelse(data.c$locality == "6", 1.4923565,
                                                    1.240636))))))
data.c$w.education = ifelse(data.c$education == "1", 20.27167,
                      ifelse(data.c$education == "2", 1.870968,
                      ifelse(data.c$education == "3", 0.8924462,
                      ifelse(data.c$education == "4", 0.3433165,
                                                       0.8695214))))

#   Specify complex survey design
cfa.design = svydesign(ids=~1, strata=~ethnicity+locality+education,
                       weights=~w.ethnicity+w.locality+w.education, data=data.c)

#   Fit CFA model
cfa.fit = cfa(cfa.model1, data.c, estimator="MLMVS")
summary(cfa.fit, fit.measures=T, ci=T)
```

```
## lavaan (0.5-23.1097) converged normally after  30 iterations
##
##   Number of observations                          2910
##
```

```
##   Estimator                                         ML      Robust
##   Minimum Function Test Statistic              138.901     105.849
##   Degrees of freedom                                11      10.551
##   P-value (Chi-square)                           0.000       0.000
##   Scaling correction factor                                  1.312
##     for the mean and variance adjusted correction
##
## Model test baseline model:
##
##   Minimum Function Test Statistic            12964.682    1837.389
##   Degrees of freedom                                21       3.358
##   P-value                                        0.000       0.000
##
## User model versus baseline model:
##
##   Comparative Fit Index (CFI)                    0.990       0.948
##   Tucker-Lewis Index (TLI)                       0.981       0.983
##
##   Robust Comparative Fit Index (CFI)                            NA
##   Robust Tucker-Lewis Index (TLI)                               NA
##
## Loglikelihood and Information Criteria:
##
##   Loglikelihood user model (H0)             -30934.331  -30934.331
##   Loglikelihood unrestricted model (H1)     -30864.880  -30864.880
##
##   Number of free parameters                         24          24
##   Akaike (AIC)                               61916.662   61916.662
##   Bayesian (BIC)                             62060.083   62060.083
##   Sample-size adjusted Bayesian (BIC)        61983.827   61983.827
##
## Root Mean Square Error of Approximation:
##
##   RMSEA                                          0.063       0.056
##   90 Percent Confidence Interval          0.054  0.073       0.048  0.064
##   P-value RMSEA <= 0.05                          0.009       0.123
##
##   Robust RMSEA                                                  NA
##   90 Percent Confidence Interval                                NA     NA
##
## Standardized Root Mean Square Residual:
##
##   SRMR                                           0.020       0.020
##
## Parameter Estimates:
##
##   Information                                 Expected
##   Standard Errors                           Robust.sem
```

```
##
## Latent Variables:
##                    Estimate  Std.Err  z-value  P(>|z|) ci.lower ci.upper
##   prestige =~
##     position          1.000                               1.000    1.000
##     reputation        0.760    0.021   36.654    0.000    0.719    0.801
##     information       1.115    0.023   47.959    0.000    1.070    1.161
##   position =~
##     wealthy           1.000                               1.000    1.000
##     powerful          0.742    0.017   42.578    0.000    0.708    0.776
##     high.socl.stts    1.085    0.015   70.883    0.000    1.055    1.115
##   reputation =~
##     reputable         1.000                               1.000    1.000
##     respected         1.049    0.024   42.913    0.000    1.001    1.097
##   information =~
##     educated          1.000                               1.000    1.000
##     intelligent       0.711    0.017   41.735    0.000    0.678    0.744
##
## Intercepts:
##                    Estimate  Std.Err  z-value  P(>|z|) ci.lower ci.upper
##    .wealthy           3.674    0.029  126.757    0.000    3.617    3.730
##    .powerful          3.681    0.028  131.530    0.000    3.627    3.736
##    .high.socl.stts    3.772    0.031  122.829    0.000    3.712    3.832
##    .reputable         4.511    0.026  176.393    0.000    4.461    4.561
##    .respected         4.579    0.025  179.884    0.000    4.529    4.629
##    .educated          4.505    0.030  151.321    0.000    4.447    4.564
##    .intelligent       5.396    0.028  190.048    0.000    5.340    5.452
##     prestige          0.000                               0.000    0.000
##     position          0.000                               0.000    0.000
##     reputation        0.000                               0.000    0.000
##     information       0.000                               0.000    0.000
##
## Variances:
##                    Estimate  Std.Err  z-value  P(>|z|) ci.lower ci.upper
##    .wealthy           0.522    0.028   18.583    0.000    0.467    0.577
##    .powerful          1.221    0.042   28.833    0.000    1.138    1.304
##    .high.socl.stts    0.481    0.029   16.648    0.000    0.424    0.538
##    .reputable         0.671    0.032   20.661    0.000    0.608    0.735
##    .respected         0.530    0.031   16.937    0.000    0.469    0.592
##    .educated          0.249    0.036    6.903    0.000    0.178    0.320
##    .intelligent       1.168    0.043   27.245    0.000    1.084    1.252
##     prestige          1.586    0.056   28.460    0.000    1.477    1.695
##     position          0.335    0.030   11.028    0.000    0.276    0.395
##     reputation        0.315    0.027   11.659    0.000    0.262    0.368
##     information       0.357    0.048    7.388    0.000    0.262    0.452
```

```
#   Re-fit CFA model using complex survey design
cfa.complex = lavaan.survey(cfa.fit, cfa.design, estimator="MLMVS")
```

```r
summary(cfa.complex, fit.measures=T, ci=T)
```

```
## lavaan (0.5-23.1097) converged normally after  30 iterations
##
##   Number of observations                          2910
##
##   Estimator                                         ML      Robust
##   Minimum Function Test Statistic              170.822      31.688
##   Degrees of freedom                                11       8.390
##   P-value (Chi-square)                           0.000       0.000
##   Scaling correction factor                                  5.391
##     for the mean and variance adjusted correction
##
## Model test baseline model:
##
##   Minimum Function Test Statistic            11605.366     571.757
##   Degrees of freedom                                21       3.392
##   P-value                                        0.000       0.000
##
## User model versus baseline model:
##
##   Comparative Fit Index (CFI)                    0.986       0.959
##   Tucker-Lewis Index (TLI)                       0.974       0.983
##
##   Robust Comparative Fit Index (CFI)                            NA
##   Robust Tucker-Lewis Index (TLI)                               NA
##
## Loglikelihood and Information Criteria:
##
##   Loglikelihood user model (H0)             -31419.015  -31419.015
##   Loglikelihood unrestricted model (H1)     -31333.604  -31333.604
##
##   Number of free parameters                         24          24
##   Akaike (AIC)                               62886.030   62886.030
##   Bayesian (BIC)                             63029.451   63029.451
##   Sample-size adjusted Bayesian (BIC)        62953.195   62953.195
##
## Root Mean Square Error of Approximation:
##
##   RMSEA                                          0.071       0.031
##   90 Percent Confidence Interval          0.062  0.080       0.026  0.036
##   P-value RMSEA <= 0.05                          0.000       1.000
##
##   Robust RMSEA                                                  NA
##   90 Percent Confidence Interval                                NA     NA
##
## Standardized Root Mean Square Residual:
##
```

```
##   SRMR                                          0.023       0.023
##
## Parameter Estimates:
##
##   Information                                Expected
##   Standard Errors                          Robust.sem
##
## Latent Variables:
##                    Estimate  Std.Err  z-value  P(>|z|) ci.lower ci.upper
##   prestige =~
##     position          1.000                               1.000    1.000
##     reputation        0.769    0.042   18.227    0.000    0.686    0.852
##     information       1.142    0.038   30.255    0.000    1.068    1.216
##   position =~
##     wealthy           1.000                               1.000    1.000
##     powerful          0.708    0.031   22.821    0.000    0.647    0.769
##     high.socl.stts    1.091    0.024   45.984    0.000    1.044    1.137
##   reputation =~
##     reputable         1.000                               1.000    1.000
##     respected         1.055    0.046   23.030    0.000    0.965    1.145
##   information =~
##     educated          1.000                               1.000    1.000
##     intelligent       0.704    0.027   26.402    0.000    0.652    0.756
##
## Intercepts:
##                    Estimate  Std.Err  z-value  P(>|z|) ci.lower ci.upper
##    .wealthy           3.638    0.045   80.239    0.000    3.549    3.727
##    .powerful          3.605    0.046   77.858    0.000    3.514    3.695
##    .high.socl.stts    3.749    0.049   76.645    0.000    3.653    3.845
##    .reputable         4.497    0.047   95.345    0.000    4.405    4.590
##    .respected         4.621    0.040  116.184    0.000    4.543    4.699
##    .educated          4.570    0.045  101.523    0.000    4.482    4.658
##    .intelligent       5.472    0.053  103.746    0.000    5.369    5.576
##     prestige          0.000                               0.000    0.000
##     position          0.000                               0.000    0.000
##     reputation        0.000                               0.000    0.000
##     information       0.000                               0.000    0.000
##
## Variances:
##                    Estimate  Std.Err  z-value  P(>|z|) ci.lower ci.upper
##    .wealthy           0.536    0.043   12.582    0.000    0.452    0.619
##    .powerful          1.287    0.086   15.000    0.000    1.119    1.455
##    .high.socl.stts    0.537    0.044   12.107    0.000    0.450    0.623
##    .reputable         0.846    0.091    9.245    0.000    0.667    1.025
##    .respected         0.561    0.060    9.324    0.000    0.443    0.678
##    .educated          0.196    0.054    3.620    0.000    0.090    0.303
##    .intelligent       1.238    0.059   21.168    0.000    1.124    1.353
##     prestige          1.418    0.088   16.037    0.000    1.245    1.591
```

```
##     position          0.402    0.056    7.160    0.000    0.292    0.512
##     reputation        0.296    0.050    5.901    0.000    0.198    0.394
##     information       0.375    0.079    4.737    0.000    0.220    0.530
```

```
#   Compare with model fit using WLSMV
cfa.fit2 = cfa(cfa.model1, data.c.o, estimator="WLSMV")
summary(cfa.fit2, fit.measures=T, ci=T)
```

```
## lavaan (0.5-23.1097) converged normally after  25 iterations
##
##   Number of observations                          2910
##
##   Estimator                                       DWLS      Robust
##   Minimum Function Test Statistic               89.785     295.085
##   Degrees of freedom                                11          11
##   P-value (Chi-square)                           0.000       0.000
##   Scaling correction factor                                  0.306
##   Shift parameter                                            1.203
##     for simple second-order correction (Mplus variant)
##
## Model test baseline model:
##
##   Minimum Function Test Statistic           103476.799   49656.215
##   Degrees of freedom                                21          21
##   P-value                                        0.000       0.000
##
## User model versus baseline model:
##
##   Comparative Fit Index (CFI)                    0.999       0.994
##   Tucker-Lewis Index (TLI)                       0.999       0.989
##
##   Robust Comparative Fit Index (CFI)                            NA
##   Robust Tucker-Lewis Index (TLI)                               NA
##
## Root Mean Square Error of Approximation:
##
##   RMSEA                                          0.050       0.094
##   90 Percent Confidence Interval          0.040  0.059       0.085  0.104
##   P-value RMSEA <= 0.05                          0.505       0.000
##
##   Robust RMSEA                                                  NA
##   90 Percent Confidence Interval                                NA     NA
##
## Standardized Root Mean Square Residual:
##
##   SRMR                                           0.022       0.022
##
## Weighted Root Mean Square Residual:
##
```

```
##   WRMR                                          1.194       1.194
##
## Parameter Estimates:
##
##   Information                                Expected
##   Standard Errors                          Robust.sem
##
## Latent Variables:
##                    Estimate  Std.Err  z-value  P(>|z|) ci.lower ci.upper
##   prestige =~
##     position          1.000                               1.000    1.000
##     reputation        0.887    0.013   69.277    0.000    0.862    0.912
##     information       1.092    0.013   83.513    0.000    1.066    1.117
##   position =~
##     wealthy           1.000                               1.000    1.000
##     powerful          0.790    0.010   76.777    0.000    0.770    0.810
##     high.socl.stts    1.026    0.008  133.162    0.000    1.011    1.041
##   reputation =~
##     reputable         1.000                               1.000    1.000
##     respected         1.054    0.013   79.889    0.000    1.028    1.080
##   information =~
##     educated          1.000                               1.000    1.000
##     intelligent       0.768    0.012   64.728    0.000    0.745    0.792
##
## Intercepts:
##                    Estimate  Std.Err  z-value  P(>|z|) ci.lower ci.upper
##    .wealthy           0.000                               0.000    0.000
##    .powerful          0.000                               0.000    0.000
##    .high.socl.stts    0.000                               0.000    0.000
##    .reputable         0.000                               0.000    0.000
##    .respected         0.000                               0.000    0.000
##    .educated          0.000                               0.000    0.000
##    .intelligent       0.000                               0.000    0.000
##     prestige          0.000                               0.000    0.000
##     position          0.000                               0.000    0.000
##     reputation        0.000                               0.000    0.000
##     information       0.000                               0.000    0.000
##
## Thresholds:
##                    Estimate  Std.Err  z-value  P(>|z|) ci.lower ci.upper
##     wealthy|t1       -1.460    0.035  -41.823    0.000   -1.528   -1.391
##     wealthy|t2       -0.619    0.025  -24.821    0.000   -0.668   -0.570
##     wealthy|t3       -0.059    0.023   -2.558    0.011   -0.105   -0.014
##     wealthy|t4        0.478    0.024   19.711    0.000    0.430    0.525
##     wealthy|t5        1.090    0.029   37.564    0.000    1.033    1.147
##     wealthy|t6        1.802    0.044   41.164    0.000    1.717    1.888
##     powerful|t1      -1.517    0.036  -42.008    0.000   -1.588   -1.446
##     powerful|t2      -0.663    0.025  -26.324    0.000   -0.713   -0.614
```

```
##     powerful|t3      -0.074    0.023   -3.188    0.001   -0.120   -0.029
##     powerful|t4       0.469    0.024   19.382    0.000    0.421    0.516
##     powerful|t5       1.160    0.030   38.776    0.000    1.101    1.219
##     powerful|t6       1.919    0.048   40.054    0.000    1.825    2.013
##     hgh.scl.stts|1   -1.407    0.034  -41.540    0.000   -1.473   -1.341
##     hgh.scl.stts|2   -0.617    0.025  -24.749    0.000   -0.665   -0.568
##     hgh.scl.stts|3   -0.100    0.023   -4.299    0.000   -0.146   -0.054
##     hgh.scl.stts|4    0.370    0.024   15.532    0.000    0.323    0.417
##     hgh.scl.stts|5    0.934    0.027   34.173    0.000    0.880    0.987
##     hgh.scl.stts|6    1.647    0.039   41.978    0.000    1.570    1.723
##     reputable|t1     -2.062    0.054  -38.183    0.000   -2.168   -1.956
##     reputable|t2     -1.309    0.032  -40.734    0.000   -1.372   -1.246
##     reputable|t3     -0.737    0.026  -28.694    0.000   -0.787   -0.687
##     reputable|t4     -0.149    0.023   -6.374    0.000   -0.194   -0.103
##     reputable|t5      0.679    0.025   26.857    0.000    0.630    0.729
##     reputable|t6      1.653    0.039   41.959    0.000    1.576    1.730
##     respected|t1     -2.224    0.063  -35.515    0.000   -2.347   -2.102
##     respected|t2     -1.382    0.033  -41.368    0.000   -1.447   -1.316
##     respected|t3     -0.752    0.026  -29.149    0.000   -0.802   -0.701
##     respected|t4     -0.190    0.023   -8.114    0.000   -0.236   -0.144
##     respected|t5      0.629    0.025   25.180    0.000    0.580    0.678
##     respected|t6      1.539    0.037   42.047    0.000    1.468    1.611
##     educated|t1      -1.862    0.046  -40.643    0.000   -1.952   -1.773
##     educated|t2      -1.119    0.029  -38.082    0.000   -1.176   -1.061
##     educated|t3      -0.549    0.025  -22.367    0.000   -0.597   -0.501
##     educated|t4      -0.103    0.023   -4.410    0.000   -0.148   -0.057
##     educated|t5       0.464    0.024   19.199    0.000    0.417    0.511
##     educated|t6       1.297    0.032   40.609    0.000    1.235    1.360
##     intelligent|t1   -2.279    0.066  -34.518    0.000   -2.408   -2.149
##     intelligent|t2   -1.627    0.039  -42.021    0.000   -1.703   -1.551
##     intelligent|t3   -1.028    0.028  -36.333    0.000   -1.084   -0.973
##     intelligent|t4   -0.654    0.025  -26.003    0.000   -0.703   -0.604
##     intelligent|t5   -0.192    0.023   -8.225    0.000   -0.238   -0.147
##     intelligent|t6    0.549    0.025   22.367    0.000    0.501    0.597
##
## Variances:
##                    Estimate  Std.Err  z-value  P(>|z|) ci.lower ci.upper
##    .wealthy           0.192                               0.192    0.192
##    .powerful          0.496                               0.496    0.496
##    .high.socl.stts    0.150                               0.150    0.150
##    .reputable         0.318                               0.318    0.318
##    .respected         0.243                               0.243    0.243
##    .educated          0.069                               0.069    0.069
##    .intelligent       0.450                               0.450    0.450
##     prestige          0.670    0.012   56.409    0.000    0.646    0.693
##     position          0.138    0.009   15.975    0.000    0.121    0.155
##     reputation        0.155    0.009   17.079    0.000    0.137    0.172
##     information       0.133    0.014    9.620    0.000    0.106    0.160
```

```
##
## Scales y*:
##                    Estimate  Std.Err  z-value  P(>|z|) ci.lower ci.upper
##     wealthy           1.000                               1.000    1.000
##     powerful          1.000                               1.000    1.000
##     high.socl.stts    1.000                               1.000    1.000
##     reputable         1.000                               1.000    1.000
##     respected         1.000                               1.000    1.000
##     educated          1.000                               1.000    1.000
##     intelligent       1.000                               1.000    1.000
```

```
#   Plot path diagram of model fit using complex survey design
# pdf("figure2.pdf", width=8, height=8)

semPaths(cfa.complex, what="std", nCharNodes=0, edge.color="#424242",
         edge.width=0.5, label.cex=1.1, edge.label.cex=.9, cut=.001,
         intercepts=F, loop=.5,
         groups=list(
           prestige=c("prestige"),
           position=c("position","wealthy","powerful","high.social.status"),
           reputation=c("reputation","reputable","respected"),
           information=c("information","educated","intelligent")),
         nodeLabels=c("wealthy","powerful","high\nsocial\nstatus",
                      "reputable","respected",
                      "educated","intelligent",
                      "Prestige",
                      "Position","Reputation","Information"),
         color=c("#FFFFFF","#4477AA","#DDCC77","#CC6677"), legend=F)
```

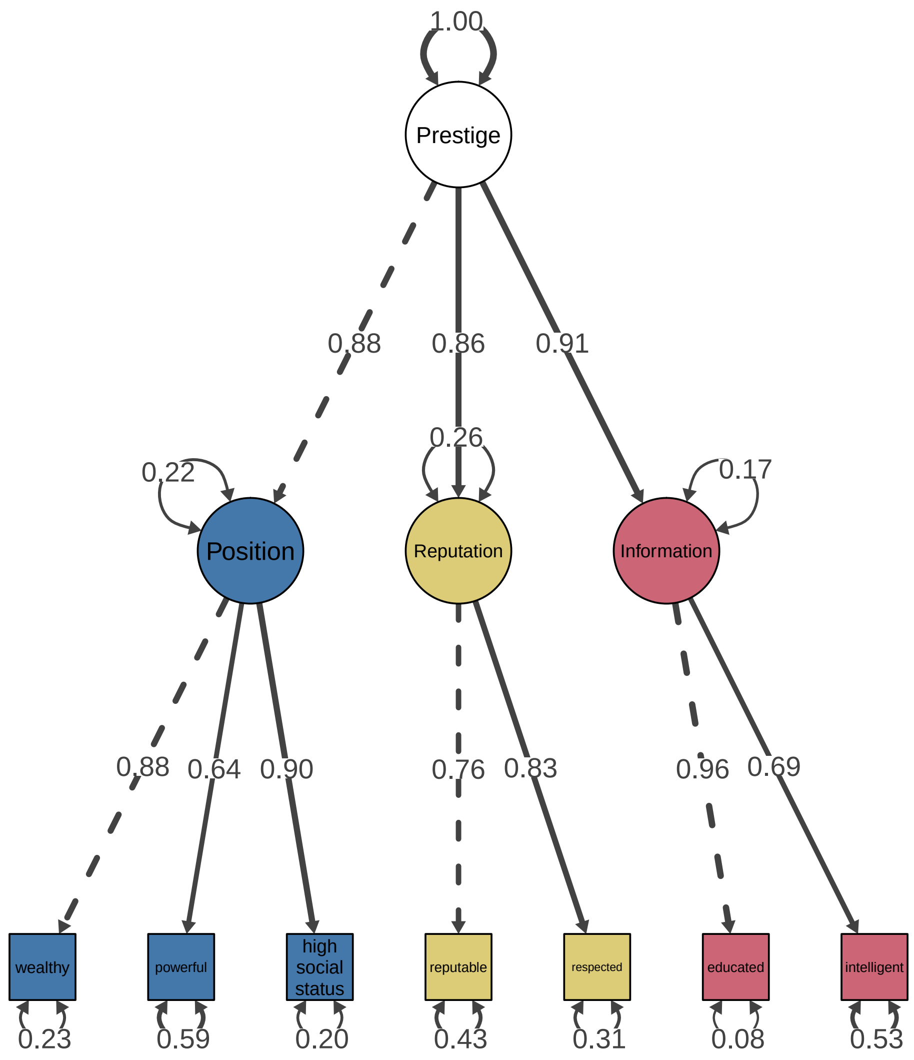


```
# dev.off()

#   Save CFA model
# saveRDS(cfa.complex, "cfa_pri.RDS")

#   Calculate predicted values (factor scores) for individual prestige
#       and PRI subscales
data.r = cbind(data.c, lavPredict(cfa.complex, newdata=data.c, method="EBM"))

#   Plot distributions of factor scores of speakers for individual prestige
#       Individual Prestige
data.r.prestige = summarySE(data.r, "prestige", "accent")
```

```
plot.r.prestige = ggplot(data.r, aes(x=accent, y=prestige)) +
    geom_violin() +
    geom_jitter(size=0.4, alpha=0.1, width=0.2, height=0.1, show.legend=F) +
    geom_errorbar(data=data.r.prestige,
                  aes(ymin=prestige-ci, ymax=prestige+ci),
                  width=0.75, size=0.5) +
    ylim(c(-3,3)) +
    theme(axis.text.x = element_text(angle = 45, vjust = 1, hjust=1))
plot.r.prestige
```

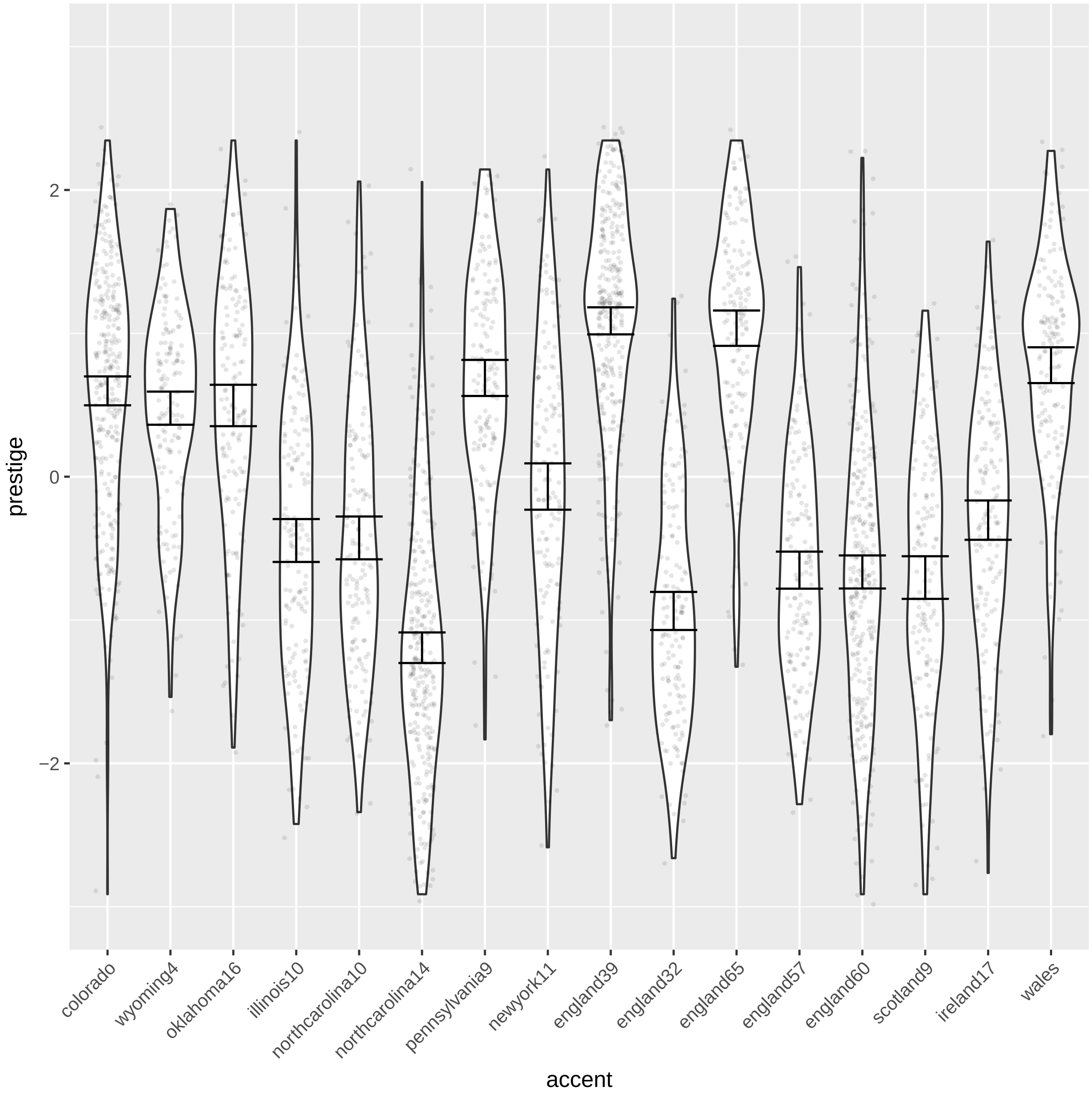


```
#   Plot distributions of factor scores of speakers PRI subscales
#       Position
data.r.position = summarySE(data.r, "position", "accent")
```

```
plot.r.position = ggplot(data.r, aes(x=accent, y=position)) +
    geom_violin() +
    geom_jitter(size=0.4, alpha=0.1, width=0.2, height=0.1, show.legend=F) +
    geom_errorbar(data=data.r.position,
                  aes(ymin=position-ci, ymax=position+ci),
                  width=0.75, size=0.5) +
    ylim(c(-3,3)) +
    labs(x="Accent", y="Position") +
    theme(axis.text.x = element_text(angle = 45, vjust = 1, hjust=1))

#       Reputation
data.r.reputation = summarySE(data.r, "reputation", "accent")

plot.r.reputation = ggplot(data.r, aes(x=accent, y=reputation)) +
    geom_violin() +
    geom_jitter(size=0.4, alpha=0.1, width=0.2, height=0.1, show.legend=F) +
    geom_errorbar(data=data.r.reputation,
                  aes(ymin=reputation-ci, ymax=reputation+ci),
                  width=0.75, size=0.5) +
    ylim(c(-3,3)) +
    labs(x="Accent", y="Reputation") +
    theme(axis.text.x = element_text(angle = 45, vjust = 1, hjust=1))

#       Information
data.r.information = summarySE(data.r, "information", "accent")

plot.r.information = ggplot(data.r, aes(x=accent, y=information)) +
    geom_violin() +
    geom_jitter(size=0.4, alpha=0.1, width=0.2, height=0.1, show.legend=F) +
    geom_errorbar(data=data.r.information,
                  aes(ymin=information-ci, ymax=information+ci),
                  width=0.75, size=0.5) +
    ylim(c(-3,3)) +
    labs(x="Accent", y="Information") +
    theme(axis.text.x = element_text(angle = 45, vjust = 1, hjust=1))

multiplot(plot.r.position, plot.r.reputation, plot.r.information)
```

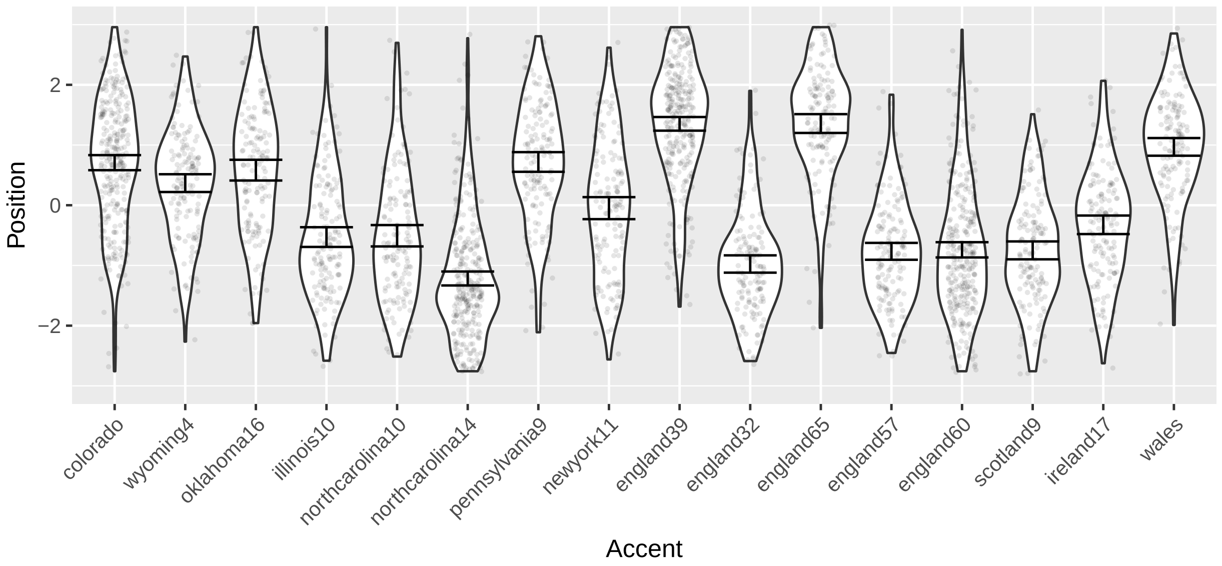
Position
Accent
colorado
wyoming4
oklahoma16
illinois10
northcarolina10
northcarolina14
pennsylvania9
newyork11
england39
england32
england65
england57
england60
scotland9
ireland17
wales
2
0
-2

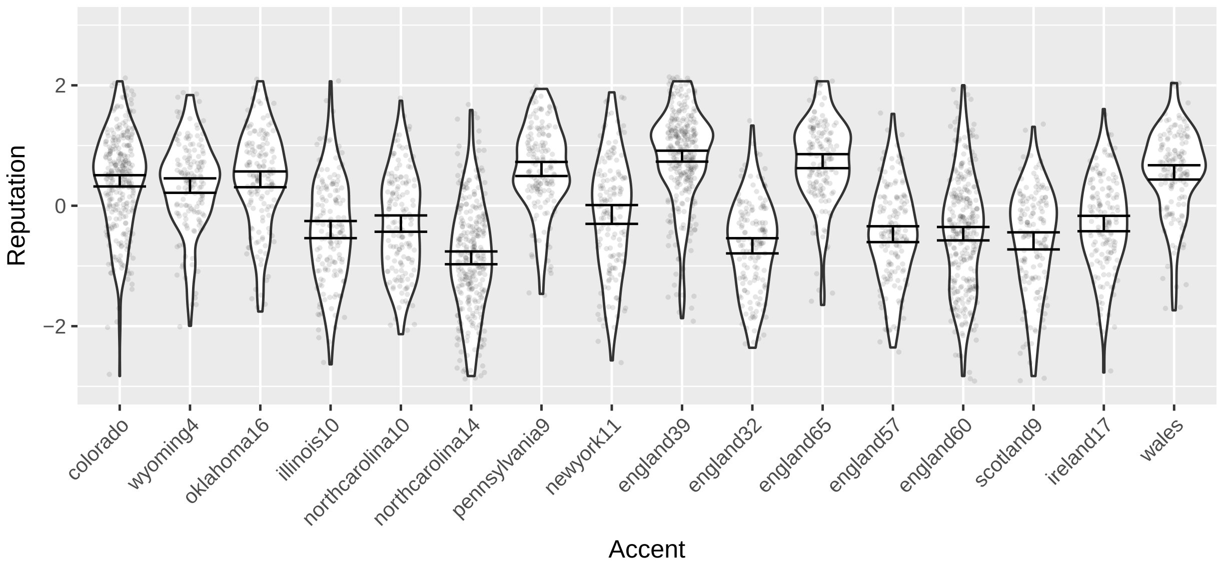
Reputation
Accent
colorado
wyoming4
oklahoma16
illinois10
northcarolina10
northcarolina14
pennsylvania9
newyork11
england39
england32
england65
england57
england60
scotland9
ireland17
wales
2
0
-2

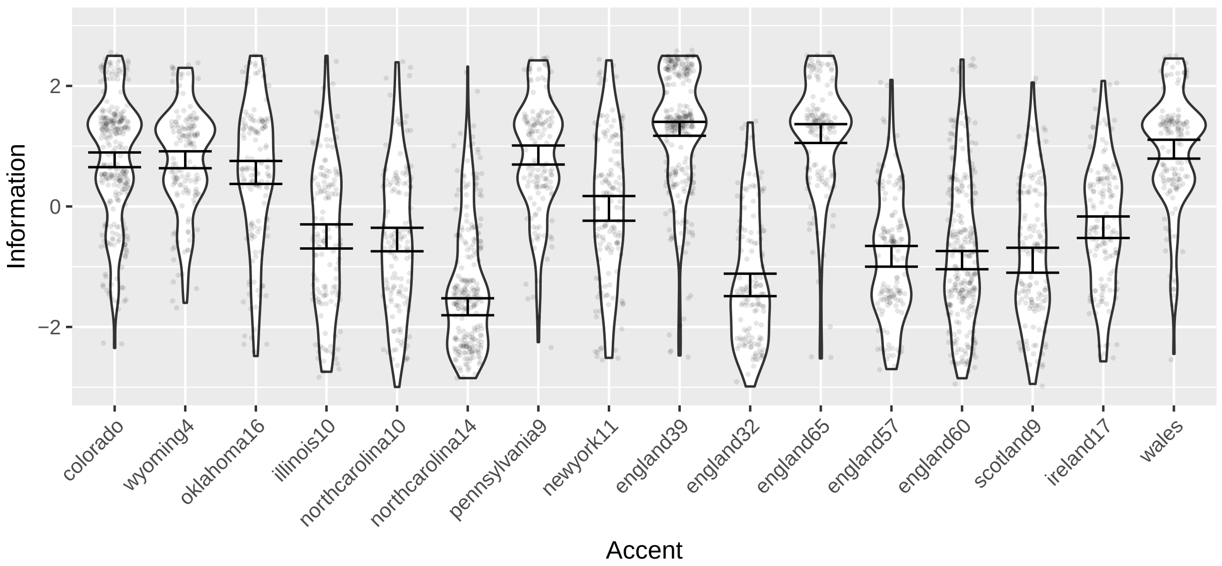
Information
Accent
colorado
wyoming4
oklahoma16
illinois10
northcarolina10
northcarolina14
pennsylvania9
newyork11
england39
england32
england65
england57
england60
scotland9
ireland17
wales
2
0
-2

SCALE VALIDITY AND RELIABILITY

Content Validity

See text of paper and results of free listing and Study 2 exploratory factor analysis.

Construct Validity

Convergent Validity

```
#   Examine polychoric correlation matrices of PRI scale items
#       Scale construction study
poly.s.p = lavCor(data.s.o[,c(22,16,13,18,19,8,14)])
corrplot.mixed(poly.s.p, lower="ellipse", upper="number")
```

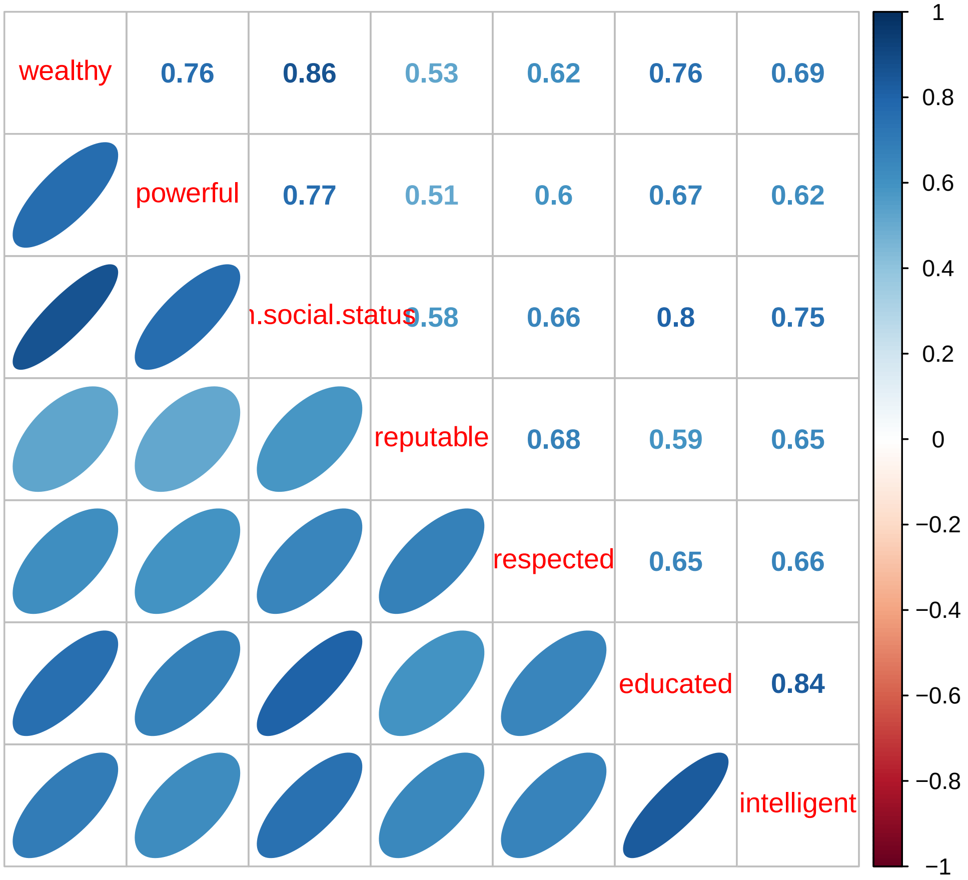


```
#       Scale evaluation study
poly.c.p = lavCor(data.c.o[,c(25,17,14,19,20,9,15)])
corrplot.mixed(poly.c.p, lower="ellipse", upper="number")
```

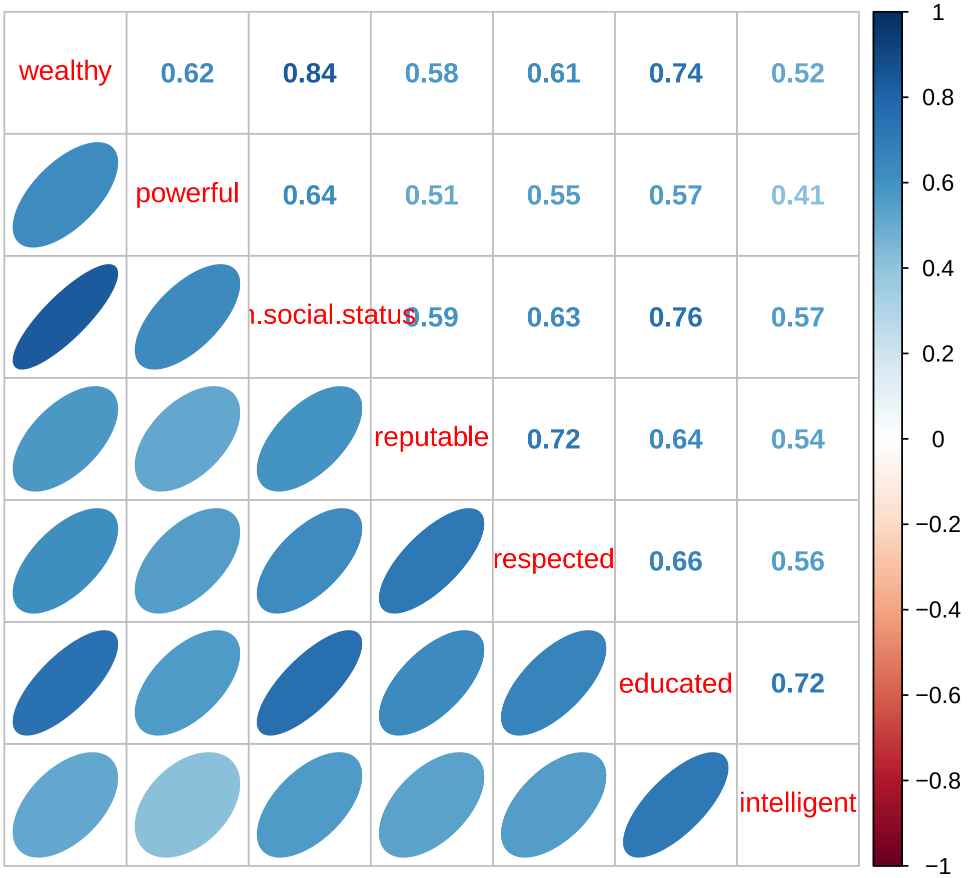


```
#       Combined data set
poly.p = lavCor(data.all.o[,c(26,18,15,20,21,10,16)])
corrplot.mixed(poly.p, lower="ellipse", upper="number")
```

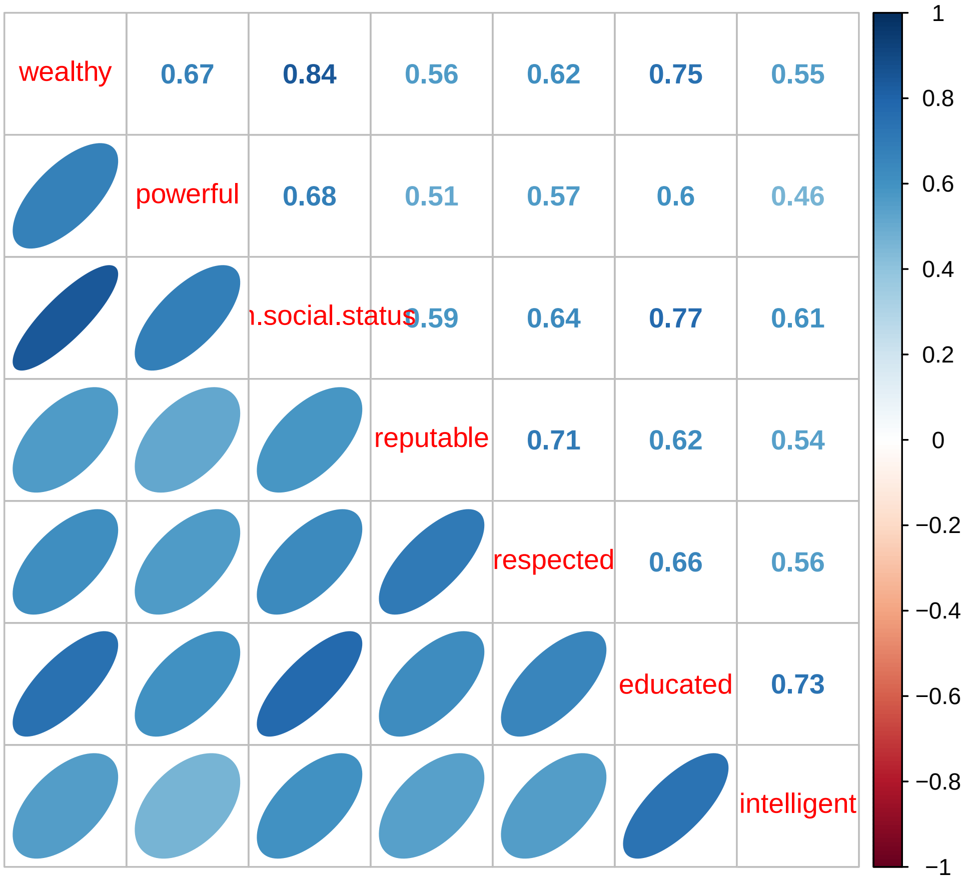


```
#   Get mean and standard deviation polychoric correlations for items
#       within and between each subscale
#       Mean
poly.avg.p = matrix(nrow=3, ncol=3)
colnames(poly.avg.p) = c("Position", "Reputation", "Information")
rownames(poly.avg.p) = c("Position", "Reputation", "Information")

poly.avg.p[1,1] = mean(c(poly.p[2:3,1], poly.p[3,2]))
poly.avg.p[2,1] = mean(c(poly.p[4:5,1], poly.p[4:5,2], poly.p[4:5,3]))
poly.avg.p[3,1] = mean(c(poly.p[6:7,1], poly.p[6:7,2], poly.p[6:7,3]))
poly.avg.p[2,2] = poly.p[5,4]
poly.avg.p[3,2] = mean(c(poly.p[6:7,4], poly.p[6:7,5]))
```

```
poly.avg.p[3,3] = poly.p[7,6]

round(poly.avg.p, 3)
```

```
##              Position Reputation Information
## Position        0.733         NA          NA
## Reputation      0.580      0.707          NA
## Information     0.625      0.594       0.733
```

```
#       Standard deviation
poly.std.p = matrix(nrow=3, ncol=3)
colnames(poly.std.p) = c("Position", "Reputation", "Information")
rownames(poly.std.p) = c("Position", "Reputation", "Information")

poly.std.p[1,1] = sd(c(poly.p[2:3,1], poly.p[3,2]))
poly.std.p[2,1] = sd(c(poly.p[4:5,1], poly.p[4:5,2], poly.p[4:5,3]))
poly.std.p[3,1] = sd(c(poly.p[6:7,1], poly.p[6:7,2], poly.p[6:7,3]))
poly.std.p[2,2] = NA
poly.std.p[3,2] = sd(c(poly.p[6:7,4], poly.p[6:7,5]))
poly.std.p[3,3] = NA

round(poly.std.p, 3)
```

```
##              Position Reputation Information
## Position        0.097         NA          NA
## Reputation      0.043         NA          NA
## Information     0.118      0.054          NA
```

```
#   Mean polychoric correlation across all PRI items
mean(unique(poly.p[poly.p != 1]))
```

```
## [1] 0.6308894
```

```
#   Standard deviation polychoric correlation across all PRI items
sd(unique(poly.p[poly.p != 1]))
```

```
## [1] 0.0935712
```

```
#   Average variance explained ("AVE")
reliability(cfa.complex)[5,]
```

```
##    position  reputation information
##   0.6748337   0.6301976   0.6987883
```

Discriminant Validity

```
#   Heterotrait-monotrait ratio of correlations ("HTMT")
htmt.c = htmt(data.c.o[,c(25,17,14,19,20,9,15,
              11,16,12,24,6,
              3,2,7,10)],
              cfa.model2)
```

```r
colnames(htmt.c) = c("Position", "Reputation", "Information",
                     "Solidarity", "Dynamism")
rownames(htmt.c) = c("Position", "Reputation", "Information",
                     "Solidarity", "Dynamism")
htmt.c[lower.tri(htmt.c) == F] = NA

round(htmt.c, 3)
```

```
##             Positn Repttn Infrmt Sldrty Dynmsm
## Position        NA
## Reputation  0.818     NA
## Information 0.841  0.835     NA
## Solidarity  0.086  0.442  0.246     NA
## Dynamism    0.727  0.773  0.735  0.670     NA
```

Criterion Validity

```r
#   Polychoric correlations between 'prestigious' and PRI items
crit.r.poly = lavCor(data.r[,c(18,17,14,25,19,20,9,15)],
                     ordered=colnames(data.r[,c(18,17,14,25,19,20,9,15)]))
crit.r.poly[-1,1]
```

```
##          powerful high.social.status            wealthy
##         0.6368356          0.8072758          0.7984209
##         reputable          respected           educated
##         0.6077425          0.6444256          0.7283810
##       intelligent
##         0.5259480
```

```r
#       Mean and standard deviation correlation of all PRI items
mean(crit.r.poly[-1,1])
```

```
## [1] 0.6784328
```

```r
sd(crit.r.poly[-1,1])
```

```
## [1] 0.103785
```

```r
#       Mean and standard deviation correlation of Position items
mean(crit.r.poly[c(2:4),1])
```

```
## [1] 0.7475107
```

```r
sd(crit.r.poly[c(2:4),1])
```

```
## [1] 0.09594969
```

```r
#       Mean and standard deviation correlation of Reputation items
mean(crit.r.poly[c(5:6),1])
```

```
## [1] 0.6260841
```

```
sd(crit.r.poly[c(5:6),1])

## [1] 0.02593888

#       Mean and standard deviation correlation of Information items
mean(crit.r.poly[c(7:8),1])

## [1] 0.6271645

sd(crit.r.poly[c(7:8),1])

## [1] 0.1431418

#   Polychoric correlations between 'prestigious' and PRI factor scores
crit.fac.poly = lavCor(data.r[,c(18,40,41,42,43)], ordered=colnames(data.r)[18])
crit.fac.poly[-1,1]

##    prestige    position  reputation information
##   0.8149960   0.8444166   0.7637328   0.7451528
```

Comparative Study

```
#   Outlier Removal

#   Check for multivariate outliers
head(mvOutlier(data.v[,c(5:11,21:37)], qqplot=T)$outlier, 10)
```

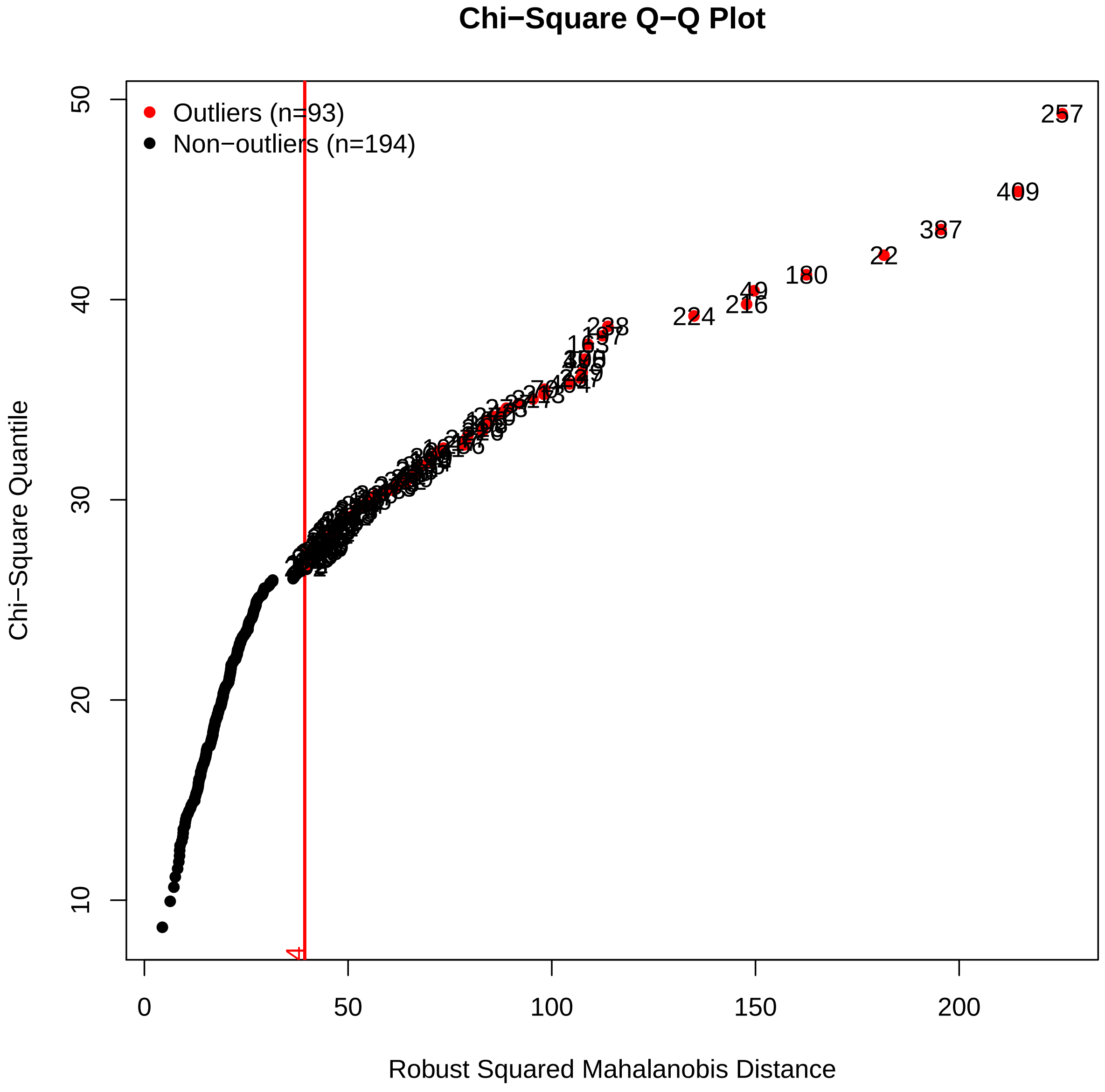


```
##      Observation Mahalanobis Distance Outlier
## 257          257              225.277    TRUE
## 409          409              214.449    TRUE
## 387          387              195.540    TRUE
## 22            22              181.527    TRUE
## 180          180              162.504    TRUE
## 49            49              149.620    TRUE
## 216          216              147.827    TRUE
## 224          224              134.909    TRUE
## 238          238              113.777    TRUE
## 197          197              112.461    TRUE
```

```
# Loop to find rows of outlier observations for removal (warning: LONG,
```

```
#           uncomment to run)
# to.remove = as.numeric(as.character(mvOutlier(data.v[,c(5:11,21:37)],
#                                               qqplot=T)$outlier[1:6,1]))
# lines.to.remove = c()
# lines.progress = txtProgressBar(min=0, max=nrow(data.v), style=3, width=100)
# for (i in 1:nrow(data.v)) {
#     x = as.numeric(as.character(mvOutlier(data.v[-i,c(5:11,21:37)], qqplot=F,
#                                           alpha=0.6)$outlier[1:10,1]))
#     if (!all(to.remove %in% x)) {
#         lines.to.remove = append(lines.to.remove, i)
#     }
#     setTxtProgressBar(lines.progress, i)
# }
# close(lines.progress)
# lines.to.remove

#       Result: 22  49 153 194 260 282

#       Check with results removed
head(mvOutlier(data.v[-c(22,49,153,194,260,282),c(5:11,21:37)],
               qqplot=T)$outlier, 10)
```

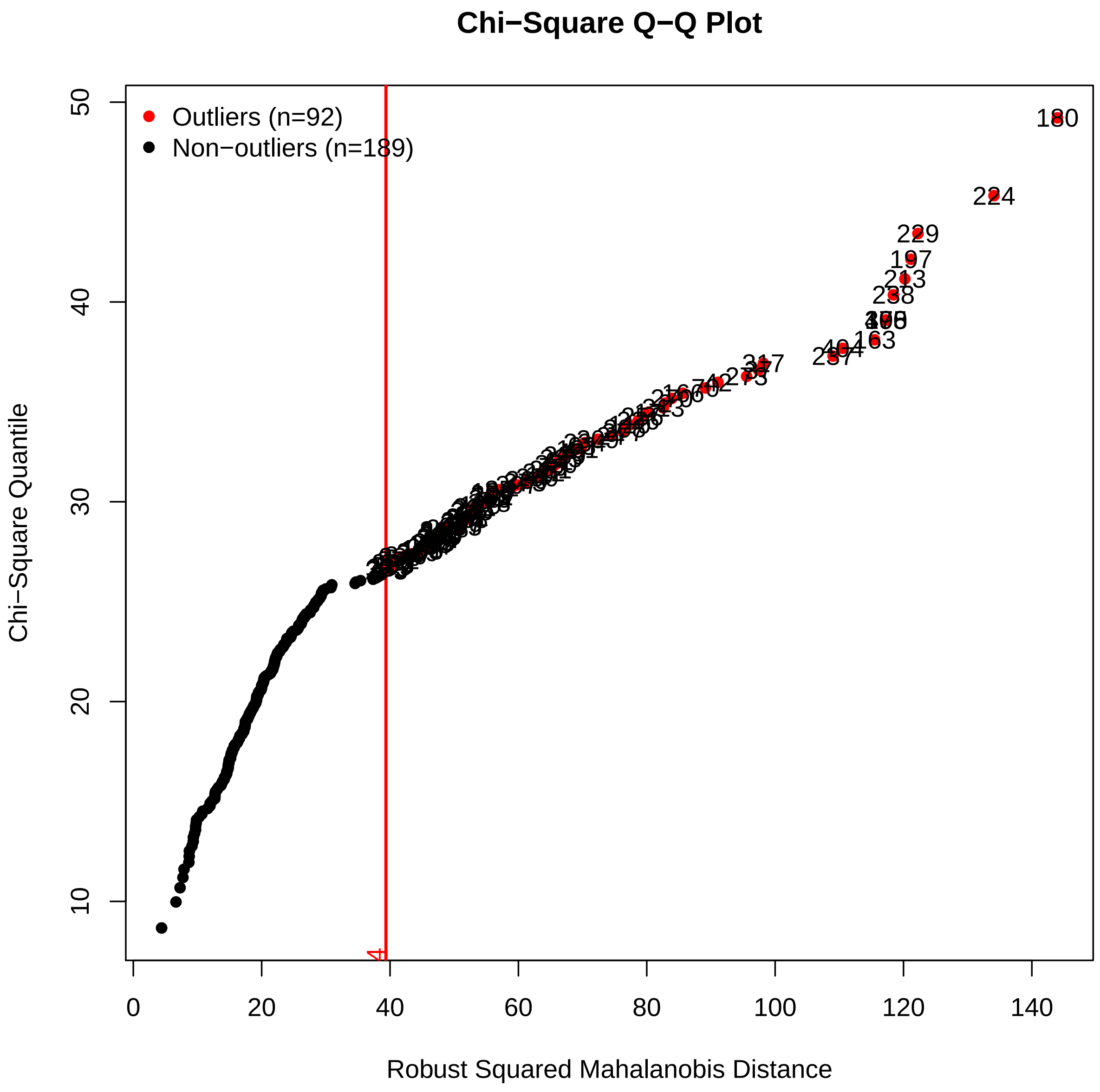


```
##      Observation Mahalanobis Distance Outlier
## 180          180              143.971    TRUE
## 224          224              134.097    TRUE
## 229          229              122.245    TRUE
## 197          197              121.167    TRUE
## 213          213              120.212    TRUE
## 238          238              118.420    TRUE
## 198          198              117.268    TRUE
## 370          370              117.268    TRUE
## 405          405              117.268    TRUE
## 163          163              115.491    TRUE
```

```
#       Identify participants
```

```
data.v[c(22,49,153,194,260,282),]

##                 id accent country prestigious wealthy powerful
## 22  1502635812927   high      us           6       6        6
## 49  1502749845669   high      us           5       5        4
## 216 1502509622551    low      us           4       4        5
## 257 1502751645097    low      us           1       1        2
## 387 1503184321567    low      uk           6       5        5
## 409 1503571948433    low      uk           4       4        4
##     high.social.status reputable respected educated intelligent friendly
## 22                   6         6         6        6           6        4
## 49                   5         5         5        5           5        6
## 216                  3         5         6        5           5        6
## 257                  1         3         5        2           2        6
## 387                  5         5         5        6           6        3
## 409                  1         4         5        4           4        4
##     kind good.natured warm comforting aggressive active confident
## 22     6            4    3          6          2      2         6
## 49     5            6    5          5          1      5         5
## 216    6            6    6          6          2      1         6
## 257    6            6    6          6          1      6         6
## 387    6            5    4          4          1      3         2
## 409    4            5    5          4          1      4         5
##     enthusiastic P.People.respect.and.admire.him
## 22             3                               6
## 49             5                               1
## 216            6                               5
## 257            6                               5
## 387            2                               6
## 409            4                               6
##     P.People.do.NOT.want.to.be.like.him
## 22                                    6
## 49                                    6
## 216                                   6
## 257                                   6
## 387                                   3
## 409                                   6
##     P.People.always.expect.him.to.be.successful
## 22                                            1
## 49                                            1
## 216                                           1
## 257                                           1
## 387                                           6
## 409                                           4
##     P.People.do.NOT.value.his.opinion
## 22                                  6
## 49                                  6
## 216                                 6
```

```
## 257                               6
## 387                               6
## 409                               6
##     P.He.is.held.in.high.esteem.by.people
## 22                                    6
## 49                                    5
## 216                                   1
## 257                                   1
## 387                                   6
## 409                                   1
##     P.His.unique.talents.and.abilities.are.recognized.by.other.people
## 22                                                                  6
## 49                                                                  1
## 216                                                                 4
## 257                                                                 6
## 387                                                                 4
## 409                                                                 1
##     P.He.is.considered.an.expert.on.some.matters.by.people
## 22                                                       6
## 49                                                       4
## 216                                                      1
## 257                                                      2
## 387                                                      1
## 409                                                      5
##     P.People.seek.his.advice.on.a.variety.of.matters
## 22                                                 5
## 49                                                 5
## 216                                                4
## 257                                                2
## 387                                                5
## 409                                                5
##     P.Other.people.do.NOT.enjoy.hanging.out.with.him
## 22                                                 6
## 49                                                 6
## 216                                                6
## 257                                                6
## 387                                                3
## 409                                                6
##     D.He.enjoys.having.control.over.other.people
## 22                                             4
## 49                                             2
## 216                                            1
## 257                                            1
## 387                                            1
## 409                                            1
##     D.He.often.tries.to.get.his.own.way.regardless.of.what.other.people.may.want
## 22                                                                             6
## 49                                                                             1
```

```
## 216                                                                                    1
## 257                                                                                    5
## 387                                                                                    6
## 409                                                                                    1
##     D.He.is.willing.to.use.aggressive.tactics.to.get.his.way
## 22                                                         1
## 49                                                         1
## 216                                                        1
## 257                                                        2
## 387                                                        5
## 409                                                        1
##     D.He.tries.to.control.others.rather.than.permit.them.to.control.him
## 22                                                                    6
## 49                                                                    1
## 216                                                                   1
## 257                                                                   4
## 387                                                                   5
## 409                                                                   1
##     D.He.does.NOT.have.a.forceful.or.dominant.personality
## 22                                                      1
## 49                                                      6
## 216                                                     6
## 257                                                     6
## 387                                                     6
## 409                                                     6
##     D.People.know.it.is.better.to.let.him.have.his.way
## 22                                                   4
## 49                                                   3
## 216                                                  1
## 257                                                  3
## 387                                                  6
## 409                                                  1
##     D.He.does.NOT.enjoy.having.authority.over.other.people
## 22                                                       6
## 49                                                       4
## 216                                                      1
## 257                                                      6
## 387                                                      6
## 409                                                      6
##     D.People.are.afraid.of.him
## 22                           4
## 49                           1
## 216                          1
## 257                          1
## 387                          5
## 409                          1
```

```
data.v.to.remove = as.character(unique(data.v$id[c(22,49,153,194,260,282)]))
```

```
#       Remove participants
data.v = data.v[!as.character(data.v$id) %in% data.v.to.remove,]
data.v.o = data.v.o[!as.character(data.v.o$id) %in% data.v.to.remove,]
data.v.l = data.v.l[!as.character(data.v.l$id) %in% data.v.to.remove,]

#   Polychoric correlations
data.v.poly = lavCor(data.v[,c(4:37)])
colnames(data.v.poly)[18:34] = abbreviate(gsub("\\.", "",
                                               colnames(data.v.poly)[18:34]), 18)
rownames(data.v.poly)[18:34] = abbreviate(gsub("\\.", "",
                                               rownames(data.v.poly)[18:34]), 18)
data.v.poly
```

```
##                    prstgs welthy powrfl hgh.s. reptbl rspctd eductd intllg
## prestigious         1.000
## wealthy             0.842  1.000
## powerful            0.749  0.688  1.000
## high.social.status  0.872  0.859  0.741  1.000
## reputable           0.719  0.624  0.615  0.692  1.000
## respected           0.760  0.678  0.684  0.728  0.783  1.000
## educated            0.810  0.780  0.690  0.814  0.737  0.800  1.000
## intelligent         0.772  0.735  0.662  0.769  0.784  0.812  0.852  1.000
## friendly           -0.069 -0.089 -0.002 -0.099  0.210  0.183  0.067  0.161
## kind               -0.024 -0.058 -0.016 -0.079  0.241  0.211  0.075  0.169
## good.natured       -0.044 -0.057  0.019 -0.058  0.238  0.201  0.101  0.176
## warm               -0.074 -0.090 -0.042 -0.098  0.207  0.172  0.033  0.145
## comforting          0.168  0.179  0.172  0.157  0.405  0.343  0.280  0.321
## aggressive          0.359  0.365  0.408  0.364  0.230  0.317  0.239  0.230
## active              0.205  0.164  0.303  0.173  0.386  0.384  0.317  0.370
## confident           0.582  0.559  0.588  0.600  0.696  0.700  0.653  0.724
## enthusiastic       -0.009 -0.036  0.108 -0.066  0.187  0.228  0.090  0.167
## PPeoplrspctnddmrhm  0.666  0.617  0.598  0.631  0.728  0.735  0.715  0.750
## PPepldNOTwnttblkhm  0.404  0.393  0.292  0.409  0.453  0.441  0.459  0.439
## PPpllwysxpcthmtbsc  0.804  0.738  0.684  0.765  0.707  0.725  0.784  0.763
## PPeopledNOTvlhspnn  0.525  0.508  0.396  0.518  0.535  0.508  0.568  0.496
## PHshldnhghstmbyppl  0.697  0.677  0.606  0.663  0.732  0.734  0.727  0.753
## PHsnqtlntsndbltsrr  0.640  0.594  0.533  0.577  0.692  0.684  0.665  0.721
## PHscnsdrdnxprtnsmm  0.748  0.699  0.627  0.704  0.729  0.742  0.785  0.775
## PPplskhsdvcnvrtyfm  0.630  0.620  0.532  0.604  0.722  0.714  0.694  0.735
## POthrppldNOTnjyhng -0.006 -0.020 -0.056 -0.023  0.161  0.092  0.053  0.082
## DHnjyshvngcntrlvrt  0.509  0.455  0.544  0.505  0.319  0.391  0.442  0.379
## DHftntrstgthswnwyr  0.414  0.363  0.452  0.426  0.276  0.335  0.365  0.327
## DHswllngtsggrssvtc  0.400  0.390  0.480  0.438  0.221  0.335  0.321  0.317
## DHtrstcntrlthrsrth  0.480  0.438  0.541  0.527  0.302  0.363  0.446  0.392
## DHdsNOThvfrcflrdmn  0.360  0.337  0.378  0.432  0.201  0.218  0.302  0.263
## DPplknwtsbttrtlthm  0.468  0.420  0.528  0.492  0.335  0.368  0.405  0.372
## DHdsNOTnjyhvngthrt  0.366  0.324  0.288  0.392  0.096  0.211  0.269  0.230
## DPeopleareafradfhm  0.301  0.316  0.390  0.326  0.126  0.175  0.203  0.182
```

```
##                     frndly kind   gd.ntr warm   cmfrtn aggrss active cnfdnt
## prestigious
## wealthy
## powerful
## high.social.status
## reputable
## respected
## educated
## intelligent
## friendly             1.000
## kind                 0.805  1.000
## good.natured         0.796  0.754  1.000
## warm                 0.762  0.792  0.753  1.000
## comforting           0.630  0.678  0.615  0.715  1.000
## aggressive          -0.203 -0.144 -0.097 -0.128 -0.112  1.000
## active               0.500  0.514  0.503  0.515  0.452  0.163  1.000
## confident            0.322  0.315  0.312  0.309  0.426  0.250  0.454  1.000
## enthusiastic         0.616  0.586  0.611  0.636  0.522  0.054  0.616  0.345
## PPeoplrspctnddmrhm   0.251  0.306  0.254  0.275  0.476  0.108  0.400  0.667
## PPepldNOTwnttblkhm  -0.005 -0.011  0.005  0.073  0.270 -0.048  0.222  0.379
## PPpllwysxpcthmtbsc  -0.035  0.020 -0.047 -0.019  0.224  0.263  0.254  0.659
## PPeopledNOTvlhspnn  -0.085 -0.018 -0.049 -0.015  0.214  0.042  0.141  0.439
## PHshldnhghstmbyppl   0.188  0.202  0.195  0.203  0.391  0.201  0.379  0.665
## PHsnqtlntsndbltsrr   0.256  0.231  0.239  0.240  0.404  0.150  0.381  0.637
## PHscnsdrdnxprtnsmm   0.105  0.115  0.080  0.087  0.318  0.227  0.323  0.665
## PPplskhsdvcnvrtyfm   0.238  0.263  0.240  0.274  0.431  0.106  0.387  0.657
## POthrppldNOTnjyhng   0.327  0.338  0.354  0.380  0.398 -0.241  0.254  0.170
## DHnjyshvngcntrlvrt  -0.299 -0.287 -0.269 -0.337 -0.174  0.412 -0.008  0.346
## DHftntrstgthswnwyr  -0.241 -0.215 -0.245 -0.256 -0.175  0.453  0.057  0.289
## DHswllngtsggrssvtc  -0.305 -0.300 -0.248 -0.292 -0.190  0.557 -0.042  0.269
## DHtrstcntrlthrsrth  -0.284 -0.309 -0.277 -0.276 -0.172  0.441  0.012  0.323
## DHdsNOThvfrcflrdmn  -0.440 -0.435 -0.392 -0.398 -0.295  0.310 -0.077  0.175
## DPplknwtsbttrtlthm  -0.172 -0.186 -0.156 -0.182 -0.118  0.381  0.037  0.284
## DHdsNOTnjyhvngthrt  -0.495 -0.495 -0.482 -0.462 -0.344  0.248 -0.160  0.126
## DPeopleareafradfhm  -0.317 -0.332 -0.284 -0.326 -0.298  0.509 -0.042  0.163
##                     enthss PPplrs PPpldNOTw PPpllw PPpldNOTv PHshld PHsnqt
## prestigious
## wealthy
## powerful
## high.social.status
## reputable
## respected
## educated
## intelligent
## friendly
## kind
## good.natured
## warm
```

```
## comforting
## aggressive
## active
## confident
## enthusiastic         1.000
## PPeoplrspctnddmrhm  0.273  1.000
## PPepldNOTwnttblkhm  0.085  0.544  1.000
## PPpllwysxpcthmtbsc  0.029  0.752  0.484      1.000
## PPeopledNOTvlhspnn  0.011  0.595  0.698      0.589  1.000
## PHshldnhghstmbyppl  0.220  0.845  0.533      0.741  0.568      1.000
## PHsnqtlntsndbltsrr  0.301  0.763  0.500      0.723  0.536      0.747  1.000
## PHscnsdrdnxprtnsmm  0.134  0.793  0.480      0.771  0.557      0.806  0.720
## PPplskhsdvcnvrtyfm  0.240  0.809  0.507      0.709  0.551      0.818  0.734
## POthrppldNOTnjyhng  0.209  0.246  0.457      0.070  0.395      0.225  0.209
## DHnjyshvngcntrlvrt -0.128  0.281  0.047      0.507  0.167      0.322  0.294
## DHftntrstgthswnwyr -0.078  0.198 -0.059      0.448  0.029      0.273  0.202
## DHswllngtsggrssvtc -0.130  0.156 -0.047      0.348  0.001      0.204  0.152
## DHtrstcntrlthrsrth -0.125  0.295  0.056      0.500  0.150      0.319  0.263
## DHdsNOThvfrcflrdmn -0.257  0.112  0.217      0.305  0.329      0.144  0.091
## DPplknwtsbttrtlthm -0.051  0.314  0.014      0.466  0.089      0.349  0.262
## DHdsNOTnjyhvngthrt -0.295  0.044  0.207      0.328  0.262      0.139  0.073
## DPeopleareafradfhm -0.063  0.054 -0.149      0.283 -0.060      0.069  0.042
##                    PHscns PPplsk POtNOT DHnjys DHftnt DHswll DHtrst
## prestigious
## wealthy
## powerful
## high.social.status
## reputable
## respected
## educated
## intelligent
## friendly
## kind
## good.natured
## warm
## comforting
## aggressive
## active
## confident
## enthusiastic
## PPeoplrspctnddmrhm
## PPepldNOTwnttblkhm
## PPpllwysxpcthmtbsc
## PPeopledNOTvlhspnn
## PHshldnhghstmbyppl
## PHsnqtlntsndbltsrr
## PHscnsdrdnxprtnsmm  1.000
## PPplskhsdvcnvrtyfm  0.796  1.000
```

```
## POthrppldNOTnjyhng  0.069  0.263  1.000
## DHnjyshvngcntrlvrt  0.421  0.309 -0.378  1.000
## DHftntrstgthswnwyr  0.351  0.245 -0.418  0.744  1.000
## DHswllngtsggrssvtc  0.266  0.181 -0.429  0.707  0.702  1.000
## DHtrstcntrlthrsrth  0.453  0.309 -0.341  0.704  0.743  0.691  1.000
## DHdsNOThvfrcflrdmn  0.224  0.142 -0.159  0.491  0.393  0.466  0.530
## DPplknwtsbttrtlthm  0.418  0.335 -0.305  0.677  0.698  0.638  0.738
## DHdsNOTnjyhvngthrt  0.216  0.101 -0.156  0.507  0.435  0.401  0.482
## DPeopleareafradfhm  0.168  0.055 -0.482  0.588  0.620  0.650  0.627
##                    DHdsNOTh DPplkn DHdsNOTn DPplrf
## prestigious
## wealthy
## powerful
## high.social.status
## reputable
## respected
## educated
## intelligent
## friendly
## kind
## good.natured
## warm
## comforting
## aggressive
## active
## confident
## enthusiastic
## PPeoplrspctnddmrhm
## PPepldNOTwnttblkhm
## PPpllwysxpcthmtbsc
## PPeopledNOTvlhspnn
## PHshldnhghstmbyppl
## PHsnqtlntsndbltsrr
## PHscnsdrdnxprtnsmm
## PPplskhsdvcnvrtyfm
## POthrppldNOTnjyhng
## DHnjyshvngcntrlvrt
## DHftntrstgthswnwyr
## DHswllngtsggrssvtc
## DHtrstcntrlthrsrth
## DHdsNOThvfrcflrdmn  1.000
## DPplknwtsbttrtlthm  0.445    1.000
## DHdsNOTnjyhvngthrt  0.668    0.376  1.000
## DPeopleareafradfhm  0.418    0.605  0.355    1.000
```

```
#   List of unabbreviated Cheng et al. (2010) items
colnames(data.v)[21:37]
```

```
##  [1] "P.People.respect.and.admire.him"
```

```
##  [2] "P.People.do.NOT.want.to.be.like.him"
##  [3] "P.People.always.expect.him.to.be.successful"
##  [4] "P.People.do.NOT.value.his.opinion"
##  [5] "P.He.is.held.in.high.esteem.by.people"
##  [6] "P.His.unique.talents.and.abilities.are.recognized.by.other.people"
##  [7] "P.He.is.considered.an.expert.on.some.matters.by.people"
##  [8] "P.People.seek.his.advice.on.a.variety.of.matters"
##  [9] "P.Other.people.do.NOT.enjoy.hanging.out.with.him"
## [10] "D.He.enjoys.having.control.over.other.people"
## [11] "D.He.often.tries.to.get.his.own.way.regardless.of.what.other.people.may.want"
## [12] "D.He.is.willing.to.use.aggressive.tactics.to.get.his.way"
## [13] "D.He.tries.to.control.others.rather.than.permit.them.to.control.him"
## [14] "D.He.does.NOT.have.a.forceful.or.dominant.personality"
## [15] "D.People.know.it.is.better.to.let.him.have.his.way"
## [16] "D.He.does.NOT.enjoy.having.authority.over.other.people"
## [17] "D.People.are.afraid.of.him"
```

```
#   Mean and standard deviation polychoric correlation between PRI items and
#       Cheng et al. (2010) prestige items
mean(c(data.v.poly[c(2:8),c(18:26)]))
```

```
## [1] 0.5663193
```

```
sd(c(data.v.poly[c(2:8),c(18:26)]))
```

```
## [1] 0.2206011
```

```
#       With reversed Cheng et al. (2010) items removed
mean(c(data.v.poly[c(2:8),c(18,20,22:25)]))
```

```
## [1] 0.6898725
```

```
sd(c(data.v.poly[c(2:8),c(18,20,22:25)]))
```

```
## [1] 0.06615521
```

```
#   Fit model for non-prestige domains to scale evaluation data
soldyn.fit = cfa(cfa.model3, data.c, estimator="MLMVS")
soldyn.complex = lavaan.survey(soldyn.fit, cfa.design, estimator="MLMVS")

#   Fit model for Cheng et al. (2010) prestige and dominance domains
#       to comparative data
#       Define model
cheng.model1 = "
C.prestige             =~ P.People.respect.and.admire.him +
    P.People.do.NOT.want.to.be.like.him +
    P.People.always.expect.him.to.be.successful +
    P.People.do.NOT.value.his.opinion +
    P.He.is.held.in.high.esteem.by.people +
    P.His.unique.talents.and.abilities.are.recognized.by.other.people +
    P.He.is.considered.an.expert.on.some.matters.by.people +
    P.People.seek.his.advice.on.a.variety.of.matters +
```

```
    P.Other.people.do.NOT.enjoy.hanging.out.with.him

C.dominance            =~ D.He.enjoys.having.control.over.other.people +
    D.He.often.tries.to.get.his.own.way.regardless.of.what.other.people.may.want +
    D.He.is.willing.to.use.aggressive.tactics.to.get.his.way +
    D.He.tries.to.control.others.rather.than.permit.them.to.control.him +
    D.He.does.NOT.have.a.forceful.or.dominant.personality +
    D.People.know.it.is.better.to.let.him.have.his.way +
    D.He.does.NOT.enjoy.having.authority.over.other.people +
    D.People.are.afraid.of.him
"

#       Fit CFA model
cheng.fit = cfa(cheng.model1, data.v.o, estimator="WLSMV")
summary(cheng.fit, fit.measures=T, ci=T)
```

```
## lavaan (0.5-23.1097) converged normally after  39 iterations
##
##   Number of observations                           275
##
##   Estimator                                       DWLS      Robust
##   Minimum Function Test Statistic             1987.133    1808.913
##   Degrees of freedom                               118         118
##   P-value (Chi-square)                           0.000       0.000
##   Scaling correction factor                                  1.141
##   Shift parameter                                           67.654
##     for simple second-order correction (Mplus variant)
##
## Model test baseline model:
##
##   Minimum Function Test Statistic            45947.150   13706.626
##   Degrees of freedom                               136         136
##   P-value                                        0.000       0.000
##
## User model versus baseline model:
##
##   Comparative Fit Index (CFI)                    0.959       0.875
##   Tucker-Lewis Index (TLI)                       0.953       0.856
##
##   Robust Comparative Fit Index (CFI)                            NA
##   Robust Tucker-Lewis Index (TLI)                               NA
##
## Root Mean Square Error of Approximation:
##
##   RMSEA                                          0.240       0.229
##   90 Percent Confidence Interval          0.231  0.250       0.219  0.238
##   P-value RMSEA <= 0.05                          0.000       0.000
##
```

```
##   Robust RMSEA                                                  NA
##   90 Percent Confidence Interval                                NA       NA
##
## Standardized Root Mean Square Residual:
##
##   SRMR                                         0.154       0.154
##
## Weighted Root Mean Square Residual:
##
##   WRMR                                         2.999       2.999
##
## Parameter Estimates:
##
##   Information                               Expected
##   Standard Errors                         Robust.sem
##
## Latent Variables:
##                    Estimate  Std.Err  z-value  P(>|z|) ci.lower ci.upper
##   C.prestige =~
##     P.Ppl.rspct...    1.000                               1.000    1.000
##     P.Pp..NOT.....    0.700    0.030   23.481    0.000    0.641    0.758
##     P.Ppl.lwy.....    0.942    0.016   57.986    0.000    0.910    0.974
##     P.Ppl.d.NOT...    0.796    0.027   29.719    0.000    0.743    0.848
##     P.H.s.hld.....    1.000    0.014   73.406    0.000    0.973    1.026
##     P.Hs.n........    0.894    0.019   46.685    0.000    0.857    0.932
##     P.H.s.c.......    0.980    0.014   69.049    0.000    0.953    1.008
##     P.Ppl.s.......    0.963    0.014   66.488    0.000    0.935    0.991
##     P.O...NOT.....    0.078    0.051    1.519    0.129   -0.023    0.178
##   C.dominance =~
##     D.H.njys.h....    1.000                               1.000    1.000
##     D.H...........    0.986    0.024   40.325    0.000    0.938    1.034
##     D.H.s.........    0.937    0.026   36.437    0.000    0.886    0.987
##     D.H...........    1.018    0.024   42.036    0.000    0.970    1.065
##     D.H..NOT......    0.768    0.034   22.394    0.000    0.701    0.836
##     D.Pp..........    0.953    0.026   37.265    0.000    0.903    1.003
##     D.H..NOT......    0.735    0.036   20.666    0.000    0.665    0.804
##     D.Ppl.r.frd.f.    0.842    0.031   27.237    0.000    0.782    0.903
##
## Covariances:
##                    Estimate  Std.Err  z-value  P(>|z|) ci.lower ci.upper
##   C.prestige ~~
##     C.dominance       0.297    0.037    7.955    0.000    0.224    0.371
##
## Intercepts:
##                    Estimate  Std.Err  z-value  P(>|z|) ci.lower ci.upper
##    .P.Ppl.rspct...    0.000                               0.000    0.000
##    .P.Pp..NOT.....    0.000                               0.000    0.000
##    .P.Ppl.lwy.....    0.000                               0.000    0.000
```

```
##    .P.Ppl.d.NOT...    0.000                               0.000    0.000
##    .P.H.s.hld.....    0.000                               0.000    0.000
##    .P.Hs.n........    0.000                               0.000    0.000
##    .P.H.s.c.......    0.000                               0.000    0.000
##    .P.Ppl.s.......    0.000                               0.000    0.000
##    .P.O...NOT.....    0.000                               0.000    0.000
##    .D.H.njys.h....    0.000                               0.000    0.000
##    .D.H...........    0.000                               0.000    0.000
##    .D.H.s.........    0.000                               0.000    0.000
##    .D.H...........    0.000                               0.000    0.000
##    .D.H..NOT......    0.000                               0.000    0.000
##    .D.Pp..........    0.000                               0.000    0.000
##    .D.H..NOT......    0.000                               0.000    0.000
##    .D.Ppl.r.frd.f.    0.000                               0.000    0.000
##     C.prestige        0.000                               0.000    0.000
##     C.dominance       0.000                               0.000    0.000
##
## Thresholds:
##                    Estimate  Std.Err  z-value  P(>|z|) ci.lower ci.upper
##     P.Ppl.rsp...|1   -1.710    0.133  -12.812    0.000   -1.972   -1.448
##     P.Ppl.rsp...|2   -1.251    0.102  -12.299    0.000   -1.450   -1.052
##     P.Ppl.rsp...|3   -0.816    0.086   -9.529    0.000   -0.984   -0.648
##     P.Ppl.rsp...|4    0.096    0.076    1.264    0.206   -0.053    0.245
##     P.Ppl.rsp...|5    1.313    0.105   12.513    0.000    1.108    1.519
##     P.P..NOT.....|   -1.894    0.153  -12.375    0.000   -2.194   -1.594
##     P.P..NOT.....|   -1.483    0.115  -12.863    0.000   -1.709   -1.257
##     P.P..NOT.....|   -0.829    0.086   -9.640    0.000   -0.997   -0.660
##     P.P..NOT.....|   -0.014    0.076   -0.181    0.857   -0.162    0.135
##     P.P..NOT.....|    0.881    0.087   10.079    0.000    0.710    1.053
##     P.Ppl.l.....|1   -1.381    0.109  -12.694    0.000   -1.594   -1.168
##     P.Ppl.l.....|2   -0.881    0.087  -10.079    0.000   -1.053   -0.710
##     P.Ppl.l.....|3   -0.235    0.076   -3.067    0.002   -0.384   -0.085
##     P.Ppl.l.....|4    0.301    0.077    3.907    0.000    0.150    0.452
##     P.Ppl.l.....|5    1.251    0.102   12.299    0.000    1.052    1.450
##     P.Pp..NOT...|1   -2.182    0.196  -11.128    0.000   -2.567   -1.798
##     P.Pp..NOT...|2   -1.430    0.112  -12.791    0.000   -1.649   -1.211
##     P.Pp..NOT...|3   -0.803    0.085   -9.417    0.000   -0.970   -0.636
##     P.Pp..NOT...|4   -0.197    0.076   -2.587    0.010   -0.347   -0.048
##     P.Pp..NOT...|5    0.616    0.081    7.584    0.000    0.456    0.775
##     P.H.s.h.....|1   -1.710    0.133  -12.812    0.000   -1.972   -1.448
##     P.H.s.h.....|2   -1.175    0.098  -11.973    0.000   -1.367   -0.983
##     P.H.s.h.....|3   -0.627    0.081   -7.701    0.000   -0.786   -0.467
##     P.H.s.h.....|4    0.188    0.076    2.467    0.014    0.039    0.337
##     P.H.s.h.....|5    1.335    0.106   12.578    0.000    1.127    1.543
##     P.H.........|1   -1.751    0.137  -12.744    0.000   -2.020   -1.481
##     P.H.........|2   -1.175    0.098  -11.973    0.000   -1.367   -0.983
##     P.H.........|3   -0.540    0.080   -6.763    0.000   -0.697   -0.384
##     P.H.........|4    0.301    0.077    3.907    0.000    0.150    0.452
```

```
##     P.H.........|5    1.335    0.106   12.578    0.000    1.127    1.543
##     P.H.........|1   -1.570    0.122  -12.911    0.000   -1.809   -1.332
##     P.H.........|2   -1.139    0.097  -11.796    0.000   -1.329   -0.950
##     P.H.........|3   -0.594    0.081   -7.350    0.000   -0.752   -0.435
##     P.H.........|4    0.225    0.076    2.947    0.003    0.075    0.375
##     P.H.........|5    1.292    0.104   12.445    0.000    1.089    1.495
##     P.Pp........|1   -1.570    0.122  -12.911    0.000   -1.809   -1.332
##     P.Pp........|2   -1.175    0.098  -11.973    0.000   -1.367   -0.983
##     P.Pp........|3   -0.649    0.082   -7.933    0.000   -0.809   -0.489
##     P.Pp........|4    0.207    0.076    2.707    0.007    0.057    0.356
##     P.Pp........|5    1.483    0.115   12.863    0.000    1.257    1.709
##     P.O...NOT.....   -2.294    0.218  -10.508    0.000   -2.721   -1.866
##     P.O...NOT.....   -2.018    0.169  -11.915    0.000   -2.349   -1.686
##     P.O...NOT.....   -1.122    0.096  -11.705    0.000   -1.310   -0.934
##     P.O...NOT.....   -0.207    0.076   -2.707    0.007   -0.356   -0.057
##     P.O...NOT.....    0.660    0.082    8.049    0.000    0.499    0.821
##     D.H.njy.....|1   -0.908    0.088  -10.295    0.000   -1.081   -0.736
##     D.H.njy.....|2   -0.282    0.077   -3.667    0.000   -0.432   -0.131
##     D.H.njy.....|3    0.339    0.077    4.386    0.000    0.188    0.491
##     D.H.njy.....|4    1.251    0.102   12.299    0.000    1.052    1.450
##     D.H.njy.....|5    2.093    0.181   11.577    0.000    1.739    2.447
##     D.H...........   -0.908    0.088  -10.295    0.000   -1.081   -0.736
##     D.H...........   -0.142    0.076   -1.865    0.062   -0.291    0.007
##     D.H...........    0.488    0.079    6.172    0.000    0.333    0.643
##     D.H...........    1.381    0.109   12.694    0.000    1.168    1.594
##     D.H...........    2.294    0.218   10.508    0.000    1.866    2.721
##     D.H..........|   -0.791    0.085   -9.305    0.000   -0.957   -0.624
##     D.H..........|   -0.050    0.076   -0.662    0.508   -0.199    0.098
##     D.H..........|    0.616    0.081    7.584    0.000    0.456    0.775
##     D.H..........|    1.381    0.109   12.694    0.000    1.168    1.594
##     D.H..........|    2.093    0.181   11.577    0.000    1.739    2.447
##     D.H...........   -0.895    0.088  -10.188    0.000   -1.067   -0.723
##     D.H...........   -0.207    0.076   -2.707    0.007   -0.356   -0.057
##     D.H...........    0.458    0.079    5.816    0.000    0.303    0.612
##     D.H...........    1.251    0.102   12.299    0.000    1.052    1.450
##     D.H...........    2.018    0.169   11.915    0.000    1.686    2.349
##     D.H..NOT......   -1.405    0.110  -12.745    0.000   -1.621   -1.189
##     D.H..NOT......   -0.498    0.079   -6.291    0.000   -0.654   -0.343
##     D.H..NOT......    0.087    0.076    1.144    0.253   -0.062    0.235
##     D.H..NOT......    0.754    0.084    8.967    0.000    0.589    0.919
##     D.H..NOT......    1.381    0.109   12.694    0.000    1.168    1.594
##     D.P..........|   -1.251    0.102  -12.299    0.000   -1.450   -1.052
##     D.P..........|   -0.329    0.077   -4.266    0.000   -0.481   -0.178
##     D.P..........|    0.388    0.078    4.983    0.000    0.235    0.540
##     D.P..........|    1.212    0.100   12.141    0.000    1.016    1.408
##     D.P..........|    2.093    0.181   11.577    0.000    1.739    2.447
##     D.H..NOT......   -1.842    0.147  -12.531    0.000   -2.130   -1.554
##     D.H..NOT......   -0.778    0.085   -9.193    0.000   -0.944   -0.612
```

```
##     D.H..NOT......   -0.023    0.076   -0.301    0.763   -0.171    0.126
##     D.H..NOT......    0.672    0.082    8.165    0.000    0.510    0.833
##     D.H..NOT......    1.313    0.105   12.513    0.000    1.108    1.519
##     D.Ppl.r.fr..|1   -0.388    0.078   -4.983    0.000   -0.540   -0.235
##     D.Ppl.r.fr..|2    0.368    0.078    4.744    0.000    0.216    0.520
##     D.Ppl.r.fr..|3    0.965    0.090   10.719    0.000    0.789    1.141
##     D.Ppl.r.fr..|4    1.795    0.142   12.652    0.000    1.517    2.073
##     D.Ppl.r.fr..|5    2.444    0.255    9.594    0.000    1.944    2.943
##
## Variances:
##                    Estimate  Std.Err  z-value  P(>|z|) ci.lower ci.upper
##    .P.Ppl.rspct...    0.127                               0.127    0.127
##    .P.Pp..NOT.....    0.573                               0.573    0.573
##    .P.Ppl.lwy.....    0.226                               0.226    0.226
##    .P.Ppl.d.NOT...    0.447                               0.447    0.447
##    .P.H.s.hld.....    0.128                               0.128    0.128
##    .P.Hs.n........    0.302                               0.302    0.302
##    .P.H.s.c.......    0.161                               0.161    0.161
##    .P.Ppl.s.......    0.191                               0.191    0.191
##    .P.O...NOT.....    0.995                               0.995    0.995
##    .D.H.njys.h....    0.220                               0.220    0.220
##    .D.H...........    0.241                               0.241    0.241
##    .D.H.s.........    0.316                               0.316    0.316
##    .D.H...........    0.192                               0.192    0.192
##    .D.H..NOT......    0.540                               0.540    0.540
##    .D.Pp..........    0.292                               0.292    0.292
##    .D.H..NOT......    0.579                               0.579    0.579
##    .D.Ppl.r.frd.f.    0.447                               0.447    0.447
##     C.prestige        0.873    0.019   46.696    0.000    0.836    0.909
##     C.dominance       0.780    0.032   24.654    0.000    0.718    0.842
##
## Scales y*:
##                    Estimate  Std.Err  z-value  P(>|z|) ci.lower ci.upper
##     P.Ppl.rspct...    1.000                               1.000    1.000
##     P.Pp..NOT.....    1.000                               1.000    1.000
##     P.Ppl.lwy.....    1.000                               1.000    1.000
##     P.Ppl.d.NOT...    1.000                               1.000    1.000
##     P.H.s.hld.....    1.000                               1.000    1.000
##     P.Hs.n........    1.000                               1.000    1.000
##     P.H.s.c.......    1.000                               1.000    1.000
##     P.Ppl.s.......    1.000                               1.000    1.000
##     P.O...NOT.....    1.000                               1.000    1.000
##     D.H.njys.h....    1.000                               1.000    1.000
##     D.H...........    1.000                               1.000    1.000
##     D.H.s.........    1.000                               1.000    1.000
##     D.H...........    1.000                               1.000    1.000
##     D.H..NOT......    1.000                               1.000    1.000
##     D.Pp..........    1.000                               1.000    1.000
```

```
##     D.H..NOT......    1.000                                  1.000    1.000
##     D.Ppl.r.frd.f.    1.000                                  1.000    1.000
```

```
#   Calculate predicted factor scores for comparative data
data.v.r = cbind(data.v.o, lavPredict(cfa.complex, newdata=data.v.o, method="EBM"))
data.v.r = cbind(data.v.r, lavPredict(soldyn.complex, newdata=data.v.o,
                                      method="EBM"))
data.v.r = cbind(data.v.r, lavPredict(cheng.fit, newdata=data.v.o, method="EBM"))

#   Polychoric correlations between 'prestigious' item, PRI factor scores,
#       solidarity and dynamism factor scores, and Cheng et al. (2010)
#       prestige and dominance factor scores
scale.means.poly = lavCor(data.v.r[,c(4,38:41,42:43,44:45)],
                          ordered=colnames(data.v.r)[4])
scale.means.poly
```

```
##             prstgs prestg positn repttn infrmt sldrty dynmsm C.prst C.dmnn
## prestigious  1.000
## prestige     0.905  1.000
## position     0.921  0.967  1.000
## reputation   0.877  0.968  0.908  1.000
## information  0.857  0.984  0.922  0.937  1.000
## solidarity  -0.012  0.117  0.014  0.213  0.128  1.000
## dynamism     0.127  0.247  0.149  0.334  0.253  0.823  1.000
## C.prestige   0.805  0.850  0.805  0.861  0.828  0.213  0.340  1.000
## C.dominance  0.556  0.520  0.565  0.468  0.488 -0.341 -0.104  0.392  1.000
```

```
#   Fit model for Cheng et al. (2010) with reversed items removed
#       Define model
cheng.model2 = "
prestige             =~ P.People.respect.and.admire.him +
    P.People.always.expect.him.to.be.successful +
    P.He.is.held.in.high.esteem.by.people +
    P.His.unique.talents.and.abilities.are.recognized.by.other.people +
    P.He.is.considered.an.expert.on.some.matters.by.people +
    P.People.seek.his.advice.on.a.variety.of.matters

dominance            =~ D.He.enjoys.having.control.over.other.people +
    D.He.often.tries.to.get.his.own.way.regardless.of.what.other.people.may.want +
    D.He.is.willing.to.use.aggressive.tactics.to.get.his.way +
    D.He.tries.to.control.others.rather.than.permit.them.to.control.him +
    D.People.know.it.is.better.to.let.him.have.his.way +
    D.People.are.afraid.of.him
"

#       Fit CFA model
cheng.fit2 = cfa(cheng.model2, data.v.o, estimator="WLSMV")
summary(cheng.fit2, fit.measures=T, ci=T)
```

```
## lavaan (0.5-23.1097) converged normally after  33 iterations
```

```
##
##   Number of observations                           275
##
##   Estimator                                       DWLS      Robust
##   Minimum Function Test Statistic              248.503     382.620
##   Degrees of freedom                                53          53
##   P-value (Chi-square)                           0.000       0.000
##   Scaling correction factor                                  0.691
##   Shift parameter                                           22.766
##     for simple second-order correction (Mplus variant)
##
## Model test baseline model:
##
##   Minimum Function Test Statistic            37225.877   12273.214
##   Degrees of freedom                                66          66
##   P-value                                        0.000       0.000
##
## User model versus baseline model:
##
##   Comparative Fit Index (CFI)                    0.995       0.973
##   Tucker-Lewis Index (TLI)                       0.993       0.966
##
##   Robust Comparative Fit Index (CFI)                            NA
##   Robust Tucker-Lewis Index (TLI)                               NA
##
## Root Mean Square Error of Approximation:
##
##   RMSEA                                          0.116       0.151
##   90 Percent Confidence Interval          0.102  0.131       0.137  0.165
##   P-value RMSEA <= 0.05                          0.000       0.000
##
##   Robust RMSEA                                                  NA
##   90 Percent Confidence Interval                                NA     NA
##
## Standardized Root Mean Square Residual:
##
##   SRMR                                           0.083       0.083
##
## Weighted Root Mean Square Residual:
##
##   WRMR                                           1.404       1.404
##
## Parameter Estimates:
##
##   Information                                 Expected
##   Standard Errors                           Robust.sem
##
## Latent Variables:
```

```
##                    Estimate  Std.Err  z-value  P(>|z|) ci.lower ci.upper
##   prestige =~
##     P.Ppl.rspct...    1.000                               1.000    1.000
##     P.Ppl.lwy.....    0.941    0.016   57.577    0.000    0.909    0.973
##     P.H.s.hld.....    1.004    0.014   72.926    0.000    0.977    1.031
##     P.Hs.n........    0.894    0.020   45.249    0.000    0.856    0.933
##     P.H.s.c.......    0.987    0.014   69.976    0.000    0.959    1.015
##     P.Ppl.s.......    0.964    0.015   64.470    0.000    0.935    0.993
##   dominance =~
##     D.H.njys.h....    1.000                               1.000    1.000
##     D.H...........    1.006    0.024   42.523    0.000    0.960    1.052
##     D.H.s.........    0.950    0.027   35.787    0.000    0.898    1.002
##     D.H...........    1.018    0.024   42.485    0.000    0.971    1.065
##     D.Pp..........    0.980    0.026   37.360    0.000    0.929    1.032
##     D.Ppl.r.frd.f.    0.854    0.031   27.849    0.000    0.794    0.914
##
## Covariances:
##                    Estimate  Std.Err  z-value  P(>|z|) ci.lower ci.upper
##   prestige ~~
##     dominance         0.347    0.038    9.139    0.000    0.273    0.422
##
## Intercepts:
##                    Estimate  Std.Err  z-value  P(>|z|) ci.lower ci.upper
##    .P.Ppl.rspct...    0.000                               0.000    0.000
##    .P.Ppl.lwy.....    0.000                               0.000    0.000
##    .P.H.s.hld.....    0.000                               0.000    0.000
##    .P.Hs.n........    0.000                               0.000    0.000
##    .P.H.s.c.......    0.000                               0.000    0.000
##    .P.Ppl.s.......    0.000                               0.000    0.000
##    .D.H.njys.h....    0.000                               0.000    0.000
##    .D.H...........    0.000                               0.000    0.000
##    .D.H.s.........    0.000                               0.000    0.000
##    .D.H...........    0.000                               0.000    0.000
##    .D.Pp..........    0.000                               0.000    0.000
##    .D.Ppl.r.frd.f.    0.000                               0.000    0.000
##     prestige          0.000                               0.000    0.000
##     dominance         0.000                               0.000    0.000
##
## Thresholds:
##                    Estimate  Std.Err  z-value  P(>|z|) ci.lower ci.upper
##     P.Ppl.rsp...|1   -1.710    0.133  -12.812    0.000   -1.972   -1.448
##     P.Ppl.rsp...|2   -1.251    0.102  -12.299    0.000   -1.450   -1.052
##     P.Ppl.rsp...|3   -0.816    0.086   -9.529    0.000   -0.984   -0.648
##     P.Ppl.rsp...|4    0.096    0.076    1.264    0.206   -0.053    0.245
##     P.Ppl.rsp...|5    1.313    0.105   12.513    0.000    1.108    1.519
##     P.Ppl.l.....|1   -1.381    0.109  -12.694    0.000   -1.594   -1.168
##     P.Ppl.l.....|2   -0.881    0.087  -10.079    0.000   -1.053   -0.710
##     P.Ppl.l.....|3   -0.235    0.076   -3.067    0.002   -0.384   -0.085
```

```
##      P.Ppl.l.....|4    0.301    0.077    3.907    0.000    0.150    0.452
##      P.Ppl.l.....|5    1.251    0.102   12.299    0.000    1.052    1.450
##      P.H.s.h.....|1   -1.710    0.133  -12.812    0.000   -1.972   -1.448
##      P.H.s.h.....|2   -1.175    0.098  -11.973    0.000   -1.367   -0.983
##      P.H.s.h.....|3   -0.627    0.081   -7.701    0.000   -0.786   -0.467
##      P.H.s.h.....|4    0.188    0.076    2.467    0.014    0.039    0.337
##      P.H.s.h.....|5    1.335    0.106   12.578    0.000    1.127    1.543
##      P.H.........|1   -1.751    0.137  -12.744    0.000   -2.020   -1.481
##      P.H.........|2   -1.175    0.098  -11.973    0.000   -1.367   -0.983
##      P.H.........|3   -0.540    0.080   -6.763    0.000   -0.697   -0.384
##      P.H.........|4    0.301    0.077    3.907    0.000    0.150    0.452
##      P.H.........|5    1.335    0.106   12.578    0.000    1.127    1.543
##      P.H.........|1   -1.570    0.122  -12.911    0.000   -1.809   -1.332
##      P.H.........|2   -1.139    0.097  -11.796    0.000   -1.329   -0.950
##      P.H.........|3   -0.594    0.081   -7.350    0.000   -0.752   -0.435
##      P.H.........|4    0.225    0.076    2.947    0.003    0.075    0.375
##      P.H.........|5    1.292    0.104   12.445    0.000    1.089    1.495
##      P.Pp........|1   -1.570    0.122  -12.911    0.000   -1.809   -1.332
##      P.Pp........|2   -1.175    0.098  -11.973    0.000   -1.367   -0.983
##      P.Pp........|3   -0.649    0.082   -7.933    0.000   -0.809   -0.489
##      P.Pp........|4    0.207    0.076    2.707    0.007    0.057    0.356
##      P.Pp........|5    1.483    0.115   12.863    0.000    1.257    1.709
##      D.H.njy.....|1   -0.908    0.088  -10.295    0.000   -1.081   -0.736
##      D.H.njy.....|2   -0.282    0.077   -3.667    0.000   -0.432   -0.131
##      D.H.njy.....|3    0.339    0.077    4.386    0.000    0.188    0.491
##      D.H.njy.....|4    1.251    0.102   12.299    0.000    1.052    1.450
##      D.H.njy.....|5    2.093    0.181   11.577    0.000    1.739    2.447
##      D.H..........    -0.908    0.088  -10.295    0.000   -1.081   -0.736
##      D.H..........    -0.142    0.076   -1.865    0.062   -0.291    0.007
##      D.H..........     0.488    0.079    6.172    0.000    0.333    0.643
##      D.H..........     1.381    0.109   12.694    0.000    1.168    1.594
##      D.H..........     2.294    0.218   10.508    0.000    1.866    2.721
##      D.H.........|    -0.791    0.085   -9.305    0.000   -0.957   -0.624
##      D.H.........|    -0.050    0.076   -0.662    0.508   -0.199    0.098
##      D.H.........|     0.616    0.081    7.584    0.000    0.456    0.775
##      D.H.........|     1.381    0.109   12.694    0.000    1.168    1.594
##      D.H.........|     2.093    0.181   11.577    0.000    1.739    2.447
##      D.H..........    -0.895    0.088  -10.188    0.000   -1.067   -0.723
##      D.H..........    -0.207    0.076   -2.707    0.007   -0.356   -0.057
##      D.H..........     0.458    0.079    5.816    0.000    0.303    0.612
##      D.H..........     1.251    0.102   12.299    0.000    1.052    1.450
##      D.H..........     2.018    0.169   11.915    0.000    1.686    2.349
##      D.P.........|    -1.251    0.102  -12.299    0.000   -1.450   -1.052
##      D.P.........|    -0.329    0.077   -4.266    0.000   -0.481   -0.178
##      D.P.........|     0.388    0.078    4.983    0.000    0.235    0.540
##      D.P.........|     1.212    0.100   12.141    0.000    1.016    1.408
##      D.P.........|     2.093    0.181   11.577    0.000    1.739    2.447
##      D.Ppl.r.fr..|1   -0.388    0.078   -4.983    0.000   -0.540   -0.235
```

```
##     D.Ppl.r.fr..|2    0.368    0.078    4.744    0.000    0.216    0.520
##     D.Ppl.r.fr..|3    0.965    0.090   10.719    0.000    0.789    1.141
##     D.Ppl.r.fr..|4    1.795    0.142   12.652    0.000    1.517    2.073
##     D.Ppl.r.fr..|5    2.444    0.255    9.594    0.000    1.944    2.943
##
## Variances:
##                    Estimate  Std.Err  z-value  P(>|z|) ci.lower ci.upper
##    .P.Ppl.rspct...    0.130                               0.130    0.130
##    .P.Ppl.lwy.....    0.229                               0.229    0.229
##    .P.H.s.hld.....    0.124                               0.124    0.124
##    .P.Hs.n........    0.304                               0.304    0.304
##    .P.H.s.c.......    0.152                               0.152    0.152
##    .P.Ppl.s.......    0.191                               0.191    0.191
##    .D.H.njys.h....    0.227                               0.227    0.227
##    .D.H...........    0.218                               0.218    0.218
##    .D.H.s.........    0.302                               0.302    0.302
##    .D.H...........    0.198                               0.198    0.198
##    .D.Pp..........    0.257                               0.257    0.257
##    .D.Ppl.r.frd.f.    0.436                               0.436    0.436
##     prestige          0.870    0.019   46.577    0.000    0.834    0.907
##     dominance         0.773    0.031   24.702    0.000    0.712    0.834
##
## Scales y*:
##                    Estimate  Std.Err  z-value  P(>|z|) ci.lower ci.upper
##     P.Ppl.rspct...    1.000                               1.000    1.000
##     P.Ppl.lwy.....    1.000                               1.000    1.000
##     P.H.s.hld.....    1.000                               1.000    1.000
##     P.Hs.n........    1.000                               1.000    1.000
##     P.H.s.c.......    1.000                               1.000    1.000
##     P.Ppl.s.......    1.000                               1.000    1.000
##     D.H.njys.h....    1.000                               1.000    1.000
##     D.H...........    1.000                               1.000    1.000
##     D.H.s.........    1.000                               1.000    1.000
##     D.H...........    1.000                               1.000    1.000
##     D.Pp..........    1.000                               1.000    1.000
##     D.Ppl.r.frd.f.    1.000                               1.000    1.000
```

```
#   Calculate new predicted factor scores for Cheng et al. (2010) prestige
#       and dominance domains
data.v.r2 = data.v.r
data.v.r2[,c(44:45)] = lavPredict(cheng.fit2, newdata=data.v.o, method="EBM")

#   Polychoric correlations between 'prestigious' item, PRI factor scores,
#       solidarity and dynamism factor scores, and Cheng et al. (2010)
#       prestige and dominance factor scores without reversed items
scale.means.poly2 = lavCor(data.v.r2[,c(4,38:41,42:43,44:45)],
                           ordered=colnames(data.v.r2)[4])
scale.means.poly2
```

```
##             prstgs prestg positn repttn infrmt sldrty dynmsm C.prst C.dmnn
## prestigious  1.000
## prestige     0.905  1.000
## position     0.921  0.967  1.000
## reputation   0.877  0.968  0.908  1.000
## information  0.857  0.984  0.922  0.937  1.000
## solidarity  -0.012  0.117  0.014  0.213  0.128  1.000
## dynamism     0.127  0.247  0.149  0.334  0.253  0.823  1.000
## C.prestige   0.812  0.856  0.810  0.867  0.832  0.231  0.356  1.000
## C.dominance  0.561  0.533  0.569  0.489  0.501 -0.257 -0.039  0.449  1.000
```

```
#   Fit PRI model to comparative data
pri.fit = cfa(cfa.model1, data.v.o, estimator="WLSMV")
summary(pri.fit, fit.measures=T, ci=T)
```

```
## lavaan (0.5-23.1097) converged normally after  29 iterations
##
##   Number of observations                           275
##
##   Estimator                                       DWLS      Robust
##   Minimum Function Test Statistic               15.087      44.899
##   Degrees of freedom                                11          11
##   P-value (Chi-square)                           0.179       0.000
##   Scaling correction factor                                  0.354
##   Shift parameter                                            2.294
##     for simple second-order correction (Mplus variant)
##
## Model test baseline model:
##
##   Minimum Function Test Statistic            29411.314   13757.988
##   Degrees of freedom                                21          21
##   P-value                                        0.000       0.000
##
## User model versus baseline model:
##
##   Comparative Fit Index (CFI)                    1.000       0.998
##   Tucker-Lewis Index (TLI)                       1.000       0.995
##
##   Robust Comparative Fit Index (CFI)                            NA
##   Robust Tucker-Lewis Index (TLI)                               NA
##
## Root Mean Square Error of Approximation:
##
##   RMSEA                                          0.037       0.106
##   90 Percent Confidence Interval          0.000  0.078       0.075  0.139
##   P-value RMSEA <= 0.05                          0.649       0.002
##
##   Robust RMSEA                                                  NA
##   90 Percent Confidence Interval                                NA      NA
```

```
##
## Standardized Root Mean Square Residual:
##
##   SRMR                                           0.019       0.019
##
## Weighted Root Mean Square Residual:
##
##   WRMR                                           0.519       0.519
##
## Parameter Estimates:
##
##   Information                                 Expected
##   Standard Errors                           Robust.sem
##
## Latent Variables:
##                    Estimate  Std.Err  z-value  P(>|z|) ci.lower ci.upper
##   prestige =~
##     position          1.000                               1.000    1.000
##     reputation        1.003    0.030   33.026    0.000    0.944    1.063
##     information       1.153    0.030   38.520    0.000    1.094    1.211
##   position =~
##     wealthy           1.000                               1.000    1.000
##     powerful          0.899    0.025   35.811    0.000    0.849    0.948
##     high.socl.stts    1.055    0.021   50.525    0.000    1.014    1.096
##   reputation =~
##     reputable         1.000                               1.000    1.000
##     respected         1.066    0.024   44.290    0.000    1.019    1.113
##   information =~
##     educated          1.000                               1.000    1.000
##     intelligent       0.990    0.013   75.056    0.000    0.964    1.015
##
## Intercepts:
##                    Estimate  Std.Err  z-value  P(>|z|) ci.lower ci.upper
##    .wealthy           0.000                               0.000    0.000
##    .powerful          0.000                               0.000    0.000
##    .high.socl.stts    0.000                               0.000    0.000
##    .reputable         0.000                               0.000    0.000
##    .respected         0.000                               0.000    0.000
##    .educated          0.000                               0.000    0.000
##    .intelligent       0.000                               0.000    0.000
##     prestige          0.000                               0.000    0.000
##     position          0.000                               0.000    0.000
##     reputation        0.000                               0.000    0.000
##     information       0.000                               0.000    0.000
##
## Thresholds:
##                    Estimate  Std.Err  z-value  P(>|z|) ci.lower ci.upper
##     wealthy|t1       -1.251    0.102  -12.299    0.000   -1.450   -1.052
```

```
##     wealthy|t2       -0.627    0.081   -7.701    0.000   -0.786   -0.467
##     wealthy|t3       -0.059    0.076   -0.782    0.434   -0.208    0.089
##     wealthy|t4        0.616    0.081    7.584    0.000    0.456    0.775
##     wealthy|t5        1.602    0.124   12.907    0.000    1.359    1.846
##     powerful|t1      -1.570    0.122  -12.911    0.000   -1.809   -1.332
##     powerful|t2      -0.803    0.085   -9.417    0.000   -0.970   -0.636
##     powerful|t3      -0.123    0.076   -1.625    0.104   -0.272    0.025
##     powerful|t4       0.572    0.080    7.116    0.000    0.415    0.730
##     powerful|t5       1.602    0.124   12.907    0.000    1.359    1.846
##     hgh.scl.stts|1   -1.335    0.106  -12.578    0.000   -1.543   -1.127
##     hgh.scl.stts|2   -0.695    0.083   -8.395    0.000   -0.857   -0.532
##     hgh.scl.stts|3   -0.123    0.076   -1.625    0.104   -0.272    0.025
##     hgh.scl.stts|4    0.339    0.077    4.386    0.000    0.188    0.491
##     hgh.scl.stts|5    1.251    0.102   12.299    0.000    1.052    1.450
##     reputable|t1     -1.636    0.127  -12.891    0.000   -1.885   -1.387
##     reputable|t2     -1.251    0.102  -12.299    0.000   -1.450   -1.052
##     reputable|t3     -0.829    0.086   -9.640    0.000   -0.997   -0.660
##     reputable|t4      0.041    0.076    0.542    0.588   -0.107    0.189
##     reputable|t5      1.157    0.097   11.886    0.000    0.966    1.348
##     respected|t1     -1.710    0.133  -12.812    0.000   -1.972   -1.448
##     respected|t2     -1.335    0.106  -12.578    0.000   -1.543   -1.127
##     respected|t3     -0.754    0.084   -8.967    0.000   -0.919   -0.589
##     respected|t4     -0.041    0.076   -0.542    0.588   -0.189    0.107
##     respected|t5      0.994    0.091   10.925    0.000    0.816    1.173
##     educated|t1      -1.483    0.115  -12.863    0.000   -1.709   -1.257
##     educated|t2      -1.056    0.093  -11.326    0.000   -1.239   -0.873
##     educated|t3      -0.660    0.082   -8.049    0.000   -0.821   -0.499
##     educated|t4      -0.087    0.076   -1.144    0.253   -0.235    0.062
##     educated|t5       0.683    0.082    8.280    0.000    0.521    0.845
##     intelligent|t1   -1.751    0.137  -12.744    0.000   -2.020   -1.481
##     intelligent|t2   -1.335    0.106  -12.578    0.000   -1.543   -1.127
##     intelligent|t3   -0.895    0.088  -10.188    0.000   -1.067   -0.723
##     intelligent|t4   -0.068    0.076   -0.903    0.367   -0.217    0.080
##     intelligent|t5    0.868    0.087    9.970    0.000    0.697    1.039
##
## Variances:
##                    Estimate  Std.Err  z-value  P(>|z|) ci.lower ci.upper
##    .wealthy           0.166                               0.166    0.166
##    .powerful          0.326                               0.326    0.326
##    .high.socl.stts    0.071                               0.071    0.071
##    .reputable         0.219                               0.219    0.219
##    .respected         0.113                               0.113    0.113
##    .educated          0.081                               0.081    0.081
##    .intelligent       0.100                               0.100    0.100
##     prestige          0.698    0.032   21.727    0.000    0.635    0.761
##     position          0.136    0.020    6.861    0.000    0.097    0.175
##     reputation        0.078    0.020    3.991    0.000    0.040    0.117
##     information      -0.008    0.020   -0.429    0.668   -0.047    0.030
```

```
##
## Scales y*:
##                    Estimate  Std.Err  z-value  P(>|z|) ci.lower ci.upper
##     wealthy           1.000                               1.000    1.000
##     powerful          1.000                               1.000    1.000
##     high.socl.stts    1.000                               1.000    1.000
##     reputable         1.000                               1.000    1.000
##     respected         1.000                               1.000    1.000
##     educated          1.000                               1.000    1.000
##     intelligent       1.000                               1.000    1.000
```

Interrater Reliability

```
#   Reshape scale construction study PRI attitudinal item data to wide format
data.s.ir = data.s.l[,c("id","accent","item","score")]
data.s.ir = data.s.ir[data.s.ir$item %in%
                         c("wealthy","powerful","high.social.status",
                           "reputable","respected",
                           "educated","intelligent"),]
data.s.ir = as.data.frame(complete(data.s.ir, id, nesting(accent, item)))
data.s.ir = data.s.ir[order(data.s.ir$id, data.s.ir$accent, data.s.ir$item),]
data.s.ir = reshape(data.s.ir, idvar=c("id","accent"),
                    timevar="item", direction="wide")
data.s.ir = reshape(data.s.ir, idvar="id", timevar="accent", direction="wide")
data.s.ir = data.s.ir[!as.character(data.s.ir$id) %in% data.s.to.remove,]

#   Calculate interrater reliability indices of scale items in
#       scale construction data
kripp.alpha(as.matrix(data.s.ir[,-1]), "ordinal")
```

```
##  Krippendorff's alpha
##
##  Subjects = 28
##    Raters = 302
##     alpha = 0.414
```

```
icc(t(as.matrix(data.s.ir[,-1])), "twoway", "consistency", "single")
```

```
##  Single Score Intraclass Correlation
##
##    Model: twoway
##    Type : consistency
##
##    Subjects = 28
##      Raters = 302
##    ICC(C,1) = 0.473
##
##  F-Test, H0: r0 = 0 ; H1: r0 > 0
```

```
## F(27,8127) = 272 , p = 0
##
## 95%-Confidence Interval for ICC Population Values:
##  0.359 < ICC < 0.625
```

```
#   Reshape scale evaluation study PRI attitudinal item data to wide format
data.c.ir = data.c.l[,c("id","accent","item","score")]
data.c.ir = data.c.ir[data.c.ir$item %in%
                         c("wealthy","powerful","high.social.status",
                           "reputable","respected",
                           "educated","intelligent"),]
data.c.ir = as.data.frame(complete(data.c.ir, id, nesting(accent, item)))
data.c.ir = data.c.ir[order(data.c.ir$id, data.c.ir$accent, data.c.ir$item),]
data.c.ir = reshape(data.c.ir, idvar=c("id","accent"),
                    timevar="item", direction="wide")
data.c.ir = reshape(data.c.ir, idvar="id", timevar="accent", direction="wide")
data.c.ir = data.c.ir[!as.character(data.c.ir$id) %in% data.c.to.remove,]

#   Calculate interrater reliability indices of scale items in
#       scale evaluation data
kripp.alpha(as.matrix(data.c.ir[,-1]), "ordinal")
```

```
## Krippendorff's alpha
##
## Subjects = 112
##   Raters = 291
##    alpha = 0.383
```

```
icc(t(as.matrix(data.c.ir[,-1])), "twoway", "consistency", "single")
```

```
## Single Score Intraclass Correlation
##
##   Model: twoway
##   Type : consistency
##
##   Subjects = 28
##     Raters = 291
##   ICC(C,1) = 0.459
##
## F-Test, H0: r0 = 0 ; H1: r0 > 0
## F(27,7830) = 248 , p = 0
##
## 95%-Confidence Interval for ICC Population Values:
##  0.346 < ICC < 0.612
```

```
#   Reshape comparative study PRI attitudinal item data to wide format
data.v.ir = data.v.l[,c("id","accent","item","score")]
data.v.ir = data.v.ir[data.v.ir$item %in%
                         c("wealthy","powerful","high.social.status",
                           "reputable","respected",
```

```
                           "educated","intelligent"),]
data.v.ir = as.data.frame(complete(data.v.ir, id, nesting(accent, item)))
data.v.ir = data.v.ir[order(data.v.ir$id, data.v.ir$accent, data.v.ir$item),]
data.v.ir = reshape(data.v.ir, idvar=c("id","accent"),
                    timevar="item", direction="wide")
data.v.ir = reshape(data.v.ir, idvar="id", timevar="accent", direction="wide")
data.v.ir = data.v.ir[!as.character(data.v.ir$id) %in% data.v.to.remove,]

#   Calculate interrater reliability indices of scale items in
#       comparative data
kripp.alpha(as.matrix(data.v.ir[,-1]), "ordinal")

##  Krippendorff's alpha
##
##  Subjects = 14
##    Raters = 138
##     alpha = 0.438

icc(t(as.matrix(data.v.ir[,-1])), "twoway", "consistency", "single")

##  Single Score Intraclass Correlation
##
##    Model: twoway
##    Type : consistency
##
##    Subjects = 7
##      Raters = 138
##    ICC(C,1) = 0.208
##
##  F-Test, H0: r0 = 0 ; H1: r0 > 0
##    F(6,822) = 37.2 , p = 5.71e-40
##
##  95%-Confidence Interval for ICC Population Values:
##   0.094 < ICC < 0.565

#   Reshape comparative study Cheng et al. (2010) attitudinal item data
#       to wide format
data.v.ir2 = data.v.l[,c("id","accent","item","score")]
data.v.ir2 = data.v.ir2[data.v.ir2$item %in%
                         colnames(data.v)[21:37],]
data.v.ir2 = as.data.frame(complete(data.v.ir2, id, nesting(accent, item)))
data.v.ir2 = data.v.ir2[order(data.v.ir2$id, data.v.ir2$accent, data.v.ir2$item),]
data.v.ir2 = reshape(data.v.ir2, idvar=c("id","accent"),
                    timevar="item", direction="wide")
data.v.ir2 = reshape(data.v.ir2, idvar="id", timevar="accent", direction="wide")
data.v.ir2 = data.v.ir2[!as.character(data.v.ir2$id) %in% data.v.to.remove,]

#   Calculate interrater reliability indices of Cheng et al. (2010) items in
#       comparative data
kripp.alpha(as.matrix(data.v.ir2[,-1]), "ordinal")
```

```
##  Krippendorff's alpha
##
##  Subjects = 34
##    Raters = 138
##     alpha = 0.405
```

```
icc(t(as.matrix(data.v.ir2[,-1])), "twoway", "consistency", "single")
```

```
##  Single Score Intraclass Correlation
##
##    Model: twoway
##    Type : consistency
##
##    Subjects = 17
##      Raters = 138
##    ICC(C,1) = 0.338
##
##  F-Test, H0: r0 = 0 ; H1: r0 > 0
##  F(16,2192) = 71.6 , p = 2.09e-186
##
##  95%-Confidence Interval for ICC Population Values:
##   0.218 < ICC < 0.545
```

Internal Consistency

```
#   Calculate internal consistency of PRI scale and individual prestige construct
#       Cronbach's alpha and itemwise reliability statistics
psych::alpha(fitted(cfa.complex)$cov, n.obs=nrow(data.c))$total
```

```
##  raw_alpha std.alpha   G6(smc) average_r      S/N         ase
##  0.8928735 0.8923341 0.8931629 0.5421244 8.287995 0.002999166
```

```
psych::alpha(fitted(cfa.complex)$cov, n.obs=nrow(data.c))
```

```
##
## Reliability analysis
## Call: psych::alpha(x = fitted(cfa.complex)$cov, n.obs = nrow(data.c))
##
##   raw_alpha std.alpha G6(smc) average_r S/N   ase
##       0.89      0.89    0.89      0.54 8.3 0.003
##
##  lower alpha upper     95% confidence boundaries
## 0.89 0.89 0.9
##
##  Reliability if an item is dropped:
##                    raw_alpha std.alpha G6(smc) average_r S/N alpha se
## wealthy                 0.87      0.87    0.86      0.52 6.5   0.0038
## powerful                0.89      0.89    0.89      0.57 8.1   0.0031
## high.social.status      0.87      0.87    0.86      0.52 6.4   0.0039
```

```
## reputable                0.88      0.88    0.88      0.56 7.6   0.0033
## respected                0.88      0.88    0.87      0.54 7.1   0.0034
## educated                 0.86      0.86    0.86      0.51 6.3   0.0039
## intelligent              0.89      0.89    0.88      0.57 7.9   0.0032
##
##  Item statistics
##                      r r.cor r.drop
## wealthy           0.84  0.82   0.77
## powerful          0.69  0.61   0.58
## high.social.status 0.85  0.84   0.78
## reputable         0.73  0.67   0.62
## respected         0.78  0.73   0.68
## educated          0.86  0.85   0.81
## intelligent       0.71  0.64   0.59
```

```
#       Raykov omega
reliabilityL2(cfa.complex, "prestige")
```

```
##       omegaL1       omegaL2 partialOmegaL1
##     0.8414905     0.9180585      0.9160317
```

```
#   Calculate internal consistency of PRI subscales
reliability(cfa.complex)[1:4,]
```

```
##         position reputation information
## alpha  0.8439488  0.7724712   0.7936687
## omega  0.8579791  0.7730284   0.8183194
## omega2 0.8579791  0.7730284   0.8183194
## omega3 0.8591647  0.7730281   0.8183190
```

R Session Information

```
## R version 3.4.0 (2017-04-21)
## Platform: x86_64-w64-mingw32/x64 (64-bit)
## Running under: Windows 10 x64 (build 10240)
##
## Matrix products: default
##
## locale:
## [1] LC_COLLATE=English_United States.1252
## [2] LC_CTYPE=English_United States.1252
## [3] LC_MONETARY=English_United States.1252
## [4] LC_NUMERIC=C
## [5] LC_TIME=English_United States.1252
##
## attached base packages:
## [1] grid      stats     graphics  grDevices utils     datasets  methods
## [8] base
##
## other attached packages:
##  [1] GPArotation_2014.11-1 irr_0.84              lpSolve_5.6.13
##  [4] fpc_2.1-10            cluster_2.0.6         scatterplot3d_0.3-40
##  [7] corrplot_0.84         ggplot2_2.2.1         rcompanion_1.5.6
## [10] coin_1.1-3            dplyr_0.5.0           tidyr_0.6.3
## [13] semPlot_1.1           lavaan.survey_1.1.3.1 survey_3.31-5
## [16] survival_2.41-3       Matrix_1.2-9          semTools_0.4-14
## [19] lavaan_0.5-23.1097    MVN_4.0.2             e1071_1.6-8
## [22] psych_1.7.5           Rmisc_1.5             plyr_1.8.4
## [25] lattice_0.20-35
##
## loaded via a namespace (and not attached):
##   [1] backports_1.1.0      Hmisc_4.0-3          miscTools_0.6-22
##   [4] BSDA_1.01            igraph_1.0.1         lazyeval_0.2.0
##   [7] sp_1.2-4             splines_3.4.0        TH.data_1.0-8
##  [10] digest_0.6.12        htmltools_0.3.6      matrixcalc_1.0-3
##  [13] memoise_1.1.0        magrittr_1.5         checkmate_1.8.2
##  [16] lisrelToR_0.1.4      sna_2.4              sandwich_2.3-4
##  [19] jpeg_0.1-8           sem_3.1-9            colorspace_1.3-2
##  [22] rrcov_1.4-3          jsonlite_1.5         lme4_1.1-13
##  [25] zoo_1.8-0            gtable_0.2.0         MatrixModels_0.4-1
##  [28] mi_1.0               car_2.1-4            kernlab_0.9-25
##  [31] maxLik_1.3-4         prabclus_2.2-6       DEoptimR_1.0-8
##  [34] ggm_2.3              abind_1.4-5          SparseM_1.77
##  [37] VIM_4.7.0            scales_0.4.1         sgeostat_1.0-27
##  [40] mvtnorm_1.0-6        DBI_0.6-1            GGally_1.3.0
##  [43] Rcpp_0.12.11         sROC_0.1-2           laeken_0.4.6
##  [46] htmlTable_1.9        foreign_0.8-67       mclust_5.3
```

```
##  [49] Formula_1.2-1         stats4_3.4.0          vcd_1.4-3
##  [52] httr_1.2.1            htmlwidgets_0.8       RColorBrewer_1.1-2
##  [55] acepack_1.4.1         modeltools_0.2-21     manipulate_1.0.1
##  [58] reshape_0.8.6         XML_3.98-1.7          flexmix_2.3-14
##  [61] multcompView_0.1-7    nnet_7.3-12           labeling_0.3
##  [64] rlang_0.2.0           reshape2_1.4.2        munsell_0.4.3
##  [67] tools_3.4.0           moments_0.14          ade4_1.7-6
##  [70] statnet.common_3.3.0  pls_2.6-0             devtools_1.13.2
##  [73] EMT_1.1               fdrtool_1.2.15        evaluate_0.10
##  [76] stringr_1.2.0         cvTools_0.3.2         arm_1.9-3
##  [79] yaml_2.1.14           knitr_1.16            robustbase_0.92-7
##  [82] RVAideMemoire_0.9-65  glasso_1.8            WRS2_0.9-2
##  [85] nlme_3.1-131          whisker_0.3-2         quantreg_5.33
##  [88] compiler_3.4.0        pbkrtest_0.4-7        curl_2.6
##  [91] png_0.1-7             huge_1.2.7            tibble_1.3.3
##  [94] robCompositions_2.0.3 hermite_1.1.1         DescTools_0.99.20
##  [97] pbivnorm_0.6.0        pcaPP_1.9-61          stringi_1.1.5
## [100] qgraph_1.4.3          rockchalk_1.8.101     trimcluster_0.1-2
## [103] nloptr_1.0.4          permute_0.9-4         vegan_2.4-3
## [106] mc2d_0.1-18           lmtest_0.9-35         ucminf_1.1-4
## [109] OpenMx_2.7.11         data.table_1.10.4     corpcor_1.6.9
## [112] ordinal_2015.6-28     R6_2.2.1              latticeExtra_0.6-28
## [115] network_1.13.0        gridExtra_2.2.1       codetools_0.2-15
## [118] boot_1.3-19           MASS_7.3-47           gtools_3.5.0
## [121] assertthat_0.2.0      rprojroot_1.2         rjson_0.2.15
## [124] withr_1.0.2           nortest_1.0-4         mnormt_1.5-5
## [127] multcomp_1.4-6        expm_0.999-2          diptest_0.75-7
## [130] mgcv_1.8-17           parallel_3.4.0        quadprog_1.5-5
## [133] rpart_4.1-11          coda_0.19-1           class_7.3-14
## [136] minqa_1.2.4           rmarkdown_1.5         mvoutlier_2.0.8
## [139] d3Network_0.5.2.1     base64enc_0.1-3       ellipse_0.3-8
```

References

- Hershberger, S. L. (2005). Tetrachoric correlation. In *Encyclopedia of statistics in behavioral science*. John Wiley & Sons.
- van der Eijk, C., & Rose, J. (2015). Risky business: Factor analysis of survey data – Assessing the probability of incorrect dimensionalisation. *PLOS ONE, 10*(3), e0118900.
