# Supplementary material for: The Position-Reputation-Information (PRI) scale of individual prestige
Source: PLoS One. 2020 Jun 25;15(6):e0234428. doi: 10.1371/journal.pone.0234428 (PMC7316272; doi:10.1371/journal.pone.0234428)
Supplement: S1 Metadata — Complete information on data sets and variables contained in S1 Data, in Word DOCX format. (DOCX) [file pone.0234428.s004.docx]

**The Position-Reputation-Information (PRI) scale of individual prestige**

Richard E.W. Berl^1*^, Alarna N. Samarasinghe^2^, Fiona M. Jordan^2,3^, Michael C. Gavin^1,3^

^1^ Department of Human Dimensions of Natural Resources, Colorado State University, Fort Collins, Colorado, United States of America

^2^ Department of Anthropology and Archaeology, University of Bristol, Bristol, United Kingdom

^3^ Max Planck Institute for the Science of Human History, Jena, Germany

* Corresponding author

E-mail: rewberl@colostate.edu (REWB)

# S4 Metadata: Data set descriptions

List of data sets included:

- **list_f**: free list data
- **list_f_r**: free list data, with replacements
- **data_f**: free list demographic data
- **data_p**: pilot study attitudinal data
- **data_p_o**: pilot study attitudinal data, ordered variables
- **data_s**: scale construction study attitudinal data
- **data_s_o**: scale construction study attitudinal data, ordered variables
- **data_s_l**: scale construction study attitudinal data, long format
- **data_t**: scale construction study triad test data
- **data_c**: scale evaluation study attitudinal data
- **data_c_o**: scale evaluation study attitudinal data, ordered variables
- **data_c_l**: scale evaluation study attitudinal data, long format
- **data_v**: criterion validity comparative attitudinal data
- **data_v_o**: criterion validity comparative attitudinal data, ordered variables
- **data_v_o**: criterion validity comparative attitudinal data, long format

### list_f

Description: Free list data for 68 participants (*n* = 48 US, 20 UK) responding to the following three prompts:

1. *List all of the words or phrases that you can think of that are related to “prestige.”*
2. *List all of the words or phrases that you can think of that describe “prestigious” people.*
3. *List all of the characteristics that you can think of that make a person “prestigious.”*

See paper text for additional detail on free list task.

Format: A list object with 68 member labels corresponding to participant identifiers (see **data_f**, below). Three elements are given for each member, each a vector of character strings corresponding to the responses given for each prompt. Vectors indices are in order of prompt and contents are in order of responses given. In CSV format, the three list elements are collapsed by the semicolon character.

Example:

> list.f$C43169

[[1]]

[1] "success" "power" "intelligence"

[4] "respect"

[[2]]

[1] "hard-working" "successful" "well-respected"

[4] "intelligent" "powerful" "influential"

[7] "knowledgeable"

[[3]]

[1] "hard-working" "driven" "assertive"

[4] "dedicated" "intelligent"

### list_f_r

Description: As for **list_f**, above, but terms have been grouped for common meaning and converted to adjective form, reducing the pool of unique items from 717 (in **list_f**) to 303. See paper text for additional detail.

Format: As in **list_f**, above.

Example:

> list.f.r$C43169

[[1]]

[1] "successful" "powerful" "intelligent" "respected"

[[2]]

[1] "hardworking" "successful" "respected"

[4] "intelligent" "powerful" "influential"

[7] "knowledgeable"

[[3]]

[1] "hardworking" "driven" "assertive" "dedicated"

[5] "intelligent"

### data_f

Description: Demographic data for participants in free list task (**list_f** and **list_f_r**, above).

Format: 68R x 41C data frame.

Variables:

1. id
   1. Type: nominal
   2. Description: unique participant identifier (anonymized)
2. country
   1. Type: nominal
   2. Description: country in which participant currently lives
   3. Levels: us (United States), uk (United Kingdom)
3. age
   1. Type: integer
   2. Description: participant age, in years
   3. Range: 18 to 50
4. age.o
   1. Type: ordinal
   2. Description: participant age, in years, binned to 10 intervals each of width 3 years
   3. Levels: [18,23] (23,28] (28,33] (33,38] (38,43] (43,48] (48,53] (53,58] (58,63] [63,68] (68,73]
5. gender
   1. Type: nominal
   2. Description: participant gender, self-reported
      1. option for non-binary gender was provided but not selected by any participants
   3. Levels: male, female
6. ethnicity
   1. Type: nominal
   2. Description: participant ethnicity, self-reported, reduced to three levels for analytical tractability
      1. US categories followed recommendations of the United States [2010 Census Race and Hispanic Origin Alternative Questionnaire Experiment](https://www.census.gov/2010census/news/press-kits/aqe/aqe.html) ([PDF](https://www.census.gov/2010census/pdf/2010_Census_Race_HO_AQE.pdf)) as results of the [2015 National Content Test](https://www.census.gov/programs-surveys/decennial-census/2020-census/planning-management/final-analysis/2015nct-race-ethnicity-analysis.html) were not yet available
      2. UK categories followed methodological guidelines of the United Kingdom Office for National Statistics ([Archived Website](http://webarchive.nationalarchives.gov.uk/20160105160709/http:/www.ons.gov.uk/ons/guide-method/measuring-equality/equality/ethnic-nat-identity-religion/ethnic-group/index.html)) ([Updated Website](https://www.ons.gov.uk/peoplepopulationandcommunity/culturalidentity/ethnicity))
      3. selections in multiple categories allowed; only those that selected more than one which included white were classified as mixed, otherwise were poc (“person of color”)
   3. Levels: white, mixed, poc
7. locality
   1. Type: ordinal
   2. Description: size of settlement in which participant spent the majority of his or her childhood
   3. Levels:
8. fewer than 5,000 people
9. 5,000 to 9,999 people
10. 10,000 to 24,999 people
11. 25,000 to 49,999 people
12. 50,000 to 99,999 people
13. 100,000 to 249,999 people
14. 250,000 or more people
15. english
    1. Type: nominal
    2. Description: participant proficiency with English language
    3. Levels: native, nonnative
16. education
    1. Type: ordinal
    2. Description: level of educational attainment of participant
    3. Levels (US / UK):
17. less than high school diploma / no formal qualifications
18. high school diploma or GED / GCSEs or equivalent
19. 2-year associate’s degree or trade school / A-levels or equivalent
20. 4-year college degree (e.g. bachelor’s degree) / 3-year/university degree (e.g. bachelor’s degree) or equivalent
21. advanced degree beyond 4/3-year degree (e.g. master’s, PhD, medical doctor, law degree) or equivalent
22. occupation
    1. Type: nominal
    2. Description: occupational category of participant
       1. categories from the [U.S. Current Population Survey](https://www.bls.gov/cps/cpsoccind.htm) by the United States Bureau of Labor Statistics
    3. Levels:
23. student
24. homemaker or stay-at-home parent
25. production, transportation, or material moving (e.g. processing, assembly, driving)
26. natural resource extraction, construction, or maintenance (e.g. farming, forestry, skilled trades, repair)
27. sales or office (e.g. retail, marketing, clerical)
28. service (e.g. healthcare support, police and corrections, food service, personal care)
29. management or professional (e.g. finance, engineering, education, research, law, medicine)
30. income
    1. Type: ordinal
    2. Description: approximate gross annual income of participant’s household
    3. Levels (US / UK):
31. less than $10,000 / less than £11,000
32. $10,000 to $29,999 / £11,000 to £24,999
33. $30,000 to $49,999 / 25,000 to £39,999
34. $50,000 to $69,999 / £40,000 to £54,999
35. $70,000 to $89,999 / £55,000 to £69,999
36. $90,000 to $109,999 / £70,000 to £84,999
37. $110,000 to $129,999 / £85,000 to £99,999
38. $130,000 to $149,999 / £100,000 to £114,999
39. $150,000 to $199,999 / £115,000 to £129,999
40. $200,000 to $249,999 / £130,000 to £149,999
41. $250,000 or more / £150,000 or more

### data_p

Description: Attitudinal and demographic data collected from participants in the speech evaluation pilot study. See paper text for additional detail.

Format: 281R x 24C data frame.

Variables:

1. id
   1. Type: nominal
   2. Description: unique participant identifier (anonymized)
2. friendly through 16. wealthy
   1. Type: integer
   2. Description: Likert-type attitudinal item ratings by participant for a particular speaker and information type
   3. Levels: 1 (low) through 7 (high)
   4. List of variables: friendly, reliable, ambitious, sincere, intelligent, sociable, hardworking, easygoing, prestigious, reputable, confident, pleasant, interesting, attractive
3. country
   1. Type: nominal
   2. Description: country in which participant currently lives
   3. Levels: us (United States), uk (United Kingdom)
4. accent
   1. Type: nominal
   2. Description: regional accent of speaker, relative to participant’s country of residence
   3. Levels:
      1. standard (in UK: Southeast England; in US: American West [Urban])
      2. nonstandard (in UK: Northwest England; in US: New York City [Long Island])
5. first
   1. Type: nominal
   2. Description: which of the two recordings was presented first in this trial
   3. Levels: standard, nonstandard
6. info
   1. Type: nominal
   2. Description: which type of information was being said by the speaker
      1. patterned after: Mesoudi, A., Whiten, A., & Dunbar, R. (2006). A bias for social information in human cultural transmission. British Journal of Psychology, 97(3), 405-423.
   3. Levels: social, gossip
      1. “social” text: *One morning, Nancy's alarm clock broke and she overslept. When she woke up, she realized that she was late for an important lecture. She got dressed as quickly as she could, left the house and ran to the lecture hall. When she got there, the lecture hall was empty. Nancy had missed the lecture.*
      2. “gossip” text: *Nancy is having an affair with her married college professor. Nancy recently became pregnant with the professor's child. The professor promised Nancy that he would leave his wife, but since Nancy told him she was pregnant, the professor refused to see her. So Nancy told the professor's wife about the affair. The professor's wife was so upset that she left the professor.*
7. age
   1. Type: integer
   2. Description: participant age, in years
   3. Range: 18 to 64
8. age.o
   1. Type: ordinal
   2. Description: participant age, in years, binned to 10 intervals each of width 3 years
   3. Levels: [18,23] (23,28] (28,33] (33,38] (38,43] (43,48] (48,53] (53,58] (58,63] (63,68] (68,73]
9. gender
   1. Type: nominal
   2. Description: participant gender, self-reported
      1. participants asked to give a binary response
   3. Levels: male, female
10. english
    1. Type: nominal
    2. Description: participant proficiency with English language
    3. Levels: native, nonnative

### data_s

Description: Attitudinal and demographic data collected from participants in the scale construction study. See paper text for additional detail.

Format: 1212R x 33C data frame.

Variables:

1. id
   1. Type: nominal
   2. Description: unique participant identifier (anonymized)
2. active through 22. wealthy
   1. Type: integer
   2. Description: Likert-type attitudinal item ratings by participant for a particular speaker
   3. Levels: 1 (low) through 7 (high)
   4. List of variables: active, aggressive, ambitious, clear, comforting, confident, educated, enthusiastic, friendly, good.natured, hardworking, high.social.status, intelligent, kind, powerful, prestigious, reputable, respected, successful, warm, wealthy
3. accent
   1. Type: nominal
   2. Description: regional accent of speaker
   3. Levels:
      1. england39 (Received Pronunciation)
      2. colorado (American West [Urban])
      3. england60 (Northwest England)
      4. northcarolina14 (American Inland South [Blue-Collar])
4. country
   1. Type: nominal
   2. Description: country in which participant currently lives
   3. Levels: us (United States), uk (United Kingdom)
5. age
   1. Type: integer
   2. Description: participant age, in years
   3. Range: 18 to 70
6. age.o
   1. Type: ordinal
   2. Description: participant age, in years, binned to 10 intervals each of width 3 years
   3. Levels: [18,23] (23,28] (28,33] (33,38] (38,43] (43,48] (48,53] (53,58] (58,63] (63,68] (68,73]
7. gender
   1. Type: nominal
   2. Description: participant gender, self-reported
      1. option for non-binary gender was provided but not selected by any participants
   3. Levels: male, female
8. ethnicity
   1. Type: nominal
   2. Description: participant ethnicity, self-reported, reduced to three levels for analytical tractability
      1. US categories followed recommendations of the United States [2010 Census Race and Hispanic Origin Alternative Questionnaire Experiment](https://www.census.gov/2010census/news/press-kits/aqe/aqe.html) ([PDF](https://www.census.gov/2010census/pdf/2010_Census_Race_HO_AQE.pdf)) as results of the [2015 National Content Test](https://www.census.gov/programs-surveys/decennial-census/2020-census/planning-management/final-analysis/2015nct-race-ethnicity-analysis.html) were not yet available
      2. UK categories followed methodological guidelines of the United Kingdom Office for National Statistics ([Archived Website](http://webarchive.nationalarchives.gov.uk/20160105160709/http:/www.ons.gov.uk/ons/guide-method/measuring-equality/equality/ethnic-nat-identity-religion/ethnic-group/index.html)) ([Updated Website](https://www.ons.gov.uk/peoplepopulationandcommunity/culturalidentity/ethnicity))
      3. selections in multiple categories allowed; only those that selected more than one which included white were classified as mixed, otherwise were poc (“person of color”)
   3. Levels: white, mixed, poc
9. locality
   1. Type: ordinal
   2. Description: size of settlement in which participant spent the majority of his or her childhood
   3. Levels:
10. fewer than 5,000 people
11. 5,000 to 9,999 people
12. 10,000 to 24,999 people
13. 25,000 to 49,999 people
14. 50,000 to 99,999 people
15. 100,000 to 249,999 people
16. 250,000 or more people
17. english
    1. Type: nominal
    2. Description: participant proficiency with English language
    3. Levels: native, nonnative
18. education
    1. Type: ordinal
    2. Description: level of educational attainment of participant
    3. Levels (US / UK):
19. less than high school diploma / no formal qualifications
20. high school diploma or GED / GCSEs or equivalent
21. 2-year associate’s degree or trade school / A-levels or equivalent
22. 4-year college degree (e.g. bachelor’s degree) / 3-year/university degree (e.g. bachelor’s degree) or equivalent
23. advanced degree beyond 4/3-year degree (e.g. master’s, PhD, medical doctor, law degree) or equivalent
24. occupation
    1. Type: nominal
    2. Description: occupational category of participant
       1. categories from the [U.S. Current Population Survey](https://www.bls.gov/cps/cpsoccind.htm) by the United States Bureau of Labor Statistics
    3. Levels:
25. student
26. homemaker or stay-at-home parent
27. production, transportation, or material moving (e.g. processing, assembly, driving)
28. natural resource extraction, construction, or maintenance (e.g. farming, forestry, skilled trades, repair)
29. sales or office (e.g. retail, marketing, clerical)
30. service (e.g. healthcare support, police and corrections, food service, personal care)
31. management or professional (e.g. finance, engineering, education, research, law, medicine)
32. income
    1. Type: ordinal
    2. Description: approximate gross annual income of participant’s household
    3. Levels (US / UK):
33. less than $10,000 / less than £11,000
34. $10,000 to $29,999 / £11,000 to £24,999
35. $30,000 to $49,999 / 25,000 to £39,999
36. $50,000 to $69,999 / £40,000 to £54,999
37. $70,000 to $89,999 / £55,000 to £69,999
38. $90,000 to $109,999 / £70,000 to £84,999
39. $110,000 to $129,999 / £85,000 to £99,999
40. $130,000 to $149,999 / £100,000 to £114,999
41. $150,000 to $199,999 / £115,000 to £129,999
42. $200,000 to $249,999 / £130,000 to £149,999
43. $250,000 or more / £150,000 or more

### data_s_o

Description: As for **data_s**, above, but with attitudinal item ratings (variables 2 through 22) formatted as ordinal variables.

Format: As for **data_s**, above.

Variables: As in **data_s**, above.

### data_s_l

Description: As for **data_s**, above, but with attitudinal item ratings in long format.

Format: 25452R x 14C data frame.

Variables:

1. id through 12. income
   1. Identifier and demographic variables as described for **data_s**, above
   2. List of variables: id, accent, country, age, age.o, gender, ethnicity, locality, english, education, occupation, income
2. item
   1. Type: nominal
   2. Description: attitudinal variables as described for **data_s**, above
   3. Levels: prestigious, wealthy, high.social.status, powerful, respected, educated, hardworking, successful, intelligent, reputable, ambitious, friendly, kind, good.natured, warm, comforting, aggressive, active, confident, enthusiastic, clear
3. score
   1. Type: integer
   2. Description: Likert-type attitudinal item rating by participant for a particular speaker and variable
   3. Levels: 1 (low) through 7 (high)

### data_t

Description: Triad test data collected from participants in the scale construction study. See paper text for additional detail.

Format: 16940R x 13C data frame.

Variables:

1. id
   1. Type: nominal
   2. Description: unique participant identifier (anonymized), consistent with attitudinal data from same study
2. country
   1. Type: nominal
   2. Description: country in which participant currently lives
   3. Levels: us (United States), uk (United Kingdom)
3. prestigious through 13. ambitious
   1. Type: binary
   2. Description: associations between variables, of the three presented in each comparison
   3. Levels: 0 (unlike the others), 1 (like the other), NA (not presented)
   4. List of variables: prestigious, wealthy, high.social.status, powerful, respected, educated, hardworking, successful, intelligent, reputable, ambitious

Example:

> head(data.t)

id country prestigious wealthy high.social.status

1 4711016067 us NA NA NA

2 4711016067 us NA NA NA

3 4711016067 us NA NA NA

4 4711016067 us NA NA NA

5 4711016067 us NA NA NA

6 4711016067 us 1 NA NA

powerful respected educated hardworking successful

1 NA NA NA 0 NA

2 NA 0 1 NA NA

3 NA 1 NA 1 NA

4 NA NA NA 1 NA

5 NA NA 1 NA NA

6 NA NA NA NA NA

intelligent reputable ambitious

1 1 1 NA

2 1 NA NA

3 NA NA NA

4 0 1 NA

5 NA 1 0

6 NA NA 0

### data_c

Description: Attitudinal and demographic data collected from participants in the scale evaluation study. See paper text for additional detail.

Format: 2950R x 36C data frame.

Variables:

1. id
   1. Type: nominal
   2. Description: unique participant identifier (anonymized)
2. active through 25. wealthy
   1. Type: integer
   2. Description: Likert-type attitudinal item ratings by participant for a particular speaker
   3. Levels: 1 (low) through 7 (high)
   4. List of variables: active, aggressive, ambitious, clear, comforting, confident, driven, educated, enthusiastic, friendly, good.natured, hardworking, high.social.status, intelligent, kind, powerful, prestigious, reputable, respected, skilled, successful, talented, warm, wealthy
3. accent
   1. Type: nominal
   2. Description: regional accent of speaker
   3. Levels:
      1. colorado (American West [Urban])
      2. wyoming4 (American West [Rural])
      3. oklahoma16 (Midland)
      4. illinois10 (Inland North)
      5. northcarolina10 (American Inland South [White-Collar])
      6. northcarolina14 (American Inland South [Blue-Collar])
      7. pennsylvania9 (Mid-Atlantic)
      8. newyork11 (New York City)
      9. england39 (Received Pronunciation)
      10. england32 (Southwest England)
      11. england65 (Southeast England)
      12. england57 (Yorkshire)
      13. england60 (Northwest England)
      14. scotland9 (Scotland)
      15. ireland17 (Ireland)
      16. wales (Wales)
4. country
   1. Type: nominal
   2. Description: country in which participant currently lives
   3. Levels: us (United States), uk (United Kingdom)
5. age
   1. Type: integer
   2. Description: participant age, in years
   3. Range: 18 to 64
6. age.o
   1. Type: ordinal
   2. Description: participant age, in years, binned to 10 intervals each of width 3 years
   3. Levels: [18,23] (23,28] (28,33] (33,38] (38,43] (43,48] (48,53] (53,58] (58,63] (63,68] (68,73]
7. gender
   1. Type: nominal
   2. Description: participant gender, self-reported
   3. Levels: male, female, nonbinary
8. ethnicity
   1. Type: nominal
   2. Description: participant ethnicity, self-reported, reduced to three levels for analytical tractability
      1. US categories followed recommendations of the United States [2010 Census Race and Hispanic Origin Alternative Questionnaire Experiment](https://www.census.gov/2010census/news/press-kits/aqe/aqe.html) ([PDF](https://www.census.gov/2010census/pdf/2010_Census_Race_HO_AQE.pdf)) as results of the [2015 National Content Test](https://www.census.gov/programs-surveys/decennial-census/2020-census/planning-management/final-analysis/2015nct-race-ethnicity-analysis.html) were not yet available
      2. UK categories followed methodological guidelines of the United Kingdom Office for National Statistics ([Archived Website](http://webarchive.nationalarchives.gov.uk/20160105160709/http:/www.ons.gov.uk/ons/guide-method/measuring-equality/equality/ethnic-nat-identity-religion/ethnic-group/index.html)) ([Updated Website](https://www.ons.gov.uk/peoplepopulationandcommunity/culturalidentity/ethnicity))
      3. selections in multiple categories allowed; only those that selected more than one which included white were classified as mixed, otherwise were poc (“person of color”)
   3. Levels: white, mixed, poc
9. locality
   1. Type: ordinal
   2. Description: size of settlement in which participant spent the majority of his or her childhood
   3. Levels:
10. fewer than 5,000 people
11. 5,000 to 9,999 people
12. 10,000 to 24,999 people
13. 25,000 to 49,999 people
14. 50,000 to 99,999 people
15. 100,000 to 249,999 people
16. 250,000 or more people
17. english
    1. Type: nominal
    2. Description: participant proficiency with English language
    3. Levels: native, nonnative
18. education
    1. Type: ordinal
    2. Description: level of educational attainment of participant
    3. Levels (US / UK):
19. less than high school diploma / no formal qualifications
20. high school diploma or GED / GCSEs or equivalent
21. 2-year associate’s degree or trade school / A-levels or equivalent
22. 4-year college degree (e.g. bachelor’s degree) / 3-year/university degree (e.g. bachelor’s degree) or equivalent
23. advanced degree beyond 4/3-year degree (e.g. master’s, PhD, medical doctor, law degree) or equivalent
24. occupation
    1. Type: nominal
    2. Description: occupational category of participant
       1. categories from the [U.S. Current Population Survey](https://www.bls.gov/cps/cpsoccind.htm) by the United States Bureau of Labor Statistics
    3. Levels:
25. student
26. homemaker or stay-at-home parent
27. production, transportation, or material moving (e.g. processing, assembly, driving)
28. natural resource extraction, construction, or maintenance (e.g. farming, forestry, skilled trades, repair)
29. sales or office (e.g. retail, marketing, clerical)
30. service (e.g. healthcare support, police and corrections, food service, personal care)
31. management or professional (e.g. finance, engineering, education, research, law, medicine)
32. income
    1. Type: ordinal
    2. Description: approximate gross annual income of participant’s household
    3. Levels (US / UK):
33. less than $10,000 / less than £11,000
34. $10,000 to $29,999 / £11,000 to £24,999
35. $30,000 to $49,999 / 25,000 to £39,999
36. $50,000 to $69,999 / £40,000 to £54,999
37. $70,000 to $89,999 / £55,000 to £69,999
38. $90,000 to $109,999 / £70,000 to £84,999
39. $110,000 to $129,999 / £85,000 to £99,999
40. $130,000 to $149,999 / £100,000 to £114,999
41. $150,000 to $199,999 / £115,000 to £129,999
42. $200,000 to $249,999 / £130,000 to £149,999
43. $250,000 or more / £150,000 or more

### data_c_o

Description: As for **data_c**, above, but with attitudinal item ratings (variables 2 through 25) formatted as ordinal variables.

Format: As for **data_c**, above.

Variables: As in **data_c**, above.

### data_c_l

Description: As for **data_c**, above, but with attitudinal item ratings in long format.

Format: 70800R x 14C data frame.

Variables:

1. id through 12. income
   1. Identifier and demographic variables as described for **data_c**, above
   2. List of variables: id, accent, country, age, age.o, gender, ethnicity, locality, english, education, occupation, income
2. item
   1. Type: nominal
   2. Description: attitudinal variables as described for **data_c**, above
   3. Levels: prestigious, wealthy, high.social.status, powerful, respected, educated, hardworking, successful, intelligent, reputable, ambitious, talented, driven, skilled, friendly, kind, good.natured, warm, comforting, aggressive, active, confident, enthusiastic, clear
3. score
   1. Type: integer
   2. Description: Likert-type attitudinal item rating by participant for a particular speaker and variable
   3. Levels: 1 (low) through 7 (high)

### data_v

Description: Attitudinal data collected from participants for comparative criterion validity assessment with prestige-dominance scale of Cheng et al. (2010). See paper text for additional detail. Detailed demographics for participants and speakers are not included as the authors are preparing parts of this data set for more thorough analysis in a different study.

Format: 287R x 38C data frame.

Variables:

1. id
   1. Type: nominal
   2. Description: unique participant identifier (anonymized)
2. accent
   1. Type: nominal
   2. Description: relative prestige level of speaker’s regional accent
      1. Note: Recordings done by authors using different speakers and texts than in prior studies. Texts were consistent and recordings were randomized in presentation order and accent between participants.
   3. Levels:
      1. high (Received Pronunciation in both US and UK)
      2. low (in UK: Northwest England; in US: Inland South)
3. country
   1. Type: nominal
   2. Description: country in which participant currently lives
   3. Levels: us (United States), uk (United Kingdom)
4. prestigious through 20. enthusiastic
   1. Type: integer
   2. Description: Likert-type attitudinal ratings by participant for a particular speaker, for PRI scale items (4-11) and additional solidarity (12-16) and dynamism (17-20) domains
   3. Levels: 1 (low) through 6 (high)
   4. List of variables: prestigious, wealthy, powerful, high.social.status, reputable, respected, educated, intelligent, friendly, kind, good.natured, warm, comforting, aggressive, active, confident, enthusiastic
5. P.People.respect.and.admire.him through 37. D.People.are.afraid.of.him
   1. Type: integer
   2. Description: Likert-type attitudinal ratings by participant for a particular speaker, for Cheng et. al (2010) prestige-dominance scale prestige items (21-29) and dominance items (30-37)
   3. Levels: 1 (low) through 6 (high)
   4. List of variables: P.People.respect.and.admire.him, P.People.do.NOT.want.to.be.like.him, P.People.always.expect.him.to.be.successful, P.People.do.NOT.value.his.opinion, P.He.is.held.in.high.esteem.by.people, P.His.unique.talents.and.abilities.are.recognized.by.other.people, P.He.is.considered.an.expert.on.some.matters.by.people, P.People.seek.his.advice.on.a.variety.of.matters, P.Other.people.do.NOT.enjoy.hanging.out.with.him, D.He.enjoys.having.control.over.other.people, D.He.often.tries.to.get.his.own.way.regardless.of.what.other.people.may.want,

D.He.is.willing.to.use.aggressive.tactics.to.get.his.way, D.He.tries.to.control.others.rather.than.permit.them.to.control.him, D.He.does.NOT.have.a.forceful.or.dominant.personality, D.People.know.it.is.better.to.let.him.have.his.way, D.He.does.NOT.enjoy.having.authority.over.other.people, D.People.are.afraid.of.him

### data_v_o

Description: As for **data_v**, above, but with attitudinal item ratings (variables 4 through 37) formatted as ordinal variables.

Format: As for **data_v**, above.

Variables: As in **data_v**, above.

### data_v_l

Description: As for **data_v**, above, but with attitudinal item ratings in long format.

Format: 9758R x 5C data frame.

Variables:

1. id through 3. country
   1. Identifier and demographic variables as described for **data_v** above
   2. List of variables: id, accent, country
2. item
   1. Type: nominal
   2. Description: attitudinal variables as described for **data_v**, above
   3. Levels: prestigious, wealthy, powerful, high.social.status, reputable, respected, educated, intelligent, friendly, kind, good.natured, warm, comforting, aggressive, active, confident, enthusiastic, P.People.respect.and.admire.him,

P.People.do.NOT.want.to.be.like.him, P.People.always.expect.him.to.be.successful, P.People.do.NOT.value.his.opinion, P.He.is.held.in.high.esteem.by.people, P.His.unique.talents.and.abilities.are.recognized.by.other.people, P.He.is.considered.an.expert.on.some.matters.by.people, P.People.seek.his.advice.on.a.variety.of.matters, P.Other.people.do.NOT.enjoy.hanging.out.with.him, D.He.enjoys.having.control.over.other.people, D.He.often.tries.to.get.his.own.way.regardless.of.what.other.people.may.want,

D.He.is.willing.to.use.aggressive.tactics.to.get.his.way, D.He.tries.to.control.others.rather.than.permit.them.to.control.him, D.He.does.NOT.have.a.forceful.or.dominant.personality, D.People.know.it.is.better.to.let.him.have.his.way, D.He.does.NOT.enjoy.having.authority.over.other.people, D.People.are.afraid.of.him

1. score
   1. Type: integer
   2. Description: Likert-type attitudinal item rating by participant for a particular speaker and variable
   3. Levels: 1 (low) through 6 (high)
