# Supplementary material for: The Position-Reputation-Information (PRI) scale of individual prestige
Source: PLoS One. 2020 Jun 25;15(6):e0234428. doi: 10.1371/journal.pone.0234428 (PMC7316272; doi:10.1371/journal.pone.0234428)
Supplement: S1 Table — Demographic information for speakers used in scale construction and scale evaluation studies, in Word DOCX format. Contains information on speaker accent, recording ID (if from IDEA, see Acknowledgements), country, state, age (in years) at time of recording, place of birth, place raised (for majority of childhood), gender, ethnicity, occupation, education, other places lived, other possible influences on speech, identity of recorder, and recording date. (DOCX) [file pone.0234428.s005.docx]

**The Position-Reputation-Information (PRI) scale of individual prestige**

Richard E.W. Berl^1*^, Alarna N. Samarasinghe^2^, Fiona M. Jordan^2,3^, Michael C. Gavin^1,3^

^1^ Department of Human Dimensions of Natural Resources, Colorado State University, Fort Collins, Colorado, United States of America

^2^ Department of Anthropology and Archaeology, University of Bristol, Bristol, United Kingdom

^3^ Max Planck Institute for the Science of Human History, Jena, Germany

* Corresponding author

E-mail: rewberl@colostate.edu (REWB)

# S5 Table: Speaker demographics

|  | **Accent/Dialect Region** | **Recording ID** | **Country** | **State/County** |
| --- | --- | --- | --- | --- |
| 1 | American West (Urban) | colorado | USA | Colorado |
| 2 | American West (Rural) | wyoming4 | USA | Wyoming |
| 3 | Midland | oklahoma16 | USA | Oklahoma |
| 4 | Inland North | illinois10 | USA | Illinois |
| 5 | American Inland South (White-Collar) | northcarolina10 | USA | North Carolina |
| 6 | American Inland South (Blue-Collar) | northcarolina14 | USA | North Carolina |
| 7 | Mid-Atlantic | pennsylvania9 | USA | Pennsylvania |
| 8 | New York City | newyork11 | USA | New York |
| 9 | Received Pronunciation | england39 | UK | Berkshire |
| 10 | Southwest England | england32 | UK | Devon |
| 11 | Southeast England | england65 | UK | Surrey |
| 12 | Yorkshire | england57 | UK | West Yorkshire |
| 13 | Northwest England | england60 | UK | Lancashire |
| 14 | Scotland | scotland9 | UK | Renfrewshire |
| 15 | Ireland | ireland17 | UK | Dublin |
| 16 | Wales | wales | UK | Swansea |

|  | **Age** | **Place of Birth** | **Place Raised** |
| --- | --- | --- | --- |
| 1 | 48 | Boulder, Colorado | Boulder and Montrose, Colorado (equally) |
| 2 | 31 | Basin, Wyoming | Basin, Wyoming |
| 3 | 55 | Oklahoma City, Oklahoma | Unknown [Missing Data] |
| 4 | 51 | Chicago, Illinois | Chicago, Illinois |
| 5 | 42 | Burnsville, North Carolina | Burnsville, North Carolina |
| 6 | 42 | Tennessee | Tennessee and Burnsville, North Carolina (after age 5) |
| 7 | 59 | Uniontown, Pennsylvania | Clifton Heights and Swarthmore, Pennsylvania |
| 8 | 48 | Brooklyn, New York City, New York | Rosedale, Queens and Long Island, New York City, New York |
| 9 | 52 | Maidenhead, Berkshire, England | Maidenhead, Berkshire, England; West Sussex, England (age 11-17); London, England (1 year); East Sussex, England (3 years) |
| 10 | 49 | Appledore, North Devon, Devon, England | Appledore and Wesley, North Devon, Devon, England |
| 11 | 50 | Woking, Surrey, England | Woking and Addlestone, Surrey, England |
| 12 | 52 | Leeds, West Yorkshire, England | Leeds, West Yorkshire, England |
| 13 | 59 | Platt Bridge, Wigan, Lancashire, England | Platt Bridge, Wigan, Lancashire, England |
| 14 | 33 | Johnstone, Renfrewshire, Scotland | Johnstone, Renfrewshire, Scotland |
| 15 | 38 | Dublin, Ireland | Carlow, Ireland |
| 16 | 45 | Morriston, Wales | Morriston, Wales |

|  | **Gender** | **Ethnicity** | **Occupation** | **Education** |
| --- | --- | --- | --- | --- |
| 1 | M | White | Academic Advisor | MA |
| 2 | M | Caucasian | Attorney | JD |
| 3 | M | Caucasian | Mayor | MBA |
| 4 | M | Caucasian | Financial Advisor | BA |
| 5 | M | Caucasian | Attorney | JD |
| 6 | M | Caucasian | Surveyor | High School |
| 7 | M | Caucasian | Maintenance Mechanic | High School |
| 8 | M | Caucasian (Italian-American) | Fashion/Retail Executive | University |
| 9 | M | White | Actor, Teacher | University |
| 10 | M | White | Fitter | None |
| 11 | M | White | Local Governmental Officer | A-Level |
| 12 | M | White | Plumber | "Some" |
| 13 | M | White | Engineer | Until 15 |
| 14 | M | Caucasian | Drama Student | University Student |
| 15 | M | Irish/Caucasian | Professor | MA |
| 16 | M | White/Caucasian | Academic | PhD |

|  | **Other Places Lived** | **Other Influences on Speech** |
| --- | --- | --- |
| 1 | Dublin, Ireland (1.5 years) | None |
| 2 | None | None |
| 3 | New York (university); Texas (8 months) | Broadcasting training and experience |
| 4 | Minneapolis-Saint Paul, Minnesota (10 years) | Theater actor |
| 5 | Davidson, North Carolina (university); Chapel Hill, North Carolina (law school) | None |
| 6 | Huntsville, Texas (5 years) | Theater actor |
| 7 | None | Air Force medic |
| 8 | Florence, Italy (1 year) | None |
| 9 | London, England; United States; Brighton, Sussex, England (8 years); Bristol, England (5 years) | Actor |
| 10 | Purbright, Hampshire, England (7 years) | Former soldier |
| 11 | Brighton, Sussex, England (25 years) | Worked overseas; Telephone conflict resolution |
| 12 | London, England; Halifax, West Yorkshire, England | None |
| 13 | None | Interest in Wigan dialect |
| 14 | None | Drama student |
| 15 | Philadelphia, Pennsylvania (4 years); Pensacola, Florida (6 years+) | Professor of voice |
| 16 | Leicester; Sheffield; Bristol, England | Has lived outside of Wales for the past 30 years |

|  | **Recorded By** | **Date of Recording** |
| --- | --- | --- |
| 1 | Richard E.W. Berl | 2/25/16 |
| 2 | Lou Anne Wright | 8/24/11 |
| 3 | Ben Corbett | 7/26/13 |
| 4 | Joseph Papke | 4/29/05 |
| 5 | Pat Toole | 3/01 |
| 6 | Pat Toole | 8/01 |
| 7 | Patricia Helsel | 6/22/10 |
| 8 | Daydrie Hague | 6/02 |
| 9 | Paul Meier | 01 |
| 10 | Paul Meier | 01 |
| 11 | Marina Tyndall | 6/07 |
| 12 | Paul Meier | 1/04 |
| 13 | Jeff Unsworth | 12/11/05 |
| 14 | Ros Steen | 01 |
| 15 | Kris Danford | 12/8/14 |
| 16 | Alarna N. Samarasinghe | 6/14/16 |
