# Supplementary material for: The Position-Reputation-Information (PRI) scale of individual prestige
Source: PLoS One. 2020 Jun 25;15(6):e0234428. doi: 10.1371/journal.pone.0234428 (PMC7316272; doi:10.1371/journal.pone.0234428)
Supplement: Attachment — Submitted filename: R2R.docx [file pone.0234428.s006.docx]

November 6, 2019

Dr. Meghan Byrne and Dr. George Vousden

Senior Editors, Behavioral and Social Sciences, Neuroscience, Mental Health

PLOS ONE

Dear Dr. Byrne and Dr. Vousden,

We submit to you our manuscript titled “*The Position-Reputation-Information (PRI) scale of individual prestige*” for consideration for publication in PLOS ONE.

This research article reports the development and validation of a new scale of individual prestige. The concept of prestige is central to a wide range of social science disciplines, including sociology, economics, psychology, and anthropology. Surprisingly, despite the importance of the concept, no reliable method currently exists for the measurement of an individual’s prestige. Here, we construct a valid and reliable scale of individual prestige using data-driven and theory-neutral approaches, which allowed participants to have a major role in determining and validating its content and structure.

The final version of our prestige scale is composed of seven items in three subscales: *wealthy*, *powerful*, and *high social status* in the “position” subscale; *reputable* and *respected* in the “reputation” subscale, and *educated* and *intelligent* in the “information” subscale. We consistently found this structure across two separate sources of data and it is well-supported by indices of goodness of fit, scale validity, and scale reliability. We have ensured that the process of scale development is openly accessible, repeatable, and extensible across academic disciplines and cross-cultural applications. This includes a thorough description of all methods used and the inclusion of all data and code used to run analyses. The PRI scale enables the study of prestige using a common scale, which we hope encourages fruitful conversation across disciplinary boundaries in the social sciences, and leads to a more holistic understanding of the foundations of prestige and its effects on the dynamic processes of cultural evolution. Publishing open access in PLOS ONE is the ideal way for us to share this important scale, our novel methods, and our data sets with a broad, interdisciplinary scientific audience.

A prior version of this manuscript was reviewed by a PLOS ONE editor and rejected on the grounds of length and the arrangement of the manuscript sections (Methods were presented at the end of the manuscript). We note that neither of the stated reasons are criteria for rejection under PLOS ONE submission guidelines (“Manuscripts can be any length” and “[middle section] elements can be renamed as needed and presented in any order”) but believe that our revisions have adequately addressed these concerns by rearranging the manuscript and moving much of the detailed methodological description to a supporting appendix. We are hopeful that our resubmission can be evaluated on its content by a new editor.

We suggest Alex Mesoudi as an appropriate editor for this work given his extensive experience in the field of cultural evolution, or an editor with experience in psychometrics as well as social learning, social behavior, or psychological anthropology, such as Julie Gros-Louis or Brock Bastian. We have no opposed reviewers. The submission of this manuscript has been approved by all co-authors and it is not under consideration by any other outlets. A preprint of the article prior to revisions is available on SocArXiv (https://osf.io/preprints/socarxiv/426n8/).

Thank you for your time and for considering the publication of our manuscript.

Sincerely yours,

Richard E.W. Berl

Alarna N. Samarasinghe

Fiona M. Jordan

Michael C. Gavin
